# Olaparib in patients with metastatic castration-resistant prostate cancer with DNA repair gene aberrations (TOPARP-B): a multicentre, open-label, randomised, phase 2 trial

**DOI:** 10.1016/S1470-2045(19)30684-9

**Published:** 2020-01

**Authors:** Joaquin Mateo, Nuria Porta, Diletta Bianchini, Ursula McGovern, Tony Elliott, Robert Jones, Isabel Syndikus, Christy Ralph, Suneil Jain, Mohini Varughese, Omi Parikh, Simon Crabb, Angus Robinson, Duncan McLaren, Alison Birtle, Jacob Tanguay, Susana Miranda, Ines Figueiredo, George Seed, Claudia Bertan, Penny Flohr, Berni Ebbs, Pasquale Rescigno, Gemma Fowler, Ana Ferreira, Ruth Riisnaes, Rita Pereira, Andra Curcean, Robert Chandler, Matthew Clarke, Bora Gurel, Mateus Crespo, Daniel Nava Rodrigues, Shahneen Sandhu, Aude Espinasse, Peter Chatfield, Nina Tunariu, Wei Yuan, Emma Hall, Suzanne Carreira, Johann S de Bono

**Affiliations:** a The Institute of Cancer Research and The Royal Marsden NHS Foundation Trust, London, UK; b The Institute of Cancer Research, London, UK; c University College Hospital, University College London Hospitals NHS Foundation Trust, London, UK; d The Christie NHS Foundation Trust, Manchester, UK; e University of Glasgow and Beatson West of Scotland Cancer Centre, Glasgow, UK; f The Clatterbridge Cancer Centre, Wirral, UK; g St James's Institute of Oncology, University of Leeds, Leeds, UK; h Queen's University, Belfast, UK; i Musgrove Park Hospital, Taunton, UK; j Royal Blackburn Hospital, Blackburn, UK; k University of Southampton, Southampton, UK; l Royal Sussex County Hospital, Brighton, UK; m Western General Hospital, Edinburgh, UK; n Royal Lancaster Infirmary, Lancaster, UK; o Velindre Cancer Centre, Cardiff, UK; p Vall d'Hebron Institute of Oncology (VHIO), Barcelona, Spain; q Peter McCallum Cancer Center, Melbourne, VIC, Australia

## Abstract

**Background:**

Metastatic castration-resistant prostate cancer is enriched in DNA damage response (DDR) gene aberrations. The TOPARP-B trial aims to prospectively validate the association between DDR gene aberrations and response to olaparib in metastatic castration-resistant prostate cancer.

**Methods:**

In this open-label, investigator-initiated, randomised phase 2 trial following a selection (or pick-the-winner) design, we recruited participants from 17 UK hospitals. Men aged 18 years or older with progressing metastatic castration-resistant prostate cancer previously treated with one or two taxane chemotherapy regimens and with an Eastern Cooperative Oncology Group performance status of 2 or less had tumour biopsies tested with targeted sequencing. Patients with DDR gene aberrations were randomly assigned (1:1) by a computer-generated minimisation method, with balancing for circulating tumour cell count at screening, to receive 400 mg or 300 mg olaparib twice daily, given continuously in 4-week cycles until disease progression or unacceptable toxicity. Neither participants nor investigators were masked to dose allocation. The primary endpoint of confirmed response was defined as a composite of all patients presenting with any of the following outcomes: radiological objective response (as assessed by Response Evaluation Criteria in Solid Tumors 1.1), a decrease in prostate-specific antigen (PSA) of 50% or more (PSA50) from baseline, or conversion of circulating tumour cell count (from ≥5 cells per 7·5 mL blood at baseline to <5 cells per 7·5 mL blood). A confirmed response in a consecutive assessment after at least 4 weeks was required for each component. The primary analysis was done in the evaluable population. If at least 19 (43%) of 44 evaluable patients in a dose cohort responded, then the dose cohort would be considered successful. Safety was assessed in all patients who received at least one dose of olaparib. This trial is registered at ClinicalTrials.gov, NCT01682772. Recruitment for the trial has completed and follow-up is ongoing.

**Findings:**

711 patients consented for targeted screening between April 1, 2015, and Aug 30, 2018. 161 patients had DDR gene aberrations, 98 of whom were randomly assigned and treated (49 patients for each olaparib dose), with 92 evaluable for the primary endpoint (46 patients for each olaparib dose). Median follow-up was 24·8 months (IQR 16·7–35·9). Confirmed composite response was achieved in 25 (54·3%; 95% CI 39·0–69·1) of 46 evaluable patients in the 400 mg cohort, and 18 (39·1%; 25·1–54·6) of 46 evaluable patients in the 300 mg cohort. Radiological response was achieved in eight (24·2%; 11·1–42·3) of 33 evaluable patients in the 400 mg cohort and six (16·2%; 6·2–32·0) of 37 in the 300 mg cohort; PSA50 response was achieved in 17 (37·0%; 23·2–52·5) of 46 and 13 (30·2%; 17·2–46·1) of 43; and circulating tumour cell count conversion was achieved in 15 (53·6%; 33·9–72·5) of 28 and 13 (48·1%; 28·7–68·1) of 27. The most common grade 3–4 adverse event in both cohorts was anaemia (15 [31%] of 49 patients in the 300 mg cohort and 18 [37%] of 49 in the 400 mg cohort). 19 serious adverse reactions were reported in 13 patients. One death possibly related to treatment (myocardial infarction) occurred after 11 days of treatment in the 300 mg cohort.

**Interpretation:**

Olaparib has antitumour activity against metastatic castration-resistant prostate cancer with DDR gene aberrations, supporting the implementation of genomic stratification of metastatic castration-resistant prostate cancer in clinical practice.

**Funding:**

Cancer Research UK, AstraZeneca, Prostate Cancer UK, the Prostate Cancer Foundation, the Experimental Cancer Medicine Centres Network, and the National Institute for Health Research Biomedical Research Centres.

**Research in context**

**Evidence before this study**

Trials for advanced prostate cancer have rarely pursued molecular stratification, and none of the drugs approved to date for metastatic prostate cancer care have a validated companion biomarker. Before starting this study, several genomic landscape studies were published describing an enrichment for aberrations in DNA repair genes in metastatic prostate cancers (studies identified in PubMed, searching for “prostate cancer”, “genomics”, and “biopsy”, between Jan 1, 2010 and Nov 1, 2015, with no language restrictions). Preclinical and clinical studies identified in PubMed (searching for “cancer”, “PARP”, and “BRCA” or “DNA repair” between Jan 1, 2005 and July 1, 2019, with no language restrictions) have established a correlation between different DNA repair defects and sensitivity to PARP inhibition in different tumour types, leading to drug approvals in ovarian and breast cancer. In the TOPARP-A trial, we identified an association between somatic alterations in DNA repair genes and antitumour activity of olaparib in 49 patients with metastatic prostate cancer. Other clinical trials of PARP inhibitors in prostate cancer were identified on the ClinicalTrials.gov website, searching for “prostate cancer” and “PARP” for studies published from database inception to July 1, 2019, without language restriction.

**Added value of this study**

To our knowledge, TOPARP-B is the first prospective clinical trial in a genomically defined population of patients with metastatic prostate cancer. TOPARP-B aimed to clinically qualify a predictive biomarker for treating metastatic prostate cancer. TOPARP-B also assessed different doses of olaparib, and correlated different genomic aberrations and antitumour activity. This study has confirmed the antitumour activity of olaparib against metastatic prostate cancer with defective DNA repair secondary to either germline or somatic gene inactivation.

**Implications of all the available evidence**

Randomised phase 3 trials for DNA repair-defective prostate cancers are now ongoing based on the TOPARP data. Our results, if confirmed in registration studies, would support the implementation of tumour genomic testing in clinical practice for treatment stratification in advanced prostate cancer.

## Introduction

Molecular stratification for treatment is not currently the standard of care for metastatic prostate cancers despite evidence of substantial interpatient genomic heterogeneity. Most therapeutic strategies for advanced prostate cancers target androgen receptor signalling; taxane-based chemotherapies and radiopharmaceuticals are also approved.^1^ Although these drugs have improved outcomes in the past decade, metastatic prostate cancer remains invariably fatal and new therapeutic strategies involving molecular stratification are urgently needed. Genomic studies of metastatic prostate cancer have identified a number of potentially actionable recurrent genomic aberrations,^2^, ^3^, ^4^ including loss-of-function alterations in DNA repair genes in 20–25% of cases, such as defects in homologous recombination-mediated repair genes.^3^ Among these, germline or somatic alterations in *BRCA2* are the most common, accounting for 6–12% of cases across studies.^2^, ^3^, ^4^ These data underpin the evaluation of poly(ADP-ribose) polymerase (PARP) inhibitors in metastatic prostate cancer.^5^, ^6^

Olaparib is an orally bioavailable inhibitor of the catalytic activity of PARP1 and PARP2, which have key roles in DNA damage response (DDR). Olaparib is approved for the treatment of advanced ovarian and breast cancers associated with germline *BRCA1* or *BRCA2* mutations.^7^ It is also approved as a maintenance therapy after response to platinum-based chemotherapy for ovarian cancer, indicating benefit from PARP inhibition beyond tumours with *BRCA1/2* mutations.^8^, ^9^ Furthermore, olaparib has antitumour activity in vitro and in vivo in models that are defective in other DDR proteins, including PALB2, ATM, FANCD2, RAD51, and RAD54, among others, although the magnitude of preclinical sensitisation varies between proteins, with *BRCA2* loss being arguably the most potent sensitising event.^10^, ^11^

To evaluate the antitumour activity of olaparib against metastatic castration-resistant prostate cancer, we designed TOPARP, an adaptive programme of serial phase 2 clinical trials aimed at identifying predictive biomarkers for response to PARP inhibition in metastatic castration-resistant prostate cancer. In the first trial, TOPARP-A, we identified an association between putatively deleterious DDR gene aberrations and response to olaparib in 49 molecularly unselected patients.^12^ In this Article, we present the results of TOPARP-B, which was designed to validate the observed antitumour activity of olaparib in patients with metastatic castration-resistant prostate cancer presenting with DDR gene aberrations.

## Methods

### Study design and participants

TOPARP-B is a multicentre, open-label, investigator-initiated, randomised phase 2 trial. Patients were recruited from 17 UK hospitals (appendix p 2).

Patients with prostate cancer that had developed metastasis and castration resistance were first registered on the trial for molecular preselection by targeted next-generation sequencing (NGS) of primary or metastatic prostate cancer biopsies. Eligible patients were men aged 18 years or older, with histologically confirmed prostate adenocarcinoma (metastatic and castration-resistant), and whose tumours had a putatively pathogenic mutation or homozygous deletion in a DDR gene that could be associated with sensitivity to PARP inhibition as identified by NGS. Patients were required to have previously received at least one but no more than two taxane-based chemotherapy regimens, regardless of prior exposure to novel hormonal drugs. Other inclusion criteria included: documented prostate cancer progression at trial entry, defined by either rising prostate-specific antigen (PSA) serum concentration (according to the Prostate Cancer Working Group 2 [PCWG2] criteria^13^) or radiologically (according to modified Response Evaluation Criteria in Solid Tumors [RECIST] version 1.1^14^ or by bone scan as per PCWG2 criteria); a castrate testosterone concentration of less than 50 ng/dL; an Eastern Cooperative Oncology Group (ECOG) performance status of 2 or less; and adequate organ function (including haemoglobin ≥9 g/dL after a protocol amendment on March 15, 2018 [previously ≥10 g/dL], platelets ≥100 × 10^9^ per L, serum creatinine ≤1·5 times the institutional upper limit of normal, and albumin >25 g/L). Patients previously treated with PARP inhibitors, platinum, cyclophosphamide, or mitoxantrone were not eligible, nor were patients with known symptomatic brain metastasis or untreated spinal cord compressions. The baseline count for circulating tumour cells (CellSearch system; Menarini Silicon Biosystems, Castel Maggiore, Italy) had to be five cells per 7·5 mL blood or higher except in patients with radiologically measurable target lesions of 2 cm or more in diameter on the baseline CT scan and a PSA concentration of 2 ng/mL or higher on screening. The full eligibility criteria are in the appendix (pp 3–4). The complete study protocol is available in the appendix.

The study was approved by the London–Surrey Borders Research Ethics Committee (REC reference 11/LO/2019), and co-sponsored by The Royal Marsden NHS Foundation Trust and The Institute of Cancer Research (ICR), London, UK. The trial was done in accordance with the principles of good clinical practice and overseen by independent data monitoring and trial steering committees. A trial management group was responsible for the day-to-day running of the trial. The Clinical Trials and Statistics Unit at ICR (ICR-CTSU) had overall responsibility for trial coordination, monitoring, and analysis. Patients provided written informed consent before enrolment, both for the NGS prescreening and treatment stages.

### Randomisation and masking

Eligible patients were randomly allocated (1:1) to receive olaparib at 300 mg (approved dose for the tablet formulation in ovarian and breast cancer^15^) or 400 mg twice a day. Randomisation was done centrally by the ICR-CTSU via telephone. The allocation sequence was generated centrally by a computer-generated minimisation algorithm derived by the ICR-CTSU, with circulating tumour cell count at screening (≥5 cells per 7·5 mL blood *vs* <5 cells per 7·5 mL blood) as a balancing factor. ICR-CTSU staff involved in the randomisation were not involved in the clinical running of the trial or data collection. Neither participants nor clinicians were masked to dose allocation.

### Procedures

The targeted NGS of tumour samples was done at the Cancer Biomarkers Laboratory at ICR. DNA was extracted from formalin-fixed and paraffin-embedded (FFPE) tumour blocks with a DNA FFPE Tissue Kit (Qiagen, Hilden, Germany). Samples that passed quality control testing with an FFPE QC Kit (Illumina, San Diego, CA, USA) were used for library preparation with a customised panel (GeneRead DNAseq Mix-n-Match Panel V2; Qiagen) covering 113 genes; libraries were read with a MiSeq Sequencer (Illumina). Further details on the sample processing, quality control, bioinformatics pipelines, and panel design are available in the appendix (pp 5–7).^16^ Patients previously known to have germline aberrations were eligible only on confirmatory tumour testing by NGS.

All patients received oral olaparib (300 mg or 400 mg, tablet formulation) twice daily continuously in 4-week cycles until evidence of radiographic progression (based on RECIST 1.1 for soft tissue disease, or the appearance of ≥2 lesions on bone scan), unacceptable toxicity according to investigator review, or patient decision to discontinue. Discontinuation because of clinical progression was the decision of the treating physician; discontinuation based solely on rising PSA in the absence of radiographic or clinical progression was discouraged. Patients treated with 300 mg twice daily were offered the option of dose escalation to 400 mg twice daily on confirmation of radiographic progression, providing the escalation was considered to be clinically indicated by the treating physician and the patient had not previously required a dose reduction for management of toxicity.

Clinical assessments, including reviews of adverse events (according to the National Cancer Institute Common Terminology Criteria for Adverse Events [CTCAE] version 4.02) and ECOG performance status, physical examination, and routine blood tests (haematology and biochemistry), took place 2 weeks after the start of treatment, and then at the start of every new 4-week cycle. Radiological assessments (CT and bone scans) were done every 12 weeks. Local radiological response assessments were used for the primary endpoint definition; all RECIST 1.1 responses were confirmed by central review by radiologists at ICR (AC and NT). Circulating tumour cell counts were measured every cycle for the first 12 weeks, and thereafter every 12 weeks. Circulating tumour cell counts were centrally analysed at the Cancer Biomarkers Laboratory at ICR (by PF, BE, and GF) and results were not made available to the treating physician. PSA serum measurements were collected every cycle if available, and every 12 weeks at a minimum. Blood samples for correlative biomarker studies were taken every 4 weeks. Repeated tumour biopsies were optional, and pursued when feasible at baseline, after 1–4 weeks on therapy, and at the time of progression. Guidance on drug interruptions or dose reductions for CTCAE grade 3–4 haematological and non-haematological toxicities were implemented as outlined in the protocol. Up to 42 days of temporary interruption of treatment was allowed prior to mandating permanent discontinuation.

### Outcomes

The primary endpoint was confirmed response, defined as a composite of any of the following outcomes: radiological objective response (as assessed by RECIST 1.1 [modified with PCWG2 recommendations], a decrease in PSA of 50% or more (PSA50) from baseline, and conversion of circulating tumour cell count (from ≥5 cells per 7·5 mL blood at baseline to <5 cells per 7·5 mL blood^17^). To be judged a response confirmation in a second consecutive assessment at least 4 weeks later was required.

Secondary endpoints were: radiographic progression-free survival, defined as the time from randomisation to first evidence of radiographic progression (according to RECIST 1.1 or bone scan as per PCWG2 criteria) or death; time to radiographic progression, defined as the time from randomisation to first evidence of radiographic progression; progression-free survival, defined as the time from randomisation to radiographic progression, unequivocal clinical progression, or death; overall survival, defined as the time from randomisation to death from any cause; time to PSA progression, defined as a confirmed increase of 25% or more and an absolute increase of 2 ng/mL or more in PSA from the nadir (PCWG2); duration of PSA response, defined as the time from the first documented PSA decrease of 50% or greater to PSA progression; best percentage change in PSA from baseline while on treatment; percentage change in PSA from baseline at 12 weeks (or earlier if therapy was discontinued); proportion of patients with circulating tumour cell count conversion; and the safety and tolerability profile of olaparib.

A prespecified exploratory endpoint was response in patients in whom dose was escalated to 400 mg twice daily after progression on 300 mg twice daily. A pharmacokinetics sub-study was planned but because of patients declining recruitment it was closed prematurely with no analyses pursued.

### Statistical analysis

This trial followed a selection (or pick-the-winner) design.^18^ Each dose cohort was assessed independently for the primary endpoint. The sample size needed to show the minimum desired antitumour activity was determined on the basis of A'Hern's one-stage design, with a response of 30% or less for the null hypothesis, and a response of more than 50% for the alternative hypothesis (one-sided α level of 0·05 and a β level of 0·15). Following the A'Hern design, if at least 19 (43%) of 44 evaluable patients in a dose cohort responded, then the dose cohort would be considered successful. If the 400 mg twice daily dose cohort was deemed successful, the DDR biomarker identified in TOPARP-A, in which all patients received 400 mg twice daily, would be considered validated as being predictive of response. In the event of both dose cohorts being successful, the pick-the-winner selection strategy would include consideration of secondary endpoints. No formal interim analyses were planned.

For the primary endpoint, the evaluable population was defined as all randomly assigned patients who met all of the eligibility criteria and commenced trial treatment, unless they discontinued treatment prior to 12 weeks for reasons that were not related to the study drug or disease. Sensitivity analyses of the primary endpoint were done in the intention-to-treat (ITT) population (all randomly assigned patients) and per protocol population (all evaluable patients who received at least one cycle of olaparib and had no eligibility violations). A post-hoc sensitivity analysis in patients with a circulating tumour cell count of five or more cells per 7·5 mL blood at baseline was done for comparison with TOPARP-A results. All other efficacy analyses were done in the ITT population. Toxicity was analysed in all patients who received at least one dose of olaparib, and the worst grades of adverse events that occurred during treatment for each dose cohort are reported. Serious adverse events and deaths observed within 30 days of the last dose of study treatment were summarised by dose cohort, as well as the exposure to study drug and reasons for discontinuation, dose modification or interruption, and treatment delay.

Analysis of the primary endpoint was triggered when all patients had completed at least 6 months of treatment (in the absence of prior discontinuation). Evaluable patients who discontinued treatment prior to 12 weeks due to progression or toxicity and had no follow-up assessments for the primary endpoint were considered non-responders. Response is presented along with exact two-sided 95% CIs. Percentage changes from baseline in PSA concentration and the sum of target lesions (RECIST 1.1) are represented in waterfall plots. Time-to-event endpoints are summarised by Kaplan-Meier curves, and median times estimated with 95% CIs. For radiographic progression-free survival and progression-free survival, patients alive and without progression were censored at the last scheduled disease assessment during the study. For time to radiographic progression, patients who did not progress radiologically were censored at the last scheduled disease assessment during the study or date of death, whichever occurred first. Patients alive at the end of follow-up were censored for the analysis of overall survival. Landmark analyses were used to explore the association between circulating tumour cell conversion at 8 weeks and 12 weeks with radiographic progression-free survival and overall survival. Additionally, exploratory subgroup analyses according to different genes of interest were preplanned for the efficacy endpoints. Five non-mutually exclusive subgroups were predefined: patients with alterations in *BRCA1*/*2, ATM, CDK12, PALB2*, and any other gene related to DDR or associated with PARP inhibitor sensitivity. Patients who had more than one DDR gene aberration were included in the analysis of all relevant subgroups.

The trial was not powered for head-to-head direct comparisons of the two dose cohorts, and so tests to compare them were considered hypothesis-generating (ie, χ^2^ test to compare the proportion of patients with a response and log-rank test to compare Kaplan-Meier curves). Statistical analyses were done with Stata software (version 15), on a snapshot of the data taken on July 5, 2019. The statistical analysis plan is available in the appendix.

This trial is registered with ClinicalTrials.gov, NCT01682772 and on the European Clinical Trials database, EudraCT 2011–000601–49.

### Role of the funding source

The funders had no role in the study design, data collection, data analysis, data interpretation, or writing of the report. The corresponding author had full access to all data in the study and had final responsibility for the decision to submit for publication.

## Results

**Figure 1:**
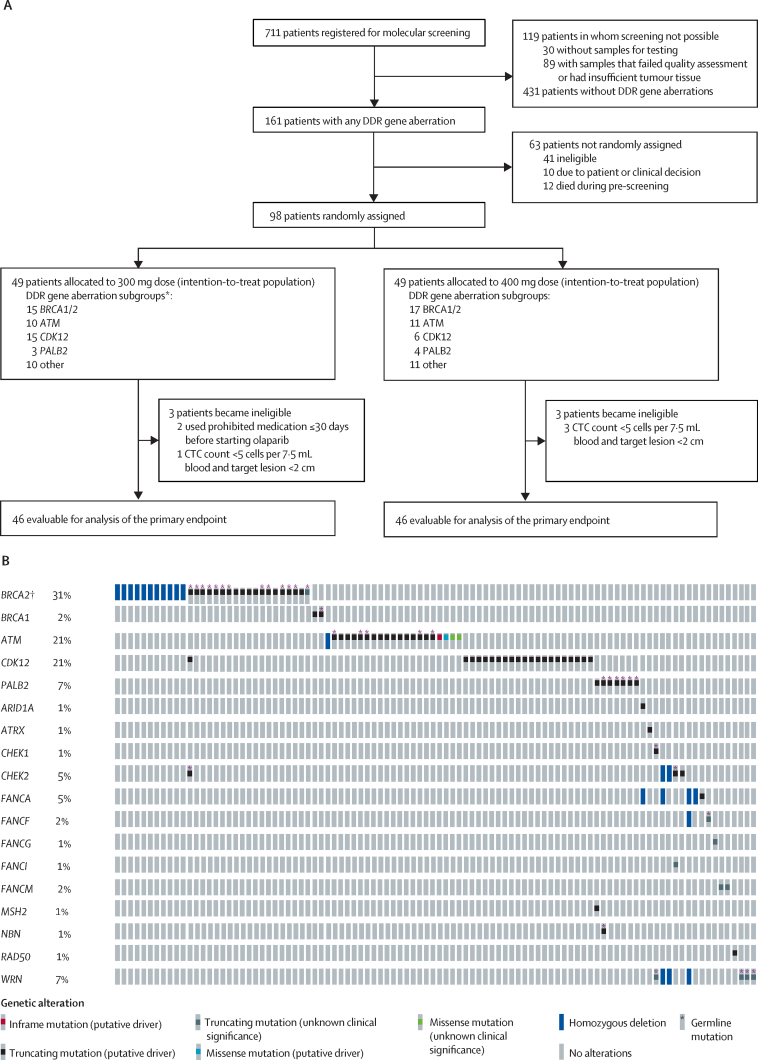
(A) Trial profile and (B) DDR gene alterations in the intention-to-treat population (n=98) DDR=DNA damage response. CTC=circulating tumour cell. *Non-mutually exclusive subgroups: one patient had *BRCA1/2, CDK12*, and other mutations, and two patients had both *PALB2* and other mutations (included in each subgroup). †The BRCA2 K3226* variant is supposedly non-pathogenic^19^ and was therefore not considered sufficient for patients to be considered eligible; however, one patient with a BRCA2 K3226* variant was included because of evidence of concomitant loss of the contralateral allele.

Between April 1, 2015 and Aug 30, 2018, 711 patients consented to NGS prescreening (figure 1A). For 30 (4%) patients, no samples were made available for testing. From 681 patients with at least one sample available, 779 tumour samples were analysed (637 [82%] primary tumour samples and 142 [18%] post-castration-resistance metastatic biopsies). For 89 (13%) patients, biomarker determination was not possible because of the sample or the sequencing data not fulfilling quality control parameters.

Of the 592 patients with evaluable tissue samples, 161 (27%) had DDR gene aberrations on the basis of NGS. An oncoprint summarising all alterations detected during prescreening is presented in the (p 14). The most commonly detected DDR gene aberrations were mutations or homozygous deletions in *BRCA2* (44 [7%] of the 592 patients), *ATM* (40 [7%]), and *CDK12* (33 [6%]).

98 patients with DDR gene aberrations were randomly assigned and treated in the two dose cohorts (49 patients in each cohort). At the time of the data snapshot, two patients remained on olaparib treatment. A greater number of participants were recruited than originally planned, at the recommendation of the independent data monitoring committee, to account for six participants (three in each cohort) who were deemed not evaluable (ineligible post-randomisation) for the primary endpoint analyses. Median follow-up was 24·8 months (IQR 16·7–35·9).

The baseline characteristics of all patients assigned to a dose cohort are shown in table 1. All patients had previously received docetaxel, and 88 (90%) had also been treated with one or both of abiraterone acetate and enzalutamide prior to study entry. The distribution of gene aberration subgroups was largely similar between the two dose cohorts, except for *CDK12* alterations (table 1). The composition of the prespecified gene aberration subgroups in the intention-to-treat population are shown in figure 1B. Baseline features of each gene aberration subgroup are summarised in the appendix (p 8).

**Table 1:** Baseline characteristics of patients in the intention-to-treat population

		**300 mg dose group (n=49)**	**400 mg dose group (n=49)**
Age at trial entry		67·3 (61·2–72·1)	67·6 (63·2–72·7)
Years from initial diagnosis		3·5 (2·4–6·4)	5·2 (3·6–7·3)
Years from diagnosis of castration-resistant prostate cancer		2·4 (1·2–3·7)	3·0 (1·8–4·0)
Metastatic disease at diagnosis			
	Yes	24 (49%)	25 (51%)
	No	24 (49%)	21 (43%)
	Not available	1 (2%)	3 (6%)
Gleason score at diagnosis			
	≤7	4 (8%)	15 (31%)
	≥8	42 (86%)	29 (59%)
	Not available	3 (6%)	5 (10%)
Previous treatment for prostate cancer			
	Prostatectomy	7 (14%)	6 (12%)
	Radical radiotherapy	22 (45%)	21 (43%)
	Bisphosphonates	2 (4%)	2 (4%)
	Radium-223	6 (12%)	8 (16%)
	Docetaxel	49 (100%)	49 (100%)
	Cabazitaxel	15 (31%)	22 (45%)
	Abiraterone acetate	24 (49%)	22 (45%)
	Enzalutamide	27 (55%)	29 (59%)
	Abiraterone acetate or enzalutamide or both	43 (88%)	45 (92%)
Evidence of progression at trial entry			
	PSA only	15 (31%)	12 (24%)
	Radiographic progression (with or without PSA progression)	34 (69%)	37 (76%)
Site of metastatic disease at trial entry*			
	Lung	4 (8%)	4 (8%)
	Lymph nodes	34 (69%)	32 (65%)
	Liver	11 (22%)	12 (24%)
	Bone	41 (84%)	41 (84%)
PSA at trial entry, ng/mL		151·5 (49·0–446·0)	158·0 (45·5–472·0)
CTC count per 7·5 mL blood at trial entry			
	<5	17 (35%)	17 (35%)
	≥5	31 (63%)	32 (65%)
	Not available†	1 (2%)	0
RECIST 1·1 soft tissue disease			
	Bone lesions only	5 (10%)	5 (10%)
	Non-measurable disease (with or without bone lesions)	5 (10%)	8 (16%)
	Measurable disease (with or without bone lesions)	39 (80%)	36 (73%)
DNA damage response gene aberration subgroup‡			
	*BRCA1/2*	15 (31%)	17 (35%)
	*ATM*	10 (20%)	11 (22%)
	*CDK12*	15 (31%)	6 (12%)
	*PALB2*	3 (6%)	4 (8%)
	Other	10 (20%)	11 (22%)

Data are median (IQR) or n (%). Percentages might not add up to 100% due to rounding. PSA=prostate-specific antigen. CTC=circulating tumour cell. RECIST=Response Evaluation Criteria in Solid Tumors.

* More than one site could be reported.

† Assessment of CTC count at screening not possible due to CTC kit shortage; the patient was allowed to be randomly assigned as he had RECIST 1·1 measurable disease; for randomisation CTC count was assumed to be <5 cells per 7·5 mL blood but the patient was unevaluable for CTC response.

‡ Non-mutually exclusive subgroups: one patient in the 300 mg cohort had *BRCA1/2, CDK12*, and other mutations, and two patients in the 300 mg cohort had *PALB2* and other mutations (in *MSH2* and *NBN*, respectively).

92 patients (46 in each dose cohort) were evaluable for the primary endpoint. 70 (76%) patients were evaluable for the RECIST 1.1 response, 89 (97%) for PSA response, and 55 (60%) for circulating tumour cell conversion. A confirmed composite response was observed in 25 (54·3%; 95% CI 39·0–69·1) of 46 patients in the 400 mg cohort and 18 (39·1%; 25·1–54·6) of 46 patients in the 300 mg cohort (p=0·14; table 2). Radiological response according to RECIST 1.1 was observed in eight (24·2%; 95% CI 11·1–42·3) of 33 evaluable patients in the 400 mg cohort and six (16·2%; 6·2–32·0) of 37 in the 300 mg cohort; PSA50 response was observed in 17 (37·0%; 23·2–52·5) of 46 and 13 (30·2%; 17·2–46·1) of 43, respectively; and circulating tumour cell count conversion was observed in 15 (53·6%; 33·9–72·5) of 28 and 13 (48·1%; 28·7–68·1) of 27, respectively. Based on the first 44 evaluable patients included in each cohort (as planned initially), 25 (57%) confirmed responses were recorded in the 400 mg cohort and 18 (41%) in the 300 mg cohort; thus, the predefined criteria for success was met for the 400 mg regimen but not for the 300 mg regimen.

**Table 2:** Overall antitumour activity of olaparib in patients with DNA damage response gene aberrations by dose cohort and gene subgroup

		**Composite overall response**	**RECIST 1.1 objective response**	**PSA50 response**	**CTC conversion**	**RECIST 1.1 or PSA50 response**
Total		43/92 (46·7%; 36·3–57·4)	14/70 (20·0%; 11·4–31·3)	30/89 (33·7%; 24·0–44·5)	28/55 (50·9%; 37·1–64·6)	32/92 (34·8%; 25·1–45·4)
By dose cohort						
	300 mg	18/46 (39·1%; 25·1–54·6)	6/37 (16·2%; 6·2–32·0)	13/43 (30·2%; 17·2–46·1)	13/27 (48·1%; 28·7–68·1)	13/46 (28·3%; 16·0–43·5)
	400 mg	25/46 (54·3%; 39·0–69·1)	8/33 (24·2%; 11·1–42·3)	17/46 (37·0%; 23·2–52·5)	15/28 (53·6%; 33·9–72·5)	19/46 (41·3%; 27·0–56·8)
By gene subgroup*						
	*BRCA1/2*	25/30 (83·3%; 65·3–94·4)	11/21 (52·4%; 29·8–74·3)	23/30 (76·7%; 57·7–90·1)	17/22 (77·3%; 54·6–92·2)	24/30 (80·0%; 61·4–92·3)
	*ATM*	7/19 (36·8%; 16·3–61·6)	1/12 (8·3%; 0·2–38·5)	1/19 (5·3%; 0·1–26·0)	5/10 (50·0%; 18·7–81·3)	2/19 (10·5%; 1·3–33·1)
	*CDK12*	5/20 (25·0%; 8·7–49·1)	0/18 (0·0%; 0–18·5†)	0/20 (0·0%; 0–16·8†)	5/12 (41·7%; 15·2–72·3)	0/20 (0·0%; 0–16·8†)
	*PALB2*	4/7 (57·1%; 18·4–90·1)	2/6 (33·3%; 4·3–77·7)	4/6 (66·7%; 22·3–95·7)	0/2 (0–84·2†)	4/7 (57·1%; 18·4–90·1)
	Other	4/20 (20·0%; 5·7–43·7)	0/17 (0·0%; 0–19·5†)	2/17 (11·8%; 1·5–36·4)	3/11 (27·3%; 6·0–61·0)	2/20 (10·0%; 1·2–31·7)

Data are n/N (%; 95% CI), where n=responding patients and N=evaluable patients. PSA50 response=PSA decrease ≥50%. RECIST=Response Evaluation Criteria in Solid Tumors. PSA=prostate-specific antigen. CTC=circulating tumour cell.

* Non-mutually exclusive subgroups: one patient treated at 300 mg had BRCA1/2, CDK12, and other mutations, and two patients treated at 300 mg had both PALB2 and other mutations. These patients have been included in analysis for each subgroup separately (for the gene subgroup analyses, dose cohorts have been pooled).

† One-sided exact binomial 95% confidence intervals.

When including in the analysis only the 55 evaluable patients with a circulating tumour cell count of ≥5 cells per 7·5 mL blood at baseline, confirmed composite response was observed in 17 (60·7%; 95% CI 40·6–78·5) of 28 evaluable patients in the 400 mg cohort and 13 (48·1%; 28·7–68·1) of 27 in the 300 mg cohort (appendix p 9). In keeping with previous reports,^17^, ^20^ circulating tumour cell conversions post-treatment were significantly associated with longer radiographic progression-free survival and overall survival in landmark analyses (appendix p 15).

**Figure 2:**
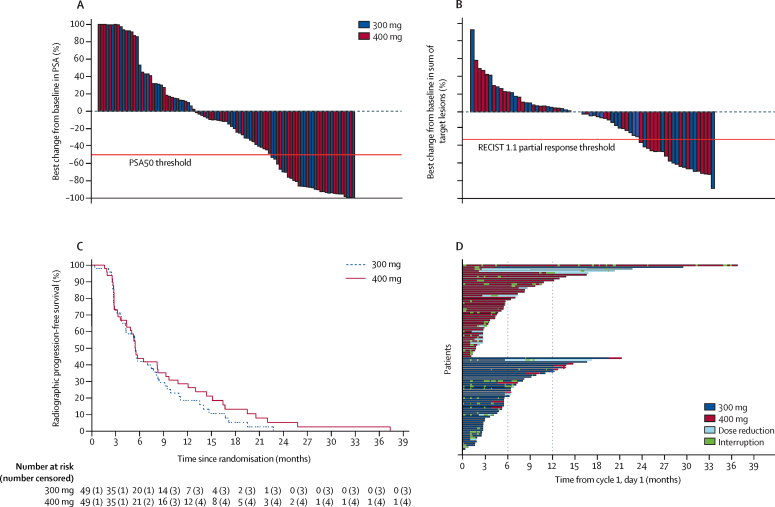
Antitumour activity by allocated dose cohort (intention-to-treat population) (A) Best percentage change from baseline in PSA during treatment. (B) Best percentage change from baseline in the sum of target lesions (Response Evaluation Criteria in Solid Tumors 1·1) during treatment. (C) Radiographic progression-free survival. (D) Swimmers plot of time on treatment for each patient, indicating periods of treatment interruptions, dose reductions, and, in the 300 mg cohort, dose escalations. Treatment periods of ≥6 months and ≥12 months are highlighted. PSA=prostate-specific antigen. PSA50=decrease in prostate-specific antigen of ≥50%.

The best percentage change from baseline in PSA concentration and in the sum of target lesions in each patient in the intention-to-treat population are presented in figure 2A and 2B. At the time of analysis, 45 (92%) of 49 patients on 400 mg olaparib and 46 (94%) of 49 patients on 300 mg olaparib had radiographic progression or died; median radiographic progression-free survival was 5·5 months (95% CI 4·4–8·3) in the 400 mg cohort and 5·6 months (3·7–7·7) in the 300 mg cohort (figure 2C). 39 (80%) patients on 400 mg and 38 (78%) patients on 300 mg had died, with a median overall survival of 14·3 months (9·7–18·9) in the 400 mg cohort and 10·1 months (9·0–17·7) in the 300 mg cohort. Further results on the secondary endpoints are summarised in the appendix (pp 16–18). The time on treatment for each patient is represented in figure 2D. A summary of treatment dose reductions, escalations (300 mg cohort), interruptions, and discontinuations in each dose cohort is presented in the appendix (p 10).

Dose escalation from 300 mg to 400 mg was pursued in 11 patients. At the time of the data snapshot, ten had discontinued treatment: two due to adverse events and eight due to disease progression. These 11 patients were on treatment with 400 mg olaparib for a median of 7·8 weeks (IQR 3·7–10·4). None of these patients achieved a response after dose escalation.

**Figure 3:**
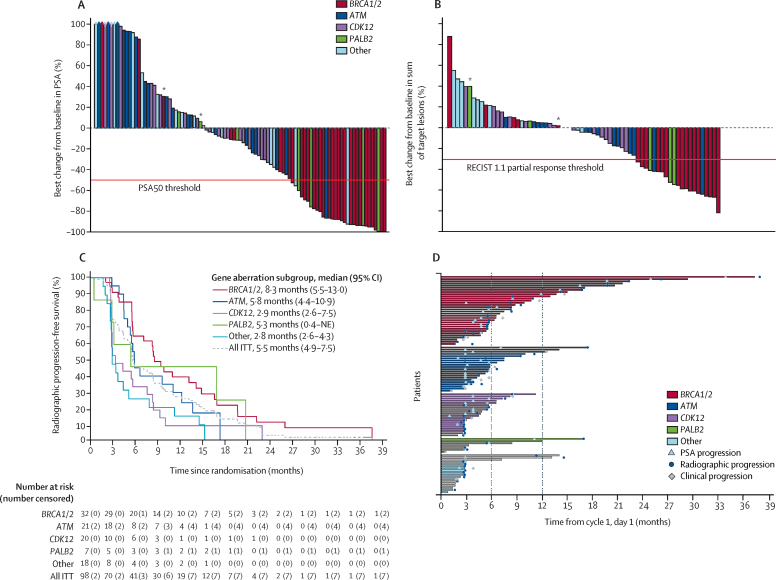
Antitumour activity by gene aberration subgroup (intention-to-treat population, pooled 300 mg and 400 mg cohorts) (A) Maximum percentage change from baseline in PSA during treatment. (B) Maximum percentage change from baseline in the sum of target lesions (Response Evaluation Criteria in Solid Tumors 1·1) during treatment. (C) Radiographic progression-free survival. (D) Swimmers plot of time on treatment for each patient. ITT=intention-to-treat. NE=not estimable. PSA=prostate-specific antigen. PSA50=decrease in prostate-specific antigen of ≥50%. *Patients presenting with mutliple mutations are represented in a single subgroup.

The confirmed composite response, and response by individual components, for each of the predefined gene subgroups are shown in table 2. Further analysis of secondary endpoints per gene subgroup are presented in figure 3 and the appendix (pp 11–14, 19). The *BRCA1/2* subgroup had the highest number of responses both for the composite endpoint of confirmed response and across all its component outcomes (table 2) and the longest median radiographic progression-free survival (figure 3C) of all DDR gene aberration subgroups. Of the 32 patients in the *BRCA1/2* subgroup, 13 had germline mutations in *BRCA2*, six somatic mutations in *BRCA2*, 11 homozygous deletions in *BRCA2*, and the remaining two cases had mutations in *BRCA1* (one germline and one somatic). Ten patients in the *BRCA1/2* subgroup (five allocated to 400 mg and five to 300 mg) remained on treatment for more than 1 year.

21 patients with suspected deleterious *ATM* aberrations were treated (table 1; one patient with homozygous deletion and the rest with germline or somatic mutations that are predicted to result in either truncation or missense mutations affecting the kinase domain), and 19 were evaluable for response (table 2). Details of each component of response in the evaluable patients with *ATM* aberrations are shown in the appendix (p 12).

No confirmed PSA50 or RECIST responses were observed in the 20 evaluable patients in the *CDK12* subgroup (table 2), although five patients achieved CTC conversion (including one with concomitant *BRCA1/2* alteration; appendix p 13). Conversely, four of seven patients with *PALB2* mutations responded to treatment (table 2).

20 patients were evaluated in the subgroup with other gene alterations associated with DDR or PARP inhibitor sensitivity (table 2). PSA50 responses were seen in two patients: one with a somatic nonsense mutation in *FANCA* and one with a *CHEK2* mutation.

The safety population included all 98 patients treated (table 3). The tolerability profile was in line with what has been previously reported for olaparib and other PARP inhibitors.^21^, ^22^, ^23^ The most common grade 3–4 adverse event in both cohorts was anaemia (15 [31%] in the 300 mg cohort and 18 [37%] in the 400 mg cohort).

**Table 3:** Treatment-emergent adverse events

	**300 mg (n=49)**			**400 mg (n=49)**		
	Grade 1–2	Grade 3	Grade 4	Grade 1–2	Grade 3	Grade 4
Anaemia	16 (33%)	14 (29%)	1 (2%)	19 (39%)	18 (37%)	0
Fatigue	19 (39%)	3 (6%)	0	27 (55%)	4 (8%)	0
Back pain	13 (27%)	4 (8%)	0	11 (22%)	3 (6%)	0
Nausea	17 (35%)	1 (2%)	0	13 (27%)	0	0
Platelet count decreased	9 (18%)	2 (4%)	1 (2%)	12 (24%)	3 (6%)	0
Decreased appetite	13 (27%)	2 (4%)	0	10 (20%)	0	0
Vomiting	10 (20%)	0	0	15 (31%)	0	0
Weight decreased	9 (18%)	1 (2%)	0	15 (31%)	0	0
Diarrhoea	8 (16%)	1 (2%)	0	10 (20%)	1 (2%)	0
Arthralgia	8 (16%)	1 (2%)	0	5 (10%)	4 (8%)	0
Hypertension	9 (18%)	1 (2%)	0	4 (8%)	4 (8%)	0
Neutrophil count decreased	9 (18%)	2 (4%)	0	4 (8%)	2 (4%)	1 (2%)
Dyspnoea	5 (10%)	1 (2%)	0	10 (20%)	1 (2%)	0
Abdominal pain	4 (8%)	0	0	6 (12%)	5 (10%)	1 (2%)
Blood creatinine increased	9 (18%)	0	0	6 (12%)	0	0
Oedema peripheral	6 (12%)	0	0	8 (16%)	1 (2%)	0
Urinary tract infection	3 (6%)	3 (6%)	0	6 (12%)	3 (6%)	0
Constipation	7 (14%)	0	0	7 (14%)	0	0
Cough	3 (6%)	0	0	9 (18%)	0	0
Musculoskeletal chest pain	3 (6%)	0	0	9 (18%)	0	0
Musculoskeletal pain	5 (10%)	1 (2%)	0	5 (10%)	1 (2%)	0
Hypokalaemia	3 (6%)	0	0	8 (16%)	0	0
Muscular weakness	4 (8%)	0	0	5 (10%)	2 (4%)	0
White blood cell count decreased	4 (8%)	0	0	6 (12%)	1 (2%)	0
Aspartate aminotransferase increased	3 (6%)	0	1 (2%)	4 (8%)	1 (2%)	0
Alkaline phosphatase increased	3 (6%)	0	0	5 (10%)	1 (2%)	0
Dysgeusia	6 (12%)	0	0	3 (6%)	0	0
Haematuria	5 (10%)	0	0	2 (4%)	2 (4%)	0
Influenza like illness	3 (6%)	0	0	6 (12%)	0	0
Muscle spasms	3 (6%)	0	0	6 (12%)	0	0
Gamma-glutamyl transferase increased	3 (6%)	0	0	2 (4%)	2 (4%)	1 (2%)
Lower respiratory tract infection	4 (8%)	1 (2%)	0	2 (4%)	1 (2%)	0
Lymphocyte count decreased	2 (4%)	1 (2%)	0	3 (6%)	2 (4%)	0
Pyrexia	4 (8%)	2 (4%)	0	2 (4%)	0	0
Alanine aminotransferase increased	2 (4%)	0	0	3 (6%)	2 (4%)	0
Groin pain	3 (6%)	0	0	2 (4%)	2 (4%)	0
Dizziness	2 (4%)	1 (2%)	0	2 (4%)	1 (2%)	0
Spinal cord compression	0	1 (2%)	0	0	5 (10%)	0
Blood bilirubin increased	1 (2%)	0	0	3 (6%)	0	1 (2%)
Cellulitis	2 (4%)	0	0	2 (4%)	1 (2%)	0
Pain	1 (2%)	1 (2%)	0	2 (4%)	1 (2%)	0
Hydronephrosis	1 (2%)	2 (4%)	0	0	1 (2%)	0
Hyponatraemia	0	1 (2%)	0	2 (4%)	1 (2%)	0
Myocardial infarction*	0	1 (2%)	1 (2%)	0	1 (2%)	0
Acute kidney injury	1 (2%)	0	1 (2%)	1 (2%)	0	0
Hyperkalaemia	0	1 (2%)	0	2 (4%)	0	0
Rectal haemorrhage	0	1 (2%)	0	1 (2%)	1 (2%)	0
Amylase increased	0	0	0	1 (2%)	1 (2%)	0
Atrial fibrillation	0	0	0	1 (2%)	1 (2%)	0
Circulatory collapse	0	2 (4%)	0	0	0	0
Confusional state	1 (2%)	1 (2%)	0	0	0	0
Femoral neck fracture	0	1 (2%)	0	1 (2%)	0	0
Femur fracture	0	0	0	0	2 (4%)	0
Mobility decreased	1 (2%)	0	0	0	1 (2%)	0
Pneumonia	0	0	0	0	2 (4%)	0
Presyncope	1 (2%)	0	0	0	1 (2%)	0
Pulmonary embolism	0	1 (2%)	0	0	1 (2%)	0
Respiratory tract infection	1 (2%)	1 (2%)	0	0	0	0
Abdominal infection	0	1 (2%)	0	0	0	0
Acute myeloid leukaemia	0	0	1 (2%)	0	0	0
Arthritis bacterial	0	1 (2%)	0	0	0	0
Bronchitis	0	1 (2%)	0	0	0	0
Cauda equina syndrome	0	0	0	0	1 (2%)	0
Embolism	0	0	0	0	1 (2%)	0
Enterocolitis infectious	0	0	0	0	1 (2%)	0
Febrile neutropenia	0	0	0	0	1 (2%)	0
Hip fracture	0	1 (2%)	0	0	0	0
Intestinal obstruction	0	0	0	0	1 (2%)	0
Jaundice	0	0	0	0	1 (2%)	0
Neutropenic sepsis	0	0	0	0	1 (2%)	0
Pyelonephritis	0	0	0	0	1 (2%)	0
Radiculopathy	0	0	0	0	1 (2%)	0
Renal colic	0	1 (2%)	0	0	0	0
Sepsis	0	0	0	0	0	1 (2%)
Ureteric obstruction	0	1 (2%)	0	0	0	0
Urosepsis	0	1 (2%)	0	0	0	0
Vascular pseudoaneurysm	0	0	1 (2%)	0	0	0
Vision blurred	0	1 (2%)	0	0	0	0

Adverse events were graded according to Common Terminology Criteria for Adverse Events version 4.02, and coded according to the the Medical Dictionary for Regulatory Activities version 22.0. Any grade 1–2 event occurring in 10% or more of patients is reported. All grade 3 and 4 events are reported.

* One death due to myocardial infarction (grade 5 event deemed a suspected unexpected serious adverse reaction) was reported (not included in table).

18 (37%) patients in the 400 mg cohort and six (12%) in the 300 mg cohort required at least one dose reduction (appendix p 10), with anaemia being the most common adverse event leading to dose reductions (two patients in the 300 mg cohort and nine in the 400 mg cohort). Eight patients who achieved a response on 400 mg continued to respond for more than 6 months after dose reduction to 300 mg or lower. Overall, 18 (19%) of the 98 patients were permanently discontinued from olaparib treatment due to adverse events (appendix p 10) The most common adverse events leading to discontinuation were anaemia (two of five patients who discontinued on 400 mg and five of 13 on 300 mg) and fatigue (three on 400 mg and four on 300 mg).

107 serious adverse events were reported in 49 (50%) patients (300 mg cohort: 49 events in 22 patients; 400 mg cohort: 58 events in 24 patients) with 19 serious adverse reactions (possibly related to study drug; 11 in the 300 mg cohort and eight in the 400 mg cohort) in 13 patients (8 patients in the 300 mg cohort and 5 in the 400 mg cohort). The most common serious adverse reaction was anaemia (occurring in six patients in the 300 mg cohort and five patients in the 400 mg cohort). Four serious adverse reactions were considered suspected unexpected, two in each dose cohort group. In the 400 mg cohort, one patient had community-acquired pneumonia, and the same patient had atrial fibrillation with myocardial infarction (recovering from both events). In the 300 mg cohort, one patient was diagnosed with myelodysplasia after 6·5 months of treatment, and developed acute myeloid leukaemia after olaparib discontinuation. One patient on 300 mg olaparib died due to a myocardial infarction (table 3), assessed as possibly drug-related, after 11 days of treatment. All other deaths were unrelated to treatment (n=76; 70 due to disease and six due to other causes).

## Discussion

The TOPARP-B trial has confirmed the antitumour activity of olaparib against metastatic castration-resistant prostate cancer with specific DDR gene aberrations. The number of composite responses observed in the cohort of patients who received 400 mg tablets of olaparib twice daily met the predefined criteria for success, validating the DDR biomarker identified in TOPARP-A as being predictive of response.^12^ Overall, the data suggest that both drug dose and the specific type of DDR gene aberration might influence antitumour activity, given that the composite response at the 300 mg regimen was lower and did not reach the predefined criteria for success. The antitumour activity observed varied considerably for different DDR gene aberrations, with the greatest antitumour activity seen in the subgroup with *BRCA1/2* alterations.

Despite randomisation, *CDK12* aberrations were imbalanced between the cohorts, with an enrichment in the 300 mg cohort. This imbalance might explain, at least in part, the inferior composite response in the 300 mg cohort.^4^, ^24^ The rationale to explore the two doses originated from prior clinical observations indicating a dose–response relationship for olaparib between 100 mg and 400 mg at twice daily dosing, although 400 mg has been associated with enhanced toxicity.^25^, ^26^ In keeping with this finding, 37% patients at 400 mg had to reduce their dose to 300 mg, most commonly because of anaemia. All of these data would need to be considered when assessing the optimal dose of olaparib for prostate cancer treatment.

Our results support the implementation of routine genomic testing of metastatic prostate cancer, to detect DNA repair defects for targeting by PARP inhibition. In a previous study, we reported an enrichment of germline inherited mutations in DDR genes in metastatic prostate cancer,^27^ which has led to the recommendation of broad germline NGS testing in all men with metastatic prostate cancer per National Comprehensive Cancer Network guidelines. The antitumour activity of olaparib indicated in this trial, in patients with metastatic castration-resistant prostate cancer with both germline and somatic aberrations of *BRCA2*, now supports the implementation of NGS testing of tumour samples.

Antitumour activity was also observed in other DDR gene aberration subgroups. Responses in tumours with *PALB2* mutations were frequent, although the low prevalence of these mutations means that further data are required to confirm these findings. Clinical qualification of low-prevalence biomarkers is challenging in the pursuit of precision medicine approaches; the validation of genomic signatures^24^, ^28^ or functional biomarkers^29^ that identify tumours with defective homologous-recombination, regardless of the mutated gene of origin, could help move the field forward, but such assays have not yet been validated in prostate cancer.

Conversely, germline and somatic *ATM* aberrations are common in metastatic prostate cancer; *ATM* functions as a cell cycle checkpoint, preventing cell cycle progression in the presence of DNA damage rather than directly mediating repair, unlike *BRCA2* and *PALB2*. In the TOPARP-A trial, five patients had *ATM* aberrations in tumour biopsies: two of these had a PSA response, and two more had circulating tumour cell conversion. Preliminary results suggest that rucaparib, another PARP inhibitor, results in few PSA decreases in patients with *ATM* aberrations.^30^ In TOPARP-B, we treated 21 patients with suspected deleterious *ATM* aberrations: two achieved a RECIST or PSA response, and several others had circulating tumour cell count conversions following therapy. Circulating tumour cell count decreases seen in this subgroup were associated with increased duration on the trial, tumour shrinkage per RECIST, and a PSA decrease, as was the case for the overall TOPARP-B population, with circulating tumour cell conversions robustly associating with increased radiographic progression-free survival and overall survival. Overall, the data suggest that the antitumour activity of olaparib in metastatic castration-resistant prostate cancer with *ATM* loss is less than that for *BRCA*-altered tumours; nevertheless, a subset of patients with *ATM*-altered metastatic castration-resistant prostate cancer appear to derive benefit. However, detection of *ATM* alterations alone might be insufficient to identify these sensitive tumours. Further studies, as well as the study of rational drug combinations, are now needed to elucidate how to best evaluate and treat metastatic castration-resistant prostate cancer with *ATM* alterations. Ongoing exploratory analyses from this trial will look to further characterise exceptional responses within each gene-defined subgroup to optimise patient stratification.

We do acknowledge limitations to this study. Although the use of targeted NGS facilitates the clinical implementation of patient stratification, this method might be insufficient to capture complex aberrations resulting in PARP inhibitor sensitivity. Furthermore, because all patients in our study had DDR gene aberrations and received olaparib, we are not able to fully differentiate the predictive value versus the prognostic effect of the gene aberrations in terms of survival. Randomised trials including patients with and without the biomarkers will be more able to clinically qualify putative predictive biomarkers.

Nonetheless, the results from TOPARP-B have overall driven the design and conduct of several registration trials of PARP inhibitors in metastatic castration-resistant prostate cancer (NCT02987543, NCT02975934, and NCT03148795), which are likely to guide the clinical use of PARP inhibitors in metastatic prostate cancer in the future. Most of these studies aim to validate PARP inhibition as a precision medicine strategy for prostate cancers with DDR gene aberrations. Other studies, in parallel, are exploring the addition of PARP inhibitors to the standard-of-care drugs targeting the androgen receptor (NCT03732820 and NCT03395197), on the basis of results from a phase 2 clinical trial indicating that a broader target population than just patients with gene aberrations might benefit from these drugs.^31^

In conclusion, the data from TOPARP-B have confirmed the antitumour activity of olaparib against metastatic prostate cancer with particular DDR gene aberrations. The high response observed in patients with metastatic castration-resistant prostate cancer with germline or somatic *BRCA1/2* aberrations, and the durability of many of these responses, support the use of olaparib in this subpopulation. The antitumour activity observed against tumours with *ATM, PALB2, FANCA*, or *CHEK2* aberrations suggest that PARP inhibitors might have a role as single drug therapies or in rational combinations against these other subtypes of metastatic prostate cancer, although further data are needed to precisely assess the clinical relevance of each of these different DDR gene aberrations in prostate cancer.

## Data sharing

The Institute of Cancer Research Clinical Trials and Statistics Unit (ICR-CTSU), London, UK, supports the wider dissemination of information from the research it does, and increased cooperation between investigators. Trial data is collected, managed, stored, shared, and archived according to ICR-CTSU standard operating procedures to ensure the enduring quality, integrity, and use of the data. Formal requests for data sharing are considered in line with ICR-CTSU procedures with due regard given to funder and sponsor guidelines. Requests are via a standard pro forma describing the nature of the proposed research and extent of data requirements. Data recipients are required to enter a formal data sharing agreement that describes the conditions for release and requirements for data transfer, storage, archiving, publication, and intellectual property. Requests are reviewed by the TOPARP Trial Management Group in terms of scientific merit and ethical considerations including patient consent. Data sharing is permitted if proposed projects have a sound scientific or patient benefit rationale as agreed by the Trial Management Group and approved by the ICR-CTSU independent data monitoring and steering committee as required. Restrictions relating to patient confidentiality and consent will be limited by aggregating and anonymising identifiable patient data. Additionally, all indirect identifiers that might lead to deductive disclosures will be removed in line with Cancer Research UK Data Sharing Guidelines.
