# Supplementary material for: Olaparib in patients with metastatic castration-resistant prostate cancer with DNA repair gene aberrations (TOPARP-B): a multicentre, open-label, randomised, phase 2 trial
Source: Lancet Oncol. 2020 Jan;21(1):162–74. doi: 10.1016/S1470-2045(19)30684-9 (PMC6941219; doi:10.1016/S1470-2045(19)30684-9)
Supplement: Supplementary appendix [file mmc1.pdf]

THE LANCET
Oncology

Supplementary appendix

This appendix formed part of the original submission and has been peer reviewed. We post it as supplied by the authors.

Supplement to: Mateo J, Porta N, Bianchini D, et al. Olaparib in patients with metastatic castration-resistant prostate cancer with DNA repair gene aberrations (TOPARP-B): a multicentre, open-label, randomised, phase 2 trial. *Lancet Oncol* 2019; published online Dec 2. http://dx.doi.org/10.1016/S1470-2045(19)30684-9.

TOPARP-B: A Randomised Phase 2 Trial Evaluating Two Doses of Olaparib Against Metastatic Castration-Resistant Prostate Cancer With DNA Repair Gene Aberrations

Supplementary Material

Table of contents

1. List of sites and investigators 2
2. Inclusion and exclusion criteria 3
3. Extended methods and NGS analysis 5
4. Supplementary Tables 8
   Table S1: Baseline characteristics on the ITT population, by gene subgroup (not-mutually exclusive groups*, pooled 300mg and 400mg BID cohorts) 8
   Table S2: Antitumor activity in DDRga patients with baseline CTC ≥5 cells/7.5 ml, by dose group (evaluable patients) 9
   Table S3: Dose reductions, interruptions and discontinuations, by dose cohort 10
   Table S4: Antitumor activity by DDRga subgroup and dose cohort (evaluable population, confirmed responses). 11
   Table S5: Line listing of response outcome for evaluable patients with ATM aberrations 12
   Table S6: Line listing of response outcome for evaluable patients with CDK12 aberrations 13
5. Supplementary Figures 14
   Figure S1: Oncoprint of all the 161 patients identified to have a tumor with a DDR gene aberration 14
   Figure S2 - Landmark analyses for Radiographic Progression Free Survival and Overall Survival, at landmark time-points 8 and 12-weeks 15
   Figure S3: Secondary endpoints results: (A) Progression Free Survival (B) Overall Survival 16
   Figure S4: Secondary endpoints results: (A) Time to PSA progression (B) Time to Radiographic Progression 17
   Figure S5: Secondary endpoints results: (A) Percentage change from baseline in PSA at 12 weeks (or earlier if allocated treatment discontinued) (B) Duration of PSA response 18
   Figure S6: Survival data by gene subgroup: (A) Progression Free Survival; (B) Overall Survival 19

1. List of sites and investigators

Site	Principal Investigator Name
The Royal Marsden NHS Foundation Trust, Sutton	Professor Johann de Bono
University College Hospital, London	Dr Ursula McGovern
The Christie Hospital, Manchester	Dr Tony Elliott
The Beatson West of Scotland Cancer Centre, Glasgow	Professor Robert Jones
The Clatterbridge Cancer Centre, Liverpool	Dr Isabel Syndikus
St James’s University Hospital, Leeds	Dr Christy Ralph
Churchill Hospital, Oxford	Dr Andrew Protheroe
Belfast City Hospital, Belfast	Dr Suneil Jain
Royal Sussex County Hospital, Brighton	Dr Angus Robinson
Royal Lancaster Infirmary, Lancaster	Dr Alison Birtle
Musgrove Park Hospital, Taunton	Dr Mohini Varughese
Royal Blackburn Hospital, Blackburn	Dr Omi Parikh
Southampton General Hospital, Southampton	Dr Simon Crabb
Airedale General Hospital, Keighley	Dr Simon Brown
Velindre Cancer Centre, Cardiff	Dr Jacob Tanguay
Western General Hospital, Edinburgh	Dr Duncan McLaren
Royal Free Hospital, London	Dr Maria Vilarino-Varela

2. Inclusion and exclusion criteria

INCLUSION CRITERIA

1. The subject is capable of understanding and complying with the protocol requirements and has signed the informed consent document.
2. Age ≥ 18 years.
3. Histologically confirmed adenocarcinoma of the prostate with archival tumor tissue available for molecular analyses. If the patient does not have a prior histological diagnosis then the planned baseline fresh biopsy may be used for both the purpose of confirming the histological diagnosis prior to trial entry and for subsequent biomarker analysis. All patients must be willing to have fresh biopsies to obtain tumor tissue for biomarker analysis. (Fresh biopsies are optional in those patients for whom sufficient archival tumor tissue is available for biomarker studies).
4. At least one but no more than two previous taxane-based chemotherapy regimens. If docetaxel chemotherapy is used more than once, this will be considered as one regime. Patients may have had prior exposure to cabazitaxel treatment. At least 28-days since the completion of prior therapy, including major surgery, chemotherapy and other investigational agents. Additionally, clinically relevant sequelae should have resolved to grade 1 or less prior to recommencing treatment. For hormonal treatment and radiotherapy refer to the guidelines below:
    - At least 28-days since the completion of prior flutamide treatment. Patients whose PSA did not decline in response to antiandrogens given as a second line or later intervention will only require a 14-days washout prior to Cycle 1, Day 1.
    - At least 42-days since the completion of prior bicalutamide (Casodex) and nilutamide (Nilandron) treatment. Patients whose PSA did not decline for 3 or 4 months in response to antiandrogens given as second line or later intervention will require only a 14-day washout period prior to Cycle 1 Day1.
    - At least 14 days from any radiotherapy with the exception of a single fraction of radiotherapy for the purposes of palliation (confined to one field) is permitted.
5. Documented prostate cancer progression as assessed by the investigator with one of the following:
    - PSA progression defined by a minimum of three rising PSA levels with an interval of ≥ 1 week between each determination. The PSA value at the Screening visit should be ≥ 2 µg/L (2 ng/ml); patients on systemic glucocorticoids for control of symptoms must have documented PSA progression by PCWG2 while on systemic glucocorticoids prior to commencing Cycle1 Day1 of treatment.
    - Radiographic progression of soft tissue disease by modified RECIST1.1 (appendix E) criteria or of bone metastasis with two or more documented new bone lesions on a bone scan with or without PSA progression.
6. Surgically or medically castrated, with testosterone levels of < 50 ng/dL (< 2.0 nM). If the patient is being treated with LHRH agonists/antagonists (patient who have not undergone orchiectomy), this therapy must have been initiated at least 4 weeks prior to Cycle 1 Day 1 and must be continued throughout the study.
7. Eastern Cooperative Oncology Group (ECOG) Performance Status of ≤ 2 (Karnofsky Performance Status ≥ 50%).
8. Life expectancy > 12 weeks.
9. Patient must be able to swallow a whole tablet.
10. Patient, and the patient's partner of childbearing potential, must agree to use medically accepted methods of contraception (e.g., barrier methods, including male condom, female condom, or diaphragm with spermicidal gel) during the course of the study and for 3 months after the last dose of study drug. Agreeable to have all the biomarker studies including the paired fresh tumor biopsies.

    Note: Paired fresh tumor biopsies are optional however a fresh tumor biopsy will be required during pre-screening if no archival tumor tissue is available for molecular analyses.
11. Subjects must have a CTC count of ≥5 cells/7.5mls blood at screening confirmed by the central laboratory.

    Note: For TOPARP-B the minimum CTC count at screening is not mandatory if the patient has measurable disease according to modified RECIST1.1 (protocol Appendix E) and a lesion >2 cms in size at screening and screening PSA ≥ 2 µg/L (2 ng/ml).
12. Subjects must have adequate bone marrow, hepatic and renal function documented within 7 days of randomisation (TOPARP – B), defined as:
    - Haemoglobin ≥9 g/dL. Patients who have anemia due to bone disease will be permitted to have a blood transfusion prior to trial entry to ensure they meet the eligibility criteria.
    - Absolute neutrophil count ≥1.5×10^9/L

- Platelets ≥100 x10^9/L
- Total bilirubin ≤1.5 x upper limit of normal (ULN) except for patients with known Gilbert's syndrome.
- Aspartate transaminase (AST) (SGOT) and alanine transaminase (ALT) (SGPT) ≤2.5 x ULN or ≤5 x ULN in the presence of liver metastases.
- Serum creatinine ≤1.5 x ULN or a calculated creatinine clearance >40mL/min for patients with creatinine levels above institutional normal. For GFR estimation, the Cockcroft and Gault equation should be used:

  GFR = CrCl (ml/min) = (140 - age) × wt (kg)/(serum creatinine × 72)
- Albumin >25 g/l

13. For TOPARP-B - Subjects must have genomic defects associated with olaparib sensitivity as identified by next generation sequencing (anticipated to be approximately 30% of patients) confirmed by the central laboratory or, if there is a next generation sequencing result report from an external laboratory, the patient could be enrolled prior to confirmation of these results by the central laboratory **following approval by the Chief Investigator** (in such cases the tumor sample will still be tested at the central laboratory).

EXCLUSION CRITERIA

1. Surgery, or local prostatic intervention (excluding a prostatic biopsy) less than 28 days of Cycle 1 Day 1.
2. Less than 28-days from any active anticancer therapy or investigational agents. For hormonal treatment and radiotherapy refer to the guidelines outlined in the inclusion criteria.
3. Prior treatment with a PARP inhibitor, platinum, cyclophosphamide or mitoxantrone chemotherapy.

   Note: If patients have received a treatment with the listed compounds for non-prostate cancer, the trials unit (ICR-CTSU) should be contacted to facilitate discussions with the CI as to the patients' suitability.
4. Uncontrolled intercurrent illness including, but not limited to, active infection, symptomatic congestive heart failure (New York Heart Association Class III or IV heart disease), unstable angina pectoris, cardiac arrhythmia, uncontrolled hypertension or psychiatric illness/social situations that would limit compliance with study requirements.
5. Any acute toxicities due to prior chemotherapy and / or radiotherapy that have not resolved to a NCI-CTCAE v4.02 grade ≤1 with the exception of chemotherapy-induced alopecia and grade 2 peripheral neuropathy.
6. Malignancy within the previous 2-years with a > 30% probability of recurrence within 12 months with the exception of non-melanoma skin cancer, in-situ or superficial bladder cancer.
7. Patients with myelodysplastic syndrome/acute myeloid leukaemia.
8. Patients with known symptomatic brain metastasis are not suitable for enrolment. Patients with asymptomatic, stable, treated brain metastases are eligible for study entry.
9. Patients with symptomatic or impending cord compression unless appropriately treated beforehand and clinically stable and asymptomatic.
10. Patients who have experienced a seizure or seizures within 6 months of study treatment or who are currently being treated with cytochrome P450 enzyme inducing anti-epileptic drugs for seizures (use of anti-epileptic drugs to control pain is allowed in patients not suffering from seizures unless drug is excluded due to CYP3A4 induction - phenytoin, carbamazepine, phenobarbital.
11. Patients receiving any of the following classes of inhibitors of CYP3A4:
    - Azole antifungals
    - Macrolide antibiotics
    - Protease inhibitors
12. Patients with gastrointestinal disorders likely to interfere with absorption of the study medication.
13. Initiating bisphosphonate therapy or adjusting bisphosphonate dose/regimen within 30 days prior to Cycle 1 Day 1. Patients on a stable bisphosphonate regimen are eligible and may continue.
14. Presence of a condition or situation, which, in the investigator's opinion, may put the patient at significant risk, may confound the study results, or may interfere significantly with patient's participation in the study.
15. The subject is unable or unwilling to abide by the study protocol or cooperate fully with the investigator or designee.

3. Extended methods and NGS analysis

Targeted next generation customized sequencing gene panel.

Gene ID	Symbol	# Amplicons	Target Bases	Target bases covered	% Covered
207	AKT1	30	1895	1670	88%
208	AKT2	30	1706	1554	91%
238	ALK	82	5443	5050	93%
324	APC	116	9017	8950	99%
367	AR	35	3115	2490	80%
8289	ARID1A	81	7258	6132	84%
196528	ARID2	82	5928	5830	98%
472	ATM	174	10411	10282	99%
545	ATR	140	8875	8738	98%
546	ATRX	131	8249	8233	100%
8312	AXIN1	38	2997	2486	83%
8313	AXIN2	39	2732	2528	93%
580	BARD1	35	2554	2376	93%
641	BLM	64	4674	4603	98%
673	BRAF	40	2860	2456	86%
672	BRCA1	98	6118	6118	100%
675	BRCA2	152	10777	10777	100%
701	BUB1B	60	3613	3553	98%
999	CDH1	43	2969	2891	97%
51755	CDK12	67	4806	4756	99%
1019	CDK4	19	1052	1052	100%
1027	CDKN1B	9	637	626	98%
1029	CDKN2A	14	1184	713	60%
1111	CHEK1	31	1671	1671	100%
11200	CHEK2	30	2061	1892	92%
1499	CTNNB1	40	2626	2626	100%
1643	DDB2	24	1484	1484	100%
1956	EGFR	72	4564	4348	95%
4072	EPCAM	17	1125	1002	89%
2064	ERBB2	75	4356	4236	97%
2065	ERBB3	78	4720	4651	99%
2066	ERBB4	75	4552	4536	100%
2068	ERCC2	46	2816	2554	91%
2071	ERCC3	44	2649	2601	98%
2072	ERCC4	45	2971	2971	100%
2073	ERCC5	57	3861	3792	98%
2074	ERCC6	97	6684	6684	100%
2146	EZH2	46	2680	2680	100%
54855	FAM46C	14	1196	1196	100%
2175	FANCA	97	5485	5285	96%
2187	FANCB	42	2740	2740	100%
2176	FANCC	32	2152	1944	90%
2177	FANCD2	83	5351	5038	94%
2178	FANCE	27	1811	1550	86%
2188	FANCF	14	1145	1120	98%
2189	FANCG	35	2149	2144	100%
55215	FANCI	81	4811	4811	100%
55120	FANCL	29	1483	1483	100%
57697	FANCM	96	6607	6551	99%
2263	FGFR2	50	3110	3053	98%
2261	FGFR3	42	2932	2444	83%
6927	HNF1A	35	2423	2050	85%
3265	HRAS	11	780	667	86%
3716	JAK1	65	3945	3945	100%
3717	JAK2	62	3859	3854	100%
3845	KRAS	13	787	787	100%
5604	MAP2K1	26	1436	1436	100%
5605	MAP2K2	24	1423	1240	87%
6416	MAP2K4	22	1502	1263	84%

Gene ID	Symbol	# Amplicons	Target Bases	Target bases covered	% Covered
4214	MAP3K1	65	5030	4419	88%
8491	MAP4K3	71	3365	3209	95%
4193	MDM2	29	1714	1680	98%
4233	MET	69	4704	4704	100%
4292	MLH1	46	2651	2651	100%
27030	MLH3	63	4602	4602	100%
4361	MRE11A	44	2556	2556	100%
4436	MSH2	44	3277	2926	89%
4437	MSH3	63	3894	3593	92%
2956	MSH6	52	4283	3990	93%
2475	MTOR	152	9204	9204	100%
4595	MUTYH	38	1970	1962	100%
4609	MYC	19	1425	1403	98%
4615	MYD88	16	1054	1054	100%
4683	NBN	43	2585	2528	98%
4763	NF1	143	9900	9167	93%
4771	NF2	36	2164	2164	100%
4792	NFKBIA	15	1074	827	77%
4851	NOTCH1	99	8348	7005	84%
4853	NOTCH2	111	8149	7779	95%
4893	NRAS	10	650	650	100%
4914	NTRK1	45	2980	2616	88%
79728	PALB2	53	3821	3803	100%
5156	PDGFRA	60	3831	3778	99%
5290	PIK3CA	50	3607	3313	92%
5294	PIK3CG	44	3509	3296	94%
5295	PIK3R1	42	2637	2627	100%
5395	PMS2	30	2889	2126	74%
5591	PRKDC	233	14106	13829	98%
5728	PTEN	22	1392	1248	90%
10111	RAD50	65	4439	4320	97%
5890	RAD51B	30	1600	1600	100%
5889	RAD51C	24	1315	1315	100%
5892	RAD51D	22	1386	1335	96%
5925	RB1	54	3327	2965	89%
9401	RECQL4	46	4048	3191	79%
5979	RET	59	3777	3479	92%
6597	SMARCA4	88	5761	5040	87%
6598	SMARCB1	22	1392	1279	92%
8405	SPOP	22	1305	1305	100%
6714	SRC	30	1831	1555	85%
6794	STK11	23	2135	1258	59%
7128	TNFAIP3	36	2533	2533	100%
8764	TNFRSF14	19	1286	1053	82%
7157	TP53	26	1503	1396	93%
7248	TSC1	64	3915	3915	100%
7249	TSC2	103	6537	5937	91%
7428	VHL	12	702	492	70%
7486	WRN	85	4979	4976	100%
7490	WT1	24	1784	1282	72%
7507	XPA	12	942	750	80%
7508	XPC	51	3143	3015	96%
7517	XRCC3	17	1205	1047	87%
8233	ZRSR2	28	1669	1654	99%
	**Total**	**2338**	**155986**	**144654**	**91%**

Pre-screening sequencing processing and quality control

DNA extraction and quality Control Tests

A pathologist reviewed all tumor samples to be tested. DNA was extracted from 4-8 x10uM sections from formalin-fixed and paraffin embedded (FFPE) blocks positive for tumor content using the FFPE Tissue DNA kit (Qiagen). DNA was quantified with the Quant-iT high-sensitivity PicoGreen double-stranded DNA Assay Kit (Invitrogen). A minimum of 40ng of DNA was required. The Illumina FFPE QC kit (WG-321-1001) was used for DNA quality control tests according to the manufacturer's protocol. In brief, quantitative polymerase chain reaction (qPCR) was performed using 4ng of sample or control DNA and the average Cq (quantification cycle) was determined. The average Cq value for the control DNA was subtracted from the average Cq value of the samples to obtain a ΔCq. DNA samples with a ΔCq<4 were selected for sequencing; double the amount of DNA was used for cases with ΔCq between 2-4.

Copy-number calling from targeted sequencing data

Copy number variations (CNV) in the prostate biopsies were assessed from the aligned sequence files (.bam) using CNVkit (v0.3.5, https://github.com/etal/cnvkit). The read depths of tumor samples were normalized and individually compared to a reference consisting of non-matched male germline DNA; the circular binary segmentation (CBS) algorithm was used to infer copy number segments. Quality estimation of the CNV was based on distribution of bin-level copy ratios within segments. Cases were excluded from the analysis if any of the following criteria were met: IQR>1, total reads<500000, <99.9% of reads on target, <95% paired reads or single reads>0. Manual review of copy number calls for selected oncogenes and tumor suppressors was pursued, accounting for tumor content. Copy-number estimation was pursued for 96/113 genes in the panel based on prior published validation studies; 17 sequenced genes were excluded from the copy number analyses based on poor accuracy of can for these genes based on CNVkit assessment as we have previously described (Seed et al, Clin Can Res 2017).

4. Supplementary Tables

Table S1: Baseline characteristics on the ITT population, by gene subgroup (not-mutually exclusive groups*, pooled 300mg and 400mg BID cohorts)

	**BRCA1/2 (N=32)**		**ATM (N=21)**		**CDK12 (N=21)**		**PALB2 (N=7)**		**Other (N=18)**	
**Age, mean (SD)**	65.7 (7.2)		68.7 (6.6)		68.1 (7.3)		65.4 (7.8)		69.4 (8.9)	
**Years from initial diagnosis** - median (Q1-Q3)	3.8 (2.1-6.0)		5.2 (3.3-8.2)		4.9 (3.3-6.7)		4.6 (1.4-6.5)		4.6 (2.8-7.6)	
**Years from diagnosis of CRPC** - median (Q1-Q3)	2.4 (1.6-3.8)		2.5 (1.4-4.6)		2.6 (1.7-3.7)		2.9 (0.3-4.0)		2.9 (2.0-3.8)	
**Metastatic disease at diagnosis**										
No	15	46.9	9	42.9	8	38.1	4	57.1	10	47.6
Yes	16	50	11	52.4	12	57.1	3	42.9	10	47.6
Not available	1	3.1	1	4.8	1	4.8	0	0	1	4.8
**Gleason score at diagnosis**										
≤7	10	31.3	7	33.3	0	0	0	0	2	9.5
≥8	20	62.5	11	52.4	20	95.2	6	85.7	18	85.7
Not available	2	6.3	3	14.3	1	4.8	1	14.3	1	4.8
**Previous hormonal and systemic treatment**										
Docetaxel	32	100	21	100	21	100	7	100	21	100
Cabazitaxel	12	37.5	9	42.9	8	38.1	2	28.6	6	28.6
Abiraterone	15	46.9	8	38.1	9	42.9	5	71.4	11	52.4
Enzalutamide	17	53.1	12	57.1	14	66.7	3	42.9	13	61.9
Abiraterone and/or Enzalutamide	28	87.5	18	85.7	19	90.5	6	85.7	21	100
**Evidence of progression at trial entry**										
PSA only	6	18.8	8	38.1	7	33.3	3	42.9	3	14.3
Radiographic progression (+/- PSA progression)	26	81.3	13	61.9	14	66.7	4	57.1	18	85.7
**Site of metastatic disease at trial entry(1)**										
Lung	6	18.8	0	0	2	9.5	0	0	2	9.5
Lymph nodes	22	68.8	15	71.4	12	57.1	6	85.7	14	66.7
Liver	9	28.1	0	0	6	28.6	2	28.6	7	33.3
Bone	27	84.4	17	81	19	90.5	4	57.1	15	71.4
**PSA at trial entry (ng/ml)** – median (Q1-Q3)	148.5 (38.9-473.0)		168.0 (52.0-399.2)		248.1 (28.5-506.8)		144.0 (14.0-196.0)		139.1 (41.0-540.0)	
**CTC count at trial entry**										
CTC<5	10	31.3	8	38.1	6	28.6	4	57.1	8	38.1
CTC >= 5	22	68.8	12	57.1	15	71.4	3	42.9	13	61.9
Not available(2)	0	0	1	4.8	0	0	0	0	0	0
**RECIST soft tissue disease at trial entry**										
Bone lesions only	4	12.5	3	14.3	1	4.8	0	0	2	9.5
Non-measurable disease only (+/- bone lesions)	5	15.6	4	19	2	9.5	1	14.3	1	4.8
Measurable disease (+/- bone lesions)	23	71.9	14	66.7	18	85.7	6	85.7	18	85.7

Q1: 25% percentile, Q3: 75% percentile

* Non-mutually exclusive subgroups - one patient had BRCA1/2, CDK12 and ‘Other mutations’, and two patients had PALB2 and other mutations included in analysis for each subgroup separately

(1) More than one site could be reported.

(2) Screening CTC assessment not possible due to CTC kit shortage. Patient allowed to be randomised as he had RECIST measurable disease; for randomisation CTC assumed <5 but patient was unevaluable for CTC response.

Table S2: Antitumor activity in DDRga patients with baseline CTC ≥5 cells/7.5 ml, by dose group (evaluable patients)

	Total (N=55)			Dose group					
				300mg (N=27)			400mg (N=28)		
	resp/n	%	95% CI	resp/n	%	95% CI	resp/n	%	95% CI
**Overall Response (confirmed)**	30/55	54.5%	40.6-68.0	13/27	48.1%	28.7-68.1	17/28	60.7%	40.6-78.5
RECIST Objective Response	6/36	16.7%	6.4-32.8	3/19	15.8%	3.4-39.6	3/17	17.6%	3.8-43.4
PSA fall ≥50	19/53	35.8%	23.1-50.2	8/25	32.0%	14.9-53.5	11/28	39.3%	21.5-59.4
CTC conversion	28/55	50.9%	37.1-64.6	13/27	48.1%	28.7-68.1	15/28	53.6%	33.9-72.5

Resp: responses; 95% CI: 95% confidence interval

Table S3: Dose reductions, interruptions and discontinuations, by dose cohort

		Total (N=98)		Dose cohort			
				300 mg (N=49)		400 mg (N=49)	
**Treatment discontinuation**		**n**	**%**	**n**	**%**	**n**	**%**
Yes		96	98.0	47	95.9	49	100.0
No[1]		2	2.0	2	4.1	0	0.0
**Reasons for treatment discontinuation[2]**		96	100	47	100	49	100
Radiographic Progression		58	60.4	24	51.1	34	69.4
Clinical Progression		15	15.6	8	17.0	7	14.3
AE		18	18.8	13	27.7	5	10.2
Death[3]		3	3.1	2	4.2	1	2.0
Other		2	2.1	0	0.0	2	4.1
**Reasons for ALLOCATED treatment discontinuation**		97	100	48	100	49	100
Radiographic Progression		54	55.7	20	41.7	34	69.4
Radiographic progression (dose-escalation)		11	11.3	11	22.9	0	0.0
Clinical Progression		11	11.3	4	8.3	7	14.3
AE		16	16.5	11	22.9	5	10.2
Death[3]		3	3.1	2	4.2	1	2.0
Other		2	2.1	0	0.0	2	4.1
**Number of cycles/patient**	Median (Q1-Q3)	6 (3-9)		6 (3-8)		6 (3-9)	
	Min-Max	1-40		1-23		1-40	
**Time on treatment (months)**	Median (Q1-Q3)	5.5 (2.7-8.3)		5.5 (2.7-8.1)		5.2 (2.7-8.7)	
	Min-Max	0.4-36.8		0.4-21.3		1.2-36.8	
**Patients dose-escalated after progression at 300mg**		11	11.2	11	22.4	0	0.0
**Number of dose-escalated cycles/patient**	Median (Q1-Q3)	2 (1-3)		2 (1-3)		NA	
	Min-Max	0*-3		0*-3		NA	
**Patients with dose reductions (AE-related)**							
No dose reduction		74	75.5	43	87.8	31	63.3
At least 1 dose reduction		24	24.5	6	12.2	18	36.7
**Patients with at least 1 cycle interrupted/delayed (clinical reasons)**		66	67.3	39	79.6	27	55.1
Patients with at least 1 cycle interrupted/delayed for ≥14 days		30	30.6	9	18.4	21	42.9
**Patients who forgot at least one dose in any given cycle[4]**		40	40.8	20	40.8	20	40.8

Q1: 25% percentile, Q3: 75% percentile

(1) One patient due to start 400mg after dose-escalation (2) Overall, including final reasons to stop treatment after dose-escalation (3) Deaths: 1 SAE (300 mg), 2 due to disease (1 300mg, 1 400mg) (4) No more than 2 full days of forgotten doses per cycle

*One patient to dose-escalate at the time of the snapshot.

Table S4: Antitumor activity by DDRga subgroup and dose cohort (evaluable population, confirmed responses).

		All patients (N=92)			300 mg (N=46)			400 mg (N=46)		
**DDRga subgroup**	**Endpoint**	**resp/n**	**%**	**95% CI**	**resp/n**	**%**	**95% CI**	**resp/n**	**%**	**95% CI**
**Group 1: BRCA1/2 (n=30)**	Overall Response	25/30	83.3%	65.3-94.4	11/14	78.6%	49.2-95.3	14/16	87.5%	61.7-98.4
	RECIST 1.1 Objective Response	11/21	52.4%	29.8-74.3	5/10	50.0%	18.7-81.3	6/11	54.4%	23.4-83.3
	PSA fall ≥50	23/30	76.7%	57.7-90.1	10/14	71.4%	41.9-91.6	13/16	81.3%	54.4-96.0
	CTC conversion	17/22	77.3%	54.6-92.2	9/11	81.8%	48.2-97.7	8/11	72.7%	39.0-94.0
	RECIST 1.1 or PSA response	24/30	80.0%	61.4-92.3	10/14	71.4%	41.9-91.6	14/16	87.5%	61.7-98.4
**Group 2: ATM (n=19)**	Overall Response	7/19	36.8%	16.3-61.6	2/9	22.2%	2.8-60.0	5/10	50.0%	18.7-81.3
	RECIST 1.1 Objective Response	1/12	8.3%	0.2-38.5	0/6	0.0%	0-45.9*	1/6	16.7%	0.4-64.1
	PSA fall ≥50	1/19	5.3%	0.1-26.0	1/9	11.1%	0.3-48.2	0/10	0.0%	0-30.8*
	CTC conversion	5/10	50.0%	18.7-81.3	1/4	25.0%	0.6-80.6	4/6	66.7%	22.3-95.7
	RECIST 1.1 or PSA response	2/19	10.5%	1.3-33.1	1/9	11.1%	0.3-48.2	1/10	10.0%	0.3-44.5
**Group 3: CDK12 (n=20)**	Overall Response	5/20	25.0%	8.7-49.1	4/14	28.6%	8.4-58.1	1/6	16.7%	0.4-64.1
	RECIST 1.1 Objective Response	0/18	0.0%	0-18.5*	0/13	0.0%	0-24.7*	0/5	0.0%	0-52.2*
	PSA fall ≥50	0/20	0.0%	0-16.8*	0/14	0.0%	0-23.2*	0/6	0.0%	0-45.9*
	CTC conversion	5/12	41.7%	15.2-72.3	4/9	44.4%	13.7-78.8	1/3	33.3%	0.8-90.6
	RECIST 1.1 or PSA response	0/20	0.0%	0-16.8*	0/14	0.0%	0-23.2*	0/6	0.0%	0-45.9*
**Group 4: PALB2 (n=7)**	Overall Response	4/7	57.1%	18.4-90.1	1/3	33.3%	0.8-90.6	3/4	75.0%	19.4-99.4
	RECIST 1.1 Objective Response	2/6	33.3%	4.3-77.7	1/3	33.3%	0.8-90.6	1/3	33.3%	0.8-90.6
	PSA fall ≥50	4/6	66.7%	22.3-95.7	1/2	50.0%	1.3-98.7	3/4	75.0%	19.4-99.4
	CTC conversion	0/2	0.0%	0-84.2*	0/0	0.0%	-	0/2	0.0%	0-84.2
	RECIST 1.1 or PSA response	4/7	57.1%	18.4-90.1	1/3	33.3%	0.8-90.6	3/4	75.0%	19.4-99.4
**Group 5: Other (n=20)**	Overall Response	4/20	20.0%	5.7-43.7	2/10	20.0%	2.5-55.6	2/10	20.0%	2.5-55.6
	RECIST 1.1 Objective Response	0/17	0.0%	0-19.5*	0/9	0.0%	0-33.6	0/8	0.0%	0-36.9
	PSA fall ≥50	2/17	11.8%	1.5-36.4	1/7	14.3%	0.4-57.9	1/10	10.0%	0.3-44.5
	CTC conversion	3/11	27.3%	6.0-61.0	1/5	20.0%	0.5-71.6	2/6	33.3%	4.3-77.7
	RECIST 1.1 or PSA response	2/20	10.0%	1.2-31.7	1/10	10.0%	0.3-44.5	1/10	10.0%	0.3-44.5

Non-mutually exclusive subgroups - one patient with BRCA1/2+CDK12+Other mutations and two patients with PALB2+Other mutations included in analysis for each subgroup separately. Resp: responses; 95% CI: 95% confidence interval; *One-sided exact binomial 95% confidence intervals

Table S5: Line listing of response outcome for evaluable patients with ATM aberrations

Patient ID	RECIST disease at baseline	Max % change target lesions	Best RECIST response	Months to first RECIST OR	Baseline CTC	Best % CTC change	CTC conversion (confirmed)	Months to CTC response	PSA baseline	Best % PSA change	PSA response (confirmed)	Months to PSA response	Months on allocated treatment	Reason EoT
1	Bone lesions only		NE		35	-100	Yes	0.9	11	-17.3	No		6.5	AE
2	Measurable disease	**-25**	SD		0	0			399.2	**-86.7**	Yes	2.8	**17.3**	Progression
3	Measurable disease	-6.8	SD		13	-84.6	Yes	1.2	2074	19	No		5.8	Progression
4	Non-measurable only		SD		99	**-100**	Yes	1.9	53	**-24.5**	No		**13.9**	Clinician decision
5	Measurable disease	**-42.2**	PR	2.4	3	**-100**			11.6	-44.8	No		**12.3**	Progression
6	Measurable disease	**-63.6**	Unconfirmed PR		18	**-100**	**Yes**	0.9	16	280	No		5.2	Death (disease)
7	Non-measurable only		SD		52	-100	Yes	1.9	460	-6.3	No		5.7	AE
8	Measurable disease	4.8	SD		0	0			187	93	No		5.7	Progression
9	Non-measurable only		SD		1145	-70	No		638	30.1	No		4.2	Progression
10	Measurable disease	**-17.8**	SD		537	**-94.8**	No		286	93	No		2.7	AE
11	Measurable disease	-5	SD		4	**-100**			904	-40.2	No		**9.2**	Progression
12	Measurable disease	10.4	SD		1	-100			32	87.5	No		4.4	Progression
13	Measurable disease	-4.3	SD		12	-25	No		331	94.3	No		3.7	Progression
14	Measurable disease	6.3	SD						450	12.4	No		4.6	Progression
15	Measurable disease	**-26.5**	SD		2	**-100**			168.5	12.7	No		**9.9**	AE
16	Bone lesions only		NE		4	-50			54.2	43	No		2.7	Progression
17	Bone lesions only		PD		96	-27.1	No		203.7	176.3	No		3.6	Progression
18	Non-measurable only		SD		279	-7.9	No		143	110.5	No		1.2	Progression
19	Measurable disease	4.4	SD		3	**-100**			221	-11.8	No		**7.4**	Progression

Table S6: Line listing of response outcome for evaluable patients with CDK12 aberrations

Patient ID	RECIST disease at baseline	Max % change target lesions	Best RECIST response	Months to first RECIST OR	Baseline CTC	Best % CTC change	CTC conversion (confirmed)	Months to CTC response	PSA baseline	Best % PSA change	PSA response (confirmed)	Months to PSA response	Months on allocated treatment	Reason EoT
49*	Measurable disease	1.9	PD		6	-100	Yes	0.9	252	31	No		3.2	Progression
50	Measurable disease	4.1	SD		42	-95.2	Yes	0.9	294	-10.2	No		8.3	Progression
51	Measurable disease	-15.1	SD		19	-100	Yes	0.3	450	-2	No		5.5	Progression
52	Measurable disease	-19.8	PD		6	-100	Yes	0.2	1829	-3.7	No		2.7	Progression
53	Measurable disease	0	SD		179	-100	Yes	0.9	725	-33.2	No		5.3	Progression
54	Measurable disease	16.7	PD		49	-65.3	No		190	10	No		2.6	Progression
55	Bone lesions only		PD		110	-50.9	No		595	41	No		2.7	Progression
56	Measurable disease	-2.3	SD		1	-100			150	-14.7	No		7.2	Progression
57	Measurable disease	40	PD		10	-80	No		25	28	No		2.7	Progression
58	Measurable disease	10.3	SD		4	-100			12	241.7	No		4.6	Progression
59	Measurable disease	2.3	PD		0	0			139.1	-20.4	No		2	AE
60	Measurable disease	21.1	PD		1	-100			270.6	97.8	No		3.1	Progression
61	Measurable disease	6.6	SD		4	-75			306.5	-11.8	No		3.4	Progression
62	Non-measurable only		SD		39	-94.9	No		506.8	-31	No		8.1	AE
63	Measurable disease	-22.7	SD		47	-70.2	No		72.3	-30.8	No		6.5	AE
64	Measurable disease	-11.8	PD		75	-69.3	No		2455	-7.7	No		2.7	Progression
65	Measurable disease	-6.2	PD		11	-81.8	No		142	43	No		3	Progression
66	Measurable disease	0	SD		0	0			47.4	14.3	No		6.4	Progression
67	Measurable disease	15.6	SD		2	-100			152.9	-9.9	No		4.4	Progression
68	Measurable disease	-9	SD		2	-100			107.5	-27.2	No		4.8	Progression

* Concurrent BRCA2 and CDK12 aberration

5. Supplementary Figures

Figure S1: Oncoprint of all the 161 patients identified to have a tumor with a DDR gene aberration

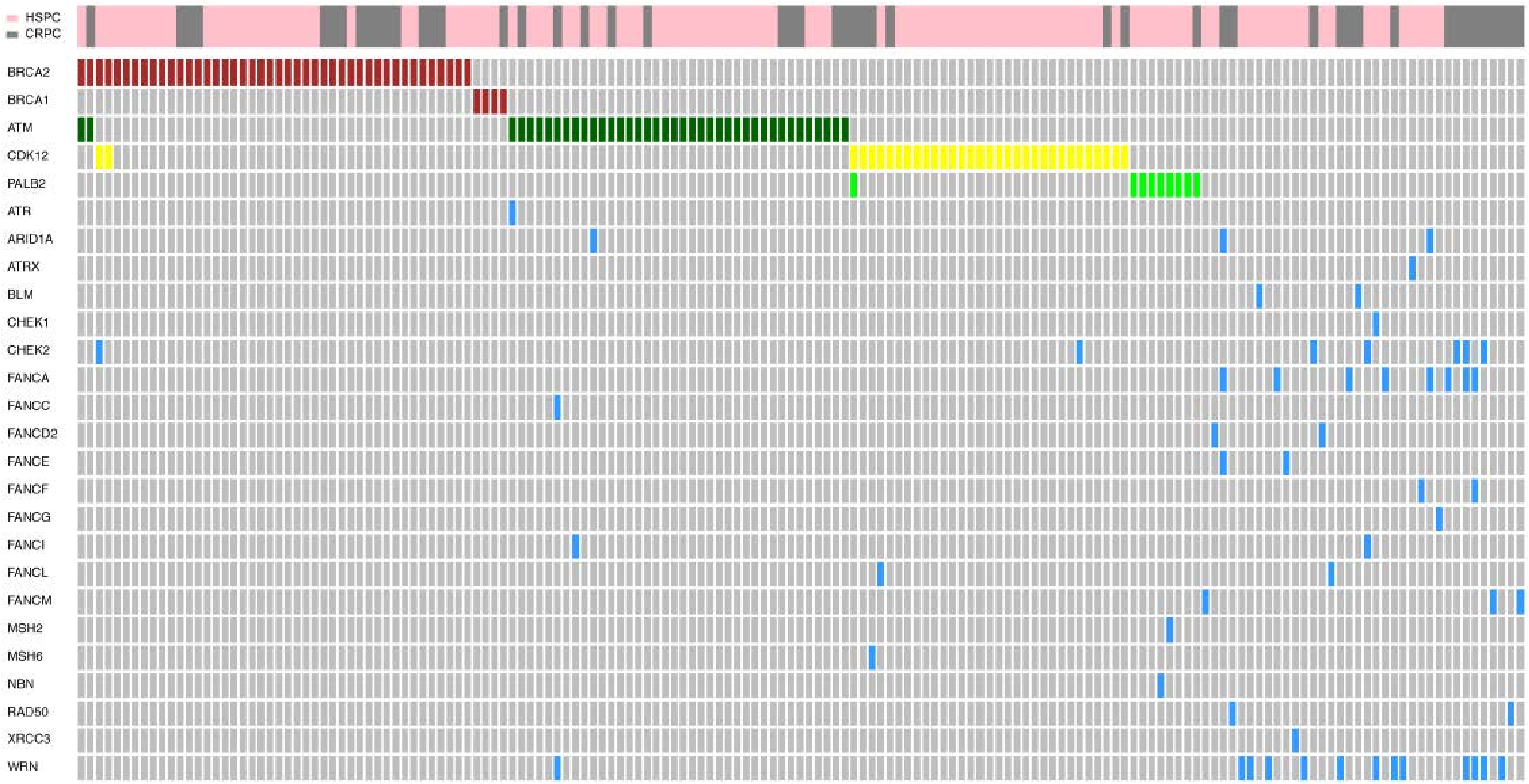

Figure S2 - Landmark analyses for Radiographic Progression Free Survival and Overall Survival, at landmark time-points 8 and 12-weeks

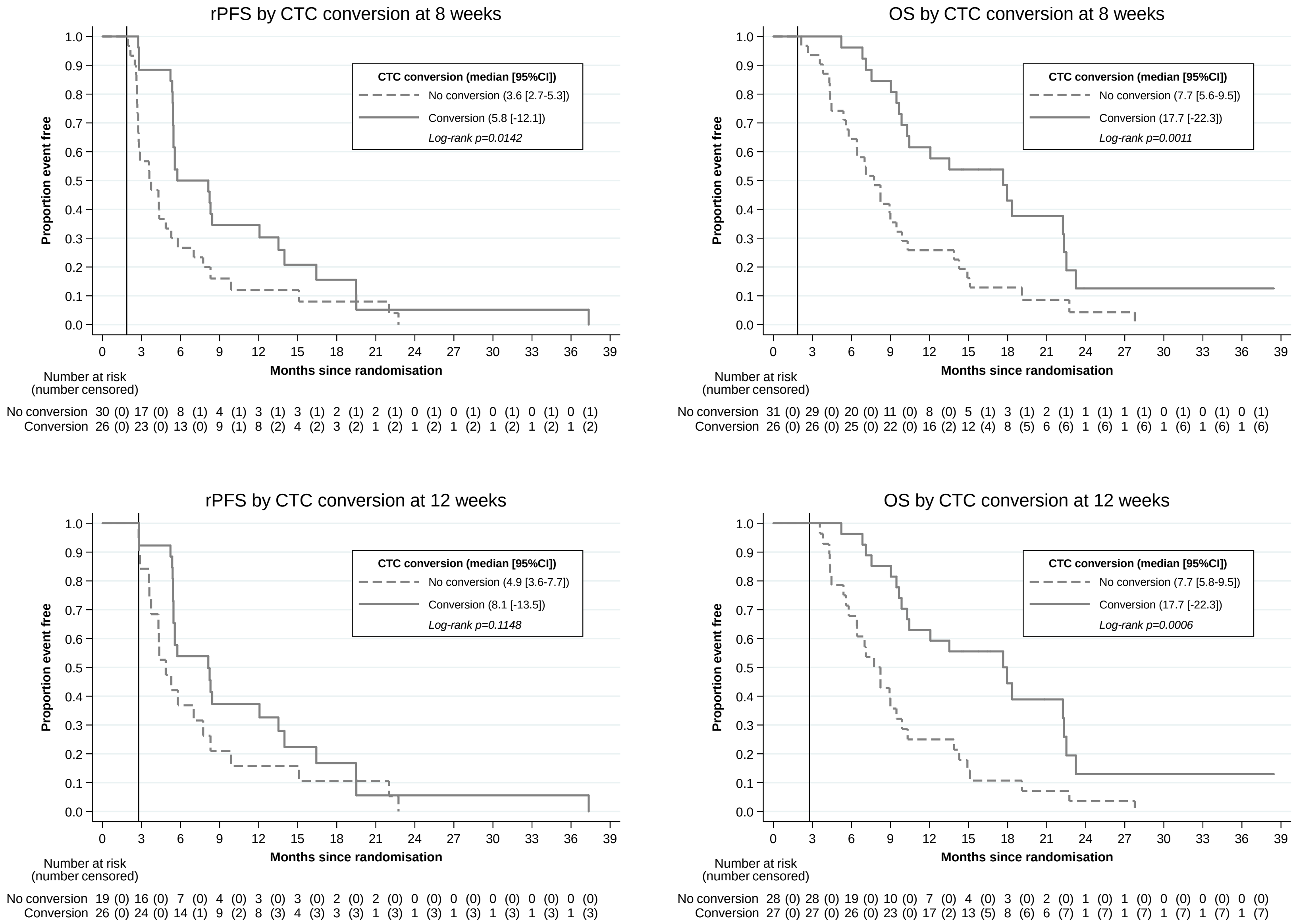

Figure S3: Secondary endpoints results: (A) Progression Free Survival (B) Overall Survival

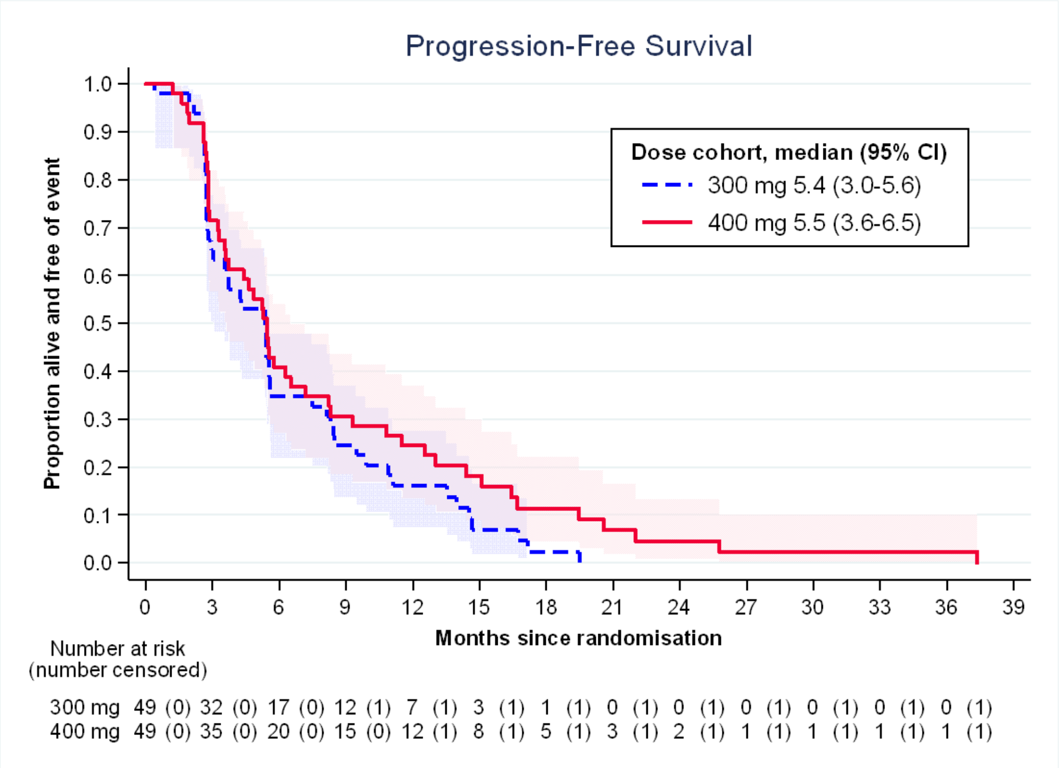

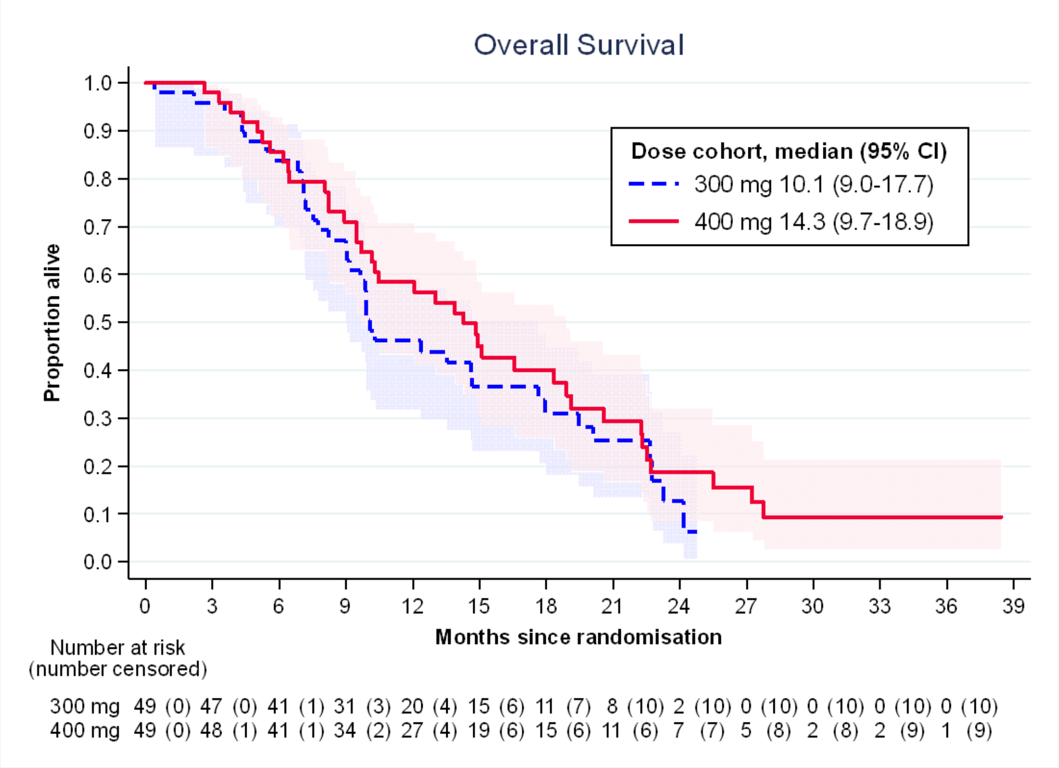

Figure S4: Secondary endpoints results: (A) Time to PSA progression (B) Time to Radiographic Progression

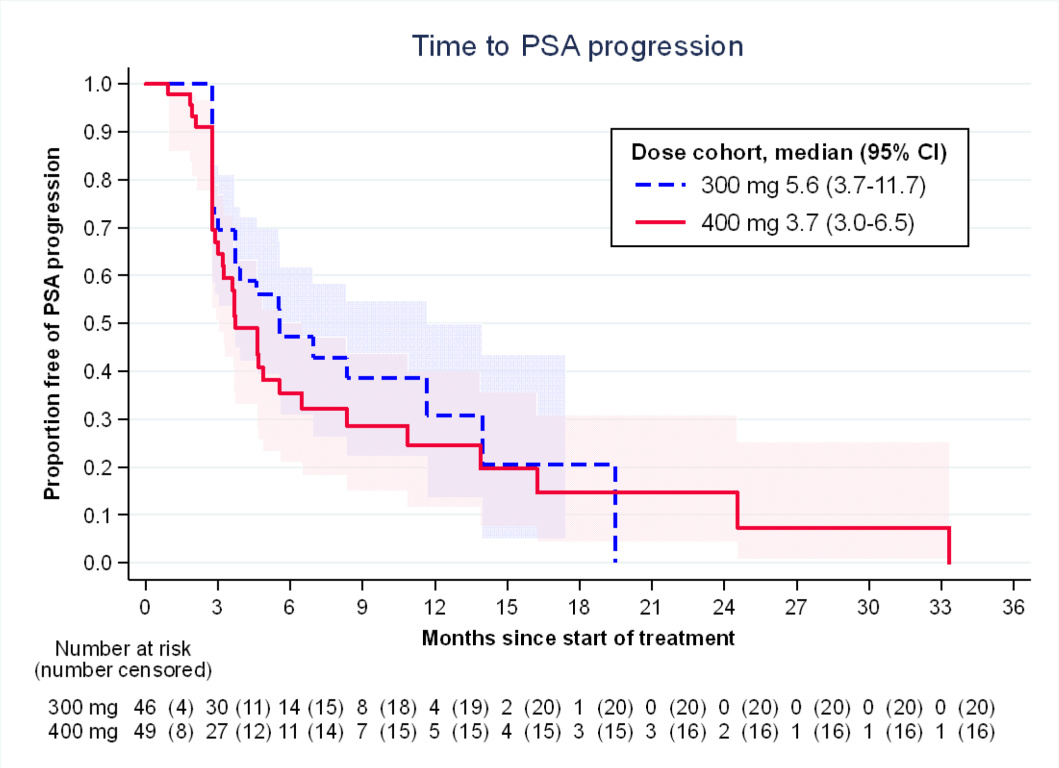

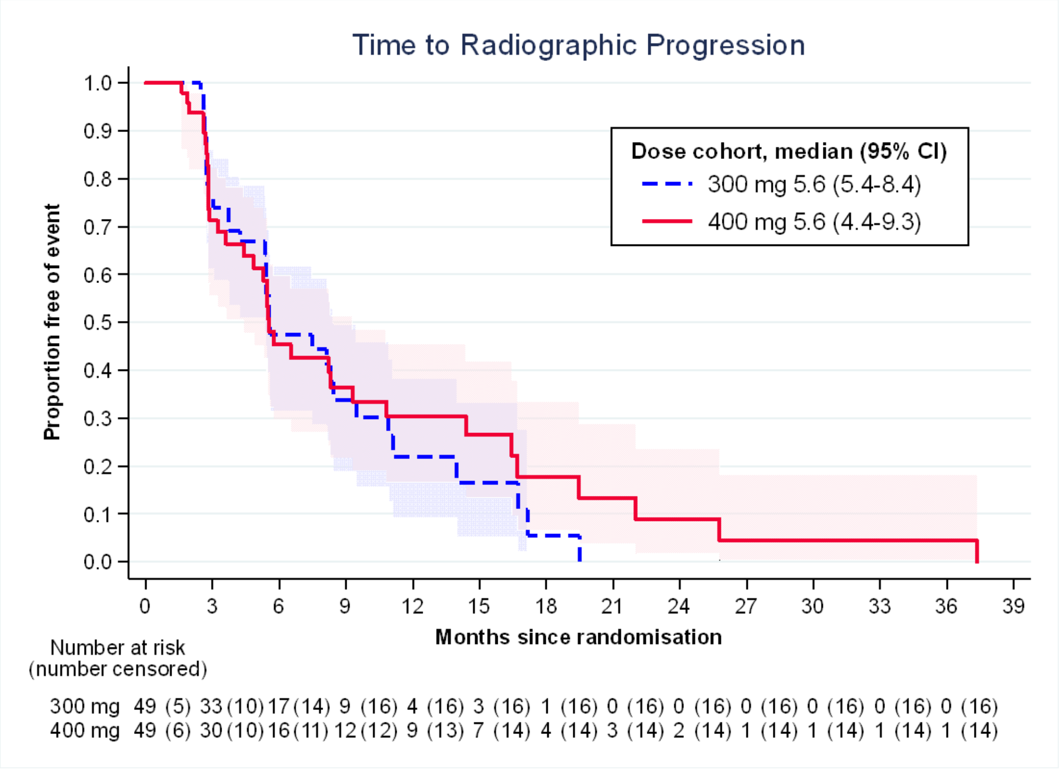

Figure S5: Secondary endpoints results: (A) Percentage change from baseline in PSA at 12 weeks (or earlier if allocated treatment discontinued) (B) Duration of PSA response

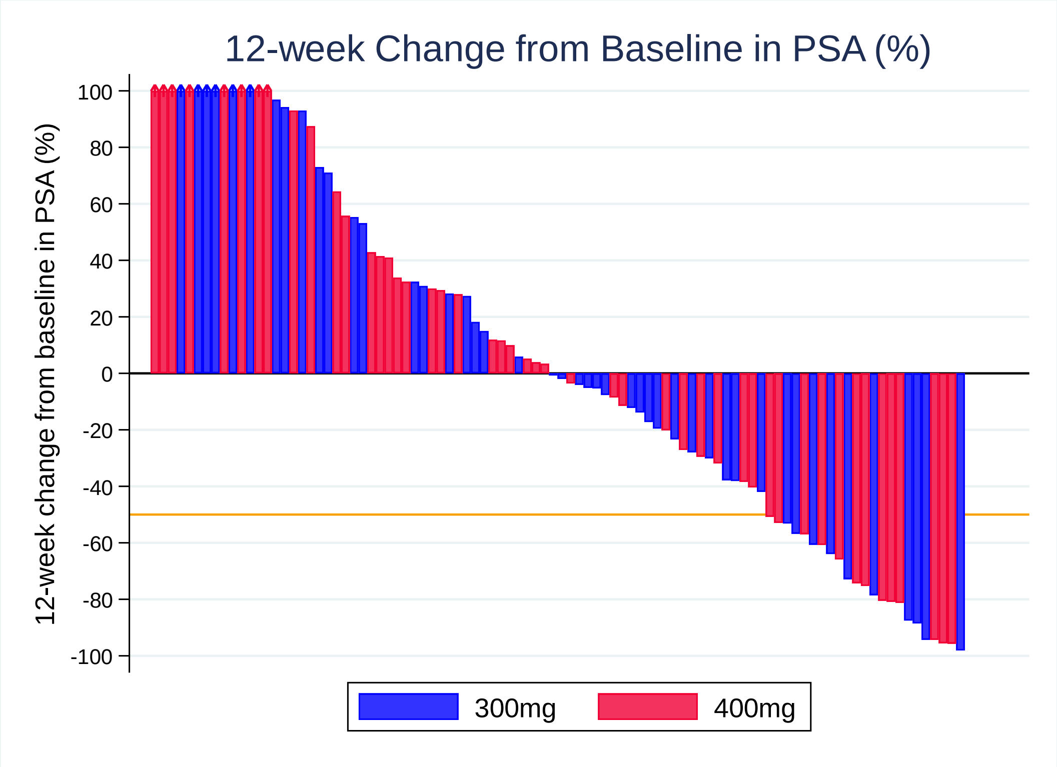

Duration of PSA response, by dose group (ITT population)

	Total	Dose group	
		**300 mg**	**400 mg**
N (PSA responders)	31	14	17
Median	4.6	5.1	4.6
95% CI	3.1-8.2	2.6-10.0	3.1-11.1

95% CI: 95% confidence interval

Figure S6: Survival data by gene subgroup: (A) Progression Free Survival; (B) Overall Survival

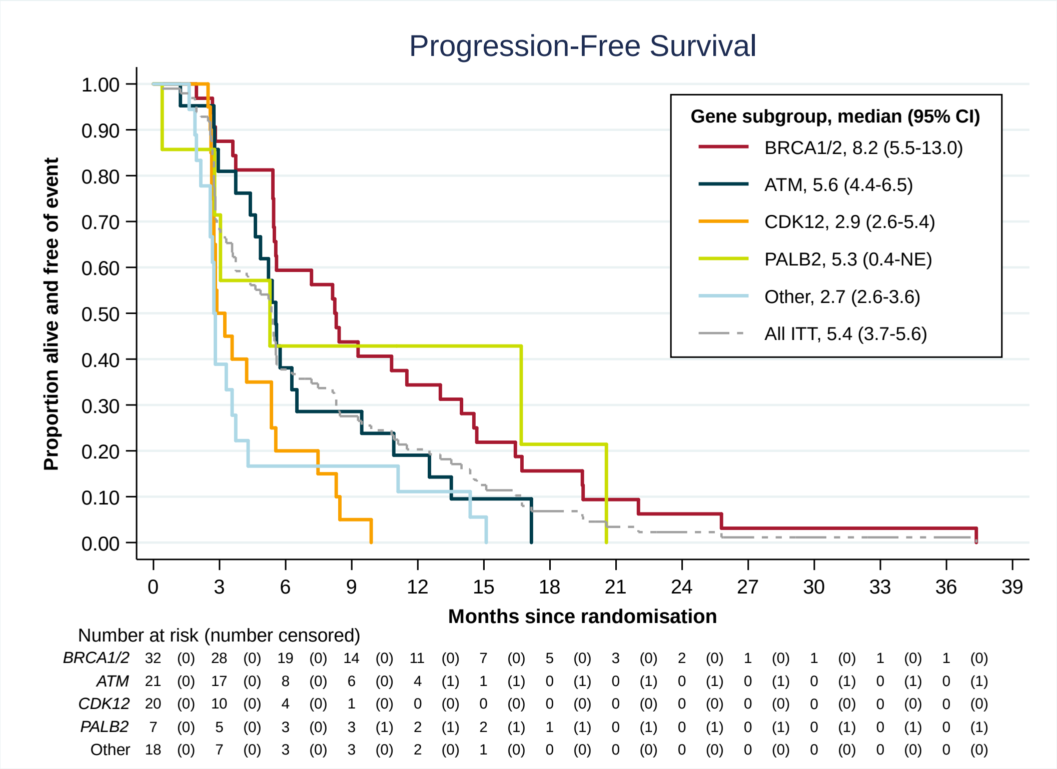

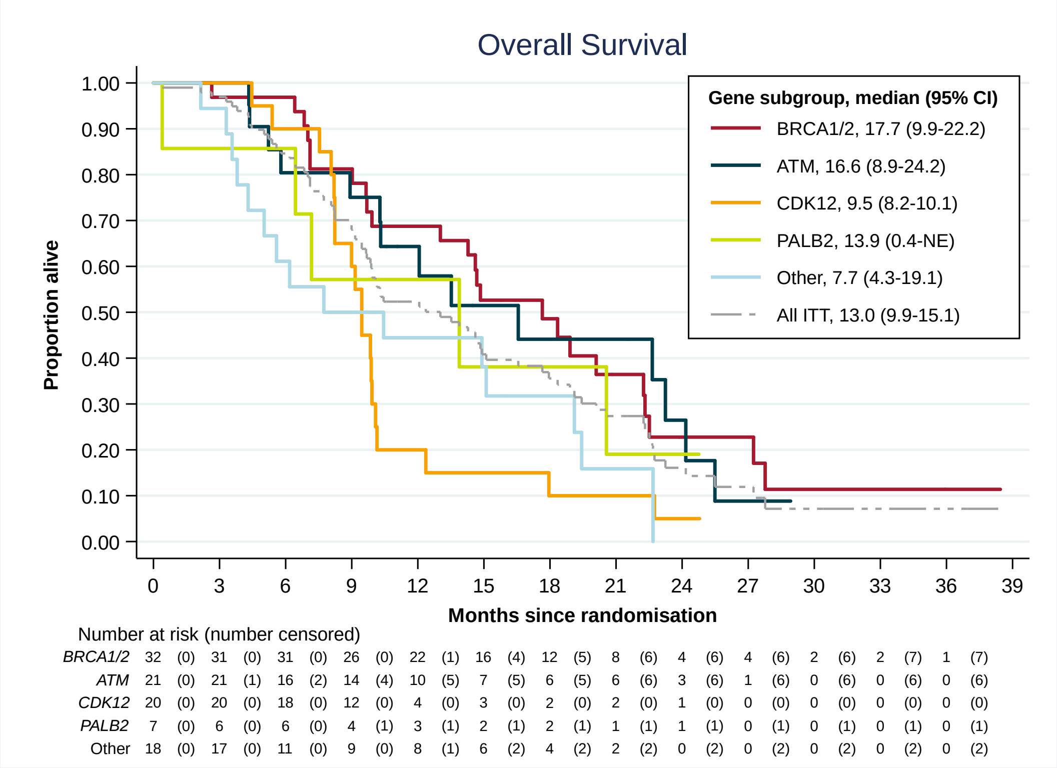

TOPARP
Phase II Trial of Olaparib in Patients with Advanced Castration Resistant Prostate Cancer

CONFIDENTIAL

**Chief Investigator**	Prof. Johann de Bono
**Co-Sponsors:**	The Royal Marsden Hospital NHS Foundation Trust / The Institute of Cancer Research
**Approval:**	Cancer Research UK Clinical Trials Awards & Advisory Committee (CTAAC) and Biomarkers Imaging Development and Discovery Committee (BIDD)
**Funders:**	Cancer Research UK<br>AstraZeneca
**Coordinating Trials Unit:**	Institute of Cancer Research Clinical Trials and Statistics Unit (ICR-CTSU)

PROTOCOL
VERSION 7.0 15th March 2018

EudraCT Number: 2011-000601-49

CTA Number: 22138/0016/001-0001

ICR-CTSU Protocol Number: ICR-CTSU/2011/10030

ICR/RMH CCR Number: 3592

Main REC Reference Number: 11/LO/2019

ISRCTN: 15124653

CR UK Reference Number: CRUK/C12540/A12354

The TOPARP trial is part of the National Institute for Health Research Clinical Research Network Trial Portfolio

CANCER RESEARCH UK

ICR The Institute of Cancer Research

This protocol is a controlled document and should not be copied, distributed or reproduced without the written permission of the ICR-CTSU

ADMINISTRATION

Scientific Coordination

Prof. Johann de Bono (Chief Investigator)

Royal Marsden NHS Foundation Trust &
The Institute of Cancer Research
Downs Road, Sutton
Surrey SM2 5PT
Tel: 0208 722 4028
Fax: 0208 642 7979
Johann.de-Bono@icr.ac.uk

Trial Coordination

ICR-CTSU (a UKCRC registered and NCRI cancer clinical trials unit) is responsible for the day to day conduct of the trial.

Clinical Trials & Statistical Unit (ICR-CTSU),
Division of Clinical Studies
The Institute of Cancer Research,
Sir Richard Doll Building,
Cotswold Road
Sutton, Surrey SM2 5NG

ICR-CTSU Scientific Lead:
Prof. Emma Hall
Tel: 0208 722 4013
Emma.Hall@icr.ac.uk

Clinical Co-ordinator
Dr. Joaquin Mateo
Joaquin.Mateo@icr.ac.uk

ICR Central Lab:
Ms. Susana Miranda
Tel: 0208 722 4083
Susana.Miranda@icr.ac.uk

ICR-CTSU Senior Trial Manager:
Mrs. Aude Espinasse
Tel: 0208 722 4509
Aude.Espinasse@icr.ac.uk

ICR-CTSU Senior Statistician:
Dr. Nuria Porta
Tel: 0208 722 4149
Nuria.Porta@icr.ac.uk

TOPARP Trial Managers:
Mr. Peter Chatfield & Miss Marie Hyslop
Tel: 0208 722 4264/4305
toparp-icrctsu@icr.ac.uk

TOPARP Trial Statistician
Dr. David Dolling
Tel: 0208 722 4008
david.dolling@icr.ac.uk

Any questions relating to this protocol should be addressed in the first instance to the TOPARP Trial Manager within ICR-CTSU:

Email: toparp-icrctsu@icr.ac.uk
General enquiries: 0208 722 4264/4305
Fax: 0208 770 7876

Protocol Development Group

Mr Roger A'Hern	ICR-CTSU, The Institute of Cancer Research, Sutton, London
Dr Gerhardt Attard	The Institute of Cancer Research, Sutton, London
Prof Johann de-Bono	The Institute of Cancer Research, Sutton, London
Dr Suzanne Carreira	The Institute of Cancer Research, Sutton, London
Prof David Dearnaley	The Institute of Cancer Research, Sutton, London
Prof Rosalind Eeles	The Institute of Cancer Research, Sutton, London
Mrs Penny Flohr	The Institute of Cancer Research, Sutton, London
Miss Alexa Gillman	ICR-CTSU, The Institute of Cancer Research, Sutton, London
Dr Emma Hall	ICR-CTSU, The Institute of Cancer Research, Sutton, London
Prof. Jorge Reis Filho	The Institute of Cancer Research, Chelsea, London
Dr Robert Jones	Beatson Oncology Centre, Glasgow
Ms Eleftheria Kalaitzaki	ICR-CTSU, The Institute of Cancer Research, Sutton, London
Dr Chris Lord	The Institute of Cancer Research, Chelsea, London
Prof Malcolm Mason	Velindre Hospital, Cardiff
Dr. Joaquin Mateo	The Institute of Cancer Research, Chelsea, London
Dr Shahneen Sandhu	Peter MacCallum Cancer Centre, Melbourne, Australia previously the Institute of Cancer Research, Sutton
Dr Martine Usdin	ICR-CTSU, The Institute of Cancer Research, Sutton, London

The Trial Management Group (TMG) will be constituted from members of the Protocol Development Group and Principal Investigators from a subset of participating centres. A copy of the current membership of the TMG can be obtained from the TOPARP Trial Manager at ICR-CTSU.

Protocol Authorised by:

Name & Role	Signature	Date
Prof. Johann de Bono (Chief Investigator)	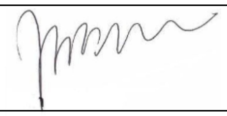	15th March 2018

This protocol describes the TOPARP trial and provides information about procedures for entering patients. The protocol should not be used as a guide for the treatment of other patients. Every care has been taken in the preparation of this protocol, however corrections or amendments may be necessary. These will be circulated to investigators in the trial. Centres entering patients for the first time are advised to contact ICR-CTSU to confirm they have the most recent version. Protocol amendments will be circulated to participating centres as they occur.

This trial will adhere to the principles outlined in the Medicines for Human Use (Clinical Trials) Regulations 2004 (SI 2004/1031) as amended. It will be conducted in compliance with the protocol, the Data Protection Act (Z6364106) and other regulatory requirements as appropriate.

Glossary of Abbreviations and Definitions of Terms

AD	Androgen deprivation
ADP	Adenosine diphosphate
AE	Adverse Event
ALP	Alkaline Phosphatase
ALT	Alanine Transaminase (SGPT)
APTT	Activated partial thromboplastin time
AST	Aspartate Transaminase (SGOT)
AR	Androgen receptor
AZ	AstraZeneca
BID	Twice daily
BER	Base excision repair
BP	Blood Pressure
BUN	Blood urea nitrogen
ºC	Degrees Celsius
FBC	Full Blood Count
cm	Centimetre
CR	Complete Response
CrCl	Creatinine clearance
CRF	Case Report Form
eCRF	Electronic Case Report Form
CRPC	Castration Resistant Prostate Cancer
CT	Computerised Tomography
CTC	Circulating Tumour Cell
CTCAE	Common Terminology Criteria for Adverse Events
D	Day (as in treatment day)
dL	Decilitres
DLT	Dose Limiting Toxicity
DNA	Deoxyribonucleic acid
DRE	Digital rectal exam
DSB	Double Strand Breaks
DW-MRI	Diffusion-weighted MRI
ECG	Electrocardiogram
ECOG	Eastern Co-operative Oncology Group
EMA	European Medicines Agency
FDA	Food and Drug Administration
FISH	Fluorescent in-situ hybridisation
FBC	Full Blood Count
GCIG	Gynecologic Cancer Intergroup
GCP	Good Clinical Practice
G-CSF	Granulocyte colony-stimulating factor
GGT	Gamma glutamyltransferase
GM-CSF	Granulocyte Macrophage Colony Stimulating Factor
Ha	Alternative Hypothesis
H2AX	Histone H2AX
Ho	Null hypothesis
HR	Homologous Recombination
IB	Investigator Brochure
IC50	Dose for 50% inhibition
ICH	International Conference on Harmonisation
IDMC	Independent Data Monitoring Committee
IEC	Independent Ethics Committee

IF	Immunoflourescence
IHC	Immunohistochemistry
IMP	Investigational Medicinal Product
INR	International Normalised Ratio
IRB	Institutional Review Board
ITT	Intention To Treat
kg	Kilogram
LD	Longest Diameter
LDH	Lactic Dehydrogenase
LHRH	Luteinizing Hormone Releasing Hormone
mg	Milligram
MRI	Magnetic Resonance Imaging
miRNAs	MicroRNAs
NAD	Nicotine adenine dinucleotide
NCI	National Cancer Institute
NCICTC	National Cancer Institute Common Terminology Criteria
nM	Nanomolar
NRS	Numeric Rating Scale
ORR	Overall Response Rate
PARP	Poly (ADP-ribose) polymerase
PBMC	Peripheral Blood Mononuclear Cells
PD	Progressive Disease
PD	Pharmacodynamic
PFS	Progression Free Survival
PICF	Patient Informed Consent Form
PIN	Prostatic Intraepithelial Neoplasm
PK	Pharmacokinetic
PO	Per Oral (by mouth)
PR	Partial Response
PSA	Prostatic Specific Antigen
PCWG2	Prostate Cancer Working Group 2
PT	Preferred Term
PTEN	Phosphatase and Tensin Homologue
RBC	Red Blood Cells
RECIST	Response Evaluation Criteria in Solid Tumours
SAE	Serious Adverse Event
SD	Stable disease
SUSAR	Serious unexpected suspected adverse (drug) reaction
SBP	Systolic blood pressure
SSB	Single Strand Break
SOC	System Organ Class
µM	Micromolar
ULN	Upper Limit of Normal
UA	Urinalysis
WBC	White Blood Cells

CONTENTS

1 TRIAL SUMMARY 9
2 INTRODUCTION 13
2.1 Metastatic Castration Resistant Prostate Cancer 13
2.2 PARP and PARP Inhibition 17
2.3 Rationale for Study 19
3 BACKGROUND THERAPEUTIC INFORMATION 23
3.1 Pre-clinical Experience with Olaparib 23
3.2 Clinical Experience with Olaparib 24
3.2.1 Pharmacokinetic (PK), Pharmacodynamic (PD) Data 24
3.2.2 Efficacy Data 25
3.3 Summary of Clinical Toxicity Data 26
4 TRIAL OBJECTIVES 32
4.1 Primary Objective 32
4.2 Secondary Objectives 32
4.3 Tertiary/exploratory Objectives 32
5 TRIAL ENDPOINTS 33
5.1 Primary Endpoints 33
5.2 Secondary Endpoints 33
5.3 Tertiary/exploratory Endpoints 33
6 TRIAL DESIGN 34
6.1 Study Flow Chart 36
7 PATIENT SELECTION & ELIGIBILITY 37
7.1 Number of Patients 37
7.2 Source of Patients 37
7.3 Inclusion Criteria 37
7.4 Exclusion Criteria 40
7.5 Restrictions 41
8 OVERVIEW OF STUDY PROCEDURES 43
8.1 Informed Consent 43
8.2 Part A - Registration, Eligibility and Trial Entry 43
8.2.1 Registration Part A 43
8.2.2 Eligibility status and trial entry Part A 44
8.3 Part B - Registration, Eligibility and Randomisation 44
8.3.1 Pre-registration Part B 44
8.3.2 Registration Part B 45
8.3.3 Eligibility status and randomisation to Part B 45
9 OVERALL STUDY ACTIVITIES 47
9.1 Pre-screening period – Part B only 47
9.2 Screening Period 47
9.3 Treatment Period 47
9.4 End of Treatment (EOT) Visit 48
9.5 Follow Up Visits 48
10 OVERALL TRIAL ASSESSMENTS 49
10.1 Clinical Measurements 49
10.2 Medical History 49
10.3 Physical Examination 49
10.4 ECG Monitoring 50
10.5 Brief Pain Scores 50
10.6 Blood, Tissue and Other Sampling 50
10.7 Efficacy Measurements 52
10.8 Safety Measurements 54

10.9 Criteria For Discontinuation Of Study Treatment 54
10.10 Withdrawal from Study Treatment 56
10.11 Patient Replacement Policy 57
11 TRIAL ASSESSMENTS BY VISIT 58
11.1 Baseline Assessments 58
11.1.1 Pre-screening assessments (Part B only) 58
11.1.2 Screening 58
11.1.3 Cycle 1 Day 1 60
11.2 On-treatment Assessments 60
11.2.1 Cycle 1 Day 8 Visit 60
11.2.2 Cycle 1 Day 15 Visit – Part A only 61
11.2.3 Day 1 of Cycles 2, 3, 5, 6, 8, 9, 11, and 12 61
11.2.4 Cycles 4, 7, and 10 62
11.2.5 Dose Escalation (300mg group only) 64
11.2.6 End of Treatment (EOT) Visit 64
11.2.7 3 monthly follow up (3 monthly from EOT) 65
11.3 Unscheduled Visits 65
11.4 Early Discontinuation 66
11.5 Schedule of Procedures 67
12 TRIAL TREATMENT 71
12.1 Drug Manufacturer 71
12.2 Presentation / Formulation of Olaparib 71
12.3 Drug, Dose and Schedule 71
12.4 Instructions for Administration of Olaparib 71
12.5 Olaparib Treatment Compliance 71
12.6 Duration of Therapy 72
12.7 Supportive Care Guidelines 72
12.8 Guidelines for Management of Toxicity and Dose- Modification 73
12.9 Packaging and Labelling 75
12.10 Drug Storage 75
12.11 Distribution of Drugs to Site 76
12.12 Pharmacy Responsibilities and Drug Accountability 76
12.13 Concurrent Medications 77
13 PHARMACOVIGILANCE 80
13.1 Definitions 80
13.2 Clinical & Laboratory Adverse Events 81
13.3 Causality 82
13.4 Severity of Adverse and Serious Adverse Events 82
13.5 Procedures for Recording and Reporting Adverse Events 84
13.5.1 Reporting Serious Adverse Events (SAEs) to ICR-CTSU 84
13.6 Review of Serious Adverse Events (SAEs) or Reactions (SARs) 85
13.7 Expedited Reporting of SUSARs 85
13.8 Follow up of Serious Adverse Events 85
13.9 Annual Reporting of Serious Adverse Reactions 85
14 STATISTICAL CONSIDERATIONS 87
14.1 Trial Design 87
14.2 Treatment Allocation 89
14.3 Analysis populations 90
14.4 Primary Efficacy Endpoint 90
14.5 Secondary Efficacy Endpoints 91
14.6 Tertiary/exploratory Endpoints 92
14.7 Data Monitoring 93
15 TRANSLATIONAL RESEARCH 94
15.1 Archival Tumour Blocks and Fresh Biopsies 95
15.2 Circulating Tumour Cell (CTC) Assessments 95
15.3 Plasma and Urine-Based Biomarkers 95

15.4 Buccal Swabs **96**
15.5 Saliva Collection **96**
15.6 Diffusion-weighted MRI **96**
15.7 Rationale of translational research **97**
15.8 Whole Genome Sequencing **97**
16 TRIAL MANAGEMENT 100
16.1 Trial Management Group (TMG) **100**
16.2 Trial Steering Committee (TSC) **100**
16.3 Independent Data Monitoring Committee (IDMC) **100**
17 RESEARCH GOVERNANCE 101
17.1 Sponsor Responsibilities **101**
17.2 Principal Investigator Responsibilities **101**
17.3 AstraZeneca Responsibilities **102**
18 TRIAL ADMINISTRATION & LOGISTICS 103
18.1 Protocol Compliance **103**
18.2 Data Acquisition **103**
18.3 Central Data Monitoring **104**
18.4 On-Site Monitoring **104**
18.5 End of trial **104**
18.6 Archiving **104**
19 PATIENT PROTECTION AND ETHICAL CONSIDERATIONS 105
19.1 Patient Confidentiality **105**
19.2 Data Protection **106**
19.3 Liability **106**
20 FINANCIAL MATTERS 107
21 PUBLICATION POLICY 107
22 REFERENCE LIST 108
23 APPENDICES 113
23.1 Appendix A: Part A - Detectable Effects **113**
23.2 Appendix B: Sample Size Calculations **114**
23.3 Appendix C: Evidence of PD to Determine Trial Entry Eligibility **118**
23.4 Appendix D: Outcome Measures of Response / Progression Post Study **119**
23.5 Appendix E: Modified RECIST1.1 .... **121**
23.6 Appendix F: ECOG Performance Status **125**
23.7 Appendix G: National Cancer Institute Common Terminology Criteria for Adverse Events (version 4.02) **126**
23.8 Appendix H: World Medical Association Declaration of Helsinki **126**
23.9 Appendix I: Brief Pain Inventory (Short Form) **127**
23.10 Appendix J: Total blood volumes drawn at each time point **129**

1 TRIAL SUMMARY

**PROTOCOL TITLE**	Phase II Trial of Olaparib in Patients with Advanced Castration Resistant Prostate Cancer (TOPARP).
**TARGET DISEASE**	Castration resistant prostate cancer (CRPC)
**STUDY OBJECTIVES**	• To evaluate the anti-tumour activity of olaparib in metastatic castration resistant prostate cancer<br>• To identify molecular signatures of tumour cells in responding and non-responding patients<br>• To clinically qualify putative predictive biomarkers of PARP inhibitor sensitivity
**STUDY DESIGN**	TOPARP- A Open-label, single arm, two part adaptive design phase II trial.<br>TOPARP-B: Open-label, two-arm randomised, each arm with a single stage phase II design.
**TRIAL POPULATION**	Patients with an advanced CRPC who have failed androgen deprivation and taxane-based treatment.
**TREATMENT REGIMEN**	TOPARP-A: Patients will receive single agent olaparib at a dose of 400 mg twice daily, continuously on a 28-day cycle. Olaparib will be administered until objective disease progression or unacceptable toxicity or patient withdrawal for whatever reason.<br><br>TOPARP-B: patients will be randomised to receive 300mg or 400mg twice daily, continuously on a 28-day cycle. Olaparib will be administered until objective disease progression or unacceptable toxicity or patient withdrawal for whatever reason. Patients randomised to the 300mg group will be offered the option of dose escalation to 400mg twice daily upon confirmation of disease progression if clinically indicated.
**RECRUITMENT TARGET**	TOPARP-A: between 30-45 patients<br>TOPARP-B: 88 patients (44 in each group)
**PRIMARY ENDPOINT**	Response will be defined on the basis of the following outcomes, if any of these occur patients will be considered to have responded:<br>• Objective response by modified RECIST1.1 (appendix E)<br>• PSA decline of ≥50% according to the Prostate Cancer Working Group 2 (PCWG2)<br>• Conversion of circulating tumour cell count (CTC) from ≥5 cells/7.5 ml blood at baseline to <5 cells/7.5 ml confirmed by a second consecutive value obtained four or more weeks later

**SECONDARY ENDPOINTS**	• Radiological progression free survival<br>• Time to radiological progression<br>• Progression free survival<br>• Overall survival<br>• Time to PSA progression<br>• Proportion of patients with conversion of CTC count from ≥5/7.5ml blood at baseline to <5/7.5 ml blood nadir<br>• Duration of PSA response, if applicable<br>• Percentage of change in PSA from baseline to 12 weeks (or earlier if discontinued therapy) and maximum PSA decline while on treatment.<br>• Safety and tolerability of olaparib<br>• TOPARP-B only - Cmax and AUC after first dose and at steady state for the 400mg and 300mg BID dose levels.
**EXPLORATORY AIMS**	• To evaluate the impact of treatment with olaparib on bone metastasis imaging by DW-MRI in selected patients and to correlate these results with PSA, CTC and radiological responses by conventional imaging.<br>• To determine the proportion of patients achieving pain improvement with olaparib treatment.<br>• TOPARP-B only - to evaluate response rate following dose-escalation from 300mg to 400mg twice daily.
**TRIAL OUTLINE**	• There will be screening period of up to 28 days prior to cycle 1 day 1. (in TOPARP-B only - post confirmation of the presence of the putative predictive biomarkers)<br>• Archival tissue is mandated for enrolment into the study. Patients who do not have a prior histological diagnosis or archival tissue available will be required to have a tumour biopsy. TOPARP-A will be open to all comers. In TOPARP-B the tumour tissue of the patients being considered for enrolment will be analysed first to ensure the presence of the putative predictive biomarkers (pre-screening stage) and only patients with the predictive biomarkers will be enrolled.<br>• Patients will receive single agent 400mg of olaparib, twice daily, continuously on 28-day cycles. In TOPARP-B, a second cohort will be enrolled at a dose of 300mg twice daily to study the tolerability and antitumor activity of both dose levels.<br>• During the study period, no other anticancer or hormonal treatment will be given although the use of LHRH agonists/antagonists must be continued, unless the patient is surgically castrate.<br><br>**Efficacy Assessments:**<br>• Baseline CT scans, bone scan and DW-MRI (optional) will be performed as close as possible to cycle 1 day 1 of treatment.

	These scans will be repeated after every 3 cycles (12 weeks) or earlier if clinically indicated. If a patient remains on treatment for >2 years, CT/MRI and bone scans should be undertaken every 3rd cycle or as per SOC but at least every 24 weeks.<br>• PSA will be assessed at baseline and every 12 weeks by PCWG2 criteria. In the absence of radiological disease progression patients should continue on study.<br><br>**Safety Assessments:**<br>Safety parameters will be continuously assessed throughout the study prior to each cycle of treatment. Adverse events (AEs) and serious adverse events (SAEs) including laboratory tests will be graded and summarised according to the National Cancer Institute (NCI) Common Toxicity Criteria for Adverse Events version 4.02 (CTCAE v4.02).<br><br>Based on Phase I and Phase II studies, olaparib is generally well tolerated. Safety concerns are mainly related to the possibility of myelosupression. Patients who experience a NCI-CTCAE grade 3-4 haematological or non-haematological toxicity that the investigator attributes to olaparib may have an interruption of olaparib treatment for up to 42 days until recovery of all toxicities to no more than NCI-CTCAE grade 1. Those patients on continuous treatment requiring a delay in dosing by >14 days will require a dose reduction to 300mg or 250mg BID depending on the starting dose of olaparib. If the adverse event recurs at the same severity, treatment can be interrupted again and on resolution to ≤ NCI-CTCAE grade 1 toxicity a further dose reductions to 250 and 200 mg BID can be made.<br><br>Olaparib should be withheld in the event of:<br>• Grade 4 neutropenia or leukopaenia<br>• Grade 2-4 neutropenia or leukopaenia with infection<br>• Platelet count lower than 50 x 10^9/L<br>• Non-haematological toxicity grade 3 or greater, except nausea, vomiting or diarrhoea and fatigue associated with suboptimal pre-medication and/or management OR transient and clinically insignificant biochemical blood results.<br>• Aspartate transaminase (AST) or alanine transaminase (ALT) elevations grade 3 or higher for more than 7 days.<br>• New or worsening pulmonary symptoms or radiological abnormality occurs which are suggestive of pneumonitis<br>• Any other toxicity that, in the view of the principal investigator and the medical monitor, represents a clinically significant hazard to the patient until resolution of toxicity to no more than grade 1.<br><br>**Biomarker Assessments:**<br>All patients enrolled in the study are required to have archival tumour

	tissue, fresh tumour biopsies (TOPARP-A), blood and urine samples collected during the study for the purpose of biomarker analyses including the following:<br><br>• Circulating tumour cells (CTCs): enumeration & molecular characterisation<br>• PTEN and ERG immunohistochemistry (IHC) on primary tumour tissue and fresh paired biopsies<br>• PTEN and ERG immunoflourescence (IF) in CTCs<br>• *TMPRSS2-ERG* and *PTEN* fluorescence in situ hybridisation (FISH) studies in primary tumour, paired biopsies and CTCs<br>• γH2AX and RAD51 foci formation in paired tumour biopsies and CTCs<br>• Markers of tumour cell proliferation and cell cycle phase in paired tumour biopsies and CTCs<br>• Circulating plasma tumour DNA<br>• Circulating plasma tumour and whole blood RNA<br>• Urine telopeptides and RNA<br>• Proteomic profile<br>Metabolomics profile
**FOLLOW UP**	Patients will be treated until disease progression (radiological or clinical progression) or withdrawal from the study for another reason. After discontinuing study treatment patients will be reviewed within 30 +/- 5 days post cessation of treatment for an end of treatment safety assessment and 3 monthly for survival data.<br><br>For TOPARP-B patients allocated to the 300mg group, dose escalation to 400mg upon progression will be allowed if clinically indicated. In that case, patients will be reviewed every 4 weeks until disease progression (radiological progression) or withdrawal from the study for another reason on the 400mg dose.<br><br>Patients who required any dose reduction from 300mg twice daily would not be eligible for dose-escalation to 400mg twice daily.

2 INTRODUCTION

2.1 Metastatic Castration Resistant Prostate Cancer

Prostate cancer is the second leading cause of male cancer-related mortality in the United Kingdom (1). While a proportion of patients have indolent disease, curable with local treatment, approximately a third of patients fail local treatment and develop incurable metastatic disease. Androgen deprivation therapy constitutes the mainstay of treatment for advanced prostate cancer. Most patients with advanced disease initially respond to hormonal deprivation therapy; however resistance inevitably develops within 1-3 years and represents a transition to more aggressive and invariably fatal disease. Recent studies have established that androgen signalling continues to play a pivotal role in tumour progression as castration resistance evolves. Androgen receptor (AR) gene amplification, AR overexpression, AR activating mutations and the presence of sufficient intratumoural androgen levels contribute to continued AR signalling despite a systemic castrate state. The recent emergence of the CYP17 inhibitor, abiraterone and other novel antiandrogens such as MDV3100 provide clinical validation that targeting AR signalling remains a useful approach in a significant proportion of CRPC patients (2, 3).

Metastatic prostate cancer is often associated with significant disease-related morbidity. The vast majority of these patients develop bone metastasis with clinical sequelae of bone pain, skeletal fractures, spinal cord compression and haematological compromise. In addition, soft tissue disease is liable to cause urinary obstructions and other problems.

Most CRPC patients have received multiple lines of hormonal manipulation prior to commencing chemotherapy for ongoing disease progression. Docetaxel and more recently cabazitaxel, Sipuleucel-T, alpharadin and abiraterone have demonstrated an overall survival advantage for metastatic CRPC (mCRPC) albeit of only several months (2, 4, 5, 6). Despite these important incremental advances in treatment options, benefits remain modest and the median survival of patients with mCRPC is unsatisfactory at approximately 18 – 36 months. It is clear that there is an urgent need for better treatments that offer durable clinical benefit and further improvement in overall survival for these patients.

Platinum Based Second-line Chemotherapy in Patients with CRPC

Platinum-based chemotherapy in Phase II trials has provided encouraging response rates (PSA and radiological) in the range of 44-60% and a median progression free survival (PFS) of 4-9.5 months in the post-docetaxel CRPC setting (7, 8). Likewise, satraplatin, an orally bioavailable platinum compound, has shown second-line antitumour activity in unselected patients in a Phase III trial, with a 50% PSA decline rate of 33% and a statistically significant PFS advantage in 40% of the patients (9). The lack of survival outcome in this study, despite the anti-tumour activity observed, may in part relate to inter-patient molecular heterogeneity and failure to optimally select the subgroup of tumours most likely to respond to this strategy.

Platinum salts induce interstrand and intrastrand DNA cross-links and consequently increased levels of double-strand (ds) DNA damage. Platinum compounds and PARP inhibitors (PARPi) exert their anti-tumour activity in a similar way by inducing increased ds-DNA breaks which overwhelms the DNA repair machinery in homologous recombination (HR) repair deficient tumours, consequently resulting in tumour cell death. Tumour sensitivity to the Poly (ADP-ribose) polymerase (PARP) inhibitor, olaparib has been shown to correlate with prior platinum sensitivity in HR deficient, *BRCA1/2* mutant cancers (10, 11). It follows therefore that the previously established antitumour activity for satraplatin in prostate cancer supports the widely held premise that there is indeed a subgroup of CRPC that harbour HR DNA repair defects and will benefit from treatment with the PARP inhibitor, olaparib. No platinum is currently approved for the treatment of CRPC; indeed most CRPC remain platinum naïve. Recruitment of patients most likely to benefit based on biomarker driven molecular sub-classification is critically important for the successful development of novel therapies such as olaparib in this disease.

Prostate Cancer Heterogeneity: Clinical and Molecular

Prostate cancer is a highly heterogeneous disease with patients responding differently to treatments and overall having very different clinical outcomes. This is also reflected at a molecular level (12-16). Different molecular variants portend different clinical outcomes possibly secondary to distinct differences in molecular drivers. Despite this, to date, Phase III trials of novel anticancer drugs in this disease have failed to stratify patients on the basis of molecular profile and likely benefit. This inter-patient molecular heterogeneity may, in part, account for the high risk of failure of pivotal Phase III trials of anticancer drugs in this disease. We envision that an improved understanding of the biological and clinical significance of the molecular sub-classification of this disease will positively impact pivotal randomised clinical trials in this disease. The identification of primary tumour prognostic and predictive molecular markers as compared to the currently utilised suboptimal clinico-pathological parameters will allow improved patient selection for optimal CRPC treatments thus maximising clinical benefit while avoiding unnecessarily exposure and toxicity in patients unlikely to benefit.

Dissecting Prostate Cancer Heterogeneity by FISH: *PTEN* & *ETS* Gene Rearrangements.

Functional Loss of *PTEN*

Prostate cancer can be molecularly sub-classified using multi-colour fluorescent in situ hybridisation (FISH). Dissecting the molecular profile of prostate cancers in this way has resulted in the characterisation of multiple commonly occurring genetic aberrations implicated in promoting tumour genesis (12, 15, 17, 18). The biological significance of gene rearrangements, deletions and amplifications as well as possible predictive and prognostic relevance are currently being extensively studied. Phosphatase and tensin homologue (*PTEN*), a tumour suppressor gene, negatively modulates the phosphatidylinositol 3-kinase (PI3)/AKT signal transduction pathway which fosters tumour growth, proliferation and survival (19-22). Bi-allelic and mono-allelic deletion of *PTEN* is a common event in prostate cancer and is implicated in tumour pathogenesis and progression (22-25). PTEN functional loss is associated with more advanced disease, higher-grade tumours and a worse clinical

outcome (15, 24-27). The frequency of reported *PTEN* inactivation in prostate cancer is variable in the literature, with mono-allelic and bi-allelic loss by FISH in the range of 30-68% (23, 26, 27, 28). Our own analysis has shown a complete loss of *PTEN* expression by immunohistochemistry (IHC) in archival tissue in approximately 50% in patients on the abiraterone phase I–II studies. The variability seen in the literature may be related to tumour stage, tissue processing, and IHC methodology. A recent study of tumour material from 59 patients with CRPC revealed that the *PTEN* gene was deleted in 77% of cases; 25% with homozygous deletions, 18% with homozygous and hemizygous deletions and 34% with hemizygous deletions. Bi-allelic *PTEN* loss was correlated with disease-specific mortality in this study (24).

Evidence for the importance of PTEN functional loss in prostate carcinogenesis also comes from transgenic studies. Critically, conditional PTEN loss in prostates from transgenic mice leads to the late development of metastatic prostate cancer (29). Prostate restricted overexpression and activation of AKT, however, resulted only in prostatic intraepithelial neoplasia (PIN) formation (30). These data suggest that PTEN loss may possibly induce prostate carcinogenesis not only through its phosphatase PI3K/AKT regulatory role but also possibly through its nuclear, DNA repair, role that has been recently reported (31, 32).

TMPRSS2 **Chromosomal Translocation**

Chromosomal translocation involving the androgen-responsive gene transmembrane protease serine 2 (*TMPRSS2*) with the oncogenic erythroblast transformation specific (*ETS*) transcription factor family of genes (*ETV1, ETV4, ETV5*) is reported in 50%-70% of prostate cancers (17, 33). *TMPRSS2/ERG* is the predominant type of gene fusion seen and results in overexpression of 3'-ERG sequence joined to the 5'-*TMPRSS2* promoter. Less frequently *TMPRSS2* becomes fused to *ETV1* and *ETV4*. *ERG* and other *ETS* family members are transcription factors that are implicated in the control of cell growth and differentiation. *TMPRSS2/ERG* fusion rearrangement has been shown to co-operate with *PTEN* haploinsufficiency to promote carcinogenesis (17, 22, 34) and accounts for continued hormone driven disease (35). Recent evidence implicates topoisomerase IIB-mediated DNA breakage during the induction of transcriptional programs in generating these *TMPRSS2/ERG* fusion rearrangements (36, 37, 38).

We have utilised validated FISH assays to evaluate *PTEN* deletions and the commonest *ETS* gene rearrangements (*ERG and ETV1*) on the Trans-Atlantic Prostate Group (TAPG) tissue microarray (TMA) (15). We demonstrated that patients with PTEN loss (heterozygous or homozygous: 17% of newly diagnosed cancers) had significantly worse cause-specific and overall survival compared with patients with no *PTEN* loss, supporting the haploinsufficiency proposed for the *PTEN* gene (Figure 1). Three hundred and eight patients with both *ETS* gene (*ERG and ETV-1*) rearrangement and PTEN loss status available were analysed for survival outcome (Figure 2). The largest group of patients (n=167, 54%) had no *ERG* or *ETV-1*-gene rearrangement and no PTEN loss and had the best outcome with 85.5% survival at 11 years. A poor prognosis group was also identified (n=19, 6%) who had PTEN loss but no ERG or *ETV-1*-gene rearrangement with a cause-specific survival of 13.7% at 11 years and a median survival of 5 years from diagnosis. Patients with an *ETS* gene rearrangement with or without PTEN loss had an intermediate prognosis in keeping with the hormonal sensitivity of these cancers, which has recently

been described (15, 27). Overall, therefore, these data indicate that prostate cancer is a molecularly heterogeneous disease, with cancers demonstrating PTEN loss having a poor outcome, and that to accelerate the successful development of targeted therapies in CRPC, identifying the appropriate subgroup of patients most likely to benefit is paramount.

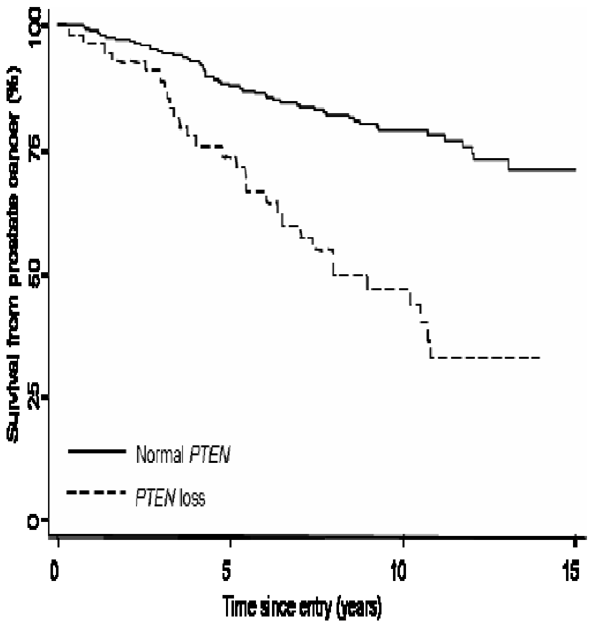

Figure 1: PTEN loss prostate cancers have a worse overall survival

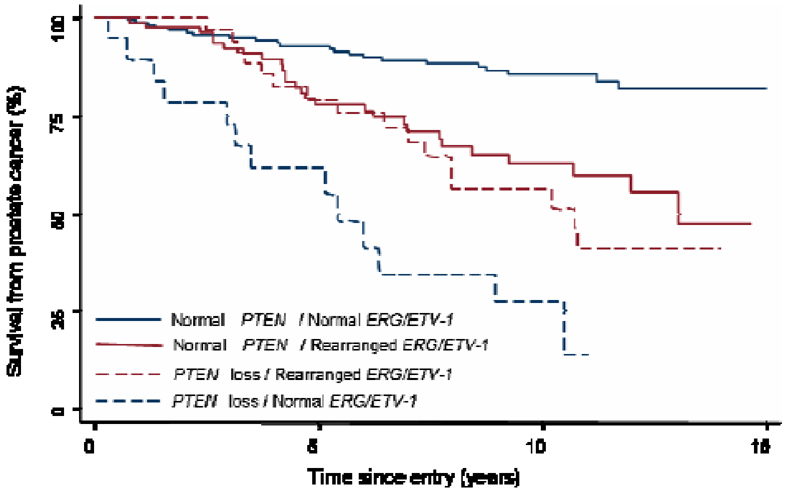

Figure 2: Multi-colour FISH based stratification by studying *ETS* rearrangements and PTEN loss.

PTEN Loss, *ETS* Gene Rearrangements and HR Repair Defects

PTEN is implicated in many functions, both PI3K/AKT dependent and independent. Recent reports suggest in addition to lipid phosphatase function, PTEN localises to the nucleus and that its function includes maintenance of genomic stability (31, 32) by a number of mechanisms including interacting with CENP-C an integral centromeric binding and

stabilising protein, regulating the transcription of RAD51, a critical DNA double strand break (DSB) repair protein (39) and modulating cell cycle progression (40).

PTEN loss results in impaired HR DNA repair with reduced RAD51 expression, increased choromosomal aberrations and genomic instability (31,39). Moreover, in vitro cell-based experiments and in vivo xenograft studies have established that PTEN deficient tumour cells much like *BRCA1/2* deficient tumours are selectively sensitive to platinum compounds and potent PARP inhibitors including olaparib (41-43). Furthermore, restoration of PTEN expression in PTEN deficient tumour cell lines restores PARP inhibitor resistance, confirming PTEN as a determinant of the response to PARP inhibition. This synthetic lethal relationship between PTEN-null tumours and PARP inhibition supports the therapeutic utility of PARP inhibitors in PTEN-deficient tumours. Moreover, *ERG*, the oncogene driven by the commonest prostate cancer genetic driver lesion *TMPRSS2/ERG*, has been shown to directly associate with PARP and requires PARP for its transcriptional regulatory function with PARP inhibition having antitumour activity in *ERG* rearranged tumour models (44,45).

Given these pre-clinical observations and the putative role of PTEN in maintaining chromosomal stability, we predict that tumours with PTEN loss and /or *ETS* gene rearrangements are likely to be hypersensitive to PARP inhibition. We therefore aim to evaluate the antitumour activity of olaparib in patients with sporadic CRPC using an adaptive biomarker driven design to identify and clinically qualify predictive biomarkers of olaparib sensitivity for optimal patient selection.

2.2 PARP and PARP Inhibition

Poly(ADP-ribose) polymerase 1 (PARP-1) is a zinc-finger DNA-binding enzyme that is activated >500 fold upon binding to DNA double or single strand breaks (46). PARP-1 (113 kDa) and PARP-2 (62 kDa) are thus far the only members of the PARP enzyme family known to be involved in DNA damage signalling and repair with a degree of functional redundancy between these proteins. PARP-1 signals the presence of DNA damage and plays a critical regulatory role in DNA damage repair. PARP-1 is involved in a wide range of cellular processes including DNA repair and maintenance of genomic integrity, regulation of protein expression at the transcriptional level, regulation of cellular replication and differentiation, regulation of telomerase activity, and involvement in the cell apoptotic pathway (47).

PARP-1 comprises 3 domains, the N-terminal DNA-binding domain containing 2 zinc fingers, the auto-modification domain and the C-terminal catalytic domain. PARP-1 binds to both single and double-stranded DNA breaks through the zinc-finger domain and catalyses the cleavage of nicotinamide adenine dinucleotide (NAD)+ into nicotinamide and ADP-ribose, and subsequently transfers successive units of ADP-ribose onto DNA, histones, PARP-1 and other DNA repair proteins to form branched ADP-ribose polymers. These branched ADP-ribose polymers result in post-translational modification of histones and other nuclear proteins which convert the DNA damage signal into intracellular signals that activate either DNA base-excision-repair (BER) pathways or cell death if the damage is beyond repair. Other transcriptional factors and signalling molecules are also poly-ADP-

ribosylated by PARP-1 to initiate a multitude of other important cellular functions (47,48). Given the critical role that PARP plays in single strand DNA repair it is not surprising that PARPi are able to potentiate the cytotoxic damage incurred with DNA-damaging chemotherapy agents and ionizing radiation (49). PARP inhibitors have also shown significant single agent activity in tumours with HR deficiency (10,11, 50,51).

Synthetic Lethality Between *BRCA1/2*-mediated Homologous Recombination Repair and PARP-1

Abrogating PARP dependent BER, which is responsible for repairing DNA single strand breaks (SSBs) results in the accumulation of SSBs. These SSBs collapse at the replication forks during DNA synthesis giving rise to double strand breaks (DSBs) that are detrimental to the cell. The DNA DSBs are preferentially repaired by high fidelity HR repair that restore genomic integrity under normal circumstances. *BRCA1* and *BRCA2* genes encode proteins that are essential in HR repair. BRCA1/2 deficient tumour cells lack HR repair capacity and are therefore unable to repair these replication associated DSB DNA damage, which accumulates in the context of suppressed PARP activity, leading to chromosomal instability, cell cycle arrest and apoptosis The profound selective sensitivity of BRCA deficient tumour cells for PARPi compared with unaffected heterozygous mutant normal tissue retaining one functional *BRCA* allele provides a large therapeutic window (47,52,53) .

Preclinical models have demonstrated that HR deficient tumour cells with *BRCA1*-/- and BRCA2-/- genotype are exquisitely sensitive to PARP inhibition with olaparib (previously referred to as KU-0059436 or AZD2281 in earlier studies), in contrast to heterozygous mutant or wild-type cells (52). Likewise, in the clinic, olaparib is an efficacious therapy with durable antitumour activity and a favourable toxicity profile in HR deficient tumours arising in *BRCA1* and *BRCA2* mutation carriers including several patients with mCRPC (11).

Epigenetic Disruption of *BRCA 1/2* Function and Other Causes of HR Impairment

It has been hypothesised that targeting SSB DNA repair with PARPi will be a useful therapeutic strategy in sporadic tumours that harbour HR repair defects. This may occur due to either epigenetic or genetic silencing of *BRCA1/2* function or disruption of any of the other key proteins that make up the HR repair pathway. Loss of *BRCA 1/2* function may arise spontaneously via somatic mutations and epigenetic mechanisms during tumour formation. These somatic mechanisms of *BRCA1/2* functional loss are increasing implicated in sporadic carcinogenesis (54,55). Epigenetic gene silencing of tumour-suppressor genes is a well-recognised phenomenon with methylation of the *BRCA1* promoter found in 11-14% of sporadic breast cancers and 5-31% of ovarian cancers (56). Epigenetic modification of the Fanconi anaemia/*BRCA* pathway through promoter hypermethylation in lung, oral cancer and cervical cancer cell lines render these cells highly sensitive to DNA cross-linking agents (57,58). Somatic mutations of *BRCA* genes and heterozygous loss also appear to be more common that previously defined (54,58,59). The term “BRCAness” is commonly used to describe the phenotypic likeness of a sporadic tumour with a familial-*BRCA1/2* mutation related cancer (56). Identification of this BRCAness phenotype secondary to epigenetic / genetic silencing of the Fanconi anaemia/*BRCA* pathway may select for clinical benefit from PARPi in theses sporadic cancers.

In addition to somatic *BRCA1/2* dysfunction, disruption of any of the other critical proteins that make up the HR repair pathway such as RAD51, RAD54, DSS1, RPA1, NBS1, ATR, ATM, CHEK1, CHEK2, FANCD2, FANCA or FANCC 2 have been shown in preclinical models to cause HR deficiency and render these tumour cells highly susceptible to synthetic lethality with PARPi (56, 60, 61).

Clinical evidence supporting single agent olaparib antitumour activity in sporadic cancer was recently reported (58,62,63), with durable objective responses observed in 28% of sporadic high grade serous ovarian cancers in one study and a further study reporting a significant improvement in progression free survival (62,63). These responses may be explained in part by the high frequency of somatic mutations of *BRCA1/2* as well as epigenetic silencing of this and other key HR genes in high grade serous ovarian cancer (58). These results have led to the further evaluation of single agent olaparib in other tumour types enriched for HR defects. Identifying the subgroup of sporadic tumours with HR defects through validated predictive biomarker studies is imperative, if we are to successfully develop novel treatments such as PARP inhibitors (PARPi) and personalise medicine.

There has been limited investigation to date of the incidence of somatic aberrations in key HR related genes in advanced prostate cancer. High penetrance germline mutations in several candidate genes involved in DSB repair including *BRCA1*, *BRCA2*, *CHEK2*, *BRIPI/FANCJ* and *NBS1* are known to account for a small fraction of prostate cancer cases (64-70). *PTEN* however is one of the commonest tumours suppressor genes that is deregulated in prostate cancer and in implicated in the pathogenesis of this tumour as discussed above. Loss of *PTEN* function may also result in deficient HR DNA repair with reduced RAD51 expression and sensitivity to PARP inhibition (39,41). Likewise, the *ERG/TMPRSS2* gene rearrangement which is observed in 50% of prostate cancers is also implicated in conferring sensitivity to PARPi (44). Overall, these data support the evaluation of PARP inhibitors in the treatment of advanced prostate cancer.

2.3 **Rationale for Study**

Despite recent advances in CRPC treatment, metastatic prostate cancer remains a common disease with a poor outlook. Improved treatments that offer durable clinical benefit and significant survival impact remain an area of unmet medical need. As with most chemotherapies, a proportion of prostate cancers will not respond due to the underlying tumour biology and inherent resistance mechanisms. Understanding the underlying molecular determinants of response and resistance to different treatments is critical for improvement in CRPC patient outcome through optimally selected treatments.

The previously established antitumour activity of platinum based regimens in advanced prostate cancer suggests that there is a subgroup of CRPC susceptible to DNA damaging agents such as platinums and PARPi.

The PARP inhibitor, olaparib is a novel anticancer agent with strong preclinical and clinical data for significant antitumour activity in HR repair deficient tumours arising in *BRCA1* and *BRCA2* mutation carriers including a number of patients with metastatic CRPC (10,11,50,51,52,53). Recent clinical evidence supports the utility of this approach in

sporadic high-grade ovarian cancers enriched for HR defects (62,63). Moreover, there is a growing body of preclinical evidence that supports extending this approach to a wide range of other sporadic tumours with disruption to other non-redundant components of the HR repair pathway including RAD51 and other essential HR repair proteins (60,61). Identifying these sporadic tumours with HR repair defects through validated predictive biomarkers remains a challenge and focus of ongoing research.

PTEN loss has been implicated to confer HR DNA repair defects with reduced RAD51 expression, increased genomic instability and tumour sensitisation to PARP inhibition (41-43). Homozygous *PTEN* loss by FISH is present in 20-30% of CRPC, with loss of protein expression by IHC being far more common (21). In addition, the *TMPRSS2/ERG* driver rearrangement, which is present in approximately 50-60% of prostate cancers and co-associates with *PTEN* loss has also been reported to confer sensitivity to PARP inhibition (44,45).

The target population for this trial are patients with advanced metastatic CRPC progressing following docetaxel treatment. A proportion of patients may also have received treatment with abiraterone and cabazitaxel. Given the high prevalence of loss of *PTEN* function and *TMPRSS2/ERG* rearrangements, as well as the frequency of DNA repair genes mutations and deletions in this population and the strong preclinical rationale for this therapeutic strategy, we anticipate that a subgroup of these patients will benefit from olaparib treatment.

Patients may continue olaparib treatment until disease progression or treatment intolerance. Patients may however withdraw from the study at any point if they choose to do so or if the investigator believes it is in the best interest of the patient. Additionally, in the event of unmanageable toxicity, directions for reducing the dose of olaparib are provided.

Rationale for Circulating Tumour Cell Based Studies in CRPC

Enumeration of circulating tumour cells (CTC) number using the Cell Search system (Veridex, PA) is a valid biomarker to monitor the effectiveness of therapy of patients with metastatic breast cancer (71-73), colorectal cancer (74) and prostate cancer (75-78). High baseline counts have consistently been associated with more aggressive disease and a worse outcome (76-78). CTC counts are highly prognostic for overall survival, and CTC count falls post-treatment associate with an improved overall survival and response to therapy in CRPC. In a pilot study, univariate analysis demonstrated that CTC counts of < 5/7.5 ml blood or ≥ 5 /7.5 ml blood correlated with OS (0.002; HR=7.37); in multivariate analyses CTC counts and PSA doubling time were the only 2 independent predictors of outcome unlike PSA, Gleason score, presence and absence of bony metastasis and age (75). In a similar study that related changes in CTC counts in patients with CRPC during the first 2 cycles of treatment with clinical outcome, we observed that patients with CTC counts that remained below 5 cells/7.5 ml blood during treatment with cytotoxic therapy had the best outcomes, whilst patients whose counts changed from ≥ 5/7.5 ml blood to ≤ 5 had improved outcomes compared to the patient cohort whose CTC count remained persistently elevated at ≥ 5/7.5 ml blood throughout treatment. In addition, patients whose CTC counts increased during treatment form < 5 to ≥ 5/7.5 ml blood had a similar poor outcome to the group whose count remained persistently elevated at ≥ 5 CTCs throughout treatment

suggesting the development of treatment resistance (76). CTC counts were commonly detected in patients with advanced CRPC; CTC counts >5/ 7.5ml were detected in 19/49 (39%) and 21/28 (75%) of chemotherapy –naïve and post–docetaxel patients respectively treated on the phase I-II Abiraterone studies (35). We now have data from almost a thousand patients participating in the randomised Phase III abiraterone trial (COU-301) that CTC conversion from ≥5 to <5 as well as any fall in CTC from 10% to 90% count post-treatment at 1, 2, 3 months after starting abiraterone is a statistically robust surrogate/intermediate endpoint of overall survival, meeting all of Prentice's criteria for surrogacy (78)

In addition, CTCs have been shown to retain many of the genomic characteristics of the primary tumour and thus provide a useful opportunity to study molecular changes in the disease profile longitudinally with ongoing treatment. Multicoloured FISH assays have been successfully applied to determine commonly found genomic alterations such as AR amplification, *PTEN* deletions and *TMPRSS2/ERG* fusion rearrangements in prostate CTCs (35,79,80). We have also developed multiplex immunoflourescence assays to evaluate protein expression in CTCs.

Rationale for Radiographic PFS and 6 months PFS as an Endpoint

Clinical research in prostate cancer has been hindered by the difficulty in objectively defining cancer progression. Composite endpoints that include patient reported, radiographic, and skeletal-related events have suffered from subjectivity. Conventional RECIST alone has limitations in CRPC in defining radiographic progression due to the fact that fewer than 50% of men with CRPC have measurable lesions greater than 2cm in size, and most of these lesions are in lymph nodes which impact survival less than visceral metastases (81). Conventional RECIST also lacks the sensitivity to differentiate true progression of bone metastasis from the flare phenomenon on bone scan or CT scan due to healing of previous bone metastasis (82,83). Although radiographic PFS is not a surrogate for overall survival in patients receiving docetaxel therapy, it outperforms PSA based progression (83).

Attribution of disease progression due to tumour flare phenomenon will be mitigated in this study by applying the new consensus guidelines that requires a confirmatory bone scan at least six weeks after the original scan to define progression. This will reduce the number of false positive determinations of CRPC progression. (Refer to Appendix D)

Progression of metastatic bone disease is of paramount importance to CRPC patients since it is responsible for severe morbidity of skeletal related events such as fracture, pain, and spinal cord compression. Similarly, progression of soft tissue metastasis may be associated with clinically significant morbidity. Prevention or delay in cancer progression should result in clear clinical benefit to the patient. In conclusion, radiologic PFS (rPFS) is an objective measurement that may associate with clinical benefit in the proposed study population.

Pain and Analgesic Assessments

Pain due to bone metastases and nerve root compression is a common experience for patients with advanced prostate cancer. Pain has previously been shown to significantly

associate with overall survival in advanced prostate cancer. The McGill Pain Questionnaire (MPQ) (PPI score 0-2 versus 3-5) successfully stratified the survival of first line chemotherapy patients in TAX 327 (84,85).

The BPI-SF, a numeric rating scale (NRS), has been selected as the pain instrument for this study due to the high correlation of numeric rating scales with verbal descriptor scales (e.g. MPQ PPI) (86) and its prior use in other metastatic prostate cancer studies (87,88). The BPI-SF assesses both the intensity of pain and the interference in the patient's function caused by their pain (89). The BPI-SF data can potentially help illustrate the overall benefit of treatment on pain. Analgesic usage will be scored according to the WHO analgesic ladder: 0 for no analgesic, 1 for non-opioid analgesics (including NSAIDs, paracetamol / acetaminophen, antidepressants, and agents intended to treat neuropathic pain), 2 for opioids for moderate pain, and 3 for opioids for severe pain (90).

3 BACKGROUND THERAPEUTIC INFORMATION

Olaparib

Olaparib previously referred to as AZD2281 or KU-0059436 in earlier studies is a potent inhibitor of PARP-1. Olaparib shows significant monotherapy activity in tumour cells with defective components of HR, such as cells with the *BRCA1 -/-* and *BRCA2 -/-* genotype both preclinically and clinically (10,11,50,51,52,53,62,63). Selective tumour sensitivity to this PARP inhibitor has also been observed in tumour cells with loss of PTEN function and *ETS* gene rearrangements (41,42,44). This sensitivity in pre-clinical models is expected to translate to clinical benefit. Olaparib (Lynparza™) was granted FDA and EMA approval (December 2014) as a treatment for women with advanced ovarian cancer associated with defective BRCA genes.

3.1 Pre-clinical Experience with Olaparib

This pre-clinical and clinical experience with olaparib (AZ2281, KU0059436) is fully described in the Investigator Brochure (IB). Key findings are summarised below.

Pharmacokinetic Data

The olaparib molecule shows cellular activity in the low nM range with a cellular dose for 50% inhibition (IC50) of 2 nM in HeLa cells. Distribution of olaparib is typical for an orally administered foreign compound, in the gastro-intestinal tract and in tissues associated with the metabolism and elimination of foreign compounds. The oral bioavailability of olaparib when dosed at 5mg/kg via a solution formulation was 17% and 19% in male and female rats and 79% -/+ 12 in male dogs. An oral study of olaparib demonstrated bioavailability of >85% for both 5% and 20% w/w microionised dispersion of the drug substance in Gelucire. Following single oral dosing (5mg/kg), absorption was rapid with an AUC of 10.3+/- 2.01 in dogs. The estimated t1/2 for olaparib was 3.0, 2.5 and 4.61+/-1.66 hours in female rats, male rats and dogs respectively. Multiple oral dosing of olaparib in 28-day rat and dog studies demonstrated that absorption was rapid and exposure increased greater then proportionally with dose across the dose range examined (upto 15mg/kg/ day in dogs and upto 40mg/kg in rats). Plasma protein binding of olaparib varied between 70.4% and 58.8% in rat and dog studies. Metabolism data to date is limited and further investigations are ongoing. To date, several metabolites have been observed in pre-clinical studies. Similar metabolite profiles were observed in the urine and faeces of male and female rats. Excretion is primarily via the faeces and to a lesser extent, the urine. In a study of [14C]-olaparib in the rat, excretion was 76±13% in faeces and 20±11% in urine. For more detailed preclinical data please refer to the IB.

Toxicology and Safety Data

Olaparib has shown comparatively low toxicity in rodent and dog toxicology studies. Oral doses of olaparib were well tolerated for up to 6 months in safety studies and showed no cardiovascular, respiratory, central, peripheral and autonomic nervous system toxicity

findings that were a concern. The toxicology studies indicate that the target organ of toxicity is the bone marrow. Specific *ex vivo* work has been conducted exposing human bone marrow cells to olaparib, which has confirmed that olaparib is also active against human bone marrow. The cytotoxic effect becomes evident at a higher concentration than that which fully ablates PARP activity (mean IC50 of 2.7 µM for myelosuppression [n = 4 human donors] compared with 0.1 µM for total PARP-1 activity inhibition). These data, along with the 28-day dog and rat studies, show a myelotoxic effect that is mild-to-moderate and is reversible. Platelets appear first affected, followed by white blood cells. Importantly, oncology clinics are used to monitoring for the onset of such effects and are expert in their management.

Olaparib showed no mutagenic potential in the Ames test. Olaparib was clastogenic in the Chinese hamster ovary (CHO) chromosome aberration test, and was genotoxic in the rat micronucleus test. Reproductive toxicology data indicate that olaparib can have adverse effects on embryofoetal survival and development at dose levels that do not induce maternal toxicity. These findings are not uncommon for many therapeutic agents used in oncology, and so do not present an unacceptable risk when appropriately clinically managed. For further details on these tests or on the fertility studies please refer to the IB.

3.2 Clinical Experience with Olaparib

3.2.1 Pharmacokinetic (PK), Pharmacodynamic (PD) Data

In the Phase I study, patients with solid tumours were given olaparib at a starting does of 10mg for the first 14 days of a 21-day cycle with a subsequent protocol amendment, changing the dose schedule to continuous daily dosing starting at 100mg BID for a 28-day cycle. Absorption was rapid and a peak plasma concentration was observed between 1-3 hours post dosing with a terminal elimination half-life of 6.10 hours, supporting twice daily dosing. Drug exposure increased dose proportionally with doses up to 100 mg but increase less than dose proportionally thereafter. The mean volume of distribution and mean plasma clearance are approximately 40.3 L and 4.55 L/h. On multiple dosing, there was no evidence of any time dependency of the PK and no marked accumulation. Exposure increased proportionally with dose at doses up to 100 mg twice daily but increased in a less than proportional fashion at higher doses. There was no evidence of any marked ethnic difference in PK of olaparib between Japanese and Caucasian patients. Following dosing to cancer patients at doses of 400 mg BID, the population estimated maximum plasma concentration at steady state (Cmax ss) ranged from 1.45 µg/mL to 11.0 µg/mL (3.34 µM to 25.3 µM, equivalent to unbound concentrations of 0.26 µg/mL to 1.99 µg/mL [0.6 µM to 4.58 µM]); the steady state area under the plasma concentration-time curve (AUC) (area under plasma concentration-time curve from zero to 12 h [AUC0-12]) ranged from 6.56 ug.h/mL to 122 ug.h/mL.

The distribution of olaparib is typical for an orally administered compound, in the gastro-intestinal tract and in tissues associated with the metabolism and elimination of foreign compounds. Following administration of a radiolabelled dose of olaparib to cancer patients, olaparib accounted for approximately 70% of the circulating material in the plasma with the remainder accounted for by 3 other components (each accounting for approximately 10% of

the material), all of which were also present in the excreta. Drug-related material was eliminated in the urine (35% to 50%) and in the faeces (12% to 60%) with 10% to 20% and 0.6% to 14% of the dosed material recovered in the urine and faeces as unchanged drug, respectively. A further 35 and 19 drug related components were excreted in the urine and faeces, respectively. The metabolites produced were predominantly a consequence of hydroxylation, oxidation or dehydrogenation of the piperazine carboxycyclopropyl, the flourophenyl and the phthalazinone ring system.

Evidence of inhibition of PARP-1 activity has been seen in peripheral blood mononuclear cells (PBMC) and tumour samples from patients dosed with olaparib at all dose levels studied (10 mg to 600 mg) but the data shows wide inter-individual variability in the extent of inhibition achieved and no evidence of any relationship between extent of inhibition and dose administered. Concentration-response curves for inhibition of PARP-1 activity in both PBMC and tumour samples have been generated from a population PK-PD analysis of the data. The population average maximal extent of inhibition in PBMC and tumour samples were 50.6% and 70%, respectively, with maximal inhibition achieved at plasma exposures (steady state AUC) in excess of 1µg.h/mL (exposures which would be expected to be reliably achieved following doses of >40 mg). The potential clinical benefit of continuous dosing is supported by preclinical studies, which show that tumours re-grow after short-term use (e.g. 14 days) and that continuous PARP inhibition with olaparib was able to prevent tumour re-growth. The maximum tolerated dose of olaparib established in the Phase I trials was 400 mg twice daily, continuously (10).

3.2.2 Efficacy Data

The phase I study was enriched with *BRCA1* and *BRCA2* mutation carriers with advanced tumours in the escalation and expansion phase given the strong preclinical rationale of selective tumour killing in BRCA1/2-deficient cell lines. In the Phase I study, 41% of 46 evaluable patients with *BRCA*-mutant ovarian cancers achieved objective responses (complete or partial responses) either by Gynecologic Cancer Intergroup (GCIG) CA125 or RECIST criteria and a further 11% of patient's attained RECIST disease stabilisation (10,11). The encouraging clinical benefit rate of >50% in the subpopulation of *BRCA1/2* mutation cancer patients provides compelling evidence of single agent PARP inhibitor activity in BRCA-deficient cancer patients. Durable antitumour responses have also been reported in *BRCA* mutated breast and prostate cancer patients with one prostate patient ongoing treatment for more than 2 years (10, 11, 50, 51).

Two parallel open-labelled multicentre phase II studies of olaparib in *BRCA1/2* mutation carriers with advanced breast and ovarian cancer have confirmed the significant therapeutic efficacy of PARPi in this population (50, 51). 54 breast cancer patients and 56 ovarian cancer patients with advanced heavily pre-treated disease were recruited to 2 non-randomised sequential dose cohorts of 400mg and 100mg BID of olaparib. Following efficacy assessment of the 2 dose levels; patients on the 100mg BID cohort in both studies were allowed to increase the dose of olaparib to 400mg BID given the improved antitumour activity seen at the higher dose level. The breast cancer study reported an objective response rate (ORR) of 41 % (11/27) with 1 complete response (CR) and 10 partial response (PR)s and a median progression free survival (PFS) of 5.7 months (m) (range

4.6-7.4m) at 400mg BID in contrast to an ORR of 22% (6/27) with 6 PRs and a median PFS of 3.8m (range 1.9-5.5m) at the lower dose level (27). In the ovarian study, patients on the 400mg BID and 100mg BID dose levels showed an ORR of 33% (11/33) versus 13% (3/24) by RECIST criteria, a response by either RECIST or by GCIG criteria of 61% (20/33) versus 17% (4/24) and a median duration of response of 290 days (range: 126-513 days) versus 269 days (range:169-288 days) respectively (50). Both dose levels demonstrated clinical activity, however the 400mg BID was more efficacious with improved outcomes establishing substantial antitumour activity for olaparib at a dose of 400mg BID in heavily pre-treated, *BRCA* mutant advanced breast and ovarian cancer patients (50,51). A further phase II study of olaparib at 400mg BID in patients with sporadic high grade ovarian cancer showed an objective response rate of 28%. This study provided the first clinical evidence for the antitumour activity of olaparib in sporadic serous ovarian cancers (62). Most recently, patients with sporadic platinum sensitive serous ovarian cancer who previously achieved either a partial or complete response from platinum therapy demonstrated a doubling of the progression free survival (PFS) from 4.8months to 8.4months with 400mg BID olaparib maintenance treatment compared to placebo. The hazard ratio (HR) was 0.35 (95% CI: 0.25, 0.49; p<0.00001) (63).

3.3 Summary of Clinical Toxicity Data

The clinical experience with olaparib is fully described in the IB and largely has been conducted with the Gelucire® 44/14 (capsule) formulation.

As of 02 October 2013 , approximately 2103 patients suffering from ovarian, breast and a variety of other advanced solid tumours had been exposed to olaparib across the dose range 10 mg daily to 600 mg BID, either as monotherapy (n=18 studies, estimated 1214 patients) or in combination with other chemotherapy/anti-cancer agents (liposomal doxorubicin, cisplatin, dacarbazine, gemcitabine, gemcitabine+cisplatin, carboplatin, carboplatin+paclitaxel, paclitaxel, topotecan, irinotecan, or bevacizumab).

Olaparib (in the capsule formation) appears to be generally well tolerated in patients with various solid tumours at doses up to and including 400mg bd, as monotherapy. Administration of olaparib has been associated with reports of laboratory findings and/or clinical diagnoses of:

- Haematological toxicity:
    - Anaemia, generally mild to moderate (CTCAE grade 1 or 2)
    - Neutropenia, predominantly mild to moderate (CTCAE grade 1 or 2)
    - Lymphopenia, generally mild or moderate (CTCAE Grade 1 or 2)
    - Thrombocytopenia, generally mild or moderate (CTCAE grade 1 or 2), sometimes severe (CTCAE grade 3 or 4)

    - Mean corpuscular volume or elevation, generally mild to moderate (CTCAE grade 1 or 2)
    - Increase in blood creatinine, generally mild or moderate intensity (CTCAE Grade 1 or 2)

- Nausea and vomiting, generally mild to moderate (CTCAE grade 1 or 2), intermittent and manageable on continued treatment

Decreased appetite, generally mild or moderate intensity (CTCAE Grade 1 or 2)

- Diarrhoea, generally mild or moderate intensity (CTCAE Grade 1 or 2)

- Dyspepsia, generally mild or moderate intensity (CTCAE grade 1 or 2)

- Stomatitis, generally mild or moderate intensity (CTCAE Grade 1 or 2)

- Dysguesia, generally mild or moderate intensity (CTCAE grade 1 or 2)

- Fatigue, (including asthenia) generally mild or moderate intensity (CTCAE grade 1 or 2)

- Headache, generally mild to moderate intensity (CTCAE grade 1 or 2)

- Dizziness, generally mild to moderate intensity (CTCAE grade 1 or 2)

- Pneumonitis events with no consistent clinical pattern have been reported in a small number of patients with extensive previous exposure to chemotherapy

- Myelodysplastic syndrome (MDS) and Acute Myeloid Leukemia (AML) have been reported very rarely in a small number of patients

From the completed studies, there are no safety concerns noted for the renal or hepatic parameters measured and no clinically significant changes of concern relating to coagulation parameters. The changes in haematological parameters (haemoglobin, neutrophils, and thrombocytes) are as expected for olaparib monotherapy or can be explained by co-existing conditions/previous chemotherapy.

Olaparib appears to be generally well tolerated at PARP inhibitory dose levels in the monotherapy clinical studies with predominantly mild toxicity. Thus it represents a potential advance in the treatment of advanced cancers by directly inhibiting PARP-1 and hence tumour growth as monotherapy.

Preliminary data from Phase I and Phase II studies of olaparib in combination with various chemotherapy agents indicate a substantial increase in neutropenia, thrombocytopenia and anaemia compared to giving these cytotoxic agents alone. These findings are consistent with pre-clinical findings. There are more severe haematological events i.e., CTCAE grade 4 neutropenia, febrile neutropenia and thrombocytopenia being reported as SAEs. When these toxicities occur it has been managed with routine clinical practise, including dose

delays, dose reductions and intermittent doses and/or the use of supportive care measures such as G-CSF.

Final laboratory data from D0810C00002 (KU36-92) study have shown mean corpuscular volume (MCV) increases in approximately 30% of patients in the study, especially with prolonged treatment with olaparib (≥6 cycles of treatment). The MCV elevations were mild to moderate in intensity, observed in the 200 mg BID dose cohort and above and were asymptomatic in nature. There was no obvious correlation of MCV with haemoglobin, platelets or white blood cell (WBC) values for these patients, and there does not appear to be any clinical consequences. MCV has not been captured as a standard laboratory parameter within the olaparib Phase II studies D0810C00008 (KU36-44) and D0810C00009 (KU36-58), however elevations in MCV were also observed in a number of patients from these studies. The MCV elevations again appear to become progressively elevated over time. Elevations in serum amylase were observed in approximately 15% of patients from final laboratory study data from D0810C00002 (KU36-92). The majority of these increases (13%) were mild to moderate (CTCAE grade 1 or 2) with no apparent link to clinical symptoms. No new significant safety information has emerged from any other reported studies with respect to elevations in serum amylase.

Rare Adverse Events

Myelodysplastic syndrome (MDS) and Acute Myeloid Leukemia (AML)

As of 02 October 2013 a total of 16 patients who received olaparib have had MDS and/or AML from 5 monotherapy studies (D0810C00002, D0810C00009, D0810C00012, D0810C00019 and D0810C00042) and 4 combination studies (D0810C00004 and D0810C00041) out of a total number of 2103 patients estimated to have received olaparib in the development programme to date (0.76%, 16/2103). Additionally, there have been 2 reports of MDS from 2 monotherapy studies (D0810C00012 [completed], D0810C00019 [ongoing: completed its primary analysis of PFS]) out of 304 patients that received placebo or comparator in the trial programme (0.66%): an AE of MDS was reported in a patient who received placebo in Study D0810C00019 (post PFS data cut-off); and MDS was reported as a secondary cause of death in a patient who received liposomal doxorubicin in Study D0810C00012.

The 13 olaparib-treated patients who reported MDS and/or AML received between 126 days and 1759 days of olaparib for ovarian/peritoneal cancer, prior to the diagnosis of MDS and/or AML. The MDS/AML events occurred post-discontinuation of olaparib treatment in 8 of the 13 patients and 8 patients died. All 13 patients had other potential contributory factors that may offer an alternative explanation for the development of MDS/AML. Ten patients had a documented BRCA mutation that may predispose them to developing MDS and/or AML (Cole and Strair 2010). The ovarian/peritoneal cancers were diagnosed around 497 days to 4503 days prior to starting olaparib treatment. The majority of patients had been treated with extensive previous chemotherapy including carboplatin, taxanes, anthracyclines and other alkylating and DNA damaging agents, (ranging from 6 to 95 cycles over periods of 6 months to 15 years). One patient had a family history of leukaemia.
Epidemiological studies from the literature have indicated a higher risk of therapy related MDS/AML in ovarian cancer populations, particularly those who received alkylating agents

(Leone et al 1999, Travis et al 1999). Pedersen-Bjergaard et al (Pedersen-Bjergaard et al 1980) reported on the cumulative probability of developing AML over time, from 0 to 5 years of observation, in first line patients with ovarian carcinoma exposed to chemotherapy with alkylating agent Theosulfan. At 2 years the risk was 0.4% (±0.4 standard error [SE]), at 3 years was 1.0% (±0.7 SE) and 4 years 2% (±1.2 SE). This illustrates the increasing risk of developing AML over time when exposed to chemotherapy. The cumulative incidence for developing MDS/AML post exposure to olaparib is currently 13 cases or 0.7% of the total exposed population, where average time from diagnosis of ovarian/peritoneal cancer to onset of the event is 5.9 years (range 2.6 to 12.7). The number of reports falls within those expected using the Pedersen-Bjergaard estimates of patients exposed to chemotherapy.

Pneumonitis

As of 02 October 2013, approximately 2103 patients are estimated to have been exposed to olaparib. There have been 10 (0.5%) reported cases of pneumonitis or suspected pneumonitis.. Three were from monotherapy studies and 7 were from combination studies (3 with olaparib in combination with liposomal doxorubicin, and 1 each with olaparib + topotecan, olaparib + paclitaxel, olaparib + gemcitabine + cisplatin and olaparib + gemcitabine). These patients had a variety of cancer types including breast cancer, nonsmall cell lung cancer, small cell lung cancer and cancer of the thymus. All but one of these patients were hospitalised for the management of pneumonitis. Five of these patients have died; 2 from pulmonary insufficiency and 3 for disease progression. In summary, the 10 reported cases of pneumonitis presented with no consistent clinical pattern and were heavily confounded by a number of predisposing factors (including disease under investigation, underlying pulmonary disease, pre-existing medical conditions, smoking history and/or previous chemotherapy and radiotherapy). Additionally there have been 3 reports of pneumonitis out of 304 patients that received placebo or comparator in the trial programme (0.99%). The frequency of pneumonitis seen with olaparib treatment is consistent with the expected rates of pneumonitis in a cancer population (93-94).

Pre-clinical and Clinical Experience with New Melt-Extrusion (tablet) formulation

Melt-Extrusion (tablet) formulation has been developed to render the olaparib amorphous and presents the drug in a solid dispersion in copovidone (30% drug loading). The milled extrudate is then blended with pharmacopoeial excipients and compressed into a tablet.

The Melt-Extrusion (tablet) formulation, when dosed to fasted dogs at a 100 mg dose, shows at least 2.4 times the Cmax & AUC than the Gelucire® 44/14 (capsule) formulation. In addition the Melt-Extrusion (tablet) formulation showed reduced pharmacokinetic variability in the dog with CV of ~ 20% as compared to ~ 50% for the Gelucire® 44/14 (capsule) formulation. It is envisioned that the new Melt-Extrusion formulation of olaparib should provide a more bioavailable and patient friendly formulation by minimising the number of dosage units and providing a more consistent PK profile.

This olaparib Melt-Extrusion formulation (tablet) will be the formulation evaluated in the Phase III clinical programme and consequently this study will also be conducted with the olaparib tablet. There is an ongoing trial (D0810C00024) being conducted at the Royal Marsden NHS Foundation Trust and other centres to establish the comparative

bioavailability of the tablet formulation compared with the capsule formulation. In addition single and multiple dose safety & tolerability data will be gathered.

Following single oral dosing with the tablet formulation, absorption was more rapid that the capsule. The $C_{max}$was typically achieved between 0.5 hours and 2 hours after dosing. Following the peak, plasma concentrations declined biphasically with the mean terminal $t_{1/2}$ of 6.97 hours. The mean volume of distribution of olaparib tablet was 54.9L and the mean plasma clearance was 542L/h. Both G_{mean} ,C_{max} and AUC increased approximately proportionally with dose (8-fold and 12-fold, respectively, for a 10-fold increase in dose).

The relative bioavailability (BA) of the tablet formulation (compared to capsule) at the 3 lower dose levels studied are shown in Table 1. At the 2 lower tablet doses, although the C_{max} achieved after dosing with the tablet formulation tended to be higher than that with the capsule, when based on AUC, the BA of the 2 formulations was actually very similar. However, at the highest tablet dose (250 mg), the exposure delivered by the tablet formulation (both C_{max} and AUC) was higher than that delivered by the capsule.

Table 1: Relative bioavailability of tablet versus capsule formulation

Parameter	25mg tablet dose vs 50mg capsule dose	50mg tablet dose vs 100mg capsule dose	250mg tablet dose vs 400mg capsule dose
C_{max} ratio	1.29	1.53	2.49
95% CI	0.92-1.81	1.17-2.00	1.93-3.20
AUC ratio	1.03	0.99	1.74
95% CI	0.83-1.27	0.71-1.38	1.27-2.38

AUC, Area under the plasma concentration-time curve; CI, Confidence interval; $C_{max,}$Concentration maximum(plasma).

On multiple dosing, a 200mg tablet BID dose gave plasma exposure within the range of those achieved following 400mg BID capsule dose (6.88ug/ml vs 5.71 ug/ml) but the average AUC_{0-12} and C_{min} were lower (36.1ug/ml vs 43.1 ug/mL and 1.00ug/mL vs 1.86ug/mL, respectively) than achieved in a separate cohort of patients following the 400mg BID capsule dose. In addition, steady state data generated from a further cohort of patients randomised to BID dosing with both 200mg tablets and 400mg capsule dose (n=6) demonstrated that the 2 formulations could not be concluded to be bioequivalent (treatment ratio for C_{max} was 0.99 (90% CI:0.85 to 1.15) but for AUC0-12 was 0.79 (90% CI:0.65 to 0.94). Following multiple doses at 250, 300, 350 and 400mg BID, exposure continued to increase with dose. Please refer to the IB for further data on the tablet formulation.

A Phase I bioavailability trial (D0810C00024) has been completed and has shown 400 mg BID to be the maximum tolerated tablet dose with frequent dose limiting toxicities including gastrointestinal toxicities being observed at higher doses in ovarian cancer patients although it was not clear if this was partly related to disease. The study suggested that 300mg and 400mg may be similar in terms of inducing tumour shrinkage in this small patient cohort with ovarian and breast cancer, with a better tolerability profile for the 300mg BID dose level. Therefore, 300mg BID of the tablet formulation was selected as the dose to be tested in randomised phase III trials in ovarian cancer (97). The TOPARP trial was

launched using 400mg BID of the tablet formulation and has been generally very well tolerated in this prostate cancer population, however,, based on the emergence of data from study D0810C00024, Part B of the trial (TOPARP-B) will include two parallel open-label cohorts to treat patients with 400mg and 300mg BID of Olaparib respectively to compare antitumour activity and tolerability. It is envisioned that a small proportion of patients may require dose reductions to 300mg BID, 250mg BID, 200mg BID for cumulative toxicity over the course of several months of treatment but that the higher dose may have superior efficacy.

4 TRIAL OBJECTIVES

4.1 Primary Objective

The primary objective of this study is to evaluate the anti-tumour activity of olaparib in patients with advanced CRPC who have progressed following one or two chemotherapy regimens including taxane-based regimens.

4.2 Secondary Objectives

The secondary objectives are to evaluate:

- Radiological progression-free survival (rPFS)
- Time to radiological progression
- Progression free survival (PFS)
- Overall survival
- Time to PSA progression
- Duration of PSA response (decline of ≥50%)
- Percentage of change in PSA from baseline to 12 weeks (or earlier if discontinued therapy) and maximum PSA decline while on treatment.
- The utility of CTCs as an early indication of benefit from PARP inhibitor therapy
- Further characterise the safety profile of olaparib in advanced CRPC patients
- TOPARP-B only - drug exposure (pharmacokinetics) with the two dose levels of 300mg or 400mg of the tablet formulation and correlation of drug exposure with safety events and antitumour activity.
- TOPARP-B only – evaluation of the two dose levels and recommendation of dose to investigate further.

4.3 Tertiary/exploratory Objectives

- To evaluate clinical benefit as indicated by an improvement in pain measurements (BPI-SF).
- To evaluate DW-MRI imaging in selected patients and to correlate these results with PSA, CTC and radiological responses by conventional imaging.
- TOPARP-B only – to evaluate the response rate following dose-escalation from 300mg to 400mg twice daily.

5 TRIAL ENDPOINTS

5.1 Primary Endpoints

Response rate:

Response will be defined on the basis of the following outcomes; if any of these occur patients will be considered to have responded;

- Objective response by modified RECIST1.1 (Appendix E) or
- Conversion of circulating tumour cell count (CTC) from ≥5/7.5 ml blood at baseline to <5/7.5 ml blood nadir confirmed by a second consecutive value obtained four or more weeks later
- PSA decline of ≥50% (according to the PCWG2) (Appendix D)

5.2 Secondary Endpoints

- Radiological progression-free survival (rPFS)
- Time to radiological progression
- Progression free survival (PFS)
- Overall survival
- Time to PSA progression
- Duration of PSA response (decline of ≥50%)
- Percentage of change in PSA from baseline to 12 weeks (or earlier if discontinued therapy) and maximum PSA decline while on treatment
- Proportion of patients with conversion of CTC count to <5/7.5ml blood nadir
- Safety and tolerability profile of olaparib in patients with metastatic CRPC
- TOPARP-B only - Cmax and AUC after first dose and at steady state for the 400mg and 300mg BID dose levels.

5.3 Tertiary/exploratory Endpoints

- Impact of olaparib treatment on bone metastases imaged by DW-MRI in selected patients (optional) and to correlate these results with PSA, CTC and radiological responses by conventional imaging
- Proportion of patients achieving pain improvement
- TOPARP-B only - response rate following dose-escalation from 300mg to 400mg twice daily.

6 TRIAL DESIGN

This study is divided into 2 parts; TOPARP-A "Part A" and TOPARP-B "Part B". Part A is an efficacy screening stage to identify predictive biomarkers. It is an open-labelled, non-randomised trial with a two stage design which allows the study of the activity of olaparib in unselected advanced CRPC patients to be evaluated and permits development of predictive biomarkers of response to olaparib. Part B is a subsequent validation stage which will be undertaken if confirmation of activity in a biomarker defined subgroup is necessary. It is an open-labelled, randomised single-stage two-group assessment of anti-tumour activity of two doses of olaparib in preselected patients based on putative biomarkers of HR repair deficiency previously identified in Part A. A total of up to 133 evaluable patients will be enrolled to Parts A & B.

Patients with advanced CRPC who have consented to participate in this trial will be screened and enrolled into either Part A or Part B of the study depending on which part of the study is running at the time of patient enrolment.

In Part A, patients will be administered oral olaparib tablets, 400mg BID continuously until disease progression is documented and confirmed (i.e., confirmed radiographic or clinical progression) or withdrawal for another reason (see section 10.10). In Part B, patients will be randomly allocated to be administered oral olaparib tablets at dose of 300mg BID or 400mg BID continuously until disease progression is documented and confirmed.

Paraffin embedded tumour blocks or sections from the archival tumour tissue at the time of the original diagnosis or from a biopsy / resection of metastatic disease is mandated from all subjects who enrol on the study. In Part A these samples should be obtained during the screening period after written informed consent has been obtained. In Part B these samples should be obtained during the **pre-screening** period after written informed consent has been obtained to test for the presence of the biomarker (eligibility criteria for Part B).

All patients participating in this trial (Part A & B) will also have mandatory circulating tumour cells (CTCs), plasma and fresh tumour biopsies collected at pre-specified times during the study (Table 3b, fresh tumour biopsies are optional in part B). The tumour material will be analysed for biomarkers of sensitivity (response) to PARP inhibition. These tests will include *PTEN* and *ERG* expression, *ETS* gene rearrangements and *PTEN* deletions, markers of cell cycle, DNA damage and repair including geminin expression, RAD51 and gamma H2AX foci formation, etc. Other biomarkers identified to be relevant to HR pathway from other translational research may be studied in parallel. Response to treatment with olaparib will be correlated to all the biomarkers evaluated.

In Part A, accrual will be in unselected patients. The tumour tissue in responders versus non-responders from Part A will be evaluated to develop a molecular classifier for response to olaparib in CRPC patients that can then be utilised to select patients in Part B in the absence of evidence of a high overall response rate in Part A. Patients registered for Part B will be made aware that their tumour tissue will first be analysed for the relevant predefined biomarkers to determine their suitability for participation. Following confirmation of the predefined biomarkers patients will be consented into Part B of the study.

Disease status will be assessed at regular intervals during the course of the trial by CTC enumeration, computed tomography (CT) scan and / or on magnetic resonance imaging (MRI), radionuclide bone scans and PSA. Each site should ideally designate the same reader who will evaluate the images for any one patient for the duration of the trial. Throughout the study, safety and tolerability will be assessed by the recording of adverse events; the monitoring of vital signs and physical examinations; and safety laboratory evaluations. Local laboratories will be used for the safety bloods. An independent Data Monitoring Committee (IDMC) will monitor safety data on an ongoing basis. Patients may continue on olaparib for as long as they remain free of intolerable toxicity and are, in the opinion of the investigator, deriving clinical benefit. Patients may however elect to withdraw from the study at any point without prejudice to further treatment. Patients will have a safety follow-up visit 30 days +/-5 after their last dose of study drug for safety and end of treatment assessments.

Although outlined in the trial schema below, Part C is not currently included in this protocol but may be incorporated following Parts A and B as an amendment to this protocol. Part C will be the subsequent randomised evaluation of olaparib. Part C will only be initiated if there is sufficient evidence of antitumour activity observed either in the unselected or selected CRPC patients to warrant further evaluation of olaparib in a randomised setting. This will occur following evaluation of safety, efficacy and biomarker analysis by the Independent data Monitoring Committee (IDMC) and will involve a separate funding application and regulatory approvals. Patients in Part C may be either preselected patients based on the molecular determinants of response as identified in Parts A and B, or be unselected, depending on the response that has been seen in Part A.

More details on the design of the trial can be found in the statistical considerations section.

6.1 Study Flow Chart

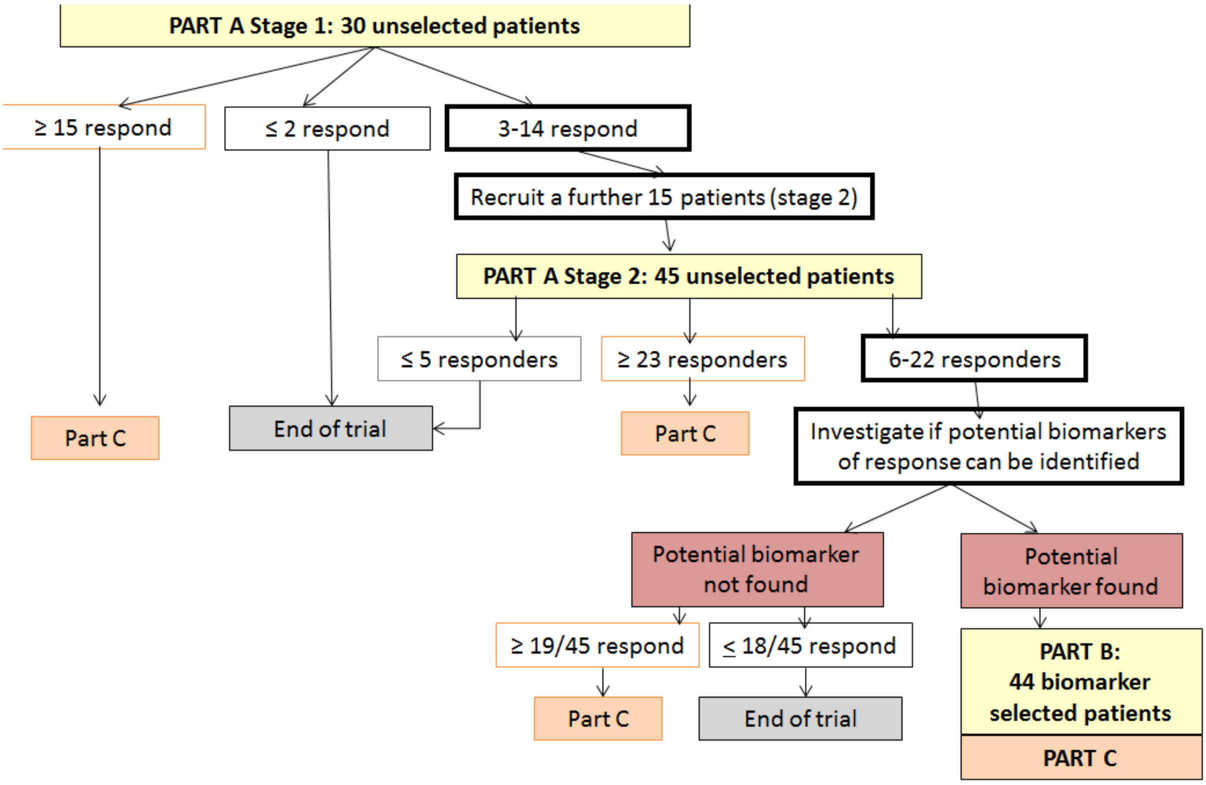

Part B: validation of the biomarker suite

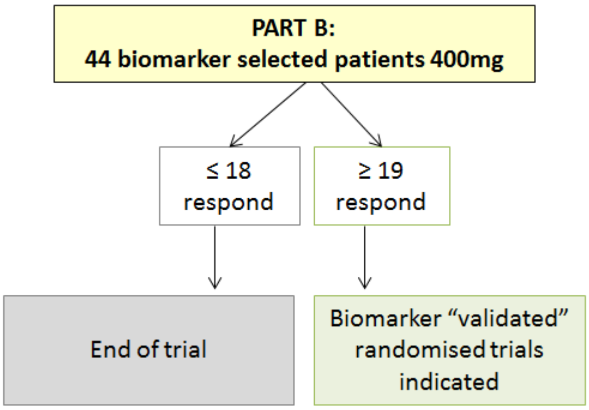

Part B: optimal dose selection

1-stage approach applied to each group (Ho RR≤30%, Ha RR≥50%, alpha 0.05, power 0.85)

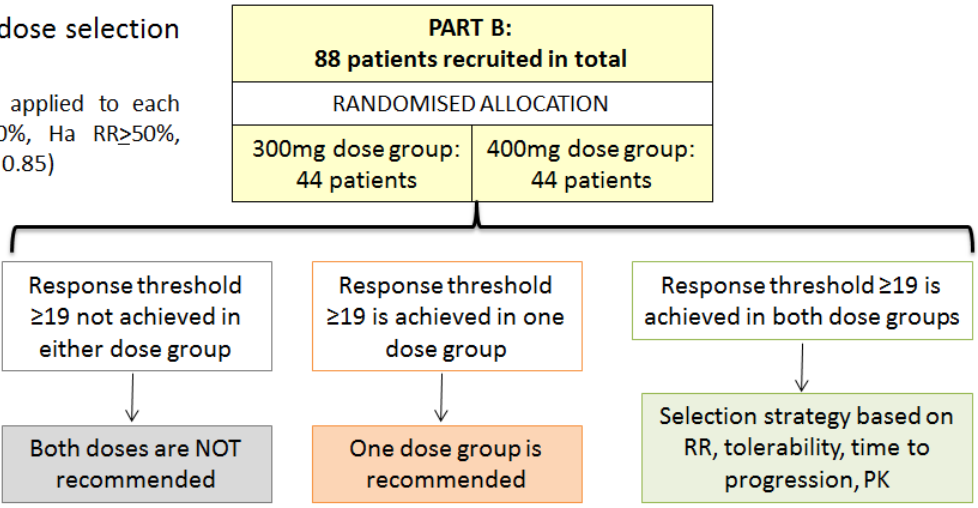

7 PATIENT SELECTION & ELIGIBILITY

Subjects with advanced progressing CRPC who have had either one or two previous lines of taxane-based chemotherapy will be invited to participate in the TOPARP study.

Eligibility criteria for this study have been carefully considered to ensure the safety of the study subjects and to safeguard the integrity of the study results.

7.1 Number of Patients

Part A: 30-45
Part B: 44 evaluable patients in each group (88 in total)

7.2 Source of Patients

Several oncology centres in the United Kingdom with extensive experience in conducting phase II/III trials have agreed to participate in this trial. Patients who have progressing castration-resistant metastatic prostate cancer will be offered participation in this trial.

Part B only – a pre-screening assessment to confirm presence of the selected biomarkers from Part A will be performed at the central laboratory prior to formal screening for entry into Part B.

7.3 Inclusion Criteria

Each patient must meet the following criteria to be enrolled in the study:

1. The subject is capable of understanding and complying with the protocol requirements and has signed the informed consent document.
2. Age ≥ 18 years.
3. Histologically confirmed adenocarcinoma of the prostate with archival tumour tissue available for molecular analyses. If the patient does not have a prior histological diagnosis then the planned baseline fresh biopsy may be used for both the purpose of confirming the histological diagnosis prior to trial entry and for subsequent biomarker analysis. All patients must be willing to have fresh biopsies to obtain tumour tissue for biomarker analysis
   Note: In part B, fresh biopsies are optional in those patients for whom sufficient archival tumour tissue is available for biomarker studies.
4. At least one but no more than two previous taxane-based chemotherapy regimens. If docetaxel chemotherapy is used more than once, this will be considered as one regime. Patients may have had prior exposure to cabazitaxel treatment. At least 28 days since the completion of prior therapy, including major surgery, chemotherapy and other investigational agents. Additionally, clinically relevant sequelae should have resolved to grade 1 or less prior to recommencing treatment. For hormonal treatment and radiotherapy refer to the guidelines below:

- At least 28 days since the completion of prior flutamide treatment. Patients whose PSA did not decline in response to antiandrogens given as a second line or later intervention will only require a 14 days washout prior to Cycle 1, Day 1.
- At least 42 days since the completion of prior bicalutamide (Casodex) and nilutimide (Nilandron) treatment. Patients whose PSA did not decline for 3 or 4 months in response to antiandrogens given as second line or later intervention will require only a 14 day washout period prior to Cycle 1 Day1.
- At least 14 days from any radiotherapy with the exception of a single fraction of radiotherapy for the purposes of palliation (confined to one field) is permitted.

5. Documented prostate cancer progression as assessed by the investigator with one of the following:
    - PSA progression defined by a minimum of three rising PSA levels with an interval of ≥ 1 week between each determination. The PSA value at the Screening visit should be ≥ 2 µg/L (2 ng/ml); patients on systemic glucorticoids for control of symptoms must have documented PSA progression by PCWG2 while on systemic glucocorticoids prior to commencing Cycle1 Day1 of treatment.
    - Radiographic progression of soft tissue disease by modified RECIST1.1 (appendix E) criteria or of bone metastasis with two or more documented new bone lesions on a bone scan with or without PSA progression.
6. Surgically or medically castrated, with testosterone levels of < 50 ng/dL (< 2.0 nM). If the patient is being treated with LHRH agonists/antagonists (patient who have not undergone orchiectomy), this therapy must have been initiated at least 4 weeks prior to Cycle 1 Day 1 and must be continued throughout the study.
7. Eastern Cooperative Oncology Group (ECOG) Performance Status of ≤ 2 (Karnofsky Performance Status ≥ 50%).
8. Life expectancy > 12 weeks.
9. Patient must be able to swallow a whole tablet.
10. Patient and the patient's partner of childbearing potential, must agree to use medically accepted methods of contraception (e.g., barrier methods, including male condom, female condom, or diaphragm with spermicidal gel) during the course of the study and for 3 months after the last dose of study drug. Agreeable to have all the biomarker studies including the paired fresh tumour biopsies.
    Note: For Part B paired fresh tumour biopsies are optional however a fresh tumour biopsy will be required during pre-screening if no archival tumour tissue is available for molecular analyses.
11. Subjects must have a CTC count of ≥5 cells/7.5mls blood at screening confirmed by the central laboratory.
    Note: For part B of the study, the minimum CTC count at screening is not mandatory if the patient has measurable disease according to modified RECIST1.1 (appendix E) and a lesion >2 cms in size at screening and screening PSA ≥ 2 µg/L (2 ng/ml).
12. Subjects must have adequate bone marrow, hepatic and renal function documented within 7 days of registration (TOPARP-A) / randomisation (TOPARP – B), defined as:
    - Haemoglobin ≥9 g/dL. Patients who have anaemia due to bone disease will be permitted to have a blood transfusion prior to trial entry to ensure they meet the eligibility criteria.
    - Absolute neutrophil count ≥1.5x10^9/L
    - Platelets ≥100 x10^9/L

- Total bilirubin ≤1.5 x upper limit of normal (ULN) except for patients with known Gilbert's syndrome.
- Aspartate transaminase (AST) (SGOT) and alanine transaminase (ALT) (SGPT) ≤2.5 x ULN or ≤5 x ULN in the presence of liver metastases.
- Serum creatinine ≤1.5 x ULN or a calculated creatinine clearance >40mL/min for patients with creatinine levels above institutional normal. For GFR estimation, the Cockcroft and Gault equation should be used:
  GFR = CrCl (ml/min) = (140 - age) × wt (kg)/(serum creatinine × 72)
- Albumin >25 g/l

13. For Part B only - Subjects must have genomic defects associated with olaparib sensitivity as identified by next generation sequencing (anticipated to be approximately 30% of patients) confirmed by the central laboratory or, if there is a next generation sequencing result report from an external laboratory, the patient could be enrolled prior to confirmation of these results by the central laboratory **following <u>approval by the Chief Investigator</u>** (in such cases the tumour sample will still be tested at the central laboratory).

7.4 Exclusion Criteria

Patients who meet any of the following criteria will be excluded from the study:

1. Surgery, or local prostatic intervention (excluding a prostatic biopsy) less than 28 days of Cycle 1 Day 1.
2. Less than 28 days from any active anticancer therapy or investigational agents. For hormonal treatment and radiotherapy refer to the guidelines outlined in the inclusion criteria.
3. Prior treatment with a PARP inhibitor, platinum, cyclophosphamide or mitoxantrone chemotherapy.
   Note: If patients have received a treatment with the listed compounds for non- prostate cancer, ICR-CTSU should be contacted to facilitate discussions with the CI as to the patients' suitability.
4. Uncontrolled intercurrent illness including, but not limited to, active infection, symptomatic congestive heart failure (New York Heart Association Class III or IV heart disease), unstable angina pectoris, cardiac arrhythmia, uncontrolled hypertension or psychiatric illness/social situations that would limit compliance with study requirements.
5. Any acute toxicities due to prior chemotherapy and / or radiotherapy that have not resolved to a NCI-CTCAE v4.02 grade $\leq$1 with the exception of chemotherapy induced alopecia and grade 2 peripheral neuropathy.
6. Malignancy within the previous 2-years with a > 30% probability of recurrence within 12 months with the exception of non-melanoma skin cancer, in-situ or superficial bladder cancer.
7. Patients with myelodysplastic syndrome/acute myeloid leukaemia.
8. Patients with known symptomatic brain metastasis are not suitable for enrolment. Patients with asymptomatic, stable, treated brain metastases are eligible for study entry.
9. Patients with symptomatic or impending cord compression unless appropriately treated beforehand and clinically stable and asymptomatic.
10. Patients who have experienced a seizure or seizures within 6 months of study treatment or who are currently being treated with cytochrome P450 enzyme inducing anti-epileptic drugs for seizures (use of anti-epileptic drugs to control pain is allowed in patients not suffering from seizures unless drug is excluded due to CYP3A4 induction - phenytoin, carbamazepine, phenobarbital (see Section 12.13).
11. Patients receiving any of the following classes of inhibitors of CYP3A4 (see Section 12.13 for guidelines and wash out periods);
    - Azole antifungals
    - Macrolide antibiotics
    - Protease inhibitors
12. Patients with gastrointestinal disorders likely to interfere with absorption of the study medication.
13. Initiating bisphosphonate therapy or adjusting bisphosphonate dose/regimen within 30 days prior to Cycle 1 Day 1. Patients on a stable bisphosphonate regimen are eligible and may continue.
14. Presence of a condition or situation, which, in the investigator's opinion, may put the patient at significant risk, may confound the study results, or may interfere significantly with patient's participation in the study.

15. The subject is unable or unwilling to abide by the study protocol or cooperate fully with the investigator or designee.

7.5 Restrictions

1. Patients must refrain from fathering a child or donating sperm during the study and for 3 months following the last dose of olaparib. Patients of child bearing potential and their partners, who are sexually active must agree to the use of two highly effective forms of contraception throughout their participation in the study and for 3 months after the last dose of study drug. Reliable methods of contraception should be used consistently and correctly. Acceptable methods of contraception to be used in this study include:

   - Condom with spermicide and one of the following:
     - Oral contraceptive or hormonal therapy (e.g. hormone implants)
     - Placement of an intra-uterine device

   - Acceptable non-hormonal birth control methods include:
     - Total sexual abstinence. Abstinence must be for the total duration of the study and for 3 months following the last dose of olaparib
     - Vasectomised sexual partner plus male condom. With participant assurance that partner received post-vasectomy confirmation of azoospermia
     - Tubal occlusion plus male condom with spermicide
     - Intrauterine Device (IUD) plus male condom and spermicide - Provided coils are copper-banded

   - Acceptable hormonal methods:
     - Etonogestrel implants (e.g., Implanon, Norplan) and male condom with spermicide
     - Normal and low dose combined oral pills+male condom with spermicide
     - Norelgestromin/ethinyl estradiol (EE) transdermal system+male condom with spermicide
     - Intravaginal device and male condom with spermicide (e.g., EE and etonogestrel)
     - Cerazette (desogestrel) and male condom with spermicide. Cerazette is currently the only highly efficacious progesterone based pill.

2. No other chemotherapy or other anticancer treatment is to be permitted with the exception of continued use of LHRH agonist/antagonists, during study participation. If possible, initiation of a new line of treatment will not be earlier than 30 days post date of last dose. At least a 10 day washout period of olaparib is recommended to ensure patient safety. Palliative radiotherapy is allowed for pre-existing small areas of painful metastases that cannot be adequately be managed with analgesics as long as the patient does not meet the criteria of disease progression or treatment discontinuation.

3. Live virus and bacterial vaccines should not be administered whilst the patient is receiving study medication and during the 30 day follow up period. An increased risk of

infection by the administration of live virus and bacterial vaccines has been observed with conventional chemotherapy drugs and the effects with olaparib are unknown.

4. In vitro data have shown that the principal enzyme responsible for the formation of the 3 main metabolites of olaparib is CYP3A4 and consequently, although the contribution of metabolic clearance to total drug clearance in man is currently unknown, **to ensure patient safety all patients must avoid concomitant use of drugs, herbal supplements and/or ingestion of foods known to modulate CYP3A4 enzyme activity** (see Section 12.13) from the time they enter the screening period until 30 days after the last dose of study medication.

8 OVERVIEW OF STUDY PROCEDURES

8.1 Informed Consent

The study objectives, overall requirements, potential benefits and risk will be discussed with potentially eligible patients. Interested patients will be given a TOPARP patient information sheet. Consent must be obtained only after allowing the patient sufficient time for consideration, and the opportunity to ask any further questions. A signed, TOPARP written informed consent form must be obtained from the patient in accordance with local practice and regulations. Pre-entry tests, not performed routinely as part of standard patient management, will only be done after the TOPARP consent form has been signed and personally dated by both the patient and the Investigator. Confirmation of the patient's informed consent and the informed consent process must be documented in the patient's medical notes. A copy of the signed form will be provided to the patient and the original retained in the investigator site file and must be available for verification by ICR-CTSU study staff or for regulatory audit/inspection at any time. Patients who consent on the TOPARP study, consent to having fresh tumour biopsies (Part A only), archival tumour tissue, CTCs and plasma collected for molecular and biomarker studies. Please refer to section 15.8 regarding consent for genetic testing.

For Part B, assessment of eligibility based on the selected biomarkers from Part A will be performed by the central laboratory prior to screening for the main part of the study. A "pre-screening patient information sheet" will be available for patients consenting to assess their tumour sample for the presence of the biomarker, prior to consenting to the main study.

Patients at participating centres, who wish to have diffusion weighted MRI (DW-MRI) scans to assess their disease during the study, will be required to sign a specific DW-MRI consent form. Participation in this component of the study is entirely voluntary. Refusal to participate in the DW-MRI component of the study will not result in ineligibility to participate in the main clinical trial and will not impact the medical care received.

8.2 Part A - Registration, Eligibility and Trial Entry

8.2.1 Registration Part A

Once informed consent has been obtained, patients must be registered for screening by contacting the ICR CTSU:

**To register a patient telephone ICR-CTSU**<br>On: 0208 643 7150<br>(09.00 – 17.00 Monday to Friday)

The following information will be required when registering the patient:

- Name of cancer centre, treating hospital, consultant and person registering patient
- Confirmation that patient has given written informed consent
- Patients initials and date of birth

- Confirmation of diagnosis

The patient will be allocated a unique registration number.

8.2.2 Eligibility status and trial entry Part A

Once all screening evaluations have been completed, an eligibility checklist should be completed by the clinician/research nurse. The eligibility checklist should be completed for all patients registered.

For those patients who remain eligible, the clinician / research nurse should contact ICR-CTSU to confirm eligibility and obtain a trial number.

**To confirm patient eligibility telephone ICR-CTSU**<br>On: 0208 643 7150<br>(09.00 – 17.00 Monday to Friday)

The following information will be required:

- Patient's full name, hospital number, NHS number, postcode and trial registration number
- Confirmation of the patient's eligibility status according to the eligibility checklist
- Confirmation that the evaluable disease being followed in the trial has been assessed by either CT or MRI and a bone scan within 28 days of scheduled start date of treatment
- Confirmation of consent for the trial

If the patient is confirmed as eligible for trial entry, the caller will be given the patient's unique trial identification number (Trial ID), and fax confirmation will be sent to the appropriate pharmacist to enable dispensing of the study drug.

An electronic screening log (in MACRO) should be completed for all registered patients who do not subsequently enter the study. This will record reasons for exclusion e.g. because screening tests identified that they were ineligible or the patient subsequently withdrew consent.

8.3 Part B - Registration, Eligibility and Randomisation

8.3.1 Pre-registration Part B

All participating sites will be required to keep a pre-registration log of all participants with mCRPC that are potentially eligible for this study. The information collected on the log will include:

- Date patient identified

- Screening outcome (patient approached/accepted participation/declined participation)
- Reasons for not approaching / declining participation (if available)
- Registration and Trial ID (if applicable)

This information will be used to monitor recruitment activity. No patient identifiable data will be sent to ICR-CTSU at this stage.

8.3.2 Registration Part B

Once informed consent has been obtained to assess the tumour sample for the presence of the biomarker and collect saliva, patients must be registered by contacting the ICR CTSU:

**To register a patient telephone ICR-CTSU**
On: 0208 643 7150
(09.00 – 17.00 Monday to Friday)

The following information will be required when registering the patient:

- Name of cancer centre, treating hospital, consultant and person registering patient
- Confirmation that the patient has given written informed consent on the pre-screening consent form OR the Chief Investigator has confirmed presence of the biomarker based on a next generation sequencing report from another laboratory
- Patients initials and date of birth

The patient will be allocated a unique registration number.

8.3.3 Eligibility status and randomisation to Part B

Once presence of the biomarker has been confirmed by the central laboratory (via ICR-CTSU) all screening evaluations should be performed. An eligibility checklist and a randomisation checklist should be completed by the clinician/research nurse. The eligibility checklist should be completed for all patients registered.

Patients must be randomised centrally by the trials unit (ICR-CTSU) before trial treatment can commence.

**Patients should be randomised by telephoning ICR-CTSU**
On: 0208 643 7150
(09.00 – 17.00 Monday to Friday)

Randomisation should take place as close to the planned start date of treatment as possible.

The following information will be required at randomisation:

- Patient's full name, hospital number, NHS number, postcode and trial registration number
- Confirmation of the presence of the biomarker required for Part B (confirmed by ICR or the Chief Investigator)
- Confirmation of the patient's eligibility status according to the eligibility checklist
- CTC count at screening
- Confirmation of modified RECIST1.1 (Appendix E) evaluable disease **OR** a CTC count ≥5 cells/7.5ml blood (confirmed by the central lab if the patient does not have RECIST1.1 measurable disease) been confirmed within 28 days
- Confirmation of consent for the trial, optional sub studies and preferences regarding genetic information

The caller will be given the patient's unique randomisation number (Trial ID) and treatment allocation.

ICR-CTSU will send confirmation to the data management contact and pharmacist at the recruiting site to confirm a patients' entry into the trial.

An electronic screening log (in MACRO) should be completed for all registered patients who do not subsequently enter the study. This will record reasons for exclusion e.g. because screening tests identified that they were ineligible or the patient subsequently withdrew consent.

9 OVERALL STUDY ACTIVITIES

9.1 Pre-screening period – Part B only

Patients will have to be tested for the presence of the selection biomarker prior to enrolment for Part B. This test can be performed at any time prior to the patient participating in the trial. Patients will be offered the TOPARP pre-screening written informed consent to allow for biomarker testing of their archival tumour samples or fresh CRPC sample (preferred option), circulating free plasma tumour DNA and/or saliva. Alternatively, if patients have been tested already and the presence of the biomarker is documented and the Chief Investigator has confirmed the presence of the biomarker, the pre-screening stage may not be necessary and saliva will be collected during the screening period. Please note all patients will need to be registered before they are randomised.

9.2 Screening Period

All patients must sign the TOPARP written informed consent before study specific screening procedures can be performed. Consent may be obtained up to 28 days prior to Cycle 1 Day 1. Patients will have a screening period of up to 28 days prior to Cycle 1 Day 1. The laboratory investigations (haematology, electrolytes, tumour markers) must be performed within 14 days of starting treatment on olaparib and testosterone levels within 28 days of starting treatment on olaparib. Results of CTC enumeration performed at the central laboratory in blood samples collected within 28 days of starting treatment will be acceptable. For details of the schedule and nature of the screening assessments, refer to Table 3. All the screening assessments must be complete and eligibility criteria met prior to enrolment / randomisation into the study.

Patients who meet the eligibility and screening requirements will return to the site for the Cycle 1 Day 1 visit. As part of the screening process, CTCs, a pre dose biopsy and blood samples will be collected for the predefined biomarkers of interest. In part B, fresh tumour biopsies are optional.

9.3 Treatment Period

All screening assessments must be completed and reviewed prior to the commencement of Cycle 1 Day 1 assessments. Olaparib will be administered orally twice daily, once in the morning and once in the evening, continuously. Patients may receive continuous treatment for as long as they remain free from intolerable toxicity, and, in the investigator's opinion, are receiving clinical benefit. The patient will return on Day 8 and Day 15 (Part A only) of Cycle 1 and subsequently on the first day of each treatment cycle in order to evaluate safety, tolerability and dosing compliance. From Cycle 2 to end of study treatment patients' Day 1 visits will occur on a 28 day cycle with a +/- 2 day window. If the study window is utilised every effort will be made for the patient to return to schedule. Patients will have additional imaging visits up to 8 days before Cycle 4 Day 1, Cycle 7 Day 1, Cycle 10 Day 1, and every 3 cycles thereafter. For details of the schedule and nature of the on-going assessments, refer to Table 3. The dose of olaparib may be reduced and/or a treatment

delayed in patients experiencing toxicities according to the dose modification guidelines as set out in section 12.8. Safety parameters will be continuously followed-up during the duration of the trial.

Part B only – patients randomised to receive 300mg bd will (if clinically indicated and no dose reductions have been made) be offered a dose escalation to 400mg bd following progression on 300mg bd as described in section 10.9.

9.4 End of Treatment (EOT) Visit

All patients will return for an end of treatment visit 30 -/+5 days from the time of last dose of olaparib treatment or prior to the start of next therapy, whichever happens first for safety and end of study treatment assessments. For details of the schedule and nature of the assessments for this end of study treatment visit, refer to Table 3 and section 11.2.6.

9.5 Follow Up Visits

Following treatment completion, patients will be followed for survival every 3 months.

10 OVERALL TRIAL ASSESSMENTS

10.1 Clinical Measurements

The Investigator will ensure that all data collected in the study are provided to ICR-CTSU. He/she ensures the accuracy, completeness, legibility and timeliness of the data recorded in the appropriate sections of the Electronic Case Report Form (eCRF) in accordance with instructions provided.

10.2 Medical History

Medical and Surgical History

Medical and surgical history, including a history of the disease being studied, other significant past and on-going conditions should be recorded. All previous radiotherapy and chemotherapy, as well as any other therapy received for prostate cancer, must be documented.

Recording of Concomitant Medications

The use of or any change in the patient’s concomitant medications will be recorded when the patient visits the hospital (including a review of the patient’s diary card for any self-administered medication).

Current Signs and Symptoms

Current signs and symptoms of the disease will be recorded, together with the appropriate NCI-CTC grade and causality, if appropriate.

10.3 Physical Examination

Evaluations should be performed by the same evaluator throughout the study whenever possible. A full physical examination will include the assessment of the body systems listed below and reporting if these findings were normal, abnormal or not done. A full examination will be conducted at the screening visit, cycle 1 day 1 and the end of study treatment visit. At all other times the extent of the examination beyond vital signs and weight will be conducted as clinically indicated. Height will be recorded at the screening visit only. Vital signs include blood pressure, heart rate, respiratory rate, and oral or aural body temperature.

The following must be assessed and recorded:

- General appearance
- Cardiovascular

- Respiratory
- Gastrointestinal
- Genitourinary
- Central Nervous System
- Dermatological
- Musculoskeletal
- Peripheral lymph nodes
- Other

10.4 ECG Monitoring

ECGs will be recorded at 25 mm/sec. All ECGs should be assessed by the Investigator as to whether they are clinically significantly abnormal / not clinically significantly abnormal. An ECG assessment will be performed at screening as a baseline assessment. Subsequent ECGs will only be performed if clinically indicated.

10.5 Brief Pain Scores

The BPI-SF will be self-administered in the clinic to the patients during screening, on the first day of each treatment cycle, at the End of Treatment Visit and, if applicable, the dose escalation visit and the results recorded in the patient's notes (Appendix I).

10.6 Blood, Tissue and Other Sampling

Blood samples for safety assessments will be done at the local institutes. Tests relating to biomarker work on CTC, plasma and tumour material will be performed primarily at The Institute of Cancer Research Cancer Biomarkers Laboratory. Samples will be collected and processed according to specified detail in the TOPARP Laboratory Manual. There may be a requirement to send some of the samples to be processed in a different laboratory. This will be done with contractual agreements in place and patient details will remain confidential. The pharmacokinetic analyses in Part B will be conducted at a laboratory contracted by the co-sponsors.

Bone marrow or blood cytogenetic analysis may be performed according to standard haematological practice for patients with prolonged haematological toxicities as defined in Section 3.3. Bone marrow analysis should include an aspirate for cellular morphology, cytogenetic analysis and flow cytometry, and a core biopsy for bone marrow cellularity. If it is not possible to conduct cytogenetic analysis or flow cytometry on the bone marrow aspirate, then attempts should be made to carry out the tests on a blood sample. If findings are consistent with MDS/AML, study drug should be discontinued and a full description of findings should be submitted with an SAE report by the investigator to ICR-CTSU (see section 13).

Routine clinical laboratory tests will include the following;

Table 2: List of Laboratory Tests

<table>
<tr><td colspan="2">Routine Local Laboratory Tests conducted each cycle</td><td>Tests at specified time points or for specified events</td></tr>
<tr><td>Haematology:
• Neutrophils
• Haemoglobin (Hgb)
• Platelet count with differential
• White blood cell (WBC) count
• Lymphocytes
• Coagulation Factors: Prothrombin Time (PT), Partial Thromboplastin Time (PPT), International Normalized Ratio (INR)</td><td>Serum Chemistry:
• Albumin (ALB)
• Alkaline phosphatase (ALK-P)
• Alanine aminotransferase (ALT;SGPT)
• Gamma-glutamyltransferase (GGT)
• Amylase
• Aspartate aminotransferase (AST;SGOT)
• Blood urea nitrogen (BUN)
• Calcium (Ca)
• Chloride (Cl)
• Creatinine (Cr)
• Random Glucose
• Lactate dehydrogenase (LDH)
• Magnesium
• Phosphate
• Potassium (K)
• Sodium (Na)
• Total bilirubin
• Total protein
• Uric acid</td><td>Urinalysis dipstick (Screening only) for:
• Blood
• Protein
• Random Glucose
• (Microscopic examination if abnormal)

Additional laboratory tests:
• Prostate specific antigen (PSA) (Screening; Cycle 1, Day 1; Cycle 4, 7, 10 and every 3rd Cycle; dose escalation, EOT)
• Serum testosterone (Screening)

In the event of protracted myelosupression:
• Bone marrow or blood cytogenetic analysis</td></tr>
<tr><td colspan="3">Central Laboratory Tests</td></tr>
<tr><td>• Circulating Tumour Cells (CTCs) for enumeration & molecular characterisation
• Circulating tumour DNA</td><td>• Circulating tumour microRNA (miRNA)
• Plasma for biomarkers</td><td>• Mandatory tumour biopsy
• Urine telopeptides</td></tr>
</table>

10.7 Efficacy Measurements

CT or MRI Scan Assessments (Modified RECIST1.1 (appendix E))

Baseline contrast-enhanced CT of the chest, abdomen and pelvis should be performed for the assessment of measurable lesions. Where contrast is contraindicated then either an MRI of the chest, abdomen and pelvis or a non-contrast CT scan can be performed if the measurable disease is easily discernible without contrast. All baseline CT will be performed during the 28-day screening window as close as possible to Cycle 1, Day 1 of treatment. The CT scans will be repeated up to 8 days before Cycle 4 Day 1, Cycle 7 Day 1, Cycle 10 Day 1 and every 3rd cycle subsequently (Table 3). CT scans may be performed earlier if clinically indicated. If a patient remains on treatment for >2 years, CT/MRI scans should be undertaken every 3rd cycle or as per SOC but at least every 24 weeks. Scans that were performed as part of standard of care prior to signature of the informed consent form can be analysed for the purposes of the study if they were performed within the correct time frame and are of sufficient quality. Subsequent, repeat imaging requires the same method of assessment to characterise each identified lesion at baseline and during follow-up. Response categories: complete response (CR), partial response (PR), stable disease (SD), and progressive disease (PD) (refer to Appendix E) will be assigned at the scheduled visits. In the case of stable disease, measurements must have met the stable disease criteria at least once after study start for a minimum interval of 12 weeks. PR or CR by RECIST must be confirmed with a repeat tumour assessment. This should be conducted at the next study visit that is at least 4 weeks after the criteria for response was met.

For the purposes of analysis, overall response using Investigator measurements of target lesions, assessment of non-target lesions and new lesions collected on the eCRF to derive the best overall response, and other protocol endpoints using these data. All medical images and tumour response assessments will be collected and retained on site as source data. A central image review may be performed by the sponsor if required.

Bone Scan Assessments

Bone scans will be used to assess bone lesions. Baseline bone scans will be performed during the 28 day screening window as close as possible to Cycle 1 Day 1, and up to 8 days prior to Cycle 4 Day 1, Cycle 7 Day 1, Cycle 10 Day 1 and every 3rd cycle subsequently (Table 3). If a patient remains on treatment for >2 years, bone scans should be undertaken every 3rd cycle or as per SOC but at least every 24 weeks. Scans that were performed as part of standard of care prior to signature of the informed consent form can be analysed as the baseline study if they were performed within the correct time frame and are of sufficient quality. **Bone deposits will be reported as absence or presence of new bone lesions. New lesions at the first scheduled reassessment at Week 12 must be confirmed by a second scan performed 6 or more weeks later. Disease progression by bone scan will be defined as two new bone lesions at the first ‘progression’ scan, confirmed by a further scan at least 6 weeks later (see section 10.9 for more details).** All medical images and tumour response assessments will be collected and archived locally.

Diffusion weighed-MRI (DW-MRI) Scan Assessments

DW-MRI constitutes an optional component of the study. DW-MRI provides additional information regarding tumour cellularity and tumour cell death that cannot be evaluated with other imaging techniques. DW-MRI will be obtained at baseline (screening), up to 8 days prior to Cycle 4 Day 1, Cycle 7 Day 1, Cycle 10 Day 1 and every 3 cycles subsequently. These results of these scans will not be utilized for the purpose of assigning disease status. Patients who wish to participate in this optional component of the study will be required to sign a separate Patient Informed Consent Form (PICF).

DW-MRI scans should be sent to ICR-CTSU for central assessment as per the DW-MRI imaging manual

PSA Measurements

PSA will be assessed at baseline (Cycle 1 Day 1) and routinely on day 1 of every 3rd cycle (12 weeks); dose escalation and EOT by PCWG2 criteria (refer to Appendix D). Increases and decreases will be tracked in order to assess PSA progression and response. PSA changes from baseline to 12 weeks (either increment or decline) and maximal change in PSA will be recorded. Responses by PCWG2 criteria must be confirmed 28 days later.

Although serial PSA will be measured on this study and PSA progression documented by the PCWG2 criteria, the PSA value on its own is not considered a reliable measure of clinical benefit and does not correlate tightly with survival. PSA flares may occur early in the treatment and may therefore confound response assessment. Additionally, there is emerging data that PARP-1 is implicated in multiple other essential cellular functions separate from DNA repair including *ETS* gene-mediated transcription regulation which may have a bearing on the response assessments by PSA. PSA reading should not be applied as the sole criteria to consider treatment discontinuation. In absence of evidence of radiological or clinical evidence of disease progression, patients should continue in the study despite an increment in PSA. **Overall, therefore patients will not be taken off trial purely for rising PSA levels in the absence of worsening scans and symptoms since PARP inhibitors are likely to impact tumour growth without impacting PSA secretion.**

PSA response and PSA progression will be defined according to the consensus guidelines of the PCWG2 criteria:

- PSA partial response is defined as a ≥ 50% decline in PSA from baseline PSA value. This PSA decline must be confirmed to be sustained by a second PSA value obtained 4 or more weeks later.

- For patients with PSA declines after baseline, the PSA progression date is defined as the date that a ≥25% increase and an absolute increase of ≥ 2 ng/mL above the nadir (minimum value observed so far) is documented, which is confirmed by a

second consecutive value obtained four or more weeks later. For patients with no PSA declines after baseline and beyond 12 weeks, PSA progression date is defined as the date that a ≥25% increase and an absolute increase of ≥ 2ng/mL above the baseline is documented, which is confirmed by a second consecutive value four or more weeks later.

CTC Conversion Rate

CTCs will be obtained at various time points before, during and after treatment for enumeration and molecular characterisation (Table 7). CTC enumeration will be performed at The Institute of Cancer Research laboratories using the analytically valid CellSearch system (Veridex, LLC). For patients with baseline CTC counts of ≥ 5 cells/7.5 ml of blood, a conversion is defined as a nadir decline in the CTC count to < 5 cells/7.5 ml of blood. The conversion of CTC counts that are ≥ 5 to < 5 and percentage decline of ≥ 30% indicative of clinical benefit and improved survival outcome will be measured (76,77,78).

A central review of bone scans and CT scans will take place. Anonymised copies of bone and CT scans should be sent to ICR-CTSU for central review on their request.

10.8 Safety Measurements

Safety parameters will be continuously assessed throughout the study prior to each cycle of treatment. These will include:

- Medical history, vital sign measurements, physical examination, and body weight
- Haematology, clinical chemistry
- Eastern Cooperative Oncology Group (ECOG) performance status
- Assessment of concomitant medications
- Adverse events (AEs) and serious adverse events (SAEs) including laboratory test AEs will be graded and summarised according to the NCI CTCAE version 4.02

10.9 Criteria For Discontinuation Of Study Treatment

The consensus guidelines of the PCWG2 recommend that confirmed radiographic progression is the most reliable indicator of clinical benefit in patients with CRPC. Based on this consideration, patients should be maintained on study treatment until confirmed radiographic progression or clinical progression or if the treating physician decides to initiate new systemic anti-cancer therapy. It is recommended that if the patient has PSA progression but no unequivocal clinical or radiological progression and an alternate treatment is not indicated, the investigators should maintain the patient's treatment until radiological disease progression is documented and confirmed. However, if the patient has unequivocal clinical progression without radiographic progression, these patients could be considered for other therapy at the discretion of the treating physician. In this circumstance, study treatment should be stopped and the patient advised regarding available treatment options. The intent of these criteria is to maintain study treatment for patients with PSA progression, given the absence of alternative treatment options available to participants who have failed both castration and chemotherapy and the high likelihood that some clones

of prostate cancer may be benefiting in the context of radiological stability despite a PSA rise.

A patient will discontinue study treatment due to progression of disease if one or more of the following findings occur:

1. **Progression by bone scan:**
   - Progression will not be defined on a scan performed before 12 weeks from Cycle 1 Day 1 as this may be indicative of a flare response.
   - The first bone scan with ≥ 2 new lesions compared with baseline is observed ≥12 weeks and ≤ 13 weeks from cycle 1 day 1 and is confirmed by a second bone scan taken ≥ 6 weeks later showing 2 additional new lesions (i.e. a total of ≥ 4 new lesions compared with the baseline bone scan).
   - The first bone scan with ≥ 2 new lesions compared with baseline is observed > 13 weeks from cycle 1 day 1 and the new lesions are verified on the next bone scan ≥ 6 weeks later (i.e. a total of ≥ 2 new lesions compared with baseline).

2. **Progression of soft tissue /visceral disease by modified RECIST1.1 (appendix E):**
   - A patient will be determined to have progressed if they have progression of target lesions, clear progression of existing non-target lesions, or the appearance of one or more new lesions by modified RECIST1.1 (appendix E). **Lymph nodes have to be >2 cm in size to be deemed measurable independent of the method used.**

3. **Unequivocal evidence of clinical progression:**
   A patient can be taken off study at the discretion of the treating physician under the following circumstances:
   - Marked escalation in cancer related pain that is assessed by the investigator to indicate the need for other systemic chemotherapy.
   - Immediate need for initiation of new anticancer treatment, surgical or radiological intervention for complications due to tumour progression even in the absence of radiological progression.
   - Marked deterioration in ECOG performance status to grade 3 or higher felt by the investigator to indicate clinical progression.
   - Felt to be in the best interest of the patient to come off study due to clinical progression.

4. **Death from any cause:**
   When study treatment is discontinued due to unequivocal clinical progression, attempts should be made to determine the patient's status at the End of Treatment Visit to assess for radiographic progression, including a confirmatory bone scan, as deemed appropriate by the treating team.

Note: patients in Part B allocated to the 300mg group will be allowed to dose-escalate to 400mg after an event qualifying as progression if clinically indicated and the patient did not have a dose reduction from 300mg BID. The date of progression would be the first date of the imaging assessment in which an event as listed above is observed or date clinician confirmed clinical progression.

10.10 Withdrawal from Study Treatment

Criteria for withdrawal of study treatment are outlined below:

1. Patient Withdraws Consent

Patients have the right to withdraw from the study at any time, without prejudice to their medical care, and are not obliged to state their reasons. If the patient withdraws consent, he should be encouraged to have a complete End of Treatment (EOT) visit and effort should be extended to clarifying the reason for the withdrawal.

Investigator may choose to withdraw a patient at any point if this is considered to be in the patient's best interest. The patient must be discontinued from the study treatment due to any of the safety reasons outlined below:

2. Sustained or Recurrent Unacceptable Side Effects:

- Any grade 3 or 4 events that have not reverted to grade 1 or the baseline (Cycle 1 Day 1) grade after a 42 days break off treatment with appropriate medical intervention. At the Investigator's discretion, patients may be considered for dose reductions [see Section 12.8 for management of toxicity & dose adjustments. If the grade 3 or 4 adverse events recurs after successive dose reductions to the lowest dose level (200 mg BID) then the patient must be discontinued].
- Unacceptable adverse events or any other toxicity in the investigator's opinion.
- Serious intercurrent illness or significant worsening of intercurrent illness that prevents further administration of treatment.
- A severe drug reaction or a hypersensitivity reaction to olaparib.

3. Non-compliance with Dosing or Trial Protocol Requirements

Patient is unable to adhere to study visit schedule or comply with protocol requirements despite several reminders about the requirements and efforts made to identify and resolve the aetiology of the problem may be discontinued at the discretion of the Investigator.

4. Use of Prohibited Concurrent Medication

The patient will be discontinued from the protocol treatment when prohibited drug is administered. Supportive care medications are permitted with their use following institutional guidelines.

5. **Other Reasons**

Additionally, patients may be discontinued from study treatment for any of the following reasons:

- Patient lost to follow-up
- Major protocol violation
- Incorrectly enrolled patients, i.e., the patient does not meet the required inclusion/exclusion criteria for the study

Attempts should be made to contact any patient failing to return for a scheduled visit/follow up, in order to ensure that the reason for not returning is not an adverse event. Likewise attempts should be made to establish whether any patient declaring a desire to discontinue from the study e.g. for personal reasons, is in reality due to an adverse event (AE). If the olaparib therapy is prematurely discontinued, the primary reason for discontinuation must be recorded in the appropriate section of the eCRF. Patients will be required to attend an end of treatment visit at least 30 -/+ 5 days after discontinuing study medication for collection and/or completion of AE information. All new events occurring during this 30-day period must be recorded (if SAEs they must be reported to the ICR-CTSU within 24 hours) and followed-up to resolution, unless in the opinion of the Investigator, the condition is unlikely to resolve due to the patients' underlying disease. Any untoward event occurring subsequent to the 30-day follow-up AE reporting period that the Investigator assesses as possibly related to the study medication should also be reported as an AE. The patient should return all study medication following cessation of the study drug.

In the unlikely event that a patient withdraws consent for further follow-up data to be collected, the appropriate form in the eCRF should be completed and returned to ICR-CTSU. Clarification should be sought to ensure that the patient is not simply withdrawing from study drug. In the extremely unlikely event that the patient wishes to have their data removed from the trial completely, the implications of this should be discussed with the patient to ensure that this is their intent and if confirmed, ICR-CTSU should be notified in writing. If this request is received after results have been published the course of action will be agreed between the Sponsor and independent Trial Steering Committee/Independent Data Monitoring and Steering Committee.

10.11 Patient Replacement Policy

Patients who prematurely withdraw from the study treatment for reasons which aren't treatment or disease related may be replaced. Patients who are found to be ineligible after randomisation may also be replaced. Replacement of patients will be dependent on authorisation by the Independent Data Monitoring Committee (IDMC).

Note: Rising PSA or worsening bone scans in the first 12 weeks is NOT treatment failure and may reflect a flare response.

11 TRIAL ASSESSMENTS BY VISIT

Access to the electronic case report form (eCRF) will be provided by ICR-CTSU for each patient eligible for the trial. The relevant eforms should be completed at each hospital visit.

Note: some of the scheduled blood samples collected at screening, C1D1, C1D8, C2D1, C3D1, C6D1 and end of treatment require the patient to be fasted for 8 hours prior to sampling (see section 15.3 for further details).

11.1 Baseline Assessments

11.1.1 Pre-screening assessments (Part B only)

Pre-screening activities/procedures outlined below will be conducted after the pre-screening consent form has been signed;

- Obtain archival tissue for analysis of the presence of the biomarker. This needs to be shipped to the central laboratory to evaluate the tumour for genomic defects associated with olaparib sensitivity.
- Research Biomarkers
  - Saliva for DNA studies

11.1.2 Screening

Screening activities that involve imaging, CTCs and collection of archival and fresh tumour tissue will be conducted within a 28-day window prior to commencing Cycle 1 Day 1. All other screening activities/procedures outlined below will be conducted during the screening period over 14-days prior to Cycle 1 Day 1.

- Obtain archival tissue for analysis of biomarkers (Days -28 to Day -1) if not obtained pre-screening for Part B. Previous biopsy samples taken prior to TOPARP consent but processed as per the TOPARP Laboratory Manual may be used if within the appropriate window. Please refer to section 15, for further details of specimens to be collected.
- Obtain mandatory fresh tumour biopsy (Days -28 to Day -1). Please refer to section 15, for further details of specimens to be collected. For part B, fresh biopsies are preferred but will be optional to enhance accrual.
- Medical history including details of the following:
  - All co-morbidities
  - Oncology history / family history of cancers
  - Prior prostate cancer history including extent of disease (stage), Gleason score at diagnosis, PSA levels
  - Previous hormonal, cytotoxic, and experimental treatments with start and stop dates
- Demographics
- Listing of current baseline signs and symptoms with associated NCI Common Terminology Criteria for Adverse Events grading (0-4)
- Physical examination

- Weight, and height
- Vital signs including blood pressure, heart rate, respiratory rate, and oral or aural body temperature
- Assessment of ECOG Performance Status
- 12 lead ECG
- Local Laboratory tests:
    - FBC: WBC with differential count, RBC, haemoglobin, hematocrit, platelets.
    - Coagulation studies (PT/PPT, INR)
    - Chemistry with electrolytes: sodium, potassium, chloride, creatinine, BUN, AST, ALT, alkaline phosphatase, total bilirubin, albumin, total protein, amylase, calcium, phosphate, random glucose, uric acid, and magnesium
    - Urinalysis: routine dipstick. Only if the dipstick is abnormal will the urine be sent for analysis
    - PSA: If patient undergoes a digital rectal exam (DRE), PSA must be sampled prior to the DRE
    - Serum testosterone (Days -28 to Day -1)
- Baseline tumour assessment (Days -28 to Day -1)
    - CT and / or other imaging procedures as clinically indicated
    - Bone Scan
    - DW-MRI (optional)
- Pain (BFI-SF) and Analgesic Usage Score
- Concomitant medications listing
    - Obtain a complete list of all prescription and non-prescription (over the counter) medications currently taken including pain medications. This also includes any nutritional supplements and/or herbal preparations.
- Assessments of signs and symptoms
- Research Biomarkers
    - Circulating Tumour Cell (CTC) blood collection for enumeration and molecular characterisation (Days -28 to Day -1). Blood samples taken prior to TOPARP consent may be used for CTC enumeration if within the appropriate window and enumeration was performed at the central laboratory.
    - Circulating Plasma DNA and RNA studies
    - PAXgene miRNA studies
    - Urine markers of bone turnover and RNA
    - Serum Proteomic studies
    - Plasma and urine metabolomics studies
    - Part A only - Buccal swab for DNA studies
    - Saliva for DNA studies if not collected at pre-screening for Part B

11.1.3 Cycle 1 Day 1

Patients who are eligible after screening and who accept to be entered into the study should be entered within 28 days of informed consent. All the screening assessments should have been completed and screenings results reviewed prior to trial entry and the commencement of Cycle 1.

The following procedures should be carried out **prior to dosing of study treatment**:

- Update listing of current baseline signs and symptoms with associated NCI Common Terminology Criteria for Adverse Events grading (0-4)
- Updated oncology history with any events that may have transpired since screening
- Physical examination
  - Full examination
  - Vital signs including blood pressure, heart rate, respiratory rate, oral body temperature
  - Height and weight
- Assessment of ECOG Performance Status
- Local Laboratory Tests:
  - FBC: WBC, RBC, haemoglobin, haematocrit, platelets with differential count.
  - Coagulation studies (PT/PPT, INR)
  - Chemistry with electrolytes: sodium, potassium, magnesium, chloride, creatinine, BUN, AST, ALT, alkaline phosphatase, total bilirubin, albumin, total protein, amylase, calcium, phosphate, random glucose, uric acid
  - PSA: If patient undergoes a DRE, PSA must be sampled prior to the DRE
- Pain (BFI-SF) and Analgesic Usage Score
- Concomitant medications listing
  - Update listing of all prescriptive and non-prescriptive (over the counter) medications taken since screening. This also includes any nutritional supplements and/or herbal preparations
- Assessment of adverse events
- Research Biomarkers
  - Circulating Tumour Cell (CTC)
  - Circulating plasma DNA and RNA studies
  - PAXgene miRNA studies
  - Urine markers of bone turnover (telopeptides) and RNA
  - Serum proteomic studies
  - Plasma and urine metabolomics studies

11.2 On-treatment Assessments

11.2.1 Cycle 1 Day 8 Visit

The following assessments should be carried out on Day 8:

- Brief history and physical examination as clinically indicated
- Vital signs including blood pressure, heart rate, respiratory rate, oral body temperature

- Assessment of ECOG Performance Status
- Local Laboratory Tests:
    - Full blood count including WBC, haemoglobin, platelets
    - Coagulation studies (PT/PPT, INR)
    - Chemistry with electrolytes: sodium, potassium, chloride, creatinine, BUN, AST, ALT, alkaline phosphatase, total bilirubin, albumin, total protein, amylase, calcium, phosphate, random glucose, uric acid, magnesium
- Concomitant Medications listing:
    - Update listing of all prescriptive and non-prescriptive (over the counter) medications taken since screening. This also includes any nutritional supplements and/or herbal preparations
- Adverse events will be monitored throughout the study. At each post-baseline visit, the investigator will begin by querying for adverse events by asking each patient a general, non-directed question such as 'How have you been feeling since the last visit?' Directed questioning and examination will then be done as appropriate.
- Dosing compliance check
- Obtain post treatment mandatory fresh tumour tissue biopsy (Cycle 1 Days 8 to Day 28). For part B, all fresh biopsies are optional.
- Research Biomarkers and Pharmacokinetics
    - Circulating Tumour Cells (CTC)
    - Circulating plasma DNA and RNA studies
    - Plasma and urine for metabolomics studies
    - Part B only - Blood for pharmacokinetics pre-dose, 0.5, 1, 1.5, 2, 4, 6, 8, and 12 hours after the morning dose

11.2.2 Cycle 1 Day 15 Visit – Part A only

- Research Biomarkers
    - Circulating Tumour Cells (CTC)

11.2.3 Day 1 of Cycles 2, 3, 5, 6, 8, 9, 11, and 12 (Continue every cycle beyond Cycle 12)

The following assessments should be carried out at the indicated time point:

- Brief history and physical examination as clinically indicated
- Vital signs including blood pressure, heart rate, respiratory rate, and oral or aural body temperature
- Weight
- Assessment of ECOG Performance Status
- Concomitant Medications listing
    - Update listing of all prescriptive and non-prescriptive (over the counter)

medications taken since screening. This also includes any nutritional supplements and/or herbal preparations.

- AE evaluation and recording at each post-baseline visit
- Part A - PSA measurements will be conducted every 12 weeks. Part B – patients allocated to the 300mg group that pursue dose-escalation to 400mg upon progression, PSA will be checked at day 1 of every visit after the dose escalation. PSA results will be assessed according to PCWG2 criteria.
- Dosing compliance check and a count of study medication will be conducted during this visit and patient dosing compliance will be assessed. If dosing compliance is not 100% in the absence of toxicity, patient should be re-instructed regarding proper dosing procedures.
- Pain (BFI-SF) and Analgesic Usage Score (each cycle)
- Local Laboratory Tests:
    - FBC: WBC, RBC, haemoglobin, haematocrit, platelets with differential count
    - Coagulation studies (PT/PPT, INR)
    - Chemistry with electrolytes: sodium, potassium, chloride, creatinine, BUN, AST, ALT, alkaline phosphatase, total bilirubin, albumin, total protein, amylase, calcium, phosphate, random glucose, LDH, uric acid, magnesium
- Research Biomarkers
    - CTC collection (Cycles 2, 3, 4, 7, every 3rd cycle thereafter and at EOT)
    - Plasma for DNA, RNA studies (Part A - Cycles 2, 3, 5 and at EOT and Part B - every cycle and EOT)
    - Paxgene miRNA studies (Cycle 3, 5 and at EOT)
    - Urine and plasma metabolomics (Cycles 2, 3 ,6 and at EOT)
    - Urine markers of bone turnover and RNA (Cycle 3, Cycle 6 and at EOT)

11.2.4 Cycles 4, 7, and 10 (continue every 3rd cycle beyond Cycle 10)

In preparation for the study visit, the following assessments should be conducted up to 8 days prior to the indicated visit. Results should be available for review at the Cycle 4, 7, or 10 Day-1 visit and every 3rd cycle beyond Cycle 10.

- CT Scan
- Bone Scan
- DW-MRI (optional)
- Other imaging procedures should be conducted as clinically indicated. If a patient remains on treatment for >2 years, CT/MRI and bone scans should be undertaken every 3rd cycle or as per SOC but at least every 24 weeks.

At the study visit the following assessments should be conducted:

- Brief history and physical examination as clinically indicated

- Vital signs including blood pressure, heart rate, respiratory rate and oral body temperature.
- Weight
- Assessment of ECOG Performance Status
- Local Laboratory Tests
  - FBC: WBC, RBC, haemoglobin, platelets with differential count, chemistry with electrolytes: sodium, potassium, chloride, carbon dioxide, creatinine, BUN, AST, ALT, alkaline phosphatase, total bilirubin, albumin, total protein, amylase, calcium, phosphate, LDH, uric acid, magnesium, random glucose.
  - Coagulation studies (PT/PPT, INR)
  - PSA (every 12 weeks): If patient undergoes a DRE, then PSA must be sampled prior to the DRE
- Concomitant medications listing
- AE evaluation and recording
- Dosing compliance check
- Pain (BFI-SF) and Analgesic Usage Score (each cycle)
- Review of imaging results (CT and bone scan) should be conducted and disease response /progression ascribed at the actual study visit (Cycle 4, 7, or 10 Day 1) unless otherwise indicated.

  If the imaging assessment indicates response (PR or CR by RECIST), a confirmation of response scan should be conducted at the next study visit that is at least 4 weeks after the criteria for response was met (e.g. Cycle 5, 9 or 11 Day 1). Results of the confirmatory scan will be added to the MACRO form “RECIST confirmation of response” of the current visit (Cycle 4, 7 or 10).

  Once the first response observed has been confirmed, there is no need to perform confirmatory scans for subsequent responses observed in the patient.
- Research Biomarkers
  - CTC collection (Cycles 2, 3, 4, 7, every 3rd cycle thereafter and at EOT)
  - Plasma for DNA, RNA studies (Cycles 2, 3, 5 and at EOT) and Part B - every cycle and EOT)
  - Paxgene miRNA studies (Cycle 3, 5 and at EOT)
  - Urine and plasma metabolomics (Cycles 2, 3, 6 and at EOT)
  - Urine markers of bone turnover and RNA (Cycles 3, 6 and at EOT)

In part B, patients allocated to the 300mg dose group would be allowed to dose-escalate to 400mg upon disease progression if clinically indicated. If that happens, patients will continue being followed with the same schedule of events except PSA will be checked at day 1 of every cycle instead of every 3 cycles. Imaging assessment will remain to be scheduled every 3 cycles, with a re-assessment of tumour measurements as per modified RECIST1.1 (appendix E). Patients who required any dose reduction during treatment at the 300mg dose will not be eligible for a dose escalation.

11.2.5 Dose Escalation (300mg group only)

When patients in the 300 mg group progress they should attend a dose escalation visit before crossing over to receive 400mg bd olaparib. The following assessments should be carried out at this visit;

- Physical examination and weight
- Vital signs including blood pressure, heart rate, respiratory rate, and oral or aural body temperature.
- Assessment of ECOG Performance Status
- 12-lead ECG is not mandated but should be done if clinically indicated
- Local Laboratory tests:
  - FBC: WBC, haemoglobin, platelets with differential count
  - Coagulation studies (PT/PPT, INR)
  - Chemistry with electrolytes: sodium, potassium, chloride, creatinine, BUN, AST, ALT, alkaline phosphatase, total bilirubin, albumin, total protein, amylase, calcium, phosphate, random glucose, LDH, uric acid, magnesium.
  - PSA: If patient undergoes a DRE, PSA must be sampled prior to the DRE.
  - Disease Progression Assessment Review of imaging (CT, Bone scan, DW-MRI) results assessment according to modified RECIST1.1 (appendix E) and PCWG2 criteria.
  - Review of PSA results conducted over the course of the study and PSA progression assessment according to PCWG2 criteria
- Concomitant medications listing
- AE evaluation and recording
- Dosing compliance check
- Pain (BFI-SF) and Analgesic Usage Score
- Optional fresh tumour biopsy
- Research Biomarkers
  - Circulating CTC collection
  - Circulating plasma for DNA, RNA studies
  - Paxgene miRNA studies
  - Urine markers of bone turnover and RNA (if not obtained at treatment discontinuation)

11.2.6 End of Treatment (EOT) Visit

Patients should return for an EOT visit 30 days +/- 5 following the patient's last dose of olaparib or prior to starting a new line of anticancer treatment, whichever occurs first. The following assessments should be carried out at the EOT visit:

- Physical examination and weight
- Vital signs including blood pressure, heart rate, respiratory rate, and oral or aural body temperature.

- Assessment of ECOG Performance Status
- 12-lead ECG is not mandated but should be done if clinically indicated
- Local Laboratory tests:
    - FBC: WBC, haemoglobin, platelets with differential count
    - Coagulation studies (PT/PPT, INR)
    - Chemistry with electrolytes: sodium, potassium, chloride, creatinine, BUN, AST, ALT, alkaline phosphatase, total bilirubin, albumin, total protein, amylase, calcium, phosphate, random glucose, LDH, uric acid, magnesium.
    - PSA: If patient undergoes a DRE, PSA must be sampled prior to the DRE.
    - Disease Progression Assessment Review of imaging (CT, Bone scan, DW-MRI) results assessment according to modified RECIST1.1 (appendix E) and PCWG2 criteria - only to be performed for those patients whose reason for treatment discontinuation was not radiological progression.
    - Review of PSA results conducted over the course of the study and PSA progression assessment according to PCWG2 criteria.
- Concomitant medications listing: update listing of all prescriptive and non-prescriptive (over the counter) medications taken since screening. This also includes any nutritional supplements and/or herbal preparations.
- Adverse events
    - AE follow-up to determine if any new or ongoing drug-related AE or any SAE regardless of relationship to study drug exists is required for 30 days post last dose or until patient receives anticancer therapy, whichever comes first.
- Final dosing compliance check if not performed when treatment discontinued.
- Pain (BFI-SF) and Analgesic Usage Score
- Tumour biopsy (this end of study treatment biopsy is optional and can be collected at any point from treatment discontinuation up to the end of treatment visit).
- Research Biomarkers (samples can be obtained within 30 days of treatment discontinuation)
    - Circulating CTC collection
    - Circulating plasma for DNA, RNA studies
    - Paxgene miRNA studies
    - Urine and plasma metabolomics
    - Urine markers of bone turnover and RNA
    - Serum proteomic studies

11.2.7 3 monthly follow up (3 monthly from EOT)

Following treatment completion, patients will be followed for survival. Additional information as to whether they have started any platinum-based chemotherapy, mitoxantrone or another PARP inhibitor will be obtained where feasible. Follow-up data may be collected by a telephone interview.

11.3 Unscheduled Visits

Unscheduled visits and procedures may be conducted at the investigator's discretion for the safety follow-up of the patient. Such visits and procedures must be fully recorded in source documents regarding the purpose and outcome of the visit/assessment. Clinically significant laboratory results from unscheduled visits should be documented in the eCRF.

11.4 Early Discontinuation

Patients will be advised in the written informed consent form that they have the right to withdraw from study drug treatment at any time without prejudice, or may be withdrawn at any time at the discretion of the investigator or the sponsor. Notification of early patient discontinuation from the study and the reason for discontinuation will be made on the protocol deviation form provided. Patients removed from therapy for any reason should continue to be followed for the collection of safety data as outlined in the EOT visit procedures. For patients who refuse or are unable to attend further clinic study visits, telephone contact should be attempted to follow up for adverse events 30 days -/+ 5 days after the last dose of study drug. All serious adverse events will be followed until resolution, until the event has stabilised and/or become chronic, until it has been determined that the event was caused by aetiology other than the study drug, or through 30 days, whichever comes first. Reasonable effort should be made to contact any patient lost to follow-up during the course of the study in order to complete study-related assessments and retrieve any outstanding data and study drug. Such efforts should be documented in the source documents.

11.5 Schedule of Procedures

Table 3a: Part A Schedule of Procedures and Clinic Visits

Procedures & Assessments	Screening		Cycle 1	Cycle 1	Cycle 1	Cycle 2, 3, 5, 6, 8, 9, 11, 12	Cycle 4, 7,10, every 3rd cycle	Cycle 4, 7 ,10, and every 3rd cycle	End of Treatment (EOT) Visit	Follow Up
**Activity day**	Day -28 to Day -1	Day -14 to Day -1	Day 1	Day 8	Day 15	Day 1	Up to 8 days before	Day 1	Day 30 after last dose of drug	3 monthly from EOT
Informed Consent	X									
Medical History[2]		X								
Obtain Archival Tissue[3]	X									
Tumour Biopsy[4]	X			X(8± 21 days)					X (optional)	
Assessment of Symptoms[5]		X	X	X		X		X	X	
Dosing compliance[5]				X		X(every cycle)		X		
Concomitant medication[5]		X	X	X		X		X	X	
Adverse events[5]			X	X		X		X	X	
Physical Examination & Vitals[6]		X	X		X	X		X	X	
Complete Pain Questionnaire[7]		X	X			X(every cycle)		X(every cycle)	X	
12 Lead ECG[8]		X								
Safety Bloods[9]		X	X	X		X		X	X	
Testosterone Level[10]		X								
PSA Levels[11]		X	X					X	X	
**Imaging Assessments**										
Bone Scan[12]	X						X			
DW-MRI (optional)[13]	X						X			
CT Scan[14]	X						X			
Disease Progression Assessment[15]	X							X	X	
Survival & further treatment										X
**Biomarker Tests (patients should be fasted for at least 8 hours prior to blood draw – see 15.3)**										
Buccal Swab[16]		X								
Saliva [17]		X								
Plasma DNA & RNA[18]		X	X	X		X (C2,3,5)			X	
Circulating Tumour Cells[19]	X		X	X	X	X (C2, 3)		X	X	
Blood miRNA[20]		X	X			X (C3,5)			X	
Plasma Proteomics[21]		X	X						X	
Plasma and urine Metabolomics[22]		X	X	X		X (C2,3,6)			X	
Markers of Bone Turnover and RNA[23]	X		X			X(C3,6)			X	

Table 3b: Part B Schedule of Procedures and Clinic Visits

Procedures & Assessments	Pre-screening	Screening		Cycle 1	Cycle 1	Cycle X (Every Cycle)	Cycle 4, 7,10, every 3rd cycle	Dose escalation (300 mg group only)	End of Treatment (EOT) Visit	Follow Up
**Activity day**	**Anytime**	**Day -28 to Day -1**	**Day -14 to Day -1**	**Day 1**	**Day 8**	**Day 1**	**Up to 8 days before**		**Day 30 after last dose of drug**	**3 monthly from EOT**
Informed consent for pre-screening	X									
Confirmation of presence of the biomarker	X									
Informed consent for the main study[1]		X								
Medical History[2]			X							
Obtain Archival Tissue[3]	X	X								
Tumour Biopsy[4]	X (optional)	X (optional)			X(8± 21 days, optional)			X (optional)	X (optional)	
Assessment of Symptoms/adverse events[5]			X	X	X	X		X	X	
Dosing compliance[6]					X	X		X	X	
Concomitant medication[7]			X	X	X	X		X	X	
Physical Examination & Vitals[6]			X	X	X	X		X	X	
Complete Pain Questionnaire[7]			X	X		X		X	X	
12 Lead ECG[8]			X					X[8] (if clinically indicated)	X[8] (if clinically indicated)	
Urinalysis			X							
Safety Bloods[9]			X	X	X	X		X	X	
Testosterone Level[10]			X							
PSA Levels[11]			X	X			X[11]	X[11]	X	
**Imaging Assessments**										
Bone Scan[12]		X					X		X[25]	
DW-MRI (optional)[13]		X					X		X[25]	
CT/MRI Scan[14]		X				(X, if RECIST confirmation[24])	X[24]		X[25]	
Disease Progression Assessment[15]		X				(X, if RECIST confirmation[24])	X[24]		X[25]	
Survival & further treatment										X

Table 3c: Part B Schedule Biomarker Procedures and Pharmacokinetics

Procedures & Assessments	Pre-screening	Screening		Cycle 1	Cycle 1	Cycle 2, 3, 4 and every Cycle onwards	Dose escalation (300mg group only)	End of Treatment (EOT) (Can also be obtained at the time of treatment discontinuation)
Activity day	Anytime	Day -28 to Day -1	Day -14 to Day -1	Day 1	Day 8	Day 1		Day 30 after last dose of drug
Saliva[17]	X		X					
Plasma DNA & RNA[18]			X	X	X	X	X	X
Circulating Tumour Cells[19]		X		X	X	X(C2,3,4 and every 3rd cycle after)	X	X
Blood miRNA[20]			X	X		X (C3,5)	X	X
Plasma Proteomics[21]			X	X				X
Plasma and urine Metabolomics[22]			X	X	X	X (C2,3,6)		X
Markers of Bone Turnover and RNA[23]			X	X		X(C3,6)	X	X

Procedures and Clinic Visits

1. Written informed consent for the main study must be obtained within 28 days period prior to Cycle 1 Day 1.
2. Medical history at screening will detail demographic, family history, co-morbidities, prior treatments for prostate cancer and baseline adverse events.
3. Archival tumour tissue will be obtained for molecular characterisation and eligibility confirmation in Part B. Patients who do not have archival tumour tissue will be required to have a repeat tumour biopsy in order to obtain the required tumour tissue for histological confirmation and for subsequent molecular characterisation. For patients who are not pre-screened in TOPARP the archival tissue should be retrieved in the screening period.
4. Fresh tumour biopsies are a mandatory component of Part A and will be obtained prior to commencing treatment (during the screening period) and post treatment (at any point from Day 8 to Day 28 of Cycle 1). The third biopsy at the time of treatment discontinuation is optional. All fresh tumour biopsies are optional for part B. However, fresh tumour biopsies are also preferred for Part B and carry a higher chance of enabling patient eligibility.
5. At each clinic review, symptoms, concomitant medication, treatment compliance and adverse events will be assessed. Adverse events will be assigned a grade according to the CTCAE version 4.02 and causality. Adverse Event follow-up is required for 30 +/-5 days post last dose of treatment to determine if any new or on-going drug related AE or any SAE regardless of relationship to drug persists.
6. Includes physical examination, blood pressure, heart rate, respiratory rate, oral body temperature, and weight. Height will only be measured at the screening visit. A full physical examination will be performed at screening, Cycle 1 Day 1 and at the EOT visit. At all other clinic reviews physical examinations will be performed as clinically indicated.
7. A Brief Pain Inventory Questionnaire will be given to the patient to fill in at screening and at the beginning of each cycle. The Brief Pain Inventory Score will be annotated in the patients CRF.
8. ECG will only be performed at the screening visit. ECGs should be obtained with the subject in the supine resting position for several minutes. Abnormal ECG related to electrolyte abnormalities should be repeated following the correction of the electrolyte abnormalities. ECGs will only be repeated beyond screening if clinically indicated.
9. A full array of safety bloods including the full blood count, blood chemistry, liver function test, clotting profile, random glucose, amylase, etc. will be obtained and checked at the beginning of each cycle of treatment.
10. Serum testosterone level will be checked during the screening period to ensure a systemically castrate state [testosterone levels of < 50 ng/dL (< 2.0 nM)]. Testing for serum testosterone, PSA and routine safety bloods will be performed at the local site laboratory.
11. PSA testing will be done at screening and every 12 weeks (Day 1) while on treatment. PSA responses by PCWG2 criteria will need to be confirmed 4 or more weeks later. **For patients in Part B allocated to the 300mg group that pursue dose-escalation upon progression, PSA will be checked at day 1 of every cycle after the dose escalation.**
12. A bone scan will be obtained at baseline and after every 3rd cycle of treatment. New bone deposits will need to be confirmed with a repeat bone scan 6 or more weeks later (after 2 years on treatment, scans every 3rd cycle or as per SOC but at least every 24 weeks).
13. DW-MRI is optional and will be performed at baseline (screening) and every 12 weeks thereafter.
14. CT scan will be obtained at baseline and after every 3 cycles of treatment (after 2 years on treatment, scans every 3rd cycle or as per SOC but at least every 24 weeks).. If a status of partial or complete response is made, changes in tumour measurements must be confirmed by repeat assessments that should be performed no less than 4 weeks after the criteria for response are first met.
15. Disease progress assessment will be made after every 3rd cycle of treatment at the clinic visits. Progression by bone scan will need to be confirmed by a second bone scan ≥ 6 weeks later, as per guidance in Appendix D.
16. A buccal swab for DNA analysis should be collected at the indicated time point (Part A only).
17. Saliva for DNA analysis should be collected using an Oragene Kit. For Part B it can be collected either at pre-screening or screening.
18. Plasma for circulating plasma DNA and RNA will be collected at the indicated time points.
19. Blood for CTC analysis should be collected at the indicated time points.
20. Blood samples for whole blood miRNA analysis will be collected at the indicated time points.
21. Blood samples will be collected for proteomics profiling at the indicated time points.
22. Urine and plasma metabolomics profiles will be evaluated both at baseline and during treatment at the indicated time points. Blood samples will be collected in -5min pre dose and 2 hour post dose. A 24 hour urine collection will be collected.
23. Urine collected after the void of the first morning sample will be collected at the indicated timepoints to assess markers of bone turnover such as telopeptides.
24. If a cycle's imaging assessment by CT/MRI indicates PR or CR by RECIST then this must be confirmed with a repeat imaging assessment by CT/MRI conducted at the next study visit that is at least 4 weeks after the criteria for response was met (e.g. if response was observed at Cycle 4, the confirmatory scan would be performed at Cycle 5).
25. Radiological assessments at the End of Treatment Visit are only to be performed to those patients whose reason for treatment discontinuation was not radiological progression and who had no assessments performed within 30 days of the visit.

Patients should be fasted for 8 hours prior to all blood samples on the days where metabolomic samples are collected (screening, C1D1, C1D8, C2D1, C3D1, C6D1 and EOT).

12 TRIAL TREATMENT

12.1 Drug Manufacturer

Olaparib tablets will be manufactured and provided free of charge by AstraZeneca to participating centres. AstraZeneca will manufacture to Good Manufacturing Practice (GMP) and UK Legislation.

12.2 Presentation / Formulation of Olaparib

Olaparib is presented for oral administration as a green, oval bi-convex film-coated tablet containing 100 mg and 150mg (in the instance of dose reduction to 250mg) of drug substance.

12.3 Drug, Dose and Schedule

The dose of 400 mg olaparib, twice daily orally, continuous dosing has been selected for this Part A based on the pharmacokinetic (PK), pharmacodynamic (PD) and clinical efficacy data form the Phase I-II studies including an ongoing bioavailability study (10, 11, 49, 50, 61, 62).

In Part B, a second cohort receiving 300mg twice daily will be included to assess the optimal dosage in CRPC.

The olaparib dose should NOT be adjusted according to the patient's body weight, even for very heavy or light patients. Patients will be given sufficient supplies for 1 cycle (28 days) of treatment. If a patient is not able to attend the clinic review due to a public holiday falling on the 28th day of the cycle or for other unforeseen circumstances consideration should be given to ensure the patient has sufficient drug supplies.

Patients should be advised to withhold study drug on the morning of their clinic appointments for trial assessments.

12.4 Instructions for Administration of Olaparib

Patients should swallow the olaparib tablets whole with a glass of water in the morning and evening at the same times each day to ensure a dose interval of approximately 12 hours. All doses should be taken with approximately 240mL of water (1 glass of water) and can be taken with a light meal. The tablets should be swallowed whole and not chewed, crushed, dissolved or divided. If vomiting occurs shortly after the olaparib is swallowed, the dose should only be replaced if all of the intact tablets can be seen and counted. Should any patient enrolled on the trial miss a scheduled dose for whatever reason (e.g. as a result of forgetting to take the dose of olaparib), he will be allowed to take the scheduled dose up to a maximum of 2 hours after the scheduled dose time. If greater than 2 hours after the scheduled dose time, the missed dose is not to be taken and the patient should take their allotted dose at the next scheduled time.

12.5 Olaparib Treatment Compliance

A member of the Investigative site's study team will query the patient for treatment compliance at day 1 of each new cycle of treatment. All patients must return their bottles of olaparib at the appropriate scheduled visit, when new bottles will be dispensed. Compliance will be assessed by capsule count and questioning by the study nurse and the information will be recorded.

12.6 Duration of Therapy

Patients may continue with olaparib treatment as long as they remain free from intolerable toxicity, in the Investigator's opinion, are receiving clinical benefit and do not meet any discontinuation criteria (see Section 10.9 for discontinuation criteria).

12.7 Supportive Care Guidelines

Based upon experience from Phase I and II studies, olaparib is generally well tolerated. The safety concerns are primarily related to the risk of myelosupression. Supportive care should be provided therapeutically and prophylactically as deemed necessary by the treating physician including but not limited to the items outlined below:

Gastrointestinal symptoms

Prophylactic anti-emetics and/or anti-diarrhoeals will not routinely be given. Should a patient develop nausea, vomiting and/or diarrhoea that, in the Investigator's opinion, is considered to be related to olaparib, then appropriate prophylactic treatment may be initiated. Nausea and vomiting should be treated aggressively, and strong consideration should be given to the administration of prophylactic antiemetic therapy according to standard institutional practice. Patients should be strongly encouraged to maintain liberal oral fluid intake.

Myelosupression

Routine prophylaxis with granulocyte colony-stimulating factor (G-CSF)/granulocyte-macrophage colony-stimulating factor (GM-CSF) and erythropoietin is not recommended. Nevertheless, use is permitted at the Investigator's discretion and according to local hospital guidelines. The occurrence of leukopenia and/or anaemia should be managed as deemed appropriate by the investigator. The use growth factors must be discontinued once the AE has recovered to CTCAE grade 1 or better. They may be resumed, if necessary, if leukopenia/anaemia develops again and discontinued once it recovers. The reason(s) for the use, doses and dates of treatment should be recorded in the patient's medical records and appropriate section of the eCRF. Checking haematinics levels (Iron, B12 and folate) and providing supplementation if these are found to be low is advocated. Transfusions may be given as clinically indicated for the treatment of anaemia and thrombocytopenia.

Symptoms Related to Castration

Treatment with androgens, estrogens and progestin to control hot flushes is not allowed. Antidepressants that do not interfere with P450 enzyme function are permitted for the management of hot flashes. Long-term androgen deprivation may lead to metabolic diseases, such as glucose intolerance, hypercholesterolemia, and osteoporosis,

appropriate medication may be prescribed to manage these conditions. All such AEs need to be recorded.

12.8 Guidelines for Management of Toxicity and Dose- Modification

Based on Phase I and Phase II studies, olaparib is generally well tolerated. Safety concerns are mainly related to the possibility of myelosupression.

Any toxicity observed during the course of the study may be managed by a dose interruption if deemed appropriate by the Investigator. Treatment must be interrupted if any clinically significant NCI-CTCAE grade 3 or 4 adverse event occurs which the Investigator considers to be related to administration of olaparib.

Repeat dose interruptions are allowed as required, for a maximum of 42 days on each occasion. Olaparib must be interrupted until the patient recovers completely or the toxicity reverts to National Cancer Institute (NCI) Common Terminology Criteria for Adverse Events (CTCAE version 4.02) grade 1 or less. When the toxicity is appropriately resolved, the patient can resume treatment with olaparib with a dose reduction according to Table 3 if a treatment break of ≥ 14 days was required for the resolution of the toxicity. If the event recurs with the same severity following olaparib re-challenge, treatment should be interrupted again, and on resolution, a further dose reduction made. A maximum of 3 dose reductions will be allowed for patients allocated to receive 400mg and 2 dose reductions for patients allocated to receive 300mg. Regular 7-day drug holidays will also be permitted if myelosuppression is an issue at these doses.

If, on re-starting treatment following either 2 or 3 dose reductions, the event continues to occur at the same severity, treatment should be discontinued and the patient withdrawn from study. If the toxicity has not resolved to at least grade 1 during the maximum 42-day period, and/or the patient has already undergone a maximum of 2 or 3 dose reductions already, the patient must permanently discontinue treatment with olaparib. Once the dose of olaparib has been reduced under no account should it be re-escalated.

The dose of olaparib should not be reduced for grade 3-4 gastrointestinal adverse events that have not been adequately managed with appropriate prophylactic measures. In all cases where the olaparib dose has been interrupted or the patient discontinued due to unusual or unusually severe toxicity considered related to olaparib, the Investigator must contact the Chief Investigator and CTSU trial management team. All dose modifications and interruptions (including any missed doses), and the reasons for the modifications/interruptions are to be recorded in the eCRF.

Olaparib should be temporarily withheld in the event of:

- Grade 4 neutropenia or leukopaenia
- Grade 2-4 neutropenia with or leukopaenia infection
- Platelet count lower than 50 x 10^9/L
- Non-haematological toxicity grade 3 or greater, except nausea, vomiting or diarrhoea and fatigue associated with suboptimal pre-medication and/or management OR transient and clinically insignificant biochemical blood results.
- Aspartate transaminase (AST) or alanine transaminase (ALT) elevations grade 3 or higher for more than 7 days.

- New or worsening pulmonary symptoms or radiological abnormality occurs which are suggestive of pneumonitis
- Any other toxicity that, in the view of the principal investigator and the medical monitor, represents a clinically significant hazard to the patient until resolution of toxicity to no more than grade 1.

Starting Dose	Olaparib Dose	Olaparib dose
	400mg BID	300 mg BID
First dose reduction due to NCI-CTC grade 3 or 4 treatment-related Serious Adverse Events / Adverse Events (excluding inadequately managed gastrointestinal symptoms)	300mg BID	250 mg BID
Recurrent episode of NCI-CTC grade 3 or 4 event despite one previous dose reduction (excluding inadequately managed gastrointestinal symptoms)	250mg BID	200 mg BID
Recurrent episode of NCI-CTC grade 3 or 4 event despite two previous dose reductions(excluding inadequately managed gastrointestinal symptoms)	200mg BID	Not applicable

Table 4: Summary Guidelines for Dose Reductions

Management of prolonged haematological toxicities including anaemia, neutropenia, leukopaenia or thrombocytopenia whilst on study treatment

Adverse event of neutropenia and leukopenia should be managed as deemed appropriate by the investigator with close follow up and interruption of study drug if CTC grade 3 or worse neutropenia or leukopaenia occurs.

For management of drug-induced anaemia, blood transfusions should be considered to maintain haemoglobin levels to 9g/dl and above.

If any study treatment is interrupted/ delayed because of one or more of the following:

- ≥2 week interruption/delay in study treatment due to CTC grade >2 neutropenia
- ≥2 week interruption/delay in study treatment due to CTC grade >2 thrombocytopenia
- ≥2 week interruption/delay in study treatment due to CTC grade >2 leukopaenia
- ≥2 week interruption/delay in study treatment due to CTC grade >2 anaemia and or development of blood transfusion dependence

Weekly blood counts should be performed during the study treatment interruption/delay. If the levels have still not recovered to CTC Grade ≤1 after 42 days of dose interruption, the patient should be referred to a haematologist for further investigations. Bone marrow analysis or blood cytogenetic analysis should be considered at this stage according to standard haematological practice.

Bone Marrow or blood cytogenetic analysis:

If a bone marrow analysis is carried out to evaluate cytopenia it should include an aspirate for cellular morphology, cytogenetic analysis and flow cytometry, and a core biopsy for

bone marrow cellularity. If it is not possible to conduct cytogenetic analysis or flow cytometry on the bone marrow aspirate, then attempts should be made to carry out the tests on a blood sample. Development of myelodysplastic syndrome should be reported as an SAE and full reports must be provided.

Management of new or worsening pulmonary symptoms

If new or worsening pulmonary symptoms (e.g. dyspnoea) or radiological abnormality occurs, an interruption in olaparib dosing is recommended and a diagnostic workup (including a high resolution CT scan) should be performed, to exclude pneumonitis. Following investigation, if no evidence of abnormality is observed on CT imaging and symptoms resolve, then olaparib treatment can be restarted, if deemed appropriate by the investigator. If significant pulmonary abnormalities are identified, these need to be discussed with the Chief Investigator and CTSU trial management team. All dose reductions and interruptions (including any missed doses), and the reasons for the reductions/interruptions are to be recorded.

12.9 Packaging and Labelling

AstraZeneca have employed Fisher Clinical Services to coordinate IMP packaging, labelling and distribution for this trial. Olaparib will be supplied in High Density Polyethylene containers containing desiccant. Bottles are secured with a child-resistant closure; induction-sealed membranes provide tamper evidence. Olaparib will be provided to each site in labelled bottles. Each bottle will have a label permanently affixed to the outside and will be labelled in accordance with Good Manufacturing Practice and local regulations, stating that the material is for clinical trial/investigational use only and should be kept out of reach of children. Labels will refer to Olaparib as AZD2281 and will include blank lines for quantity of tablets to be taken, patient trial ID and date of dispensing.

12.10 Drug Storage

Drug Storage by Pharmacy

All study medication must be kept in a secure place under appropriate storage conditions (at temperatures less than 30ºC and protected from light). Study medication should not be stored under refrigerated conditions. Additional information is provided in the olaparib Investigator's Brochure. Any deviations from the recommended storage conditions should be immediately reported to the sponsor and the use of olaparib interrupted until authorisation for its continued use has been given by AstraZeneca and the sponsor.

Storage Requirements for the Patient

Olaparib must be stored in the bottle with the cap tightly on at room temperature. Study medication should not be refrigerated. Patients should be advised to keep all medication out of the sight and reach of young children.

12.11 Distribution of Drugs to Site

No drug will be distributed to participating centres unless ICR-CTSU is satisfied that the required approvals, agreements and initiation procedures are complete.

Participating pharmacy departments should contact Fisher Clinical Services via the ICR-CTSU trial management team to request drug supplies following patient registration.

Records must be kept for all deliveries and a copy of the order / delivery note placed in the Site Pharmacy File and kept within the pharmacy department as in routine practice. At the end of the trial, it must be possible to reconcile supply and usage of all stock. Account must be given to any discrepancies and certificates of delivery and return must be signed.

12.12 Pharmacy Responsibilities and Drug Accountability

Labelling:

Labelling will be the responsibility of Fisher Clinical Services and will be compliant with Annex 13 of the Good Manufacturing Guidelines (GMP) and all applicable local regulatory requirement. Each hospital pharmacist must also insert the name of the Principal Investigator (PI) or hospital specific code by which the PI can be identified, together with the Trial ID and patient's name.

The local pharmacy department must designate a responsible person for ensuring that:

- The study drug is handled and stored safely and properly
- The study drug is labelled for each patient in accordance with Annex 13 of the EU Good Manufacturing Practice (GMP) Guidelines
- The study drug is dispensed only to trial patients and in accordance with the protocol and under the guidelines of the site's dispensing standard operating procedure
- Full accountability is maintained for the study drug
- Patients should be instructed to return unused olaparib, preferably in their original packs. Olaparib which has been dispensed to a patient and returned unused must not be re dispensed to a different patient
- There is a sufficient supply of study drug for patients' continued treatment, and in a timely manner, contact ICR-CTSU for re-supply of stock
- Study drug expiry dates are monitored and are used in order of expiry date order i.e. earliest expiry first
- All unused drugs will be accounted for and retained until advised otherwise. Unused and returned study drug may be destroyed locally in accordance with hospital protocol and following written instructions from the sponsor

Patient cards

Small wallet sized cards will be produced by ICR-CTSU on request by the participating centre. Each card will state:

- the name of the participating centre
- that the patient is participating in the TOPARP trial
- that the patient is taking olaparib

- an emergency contact number

12.13 Concurrent Medications

The concurrent administration of other anticancer therapies, including cytotoxic, hormonal (except LHRH agonists/antagonists) or immunotherapy, is prohibited during study treatment. The use of any natural/herbal products or other “folk remedies” should be discouraged but use of these products, as well as use of all vitamins, nutritional supplements and all other concomitant medications must be recorded.

Olaparib is an investigational drug for which no data on *in vivo* interactions are currently available. Based on *in vitro* data and clinical exposure data, olaparib is considered unlikely to cause clinically significant drug interactions through inhibition or induction of cytochrome P450 enzyme activity. *In vitro* data have, however, also shown that the principal enzyme responsible for the formation of the 3 main metabolites of olaparib is CYP3A4 and consequently, although the contribution of metabolic clearance to total drug clearance in man is currently unknown, to ensure patient safety, the following potent inhibitors of CYP3A4 must not be used during this study for any patient receiving olaparib. Whilst this is not an exhaustive list of inhibitors, it covers the known potent inhibitors which have most often previously been reported to be associated with clinically significant drug interactions:

- ketoconazole, itraconazole, ritonavir, indinavir, saquinavir, telithromycin, clarithromycin, nelfanavir, boceprevir and telaprevir
- ciprofloxacin, erythromycin, diltiazem, fluconazole, verapamil)

For patients taking any of the above, the required wash-out periods prior to starting olaparib is one week. It is not recommended for patients to consume grapefruit juice while on olaparib therapy.

In addition, to avoid potential reductions in exposure due to drug interactions, the exclusion of the following P450 inducers is recommended:

- Phenytoin, rifampicin, rifapentin, reifabutin, carbamazepine and phenobarbitone, nevirapine, modafinil, enzalutamide, bosentan, efavirenz and St John’s Wort (Hypericum perforatum)
- For patients taking any of the above, the required wash-out periods prior to commencing olaparib are:
- Phenobarbitone & enzalutamide - 5 weeks; any of the others - 3 weeks.

If use of any potent inducers or inhibitors of CYP3A4 are considered necessary for the patient’s safety and welfare, the Investigator must contact the ICR-CTSU and a decision to allow the patient to be enrolled in the study will be made on a case-by-case basis by the Chief Investigator.

P-gp inhibitors

It is possible that co-administration of P-gp inhibitors (eg amiodarone, azithromycin) may increase exposure to olaparib. Caution should therefore be observed.

Effect of olaparib on other drugs

Based on limited in vitro data, olaparib may increase the exposure to substrates of CYP3A4, P-gp, OATP1B1, OCT1, OCT2, OAT3, MATE1 and MATE2K.

Based on limited in vitro data, olaparib may reduce the exposure to substrates of CYP3A4, CYP1A2, 2B6, 2C9, 2C19 and P-gp.

The efficacy of hormonal contraceptives may be reduced if co administered with olaparib.

Caution should therefore be observed if substrates of these isoenzymes or transporter proteins are co-administered. Examples of substrates include:

- CYP3A4 – hormonal contraceptive, simvastatin, cisapride, cyclosporine, ergot alkaloids, fentanyl, pimozide, sirolim
- CYP1A2 – duloxetine, melatonin
- CYP2B6 – bupropion, efavirenz
- CYP2C9 – warfarin
- CYP2C19 - lansoprazole, omeprazole, S-mephenytoin
- P-gp - simvastatin, pravastatin, digoxin, dabigatran, colchicine
- OATP1B1 - bosentan, glibenclamide, repaglinide, statins and valsartan
- OCT1, MATE1, MATE2K – metformin
- OCT2 - serum creatinine
- OAT3 -furosemide, methotrexate

If at any time an investigator suspects a drug-drug interaction due to olaparib, an AE report should be completed and the study sponsor informed.

Permitted Concomitant Therapy

- Leuteinizing hormone-releasing hormone (LHRH) agonists/antagonists to maintain a testosterone level <50g/dL should be administered in patients who have not undergone an orchidectomy.
- Conventional multi-vitamins and minerals.
- Stable chronic non-immunosuppressive systemic doses of corticosteroids up to a dose of prednisolone 5mg BID or equivalent so long as the treatment was initiated prior to commencing the trial medication with evidence of progression on the dose of steroids. Higher doses of steroids are permitted if clinically indicated for acute medical conditions.
- Initiating bisphosphonate therapy or adjusting bisphosphonate dose/regimen within 30 days prior to Cycle 1 Day 1 is prohibited. Patients on a stable bisphosphonate regimen are eligible and may continue.
- Anticoagulant Therapy: Patients who are taking warfarin may participate in this trial however, it is recommended that prothrombin time (International Normalised Ratio [INR] and activated partial thromboplastin time [APTT]) be monitored carefully at least once per week for the first month, then monthly if the INR is stable. Subcutaneous heparin is permitted and is preferable given the potential for myelosupression.
- **Palliative radiotherapy**: may be used for treatment of pain at the site of bony metastases that were present at baseline, providing the Investigator does not feel that this is an indication for the patient to come off treatment due to disease progression.

Full details of all of these treatments are recorded in the patient’s notes and appropriate section of the eCRF.

Therapy considered necessary for the subject's welfare and which is believed not to interfere with the study medication may be given at the discretion of the investigator. If concurrent medications may have an effect on study outcomes, a statement to the effect that these medications should be administered in dosages that remain constant throughout the course of the trial should be included. All medications (prescriptions or over-the-counter medications), the reason(s) for the use, doses and dates of treatment should be recorded in the patient’s medical records.

13 PHARMACOVIGILANCE

13.1 Definitions

Adverse Event (AE): any untoward medical occurrence in a patient or clinical trial subject administered a medicinal product and which does not necessarily have a causal relationship with this treatment. An AE can therefore be any unfavourable and unintended sign (including an abnormal laboratory finding), symptom, or disease associated with the use of a study drug, whether or not considered related to the study drug.

Adverse Reaction (AR): all untoward and unintended responses to the study drug related to any dose administered. All AEs judged by either the reporting investigator or the sponsor as having reasonable causal relationship to a medicinal product qualify as adverse reactions i.e. an AR is possibly, probably or definitely related to the study drug. The expression reasonable causal relationship means to convey in general that there is evidence or argument to suggest a causal relationship.

Serious Adverse Event (SAE) or Serious Adverse Reaction (SAR): Any untoward medical occurrence or effect which occurs from consent to the main study until 30 days after the last dose of study drug and:

- **results in death:** the patient's death is suspected as being a direct outcome of the AE.
- **is life-threatening:** refers to an event in which the subject was at risk of death at the time of the event. It also refers to an event that would result in death with the continued use of the product; it does not refer to an event which hypothetically might have caused death if it were more severe.
- **requires hospitalisation, or prolongation of existing inpatient hospitalisation:** admission to hospital overnight or prolongation of a stay in hospital was necessary as a result of the AE.
- **results in persistent or significant disability or incapacity:** the AE results in a significant or persistent change, impairment, damage or disruption in the patient's body function/structure, physical activities or quality of life.
- **results in a congenital anomaly or birth defect**

Hospitalisation meeting the definition for "serious" is any inpatient hospital admission that includes a minimum of an overnight stay in a health care facility. Inpatient admission does not include: rehabilitation facilities, hospice facilities, skilled nursing facilities, nursing homes, routine emergency room admissions, same day surgeries (as outpatient/same day/ambulatory procedures), or social admission (e.g., subject has no place to sleep). Outpatient treatment in an emergency room is not itself an SAE, although the reasons for it may be. Hospital admissions/surgical procedures planned for a pre-existing condition before a patient is randomised to the study are not considered SAEs, unless the illness/disease deteriorates in an unexpected way during the study.

Medical judgement should be exercised in deciding whether other AE/ARs are serious. Important AE/ARs that are not immediately life-threatening or do not result in death or

hospitalisation but may jeopardise the subject or may require intervention to prevent one of the other outcomes listed in the definition above, should also be considered serious.

N.B. progressive disease and death due to disease are not considered SAEs but should be reported on the relevant forms.

Suspected Unexpected Serious Adverse Reaction (SUSAR): Any serious adverse event with a suspected relationship to study drug that is not listed on the Investigator Brochure (IB) and in the opinion of the Chief Investigator, is unexpected.

13.2 Clinical & Laboratory Adverse Events

AEs Based on Symptoms

All AEs spontaneously reported by the subject or reported in response to the open question from the study personnel: "Have you had any health problems since the previous visit?" or revealed by observation will be collected and recorded in the eCRF. When collecting AEs, the recording of diagnoses is preferred (when possible) to recording a list of signs and symptoms. However, if a diagnosis is known and there are other signs or symptoms that are not generally part of the diagnosis, the diagnosis and each sign or symptom will be recorded separately.

AEs Based on Examinations & Laboratory Tests

Deterioration as compared to baseline in protocol-mandated laboratory values, vital signs and other safety variables will only be reported as AEs if they fulfil any of the SAE criteria or are the reason for stopping treatment with the investigational product. If deterioration in a laboratory value/vital sign is associated with clinical signs and symptoms, the sign/symptom will be reported as an AE and the associated laboratory result/vital sign will be considered as additional information. Wherever possible the reporting Investigator should use the clinical, rather than the laboratory term (e.g., anaemia versus low haemoglobin value).
The criteria for determining whether an abnormal laboratory test result should be reported as an adverse event are as follows:

- Test result is associated with accompanying symptoms.
- Test result requires additional diagnostic testing or medical/surgical intervention (merely repeating an abnormal test, in the absence of any of the above conditions, does not meet criteria for reporting as an AE).
- Test result leads to a change in study dosing or discontinuation from the study, significant additional concomitant drug treatment or other therapy.
- Test result leads to any of the outcomes included in the definition of a SAE.
- Test result is considered to be an adverse event by the investigator or sponsor.
- Any abnormal test result that is determined to be an error does not require reporting as an adverse event, even if it did meet one of the above conditions except for bullet point 4.
- Relevant clinically significant laboratory results should be recorded in the patient's eCRF.

All clinically important abnormal laboratory tests occurring during the study will be repeated at appropriate intervals until they return either to baseline or to a level deemed acceptable by the investigator and the sponsor (or his/her designated representative), or until a diagnosis that explains them is made.

Olaparib adverse events of special interest

Adverse events of special interest [AESI] are events of scientific and medical interest specific to the further understanding of olaparib's safety profile and require close monitoring. An AESI may be serious or non-serious. AESIs for olaparib are the Important Potential Risks of MDS/AML, new primary malignancy (other than MDS/AML) and pneumonitis. ANY event of MDS/AML, new primary malignancy, or pneumonitis should be reported to ICR-CTSU whether it is considered a non-serious AE (eg non-melanoma skin cancer) or SAE, and regardless of investigator's assessment of causality or knowledge of the treatment arm. ICR-CTSU will forward such events to AstraZeneca Patient Safety. A questionnaire may be sent to any investigator reporting an AESI, as an aid to provide further detailed information on the event. During the study there may be other events identified as AESIs that require the use of a questionnaire to help characterise the event and gain a better understanding regarding the relationship between the event and study treatment.

13.3 Causality

Many adverse events that occur in this trial, whether they are serious or not, will be known treatment related toxicities. The Principal Investigator is responsible for the assessment of causality of serious adverse events (see definitions of causality table).

If there is any doubt about the causality of an event, the investigator should inform ICR-CTSU who will notify the Chief Investigator. The drug manufacturer and/or other clinicians may also be asked for advice.

13.4 Severity of Adverse and Serious Adverse Events

Adverse event (AE) severity is a clinical determination of the intensity of an AE and SAEs. The severity assessment for a clinical AE should be completed using the NCI Common Terminology Criteria for Adverse Events (CTCAE v4.02). Any AE not listed in the CTCAE will be graded as follows:

Table 5: Severity of Event

Grade	Definition	Description
**1**	Mild adverse event	Does not interfere with the patient's usual function [awareness of symptoms or signs, but easily tolerated (acceptable)].
**2**	Moderate adverse event	Interferes to some extent with the patient's usual function [enough discomfort to interfere with the usual activity (disturbing)].

**3**	Severe and undesirable adverse event	Interferes significantly with the patient's usual function [incapacity to work or to do usual activities (unacceptable)].
**4**	Life-threatening or disabling adverse event	Results in risk of death, organ damage or permanent disability (unacceptable).
**5**	Death related to adverse event. Relationship to Study Drug	Event has a fatal outcome.

Causality of Event

A determination should be made by the investigator regarding the relationship (if any) between an AE and the study drug. A causal relationship is present if there is a reasonable possibility that the AE may have been caused by the study drug. The following definitions should be considered when evaluating the relationship of AEs and SAEs to the study.

Table 6: Definitions for Causality

**Relationship**	**Description**
**Unrelated**	There is no evidence of any causal relationship with the trial drug
**Unlikely**	There is little evidence to suggest there is a causal relationship (e.g. the event did not occur within a reasonable time after administration of the trial medication). There is another reasonable explanation for the event (e.g. the patient's clinical condition, other concomitant treatment).
**Possible**	There is some evidence to suggest a causal relationship (e.g. because the event occurs within a reasonable time after administration of the trial medication). However, the influence of other factors may have contributed to the event (e.g. the patient's clinical condition, other concomitant treatments).
**Probable**	There is evidence to suggest a causal relationship, and the influence of other factors is unlikely.
**Definitely**	There is clear evidence to suggest a causal relationship, and other possible contributing factors can be ruled out.
**Not assessable**	There is insufficient or incomplete evidence to make a clinical judgement of the causal relationship.

The expectedness of a SAR related to the study drug should be assessed by the Chief Investigator in accordance with the information provided in the IB for olaparib.

Expected SARs should be reported as per the SAE reporting guidelines. SAEs should be reported up to 30 days after the patient's last dose of study medication.

A summary of 'expected' adverse reactions as stated in the IB is listed below:

Table 7: Expected Adverse Reactions (Olaparib Monotherapy)

**Frequency of Adverse Events**	**Very common**	**Common**	**Rare**

Grade (G)	**Mild to moderate CTCAE:G1-2**	**Mild to moderate CTCAE:G1-2**	**Mild to moderate CTCAE:G1-2**
**Adverse Events**	Nausea	Anaemia	Lymphopenia
	Vomiting	Headache	Tachycardia
	Fatigue	Dyspepsia	Thrombocytopenia
		Dizziness	Neutropenia
		Asthenia	
		Decreased appetite	
		Dysgeusia	
		Constipation	

13.5 Procedures for Recording and Reporting Adverse Events

13.5.1 Reporting Serious Adverse Events (SAEs) to ICR-CTSU

Any SAE that occurs from the time of screening and up to 30 days following the last dose of olaparib must be reported, whether or not considered causally related to the study treatment. For patients that do not enter the trial SAEs should be reported from screening until a decision is made the patient will not enter the trial. Any SAEs reported in this period should be followed until clinical recovery is complete and laboratory results have returned to normal, or until disease has stabilised.

All SAEs should be reported to ICR-CTSU, within 24 hours of the study team becoming aware of the event, by completing the trial specific SAE forms and faxing to:

The ICR-CTSU safety desk

Fax no: 0208 722 4368

For the attention of the TOPARP Trial team

Investigators or other site personnel must ensure that:

- All SAE forms must be completed, signed and dated by the Principal Investigator or designated representative.
- All SAE reports are supported by documentation in the patient's medical records. Obtain and maintain in his files all pertinent medical records, information, and medical judgments from colleagues who assisted in the treatment and follow-up of the patient.
- All patients with a serious adverse event must be followed up and the outcomes reported in a follow up severe adverse event report form to the ICR CTSU.
- In the event of fatal or life-threatening AEs where important or relevant information is missing, active follow-up must undertaken immediately.

13.6 Review of Serious Adverse Events (SAEs) or Reactions (SARs)

Reported events / reactions using the SAE forms will be assessed by the Chief Investigator (or designated representative) for causality and expectedness.

NB. The Chief Investigator cannot down grade the Principal Investigator's assessment of causality.

SAEs assessed as having a causal relationship to study drug and as being unexpected (SUSARs) will undergo expedited reporting to the relevant authorities by ICR-CTSU (see figure 4 for SAE reporting).

Centres should respond as soon as possible to requests from the Chief Investigator or designated representative (via ICR-CTSU) for further information that may be required for final assessment of the SAE.

13.7 Expedited Reporting of SUSARs

If an SAE is identified as being a SUSAR by the Chief Investigator, and is fatal or life threatening, it will be reported by ICR-CTSU to the MHRA, the Main REC, the sponsor institutions and AstraZeneca within 7 days of being notified of the event.

If an SAE is identified as a SUSAR by the Chief Investigator, and is not fatal or life threatening, it will be reported by ICR-CTSU to the MHRA, the Main REC, the sponsor institutions and AstraZeneca within 15 days of ICR-CTSU being notified of the event.

ICR-CTSU will report any additional relevant information to the MHRA, Main REC, the sponsor institutions and AstraZeneca as soon as possible, or within 8 days of the initial report of a fatal/life threatening SUSAR.

The Principal Investigators at all actively recruiting centres will be informed of any SUSARs occurring within the trial at regular intervals.

13.8 Follow up of Serious Adverse Events

Centres should continue to follow up SAEs until clinical recovery is complete and laboratory results have returned to normal, or until disease has stabilised. Information on outcome of the SAE should be completed on the relevant part of the original SAE and faxed to ICR-CTSU as soon as the Principal Investigator becomes aware.

NB Any male patient who fathers a child whilst on study medication must be followed up and the outcome reported to ICR-CTSU who will then forward the details to AstraZeneca.

13.9 Annual Reporting of Serious Adverse Reactions

An annual report will be provided to the MHRA and the Main REC, by ICR-CTSU and copied to the Co-sponsors, on the anniversary of the date when the Clinical Trials

Authorisation (CTA) was obtained. This will include all related events reported on SAE forms, and a report from the Independent Data Monitoring Committee (IDMC).

Figure 4: Flow diagram for SAE reporting, and action following report

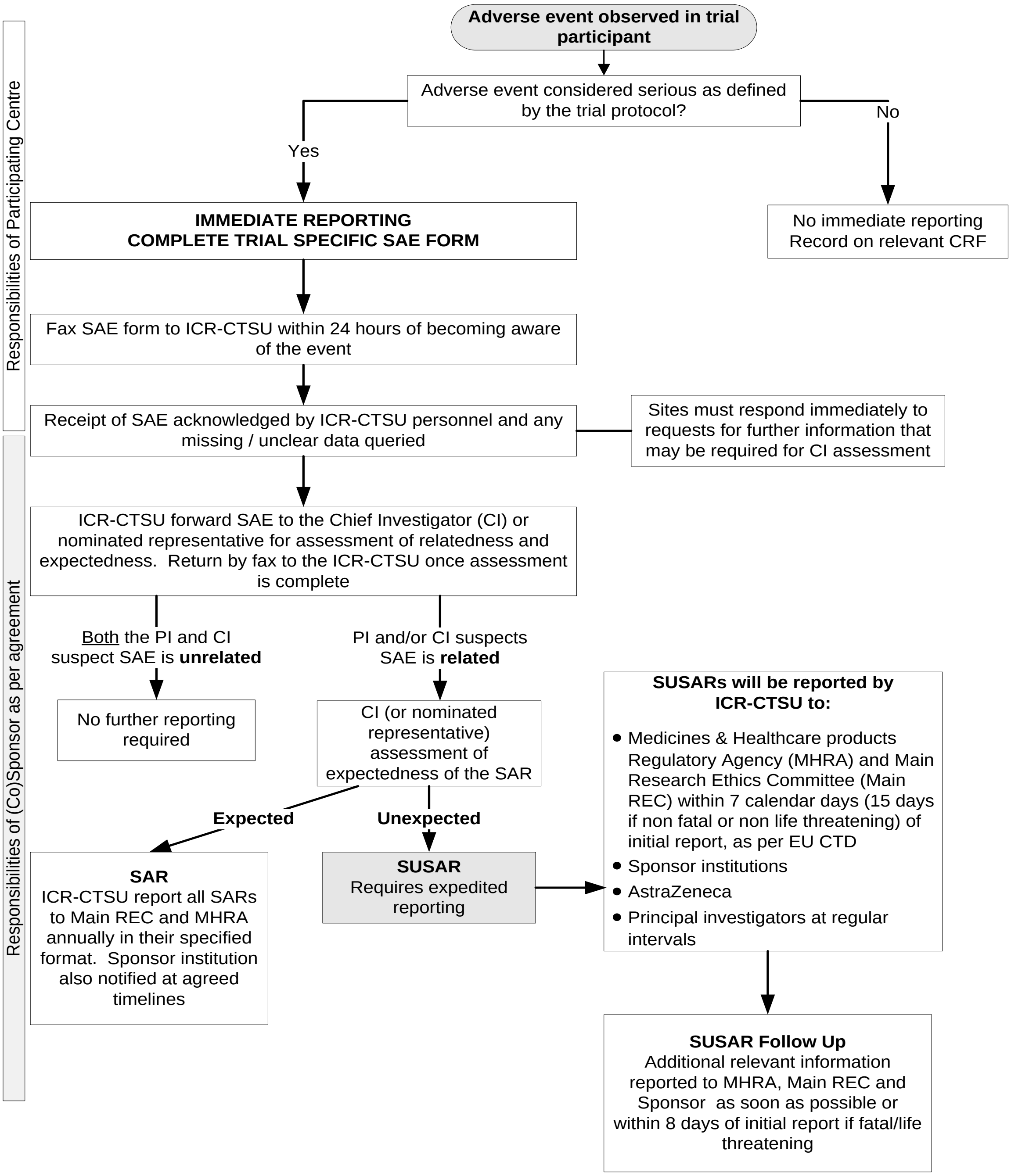

NB. All SAE's should continue to be followed up as specified in the protocol.

14 STATISTICAL CONSIDERATIONS

14.1 Trial Design

The aim of this design is to look for potentially sensitive subgroups of patients by undertaking a screening trial (TOPARP-A i.e. “part A”) followed by a validation trial (TOPARP-B i.e. “part B”) if there is evidence a sensitive subgroup exists.

In both TOPARP-A and TOPARP-B, response rate is being assessed at 6 months post registration as the primary endpoint. These trials will be followed by a randomised phase II trial of olaparib vs placebo in the sensitive patient groups with overall survival as the primary endpoint. The aim is to identify a sensitive subgroup with a 50% response rate on the basis that high response rates are necessary to be confident of demonstrating a survival benefit, the primary goal of the phase II strategy.

It is possible that all patients are sensitive to treatment in which case it has to be possible to apply the same efficacy and inefficacy criteria as are used in the validation trial to all patients in the screening trial.

Part A
The screening trial will involve all comers (unselected patients) and is an open-labelled, multi-centre two-stage design based on H_0: p_0=0.05; H_a ; p_1=0.20; α=0.02 and β=0.10. It will recruit a **maximum of 45 patients** (30 in stage 1 and 15 in stage 2) with an initial efficacy level of 20% and an inefficacy level of 5%, to allow for the fact that only a small subset of patients may be sensitive to olaparib.

This part will evaluate the anti-tumour activity of olaparib in unselected CRPC patients and will identify potential biomarkers of response.

- **Stage 1**

  The first stage will enrol 30 patients and when all 30 have been followed up for a minimum of 6 months an interim analysis will take place to evaluate the response rate:

  Given the efficacy level of 20% and inefficacy level of 5% the trial will be terminated with the conclusion of inefficacy if there are **2 or fewer responses**. If there are **15 or more responses** in the first stage, broad efficacy will be concluded and no more patients will be entered and Part C will commence in unselected patients.

  If between **3 to 14 patients respond**, patient accrual will continue to a total of 45 patients (stage 2) and an investigation into the identification of a potential biomarker of response will be carried out.

- **Stage 2**

  If the trial progresses to stage 2, a further 15 patients will be recruited, giving a total of 45 patients for part A. An interim analysis will be triggered once 45 patients have been followed up for a minimum of 6 months or alternatively we observe 23 or more responses. The recruitment will be halted.

If **23 or more** patients respond, Part C will be initiated. If **5 or fewer patients** respond, the null hypothesis will be accepted and the study will be terminated. If **between 6 and 22 patients respond**, an investigation into the identification of potential biomarkers of response will be carried out. If a potential biomarker subgroup is found, Part B will be undertaken. If a subgroup is not found, then Part C will be undertaken if at least 19 of the patients have responded, otherwise the study will be terminated since sufficient activity has not been observed based on H_0: p_0=0.3; H_a ; p_1=0.5; α =0.059 and β =0.11.

Deriving a classifier for identification of tumours with high susceptibility to PARPi olaparib in CRPC.

All patients participating in TOPARP-A will have their archival tissue, fresh tumour biopsies (and circulating tumour cells collected prior to starting olaparib. At the outset, the primary biomarkers identified for evaluation include PTEN loss by IHC and FISH, *ETS* gene rearrangements by FISH and RAD51 foci formation. It should be noted that PTEN loss and *ETS* rearrangements co-associate and are found in approximately 50% of prostate cancers. In addition that is emerging evidence to suggest that RAD51 foci formation is diminished in tumours with HR defects such as those with loss of PTEN and ETS rearrangements (15, 27, 41, 44).

See section 2.1 for a discussion of relevant biomarkers.

Biomarkers will be classified as either established markers of sensitivity to synthetic lethality with PARPi (e.g. *BRAC1 -/-, BRAC2 -/-*), or as exploratory biomarkers. Established biomarkers will have a guaranteed place in the biomarker classifier but exploratory biomarkers will have to be shown to have predictive ability from the trial data.

The correlation between biomarkers will first be examined using Spearman's rank correlation. If two biomarkers are highly correlated or known to be highly correlated from previous evidence one of the biomarkers will be dropped. The use of appropriate cut-points of continuous scores for individual biomarkers will be decided before examining their association with response.

For potential promotion to the classifier, a false discovery rate of 10% or less will be applied to all exploratory biomarkers, using the p-values from the univariate association with response (Fisher's exact test, p<0.10 2-sided). If evidence exists from other sources it may be incorporated into the assessment of specific biomarkers using meta-analysis to give an 'overall' p-value.

Secondly, identification of a multivariable predictive classifier will be undertaken using a significance level of 10% or less for the inclusion of the biomarker in the classifier (logistic regression). In the event of a tie, e.g. two or more classifiers with similar performance, a liberal approach will be adopted on the basis that it will be possible to exclude non-

responsive biomarker groups at a later date. Standard prognostic factors may be included in the classifier if they are independent of the biomarkers.

A statistical analysis plan will be agreed prior to the primary analysis.
The goal will be to develop a classifier which identifies a group with a response rate of 40% or more on the basis that the levels for inefficacy and efficacy are set at 30% and 50% respectively.

The biomarker defined subgroup satisfying the above criteria will be selected for validation in Part B. If convincing evidence arises from other translational studies of olaparib or other PARP inhibitors the IDMC will be asked to advise whether or not an analysis should take place.

Part B

The biomarker status of patients registered in Part B will be evaluated and only patients with the presence of the putative biomarker subgroup identified in Part A will be entered.Part B will follow a phase II randomised "pick-the-winner" design: eighty-eight patients will be randomised to receive either 400 mg BID or 300 mg BID, with the goal of recommending an optimal dose of olaparib in CRPC to be evaluated in further clinical trials. **Each** dose group will be assessed independently for antitumour activity: the minimum desired activity for each dose group will be assessed by a one-stage A'Hern design, which assumes H_0: p_0=0.3; H_a ; p_1=0.5; α=0.05 and β=0.15. If **19 or more** of the **44 patients** in one dose group respond (43%), then the dose group is considered successful,. If **18 or less** patients in one dose group respond, the dose group will be considered unsuccessful. If the 400 mg BID dose group meets the criteria for "success", the biomarker identified in Part A will be considered validated.

In the case where both dose groups are successful, the play-the-winner selection strategy proposed by Simon, Wittes and Ellenberg (95) modified as suggested by Yap et al (96), is applied to select the superior dose group. If the difference in response rate is larger than 10%, the dose group with higher response rate will be recommended.. If the difference in response rate is ≤10%, dose selection would be based on additional data (time to progression, tolerability, drug exposure). The criteria to select the dose will be described in detail in the Statistical Analysis Plan, which will be finalised before Part B primary analysis.

Further information regarding the sample size calculations of both parts A and B is shown in Appendix A. The detectable effects in the biomarker sensitive group assuming 80% power are presented on Appendix B, together with some power calculations for Part B "pick-the-winner" design

14.2 Treatment Allocation

All trial participants of Part A will be receive olaparib 400 mg BID.

Participants of Part B will be randomised between olaparib 400 mg BID and 300 mg BID on a 1:1 basis. Given that the two dose groups will be assessed independently, the 400 mg BID dose group included in protocol v4 prior to amendment could open to recruitment before the 300 mg BID group (added by amendment of Protocol v4). In that case, once recruitment to the 300 mg BID is open, randomisation will start with an allocation ratio of 2:3 in order to maximize the number of patients recruited concurrently.

Treatment allocation in Part B is by minimisation with a random element, with screening CTC value as a balancing factor.

14.3 Analysis populations

Analysis populations are defined as follows:

- **Intention to treat (ITT):** This population includes all patients enrolled into the study regardless of whether they are later found to be ineligible, a protocol violator, never treated or not evaluated. Patients for whom the primary endpoint cannot be evaluated will be treated as non-responders.

- **Evaluable-patient population:** Patients who meet all of the inclusion and exclusion criteria (Sections 7.3 and 7.4), and who start trial treatment are considered evaluable, unless they discontinue treatment prior to 12 weeks for reasons which aren't drug or disease related**. Where evaluability is difficult to define the final decision will rest with the IDMC.**

- **Per protocol (PP):** This population contains all enrolled patients who received at least 1 cycle of olaparib without major protocol violations.

- **Safety population:** This population includes all enrolled patients who received at least 1 dose of olaparib.

The analysis of the primary outcome will use the evaluable-patient population with sensitivity analyses in the ITT and per protocol populations. All other analyses will use the ITT population. The safety population will be used to further characterise the safety profile of olaparib in advanced CRPC patients.

14.4 Primary Efficacy Endpoint

Response will be defined by:

- Objective response by modified RECIST1.1 (Appendix E) and / or
- Conversion of circulating tumour cell count (CTC) to <5/7.5 ml blood nadir confirmed by a second consecutive value obtained four or more weeks later and / or
- PSA decline of ≥ 50% (according to the PCWG2) (Appendix D)

Evaluable patients with no confirmed response as defined above will be classified as non-responders.

Response will be evaluated 6 months post registration. The last value of PSA, CTC count, CT scan and bone scan on or up to four weeks before the date of first study treatment will be used as the baseline value for this assessment. The response rate will be presented along with its exact two-sided 95% confidence interval.

Part B additional analyses of the primary endpoint: response rate per dose group will be compared by means of a chi-square test or Fisher's test as appropriate. Unadjusted and adjusted estimates of treatment differences (odds-ratios) will be obtained by logistic regression models, adjusting as appropriate by important prognostic factors (stratification factors, other relevant). It should be noted that the study is not powered to detect differences in response rates between dose groups; therefore, these additional analyses should be interpreted with caution and assessed together with additional information from key secondary variables.

14.5 Secondary Efficacy Endpoints

The following endpoints will be assessed 6 months post registration. However, decisions made within the trial to comply with the adaptive design will not necessarily require 6 months follow up on all patients, e.g. it may be possible to enter patients into the second stage of Part A when it is clear between 3 and 14 responses will occur in the first stage even though not all 30 patients have 6 months follow up.

Time-to-PSA Progression

PSA progression is defined according to the consensus guidelines of the Prostate Cancer Clinical Trials Working Group 2.

For patients who have achieved a ≥ 50% decrease from the cycle 1 day1 (baseline), the PSA progression date is defined as the date that a ≥ 25% increase and an absolute increase of ≥ 2 ng/mL above the **nadir** is documented. This must be confirmed by a second consecutive value obtained 4 or more weeks later. For patients without a PSA decrease of this magnitude or no decrease at all, PSA progression date is defined as the date that a ≥ 25% increase and an absolute increase of ≥ 2 ng/mL above the **baseline** is documented. This must also be confirmed by a second consecutive value 4 or more weeks later. Time to PSA progression will be summarised using Kaplan-Meier methods.

Duration of PSA response

Duration of PSA response is calculated from the time the PSA value first declines by at least 50% of the cycle 1 day 1 (baseline) value (must be confirmed by a second value) until the time there is an increase of 25% of PSA nadir, provided the absolute increase is at least 2 ng/mL. The increase must be confirmed by a second consecutive measurement that is at least 25% above the nadir. If the PSA never shows a 25% increase over the nadir value, then the patient will be assessed at the last PSA measurement. Duration of PSA response will be summarised by the median and presented along its 95% confidence interval.

Pattern of PSA Responses

Waterfall plots will be presented (as per PCWG2 recommendations) that show the percentage of change in PSA from baseline to 12 weeks (or earlier for those who discontinue therapy), as well as the maximum decline in PSA that occurs at any point after treatment. Waterfall plots provide a broader display of PSA changes than dichotomised responses.

CTC Response

The proportion of patients with a CTC conversion to <5/7.5 ml blood at nadir (confirmed by a second consecutive value obtained four or more weeks later) will be presented along an exact two-sided 95% confidence interval. Its association with PFS and OS will be examined via Cox regression models first univariately and subsequently in a multivariable model adjusting for potential confounders. Waterfall plots of CTC falls will also be presented that show the percentage change in CTC counts from baseline to 12 weeks as well as maximal CTC count declines that occur at any point after treatment.

The following endpoints will be evaluated using Kaplan and Meier methods:

Radiographic Progression Free Survival (rPFS)

Radiographic progression free survival (rPFS) will be defined by either RECIST progression and /or progression on bone scan (Appendix D and E). It will be measured from the date of trial entry to the first occurrence of radiographic progression or death from any cause. If no event exists, then rPFS will be censored at the last scheduled disease assessment on study.

Time to Radiographic Progression

Time to radiographic progression (progression defined by either RECIST progression and /or progression on bone scan) will be measured from the date of trial entry to the first occurrence of radiographic progression. Death from prostate cancer or any other cause without prior radiographic evidence of progression will not count as an event. If no event exists, then time to radiographic progression will be censored at the last scheduled disease assessment on study or date of death whichever occurs earlier.

Progression Free Survival (PFS)

Progression free survival will be measured from the date of trial entry until radiographic progression, unequivocal clinical progression or death. If no event exists, then PFS will be censored at the last scheduled disease assessment on study.

Overall Survival (OS)

OS will be measured from the date of trial entry to the date of death (whatever the cause). Survival time of living patients will be censored on the last date a patient is known to be alive or lost to follow-up.

14.6 Tertiary/exploratory Endpoints

- **Pain palliation:**

Pain palliation will be assessed using the Brief Pain Inventory's (BPI Short Form) worst pain intensity score and average score. A composite of the four pain items (worse, least, average, right now) will be presented as supplemental information. Change in BPI score from baseline will be assessed at 6 months post registration. A reduction of two points in the worst pain score relates to an improvement of pain. The analgesic relief received will be described.

- **Safety Endpoint**

Safety analysis will be summarised using the safety population. Extent of exposure to study drug will be summarised and details will be provided. Treatment emergent adverse events (AEs) are those events that occur or worsen on or after first dose of study drug up through 30 days post last dose. Adverse events will be graded according to the NCI-CTCAE v4.02. Adverse events will be summarised by grade, according to the worst grade experienced. In addition, most frequently observed AEs will be summarised. In the summary of AE, an AE occurs more than once within a SOC and PT will be counted only once using the worst grade experienced. Serious AE and deaths observed within 30 days

of the last dose of study treatment will be provided in a listing.

All adverse events resulting in discontinuation, dose modification, dosing interruption, and/or treatment delay of study drug will also be listed and tabulated by preferred term. Clinical laboratory test results will be collected pre-treatment and through 30 days post last dose of study treatment. All laboratory test results will be classified according to the NCI CTCAE v4.02 criteria. Standard reference ranges will be used for missing or discrepant normal ranges. Baseline laboratory test values are the results from the last blood samples drawn on or prior to the first day of study treatment. On-study laboratory test values are those results from blood samples drawn a day after the first study treatment up until 30 days after the last dose of study treatment.

- **Impact of treatment with olaparib on bone metastases imaging by DW-MRI.**

Diffusion-weighted MRI scan will be offered to patients recruited in part A and we expect approximately 50% acceptance rate. This analysis will be **purely exploratory** and we will examine whether response to olaparib expressed by changes in apparent diffusion coefficients produced by the DW-MRI scan correlate with 1) PSA decline, 2) CTC conversion and 3) radiological responses coming from conventional imaging. In 20 patients it is possible to detect a correlation coefficient of 0.6 which is equivalent to the parameter explaining 36% of the variation using a 2-sided 5% significance level. The DW-MRI sub study will continue to be offered to patients recruited for part B to complement and confirm the data obtained in part A of the study.

- **Response rate following dose-escalation from 300mg to 400mg BID in part B.**

Patients in part B allocated to the 300mg BID dose group will be allowed to dose-escalate to 400mg BID upon confirmation of disease progression, providing it is considered clinically indicated in each case and that the patient did not required a dose reduction while on therapy. The rate of responses following dose-escalation will be calculated and presented with descriptive statistics.

14.7 Data Monitoring

An Independent Data Monitoring Committee will meet in confidence at regular intervals, firstly to review the safety analysis following the first 5 and 10 patients recruited and subsequently every 6 months (or at intervals determined by them) to review accrual, safety and efficacy data.

The replacement of patients who withdraw from the study or who are un-evaluable for reasons deemed not to be treatment related will be based on the advice of the committee. The committee will review the results of each interim analysis (progression from stage 1 to stage 2 and progression to part B) and will be asked to advise. For completeness a sensitivity analysis of the progression free rate calculated by the Kaplan Meier product limit method will also be presented in which withdrawals and deaths are censored. The summary statistic will be the progression free rate at 6 months together with its 95% one-sided confidence interval.

15 TRANSLATIONAL RESEARCH

Paraffin embedded tumour blocks or unstained sections from archival tumour tissue from the time of the original diagnosis or from a biopsy / resection of metastatic disease will be required from all subjects who enrol on the study. Patients who do not have adequate archival tumour tissue will be required to have tumour confirmed in a repeat tumour biopsy in order to be enrolled on the study. CTCs, urine, plasma samples and mandatory fresh tumour biopsies will be obtained at various time points during the study (Tables 3 & 8). CTCs will be enumerated to evaluate conversion rates (CTC from ≥5 cells/7.5ml blood at baseline to < 5cells/7.5mls) on this treatment. The tumour tissue will be assessed for multiple biomarkers including *PTEN* and *ERG* expression, *PTEN* deletions, *TMPRSS2-ETS* gene rearrangements using multicolour FISH studies, markers of DNA damage and HR repair such as γH2AX and RAD51 focus formation, markers of tumour cell proliferation and cell cycle phase and mutational analysis through hotspot and/or whole genome sequencing. Blood samples for plasma-based biomarker will be utilised for several studies including obtaining normal germline DNA and circulating tumour DNA for mutational analysis (e.g. *BRCA1, BRCA2, CHEK2, etc.*), genome sequencing, quantitative real time polymerase chain reaction for ERG detection, proteomics, metabolomics, RNA and microRNA (miRNA) studies. Urine samples will be utilised to evaluate metabolomics, RNA and urine telopeptides profiles. Additional putative predictive biomarkers involved in the HR DNA repair pathway and sensitivity to PARP inhibition may be evaluated to facilitate the characterisation optimal determinants of response and resistance to olaparib treatment. Some of these additional markers/studies may come from other translational studies.

All the tumour tissue, CTC, plasma and urine samples will be sent to the Institute of Cancer Research Biomarker Group for processing and analysis. Some of these samples may be labelled with the patients' initials, hospital pathology number and trial number when they are sent to the research laboratory. When they arrive at the laboratory each sample will be coded with a unique anonymised number and any personal details removed. The number generated is linked to the patient trial ID held at the ICR CTSU. This coding system will maintain patient confidentiality whilst allowing biological details to be compared to treatment findings.

Samples will be stored in a secure area in the Cancer Biomarkers Laboratory at the Institute of Cancer Research. Samples will only be accessible by accredited staff involved in the processing and analysis of the samples. Some of the tumour/plasma samples collected may need to be sent to another site for processing and analysis. This will only occur following an established agreement being set up and assurance that patient confidentially is maintained. The pharmacokinetic samples will be sent to laboratory contracted by the co-sponsors. **Instructions for the sample requirements, collection, handling, labelling, shipment and storage will be provided in a reference TOPARP Laboratory Manual for Investigators.**

In some cases the archival tumour blocks will be returned to the local laboratories after the study is complete, depending on local practice. Patients participating on this study will be asked to give permission for possible future research into prostate cancer using these samples. The confidential nature of the tissue and associated data will be fully protected, and any other research using your tissue will first be reviewed and approved by an ethics committee.

These tumour and plasma samples may be used for, but not restricted to, the elucidation of mechanism of response, understanding the mode of action of olaparib and improving the understanding of disease progression.

15.1 Archival Tumour Blocks and Fresh Biopsies

An adequately sized (minimum of 2 mm x 2 mm) archival tumour tissue paraffin block from resection or a core biopsy from the time of the original diagnosis or from a biopsy / resection of metastatic disease at any time prior to study entry is required. Alternatively, unstained slides prepared from the block can be provided. Mandatory fresh biopsies should be obtained at baseline (during the screening period; anytime between Day-28 to Day1), post commencement of treatment (during Cycle 1; anytime between Day 8 and Day 28) and at study discontinuation. The third biopsy is optional and will be obtained at any point between the patient coming off study treatment to the end of treatment visit. All fresh tumour biopsies will be optional in Part B.

The fresh tumour biopsy may be obtained from either the prostate or a metastatic site that is safe and amendable to a biopsy. Tumour biopsies may be obtained from the prostate itself (if it has not been surgically resected and has not been treated with radiotherapy) via an ultrasound-guided transrectal biopsy. Local recommendations for the procedure will be followed. Alternative sites where a biopsy can be obtained include lymph nodes, bone or liver where there is evidence of cancer on imaging. Patients who do not have evidence of visceral disease but do have evidence of bone metastasis on the bone scan may undergo a bone biopsy and possible aspirate from the ischium. Ideally, the pre- and post-treatment biopsies will be from the same site. Refer to the TOPARP Laboratory Manual for specific details on sample collection, handling, storage and shipping requirements.

15.2 Circulating Tumour Cell (CTC) Assessments

Baseline samples will be collected at screening and one Cycle 1 Day 1 and subsequently CTC blood samples will be collected as per Tables 3a and 3c. CTCs will be enumerated using Veridex CellSearch™ platform. CTC samples in all patients will be used to enumerate CTC counts and evaluate the molecular profile of tumour cells. Refer to the TOPARP Laboratory Manual for specific details on sample collection, handling, storage and shipping requirements. Amongst the molecular markers that will be studied include:

Immunoflorescence: PTEN, ERG, RAD51, γH2AX and markers of tumour cell proliferation and cell cycle phase.

FISH: Including *PTEN, TMPSS2/ERG*

Next generation sequencing and array CGH may be conducted on the isolated CTCs.

15.3 Plasma and Urine-Based Biomarkers

Plasma and urine samples for different analyses will be collected at multiple time points during the study (refer to Table 7 for details on the specific samples). These samples will be utilised for the purpose of analysing circulating plasma biomarkers including circulating

tumour DNA and RNA for mutational analyses, quantitative real time polymerase chain reaction of ERG detection, plasma proteomic signature, plasma and urine metabolomic studies and microRNA (miRNA) profiles. Proteomic, metabolomic and miRNA profiling may generate signatures that can be used as predictive, prognostic and pharmacodynamic biomarkers that may also be correlated with other study endpoints. Refer to the TOPARP Laboratory Manual for specific details on sample collection, handling, storage and shipping requirements.

Plasma

- **Plasma Metabolomic Profiling:** Blood samples will be obtained as per Tables 3a and 3c 5min pre-dose and 2hour post-dose. Patients will be required to fast for at least 8 hours before providing these metabolomic blood samples. During the fasting period water or tea without milk and sugar will be allowed in moderate amounts. For patients who are unable to fast (e.g. insulin dependent diabetic patients) samples should be collected consistently in a non-fasted state. Whenever possible patients should be seen first thing in the morning to limit the burden of fasting. After the first metabolomic blood draw patients will be permitted to have a light breakfast.

- **PAXgene miRNAs Analyses**: Whole blood samples will be collected as per Tables 3a and 3c.

- **Plasma Proteonomic profiling:** Plasma samples will be obtained as per Tables 3a and 3c.

- **Circulating Plasma DNA and RNA:** Plasma samples will be collected as per Tables 3a and 3c

Urine

- **Urine Markers of Bone Turnover (telopeptides):** A urine sample collected after the void of the first morning sample will be collected as per Tables 3a and 3c.

- **Urine Metabolomic Profiling:** 24 hour urine samples will be collected as per Tables 3a and 3c.

The 24 hour urine sample will be collected from the second urine of the previous day until the first urine of the clinic visit day. The urine sample for markers of bone turnover will be collected at the second urine of the clinic visit day.

15.4 Buccal Swabs

A buccal swab will be collected at screening in Part A only.

15.5 Saliva Collection

A saliva sample will be collected at screening in Part A and pre-screening/screening in Part B.

15.6 Diffusion-weighted MRI

Diffusion-weighted MRI (DW-MRI) will be performed on an optional basis in a subset of patients. DW-MRI provides better visualisation of tumour cellularity and the results of DW-MRI will be correlated with bone scan results, PSA, CTC counts and patient outcome.

15.7 Rationale of translational research

The intent of the biomarker / translational research embedded in this protocol is outlined below:

- PART B only - To evaluate if the drug disposition in castrate men at 300mg bid is significantly different to that at 400mg bid, and different to exposure in women treated for ovarian cancer.
- To characterise the molecular profile of prostate cancer and sub-divide this heterogeneous disease.
- To develop predictive biomarkers of olaparib sensitivity which may be utilised to optimise patient selection for future studies.
- To correlate the molecular profile of prostate cancer (in particular but not exclusively *PTEN*, *ERG* status by both IHC and FISH analysis) with clinical outcome.
- To determine target modulation from olaparib treatment by the generation of γH2AX foci formation, a marker of DNA damage in fresh biopsies and CTCs.
- To evaluate the utility of early changes to CTC counts as a surrogate of clinical benefit to PARP inhibitors in CRPC.
- To correlate CTC changes with PSA and radiological responses to olaparib treatment in CRP.

15.8 Whole Genome Sequencing

Samples will be sent to a laboratory approved by the Trial Management Group for genetic testing. Patients will consent to results relevant for the management of their cancer to be reported to them and their clinicians. Patients will be given the option to receive results which are not related to their cancer, but may have potential medical impact for them or significance for biological family members. The feedback of these results may not necessarily be in real-time and only very well known, established signatures will be fed back e.g. BRCA mutations. Principal Investigators should give consideration to using clinical geneticists in the feedback of results to patients.

Table 8: Part A Summary of the blood and urine-based biomarker samples for the translational research

TIME POINTS			BLOOD BASED BIOMARKERS					URINE BIOMARKERS	
	Saliva	Buccal Swab	CTC	Plasma DNA & RNA Analyses	PAXgene miRNA	Plasma Proteonomic Profile	Plasma Metabolomic Profiling (pre and post dose)	Urine Metabolomic profiling	Bone markers of turnover
Screening	X	X	2x10mls	3x10mls	1x2.5mls	1x8.5mls	2x6mls	24 urine collection	Urine collected after the void of the first morning sample
Cycle 1 day 1			2x10mls	3x10mls	1x2.5mls	1x8.5mls	2x6mls	24 urine collection	Urine collected after the void of the first morning sample
Cycle 1 day 8			2x10mls	3x10mls			2x6mls	24 urine collection	
Cycle 1 day 15			2x10mls						
Cycle 2 day1			2x10mls	3x10mls			2x 6mls	24 urine collection	
Cycle 3 day1			2x10mls	3x10mls	1x2.5mls		2x6mls	24 urine collection	Urine collected after the void of the first morning sample
Cycle 4day1			2x10mls						
Cycle 5 day1				3x10mls	1x2.5mls				
Cycle 6 day1							2x6mls	24 urine collection	Urine collected after the void of the first morning sample
Cycle 7day1 & every 3rd cycle thereafter			2x10mls						
End of Treatment Visit			2x10mls	3x10mls	1x2.5mls	1x8.5mls	2x6mls	24 urine collection	Urine collected after the void of the first morning sample

For total volumes of safety and research bloods refer to Appendix J

Table 8: Part B Summary of the blood and urine-based biomarker samples for the translational research

TIME POINTS	Saliva	BLOOD BASED BIOMARKERS					URINE BIOMARKERS	
		CTC	Plasma DNA & RNA Analyses	PAXgene miRNA	Plasma Proteonomic Profile	Plasma Metabolomic Profiling (pre and post dose)	Urine Metabolomic profiling (24hr urine collection)	Bone markers of turnover (Urine collected after void of the first morning sample)
Pre-Screening	X							
Screening	X	2x10mls	3x10mls	1x2.5mls	1x8.5mls	2x6mls	X	X
Cycle 1 day 1		2x10mls	3x10mls	1x2.5mls	1x8.5mls	2x6mls	X	X
Cycle 1 day 8		2x10mls	3x10mls			2x6mls	X	
Cycle 2 day1		2x10mls	3x10mls			2x6mls	X	
Cycle 3 day1		2x10mls	3x10mls	1x2.5mls		2x6mls	X	X
Cycle 4day1		2x10mls	3x10mls					
Cycle 5 day1			3x10mls	1x2.5mls				
Cycle 6 day1			3x10mls			2x6mls	X	X
Cycle 7day1 & every cycle thereafter			3x10mls					
Cycle 7day1 & every 3rd cycle thereafter		2x10mls	(every cycle)					
Dose Escalation (300mg group only)		2x10mls	3x10mls	1x2.5mls				X
End of Treatment Visit		2x10mls	3x10mls	1x2.5mls	1x8.5mls	2x6mls	X	X

For total volumes of safety and research bloods refer to Appendix J

16 TRIAL MANAGEMENT

The trial will be coordinated by ICR-CTSU in accordance with their quality management system.

16.1 Trial Management Group (TMG)

A Trial Management Group (TMG) will be set up and will include the Chief Investigator, Co-investigators and identified collaborators, the Trial Statistician and the Trial Managers. Principal Investigators and key study personnel will be invited to join the TMG as appropriate to ensure representation from a range of centres and professional groups. Notwithstanding the legal obligations of the Co-Sponsors and Chief Investigator, the TMG have operational responsibility for the conduct of the trial. Where possible, membership will include a lay/consumer representative. The Committee's terms of reference, roles and responsibilities will be defined in a charter issued by ICR-CTSU.

16.2 Trial Steering Committee (TSC)

A Trial Steering Committee (TSC) will be set up and will include an independent Chairman (not involved directly in the trial other than as a member of the TSC) and not less than two other independent members. It is the role of the TSC to monitor progress of the trial and to ensure there is adherence to the protocol and the principles of Good Clinical Practice. The Committee's terms of reference, roles and responsibilities will be defined in a charter issued by ICR-CTSU.

16.3 Independent Data Monitoring Committee (IDMC)

An IDMC will be instigated to monitor the progress of the trial. Membership of the IDMC will be proposed by the TMG and approved by the TSC. The Committee's terms of reference, roles and responsibilities will be defined in a charter issued by ICR-CTSU. The IDMC should meet in confidence at regular intervals, firstly to review the safety analysis following the first 5 and 10 patients recruited and at least every 6 months following. A report of the findings and recommendations will be produced following each meeting. This report will be submitted to the TMG and TSC, and if required, the main REC and the MHRA.

The IDMC reserve the right to release any data on outcome or side-effects through the TSC to the TMG (and if appropriate to participants) if it determines at any stage that the combined evidence from this and other studies justifies it.

17 RESEARCH GOVERNANCE

This Clinical Trial will be conducted in accordance with the ethical principles laid down by the Declaration of Helsinki, 1964 and as amended in 1996 and the principles of Good Clinical Practice.

17.1 Sponsor Responsibilities

The Co-sponsors are The Institute of Cancer Research (ICR) and The Royal Marsden NHS Foundation Trust (RMH). Sponsorship activities and delegated responsibilities are shared between ICR and RMH, in accordance with The Medicines for Human Use (Clinical Trials) Regulations 2004 as amended and in line with the Research Governance Framework for Health and Social Care and the principles of GCP. Responsibilities of the Co-sponsors are set out in an agreement letter between ICR and RMH.

17.2 Principal Investigator Responsibilities

Responsibilities of each Principal Investigator and participating centre will be detailed in a contract with the Co-sponsors.

Principal Investigator responsibilities include putting and keeping in place arrangements to run the trial at their site according to the trial protocol and applicable guidance notes, The Medicines for Human Use (Clinical Trials) Regulations 2004 as amended and in line with the Research Governance Framework for Health and Social Care and the principles of GCP. The above responsibilities include, but are not limited to, the following:

- Putting and keeping in place arrangements to adhere the principles of GCP
- Keeping a copy of all ‘essential documents’ (as defined under the principles of GCP) and ensuring appropriate archiving and destruction of documentation once the trial has ended as required by regulation 31 of the principal Regulations of the Medicines for Human Use (Clinical Trials) Regulations 2004 implementing the commission directive 2005/28EC
- Ensuring investigational medicinal products (IMPs) are made available to subjects free of charge
- Taking appropriate urgent safety measures and
- Ensuring recording and prompt reporting of SAEs/SARs to ICR-CTSU

ICR is responsible for co-ordinating any required legal agreements and investigator statements.

The delegation of sponsorship responsibilities does not impact on or alter standard NHS indemnity cover. The agreement of delegated responsibilities is viewed as a partnership and as such it is necessary to share pertinent information between ICR and RMH/Chief Investigator, including proposed inspections by the MHRA and/or other regulatory bodies.

17.3 AstraZeneca Responsibilities

AstraZeneca are responsible on behalf of the co-sponsors for the manufacture, packing, labelling and distributing of study drug and matching placebo to site in accordance with Good Manufacturing Practice and all applicable local legislation. Some of these responsibilities have been delegated by AstraZeneca to Fisher Clinical Services. Responsibilities are defined in an agreement between AstraZeneca and the co-sponsors, and AstraZeneca and Fisher Clinical Services.

18 TRIAL ADMINISTRATION & LOGISTICS

18.1 Protocol Compliance

The TOPARP Trial is being conducted in accordance with the professional and regulatory standards required for non-commercial research in the NHS under the EU Clinical Trials Directive. Before activating the trial, participating centres are required to sign an agreement accepting responsibility for all trial activity which takes place within their centre.

Sites may commence recruitment once centre agreements have been signed by both parties, trial documentation is in place and a site initiation (visit or teleconference) has taken place. Site initiation visits will be conducted at sites where the Principal Investigator has requested one or where ICR-CTSU deems it is appropriate.

18.2 Data Acquisition

ICR-CTSU is responsible for the data management and statistical analysis of trial data. Electronic (e) Case Report Forms (eCRF) will be used for the collection of trial specific data. The Trial Management Group reserves the right to amend or add to the eCRF as appropriate. Such changes do not constitute a protocol amendment, and revised or additional forms should be used by centres in accordance with the guidelines provided by ICR-CTSU.

The clinical data should be recorded in the TOPARP eCRFs in a timely manner.

By participating in the TOPARP trial, the Principal Investigators at each centre are confirming agreement with his/her local NHS Trust to ensure that:

- Sufficient data is recorded for all participating patients to enable accurate linkage between hospital records and eCRFs;
- Source data and all trial related documentation are accurate, complete, maintained and accessible for monitoring and audit visits;
- All staff at their centre who are involved with the trial will meet the requirements of the EU Directive;
- Original consent forms are dated and signed by both patient and investigator and are kept together in a central log together with a copy of the specific patient information sheet(s) given at the time of consent;
- All essential documents must be retained for five years after the Trial ends to comply with current legislation.
- Staff will comply with the protocol and Trial Guidance Notes for the TOPARP trial.

Completion of eCRFs will be monitored by ICR-CTSU and any missing forms will be reported to the originating site.

18.3 Central Data Monitoring

Once data has been entered by the site personnel on the eCRF, the ICR-CTSU will review and check the data for compliance with the protocol, and for inconsistent or missing data. Should any missing data or data anomalies be found, queries will be issued to the centre for resolution.

Data will be further reviewed for data anomalies / missing data, by central statistical monitoring. Any systematic inconsistencies identified may trigger monitoring visits to centres.

18.4 On-Site Monitoring

Site monitoring will be conducted at participating centres at least once during the Part A. On-site monitoring in Part B will be informed by the risks identified in Part A. Additional on-site monitoring may be triggered by routine central statistical monitoring or identification of a new risk to the trial.

If a monitoring visit is required, ICR-CTSU will contact the centre to discuss dates of proposed visit. Once a date has been confirmed, the centre should ensure that the relevant patient notes are available for monitoring.

If any problems are detected in the course of the monitoring visit, ICR-CTSU will work with the Principal Investigator to resolve issues and, if necessary, to determine the centre's future participation in the study.

ICR-CTSU staff conducting on-site monitoring will review essential documentation and carry out source data verification to confirm compliance with the site agreement and trial protocol to ensure the protection of patients' rights as detailed in the Declaration of Helsinki 1964 as amended October 1996.

18.5 End of trial

The end of trial for MHRA and MREC purposes is the date of last data capture

18.6 Archiving

Essential documents are documents that individually and collectively permit evaluation of the conduct of the trial and substantiate the quality of the data collected. They should be retained for a sufficient period (at least 5 years after the date of last date of data capture) for possible inspection by the regulatory authorities. Documents should be securely stored and access restricted to authorised personnel.

19 PATIENT PROTECTION AND ETHICAL CONSIDERATIONS

The trial will have received ethical, regulatory and institution specific approvals prior to recruitment of any patients into the study. Before entering patients, the Principal Investigator at each site is responsible for gaining Site Specific Assessment and Research and Development approval of this protocol.

Patients should be asked to sign the trial consent forms after receiving both verbal and written information about the trial. All consent forms must be countersigned by the Principal Investigator or a designated individual. A record listing the designated individuals and the circumstances under which they may countersign consent forms must be clearly documented at the research site as part of the Delegation of Responsibilities Log. This log, together with original copies of all signed patient consent forms, must be available for inspection.

The TOPARP patient information sheets should be provided in addition to any standard patient information sheets that are provided by the centre and used in routine practice.

19.1 Patient Confidentiality

Patients will be asked to consent to their full name being collected at registration in addition to their date of birth, hospital number, postcode and NHS number (CHI in Scotland) to allow tracing through national records to assist with long term follow up and to allow linkage with routinely collected NHS data. The personal data recorded on all documents will be regarded as confidential, and any information which would allow individual patients to be identified will not be released into the public domain.

Each investigator should keep a separate log of all patients' Trial IDs, names, addresses and hospital numbers. The investigator must maintain trial documents, which are to be held at the participating centres (e.g. patients' written consent forms), in strict confidence. The investigator must ensure the patients' confidentiality is maintained.

ICR-CTSU will maintain the confidentiality of all patients and will not reproduce or disclose any information by which patients could be identified. Representatives of ICR-CTSU (or third parties approved by the Sponsor) and the regulatory authorities will be required to have access to patients notes for quality assurance purposes but patients should be reassured that their confidentiality will be respected at all times. In the case of special problems and/or competent authority queries it is also necessary to have access to the complete study records provided that patient confidentiality is protected.

19.2 Data Protection

ICR-CTSU will comply with all applicable data protection laws. Any requests from patients for access to data about them held at ICR-CTSU should be directed to the Trial Manager in the first instance who will refer the request to the Data Protection Officer at The Institute of Cancer Research.

19.3 Liability

The Co-Sponsors have taken out an insurance policy to cover their study responsibilities, and certification of this will be provided to the regulatory authorities as required. ICR-CTSU will need to be satisfied that all participating sites have appropriate indemnity arrangements in place.

20 FINANCIAL MATTERS

The trial is investigator designed and led and has been approved by Clinical Trials Advisory & Awards Committee (CTAAC) of Cancer Research UK, and meets the criteria for R&D support as outlined in the Statement of Partnership on Non-Commercial R&D in the NHS in England.

ICR has received funding from Cancer Research UK and AstraZeneca (via the AstraZeneca/NCRN collaboration) for the central coordination of the trial. AstraZeneca are also providing olaparib for the purposes of this trial free of charge.

In the UK, the trial meets the criteria for R&D support as outlined in the Statement of Partnership on Non-Commercial R&D in the NHS in England. The trial is part of the National Institute for Health Research (NIHR) portfolio by virtue of its approval by CTAAC. Therefore, National Cancer Research Network (NCRN) resources should be made available for the TOPARP trial to cover UK specific research costs.

21 PUBLICATION POLICY

The main trial results will be published in a peer-reviewed journal, on behalf of all collaborators. The manuscript will be prepared by a writing group consisting of members of the TMG and key collaborators and led by the chief investigator. Participating investigators may be selected to join the writing group on the basis of intellectual and time input. All participating centres and clinicians will be acknowledged in this publication together with the dedicated staff from the ICR-CTSU. Any presentations and publications relating to the trial must be authorised by the Trial Management Group, on whose behalf publications should usually be made. Authorship of any secondary publications e.g. relating to the various biological studies will reflect the intellectual and time input into these studies, and may not be the same as on the primary publication. No investigator may present or attempt to publish data relating to the TOPARP trial without prior permission from the Trial Management Group.

22 REFERENCE LIST

1. http://www.cancerresearchuk.org
2. de Bono JS, Logothetis CJ, Molina A,et al. Abiraterone and increased survival in metastatic prostate cancer. N Engl J Med 2011;364(21):1995-2005.
3. Scher HI, Beer TM, Higano CS, et al. Antitumour activity of MDV3100 in castration-resistant prostate cancer: a phase 1-2 study. Lancet 2010; 375(9724): 1437-46.
4. Tannock IF, de Wit R, Berry WR,et al. Docetaxel plus prednisone or mitoxantrone plus prednisone for advanced prostate cancer. N Engl J Med 2004;351(15):1502-12.
5. de Bono JS, Oudard S, Ozguroglu M,et al. Prednisone plus cabazitaxel or mitoxantrone for metastatic castration-resistant prostate cancer progressing after docetaxel treatment: a randomised open-label trial. Lancet 2010;376(9747):1147-54.
6. Kantoff PW, Higano CS, Shore ND, et al. 1.Sipuleucel-T immunotherapy for castration-resistant prostate cancer.N Engl J Med. 2010;363(5):411-22.
7. Sella A, Yarom N, Zisman A, Kovel S. Paclitaxel, estramustine and carboplatin combination chemotherapy after initial docetaxel-based chemotherapy in castration-resistant prostate cancer. Oncology 2009;76(6):442-6.
8. Birtle AJ, Newby JC, Harland SJ. Epirubicin carboplatin and 5-fluorouracil (ECarboF) chemotherapy in metastatic hormone refractory prostate cancer. Br J Cancer. 2004;91(8):1472-6.
9. Sternberg CN, Petrylak DP, Sartor O, et al. Multinational, double-blind, phase III study of prednisone and either satraplatin or placebo in patients with castrate-refractory prostate cancer progressing after prior chemotherapy: the SPARC trial. J Clin Oncol 2009;27(32):5431-8.
10. Fong PC, Boss DS, Yap TA,et al. Inhibition of poly(ADP-ribose) polymerase in tumors from BRCA mutation carriers. N Engl J Med 2009;361(2):123-34.
11. Fong PC, Yap TA, Boss DS, et al. Poly(ADP)-ribose polymerase inhibition: frequent durable responses in BRCA carrier ovarian cancer correlating with platinum-free interval. J Clin Oncol 2010;28(15):2512-9.
12. Berger MF, Lawrence MS, Demichelis F, et al. The genomic complexity of primary human prostate cancer. Nature 2011;470(7333):214-20.
13. Attard G, Swennenhuis JF, Olmos D, et al. Characterization of ERG, AR and PTEN gene status in circulating tumor cells from patients with castration-resistant prostate cancer. Cancer Res 2009;69(7):2912-8.
14. Clark J, Attard G, Jhavar S, et al. Complex patterns of ETS gene alteration arise during cancer development in the human prostate. Oncogene. 2008;27(14):1993-2003.
15. Reid AH, Attard G, Ambroisine L,et al. Molecular characterisation of ERG, ETV1 and PTEN gene loci identifies patients at low and high risk of death from prostate cancer. Br J Cancer 2010;102(4):678-84.
16. Mehra R, Han B, Tomlins SA, et al. Heterogeneity of *TMPRSS2* gene rearrangements in multifocal prostate adenocarcinoma: molecular evidence for an independent group of diseases. Cancer Res 2007; 67(17): 7991-5
17. Tomlins SA, Rhodes DR, Perner S, et al. Recurrent fusion of TMPRSS2 and ETS transcription factor genes in prostate cancer. Science 2005; 310(5748): 644-8.
18. Attard G, Clark J, Ambroisine L, et al. Duplication of the fusion of TMPRSS2 to ERG sequences identifies fatal human prostate cancer. Oncogene 2008; 27(3): 253-63.
19. Salmena L, Carracedo A, Pandolfi PP. Tenets of PTEN tumor suppression. Cell 2008;133(3):403-14.
20. Whang YE, Wu X, Suzuki H, et al. Inactivation of the tumor suppressor PTEN/MMAC1 in advanced human prostate cancer through loss of expression. Proceedings of the National Academy of Sciences of the United States of America 1998; 95(9): 5246-50.
21. Sarker D, Reid AH, Yap TA, de Bono JS. Targeting the PI3K/AKT pathway for the treatment of prostate cancer. Clin Cancer Res 2009; 15(15): 4799-805.

22. King JC, Xu J, Wongvipat J, Hieronymus H, et al. Cooperativity of TMPRSS2-ERG with PI3-kinase pathway activation in prostate oncogenesis. Nat Genet 2009;41(5):524-6.
23. Verhagen PC, van Duijn PW, Hermans KG,et al. The PTEN gene in locally progressive prostate cancer is preferentially inactivated by bi-allelic gene deletion. J Pathol 2006;208(5):699-707.
24. Sircar K, Yoshimoto M, Monzon FA,et al. PTEN genomic deletion is associated with p-Akt and AR signalling in poorer outcome, hormone refractory prostate cancer. J Pathol. 2009;218(4):505-513.
25. Majumder PK, Sellers WR. Akt-regulated pathways in prostate cancer. Oncogene. 2005;24(50):7465-74.
26. Yoshimoto M, Cunha IW, Coudry RA, et al. FISH analysis of 107 prostate cancers shows that PTEN genomic deletion is associated with poor clinical outcome. Br J Cancer. 2007;97(5):678-85.
27. Yoshimoto M, Joshua AM, Cunha IW, et al. Absence of TMPRSS2:ERG fusions and PTEN losses in prostate cancer is associated with a favorable outcome. Mod Pathol. 2008;21(12):1451-60.
28. Yoshimoto M, Cutz JC, Nuin PA,et al. Interphase FISH analysis of PTEN in histologic sections shows genomic deletions in 68% of primary prostate cancer and 23% of high-grade prostatic intra-epithelial neoplasias. Cancer Genet Cytogenet 2006;169(2):128-37.
29. Wang S, Gao J, Lei Q, et al. Prostate-specific deletion of the murine Pten tumor suppressor gene leads to metastatic prostate cancer. Cancer Cell 2003;4(3): 209-221.
30. Majumder PK, Yeh JJ, George DJ, et al. Prostate intraepithelial neoplasia induced by prostate restricted Akt activation: the MPAKT model Proc Natl Acad Sci U S A. 2003.1
31. Saal LH, Gruvberger-Saal SK, Persson C, et al. Recurrent gross mutations of the PTEN tumor suppressor gene in breast cancers with deficient DSB repair Nat Genet 2008;40(1):102-7.
32. Baker SJ. PTEN enters the nuclear age. Cell 2007;128(1):25-8.
33. Kumar-Sinha C, Tomlins SA, Chinnaiyan AM. Recurrent gene fusions in prostate cancer. Nat Rev Cancer 2008;8(7):497-511.
34. Carver BS, Tran J, Gopalan A, et al. Aberrant ERG expression cooperates with loss of PTEN to promote cancer progression in the prostate. Nat Genet 2009;41(5):619-24.
35. Attard G, Swennenhuis JF, Olmos D, et al. Characterization of ERG, AR and PTEN gene status in circulating tumor cells from patients with castration-resistant prostate cancer. Cancer Res 2009;69(7):2912-8.
36. Haffner MC, Aryee MJ, Toubaji A, et al. Androgen-induced TOP2B-mediated double-strand breaks and prostate cancer gene rearrangements. Nat Genet 2010;42(8):668-75.
37. Haffner MC, De Marzo AM, Meeker AK, Nelson WG, Yegnasubramanian S. Transcription-induced DNA double strand breaks: both oncogenic force and potential therapeutic target? Clin Cancer Res 2011;17(12):3858-64.
38. Lin C, Yang L, Tanasa B,et al. Nuclear receptor-induced chromosomal proximity and DNA breaks underlie specific translocations in cancer Cell 2009;139(6):1069-83.
39. Shen WH, Balajee AS, Wang J, et al. Essential Role for Nuclear PTEN in Maintaining Chromosomal Integrity. Cell 2007;128(1):157-170.
40. Gupta A, Yang Q, Pandita RK, et al. Cell cycle checkpoint defects contribute to genomic instability in PTEN deficient cells independent of DNA DSB repair. Cell Cycle 2009;8(14):2198-210.
41. Mendes-Pereira AM, Martin SA, Brough R, et al. Synthetic lethal targeting of PTEN mutant cells with PARP inhibitors. EMBO Mol Med 2009;1(6-7):315-22.
42. Dedes KJ, Wetterskog D, Mendes-Pereira AM,et al. PTEN deficiency in endometrioid endometrial adenocarcinomas predicts sensitivity to PARP inhibitors. Sci Transl Med 2010;2(53):53ra75.
43. McEllin B, Camacho CV, Mukherjee B, et al. PTEN loss compromises homologous recombination repair in astrocytes: implications for glioblastoma therapy with temozolomide or poly(ADP-ribose) polymerase inhibitors. Cancer Res 2010;70(13):5457-64.

44. Brenner JC, Ateeq B, Li Y, Yocum AK,et al. Mechanistic rationale for inhibition of poly(ADP-ribose) polymerase in ETS gene fusion-positive prostate cancer. Cancer Cell 2011;19(5):664-78.
45. de Bono J, Sandhu S, Attard G. Beyond hormone therapy for prostate cancer with PARP inhibitors. Cancer Cell 2011;19(5):573-4.
46. Otto H, Reche PA, Bazan F, et al. In silico characterization of the family of PARP-like poly(ADP-ribosyl)transferases (pARTs). BMC Genomics 2005;6:139.
47. Virag L and Szabo C. The therapeutic potential of poly(ADP-Ribose) polymerase inhibitors. Pharmacol Rev 2002; 54(3): 375-429.
48. Nguewa PA, Fuertes MA, Valladares et al. Poly(ADP-Ribose) Polymerases : Homology, structural domains and functions. Novel Therapeutical Applications. Progr. Biophys. Mol. Biol. 2005; 88(1): 143-172.
49. Delaney CA, Wang LZ, Kyle S, et al. Potentiation of temozolomide and topotecan growth inhibition and cytotoxicity by novel poly(adenosine diphosphoribose)polymerase inhibitors in a panel of human tumor lines. Clin Cancer Res 2000;6:2860-2867.
50. Audeh MW, Carmichael J, Penson RT,et al. Oral poly(ADP-ribose) polymerase inhibitor olaparib in patients with BRCA1 or BRCA2 mutations and recurrent ovarian cancer: a proof-of-concept trial. Lancet 2010;376(9737):245-51.
51. Tutt A, Robson M, Garber JE,et al. Oral poly(ADP-ribose) polymerase inhibitor olaparib in patients with BRCA1 or BRCA2 mutations and advanced breast cancer: a proof-of-concept trial. Lancet 2010;376(9737):235-44.
52. Farmer H, McCabe N, Lord CJ, et al. Targeting the DNA repair defect in BRCA mutant cells as a therapeutic strategy. Nature 2005; 434(7035):917-20.
53. Bryant HE, Schultz N, Thomas HD,et al. Specific killing of BRCA2-deficient tumours with inhibitors of poly(ADP-ribose) polymerase.Nature.2005;434(7035):913-7.
54. Rhiem K, Todt U, Wappenschmidt B,et al. Sporadic breast carcinomas with somatic BRCA1 gene deletions share genotype/phenotype features with familial breast carcinomas. Anticancer Res. 2010;30(9):3445-9.
55. Marsit CJ, Liu M, Nelson HH et al. Inactivation of the Fanconi anemia/BRCA pathway in lung and oral cancers: implications for treatment and survival. Oncogene 2004;23: 1000-1004.
56. Turner N, Tutt A and Ashworth A. Hallmarks of ‘BRCAness’ in sporadic cancers. Nature Rev Cancer 2004; 4(10):1-6.
57. Narayan G, Arias-Pulido H, Nandula SV et al. Disruption of Fanconi anemia-BRCA pathway in cervical cancer. Cancer Res 2004; 64: 2994-2007.
58. Hennessy BT, Timms KM, Carey MS,et al. Somatic mutations in BRCA1 and BRCA2 could expand the number of patients that benefit from poly (ADP ribose) polymerase inhibitors in ovarian cancer. J Clin Oncol 2010;28(22):3570-6.
59. Bednarz N, Eltze E, Semjonow A, Rink M,et al. BRCA1 loss preexisting in small subpopulations of prostate cancer is associated with advanced disease and metastatic spread to lymph nodes and peripheral blood. Clin Cancer Res 2010;16(13):3340-8.
60. McCabe N, Turner NC, Lord CJ, et al. Deficiency in the repair of DNA damage by homologous recombination and sensitivity to poly(ADP-ribose) polymerase inhibition. Cancer Res 2006;66(16):8109-15.
61. Weston VJ, Oldreive CE, Skowronska A,et al. The PARP inhibitor olaparib induces significant killing of ATM-deficient lymphoid tumor cells in vitro and in vivo. Blood. 2010;116(22):4578-87.
62. K. A. Gelmon, H. W. Hirte, A. Robidoux, et al. Can we define tumors that will respond to PARP inhibitors? A phase II correlative study of olaparib in advanced serous ovarian cancer and triple-negative breast cancer. Proceedings of ASCO 2010.
63. J. A. Ledermann, P. Harter, C. Gourley, et al. Phase II randomized placebo-controlled study of olaparib (AZD2281) in patients with platinum-sensitive relapsed serous ovarian cancer (PSR SOC). Proceedings of ASCO 2011.
64. Dong X, Wang L, Taniguchi K,et al. Mutations in CHEK2 associated with prostate cancer risk. Am J Hum Genet. 2003;72(2):270-80.

65. Cybulski C, Huzarski T, Górski B, et al. A novel founder CHEK2 mutation is associated with increased prostate cancer risk. Cancer Res 2004;64(8):2677-9.
66. Cybulski C, Górski B, Debniak T,et al. NBS1 is a prostate cancer susceptibility gene. Cancer Res 2004;64(4):1215-9
67. Agalliu I, Karlins E, Kwon EM, et al. Rare germline mutations in the BRCA2 gene are associated with early-onset prostate cancer. Br J Cancer 2007;97(6):826-31.
68. Willems AJ, Dawson SJ, Samaratunga H,et al. Loss of heterozygosity at the BRCA2 locus detected by multiplex ligation-dependent probe amplification is common in prostate cancers from men with a germline BRCA2 mutation. Clin Cancer Res 2008; 14(10) :2953-61.
69. Kote-Jarai Z, Jugurnauth S, Mulholland S,et al. A recurrent truncating germline mutation in the BRIP1/FANCJ gene and susceptibility to prostate cancer. Br J Cancer 2009;100(2):426-30.
70. Edwards SM, Kote-Jarai Z, Meitz J,et al. Two percent of men with early-onset prostate cancer harbor germline mutations in the BRCA2 gene. Am J Hum Genet 2003;72(1):1-12.
71. Cristofanilli M, Budd GT, Ellis MJ, et al. Circulating tumor cells, disease progression, and survival in metastatic breast cancer. N Engl J Med 2004 ;351(8):781-91.
72. Budd GT, Cristofanilli M, Ellis MJ,et al. Circulating tumor cells versus imaging--predicting overall survival in metastatic breast cancer. Clin Cancer Res 2006;12(21):6403-9.
73. Hayes DF, Cristofanilli M, Budd GT,et al. Circulating tumor cells at each follow-up time point during therapy of metastatic breast cancer patients predict progression-free and overall survival. Clin Cancer Res. 2006;12(14 Pt 1):4218-24.
74. Cohen SJ, Punt CJ, Iannotti N,et al. Relationship of circulating tumor cells to tumor response, progression-free survival, and overall survival in patients with metastatic colorectal cancer. J Clin Oncol. 2008;26(19):3213-21.
75. Moreno JG, Miller MC, Gross S,et al. Circulating tumor cells predict survival in patients with metastatic prostate cancer. Urology 2005;65(4):713-8.
76. de Bono JS, Scher HI, Montgomery RB. Circulating tumor cells predict survival benefit from treatment in metastatic castration-resistant prostate cancer. Clin Cancer Res 2008;14(19):6302-9.
77. Olmos D, Arkenau HT, Ang JE, et al. Circulating tumour cell (CTC) counts as intermediate end points in castration-resistant prostate cancer (CRPC): a single-centre experience. Ann Oncol. 2009;20(1):27-33.
78. H. I. Scher, G. Heller, A. Molina, T , et al. Evaluation of circulating tumor cell (CTC) enumeration as an efficacy response biomarker of overall survival (OS) in metastatic castration-resistant prostate cancer (mCRPC): Planned final analysis (FA) of COU-AA-301, a randomized double-blind, placebo-controlled phase III study of abiraterone acetate (AA) plus low-dose prednisone (P) post docetaxel. Proceedings of ASCO 2011.
79. de Bono JS, Attard G, Adjei A, et al. Potential applications for circulating tumor cells expressing the insulin-like growth factor-I receptor. Clin Cancer Res 2007;13(12):3611-6.
80. Leversha MA, Han J, Asgari Z,et al. Fluorescence in situ hybridization analysis of circulating tumor cells in metastatic prostate cancer. Clin Cancer Res 2009;15(6):2091-7.
81. Scher, HI, Morris MJ, Kelly WK, Schwartz LH, Heller G. Prostate cancer clinical trial end points: "RECIST"ing a step backwards. Clin Cancer Res 2005;11: 5223-32.
82. Pollen JJ, Witztum KF, Ashburn WL. The flare phenomenon on radionuclide bone scan in metastatic prostate cancer. AJR Am J Roentgenol 1984; 142:773-6.
83. Scher HI, Warren M, Heller G. The association between measures of progression and survival in castrate-metastatic prostate cancer. Clin Cancer Res 2007;13:1488-92.
84. Armstrong, AJ, Garrett-Mayer ES, Yang YC, et al. A contemporary prognostic nomogram for men with hormone-refractory metastatic prostate cancer: a TAX327 study analysis. Clin Cancer Res 2007b;13:6396-403.
85. Berthold DR, Pond G, De Wit R, Eisenberger M, and Tannock IF. Association of pain and quality of life (QOL) response and survival of patients (pts) with metastatic hormone refractory prostate

cancer (mHRPC) treated with docetaxel or mitoxantrone in the TAX-327 study. Journal of Clin Oncol 2006;24:4516.

86. Paice JA and Cohen FL. Validity of a verbally administered numeric rating scale to measure cancer pain intensity. Cancer Nurs 1997;20:88-93.
87. Small EJ, Meyer M, Marshall ME, Reyno LM, et al. Suramin therapy for patients with symptomatic hormone-refractory prostate cancer: results of a randomized Phase 3 trial comparing suramin plus hydrocortisone to placebo plus hydrocortisone. J Clin Oncol 2000;18:1440-50.
88. Weinfurt KP, Anstrom KJ, Castel LD, Schulman KA and Saad F. Effect of zoledronic acid on pain associated with bone metastasis in patients with prostate cancer. Ann Oncol 2006;17:986-9.
89. Cleeland CS and Ryan KM. Pain assessment: global use of the Brief Pain Inventory. Ann Acad Med Singapore 1994;23:129-38.
90. Sandblom G, Carlsson P, Sennfalt K, Varenhorst E. A population-based study of pain and quality of life during the year before death in men with prostate cancer. Br J Cancer 2004;90:1163-8.
91. Leone G, Mele L, Pulsoni A, Equitani F, Pagano L.The incidence of secondary leukemias.Haematologica. 1999;84(10):937-45.
92. Pedersen-Bjergaard J, Nissen NI, et al.Acute non-lymphocytic leukemia in patients with ovarian carcinoma following long-term treatment with Treosulfan (=dihydroxybusulfan).Cancer.1980;45(1):19-29.
93. Camus P, Bonniaud P, Fanton A, Camus C, Baudaun N, Foucher P.Drug-induced and iatrogenic infiltrative lung disease.Clin Chest Med. 2004;25(3):479-519.
94. Christensen S, Pedersen L, Grijota M, Kornum JB, Beiderbeck A, Sørensen HT.Incidence of interstitial pneumonitis among breast cancer patients: a 10-year Danish population-based cohort study.BrJCancer.2008;98(11):1870-5.
95. Simon R, Wittes RE and Ellenberg SS, Randomized phase II clinical trials. Cancer treatment reports, 1985. 69(12): p. 1375-81.
96. Yap C,. Pettitt A, and Billingham L, Screened selection design for randomised phase II oncology trials: an example in chronic lymphocytic leukaemia. BMC Medical Research Methodology, 2013. 13(1): p. 87
97. Mateo J, Friedlander M, Sessa C, Leunen K, Nicum S, Gourley C, Fielding A, Bowen K, Kaye S, Molife LR Administration of continuous/intermittent olaparib in ovarian cancer patients with a germline BRCA1/2 mutation to determine an optimal dosing schedule for the tablet formulation. European Journal of Cancer. 2013 Sep;49:S161-S161.

23 APPENDICES

23.1 Appendix A: Part A - Detectable Effects

It is possible to calculate detectable effects in the sensitive group (80% power, y-axis) based on the proportion of sensitive patients (individual lines) and the response rate (x-axis) in the insensitive subgroup. Also shown is the probability a subgroup effect is detected (dashed lines), with 80% power this probability cannot exceed 80%. The fall in the probability of detecting a subgroup effect with increasing response rate in insensitive patients is therefore balanced by an increase in the probability of detecting an overall effect. Thus, in the extreme, if the response rate in insensitive patients is 45% and the response rate in sensitive patients is about 50% a conclusion of overall efficacy will be reached with 80% power, for subgroups of size 30% or more.

Using cut offs for Part A of a_0=15/30, a_1=23/45, as intended.

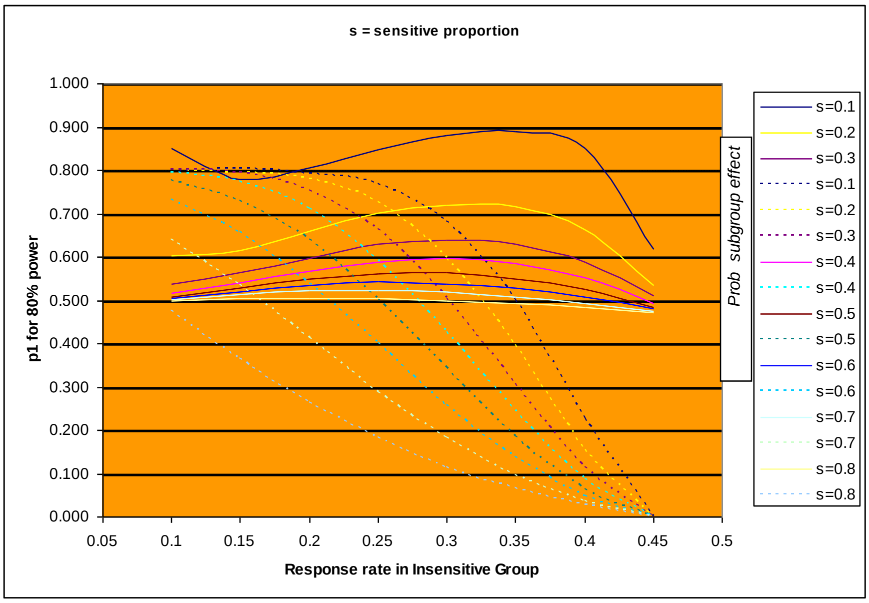

Using cut offs for Part A of a_0=18/30, a_1=27/45 the probability of detecting a subgroup effect if the sensitive group is large (>50%) can be improved.

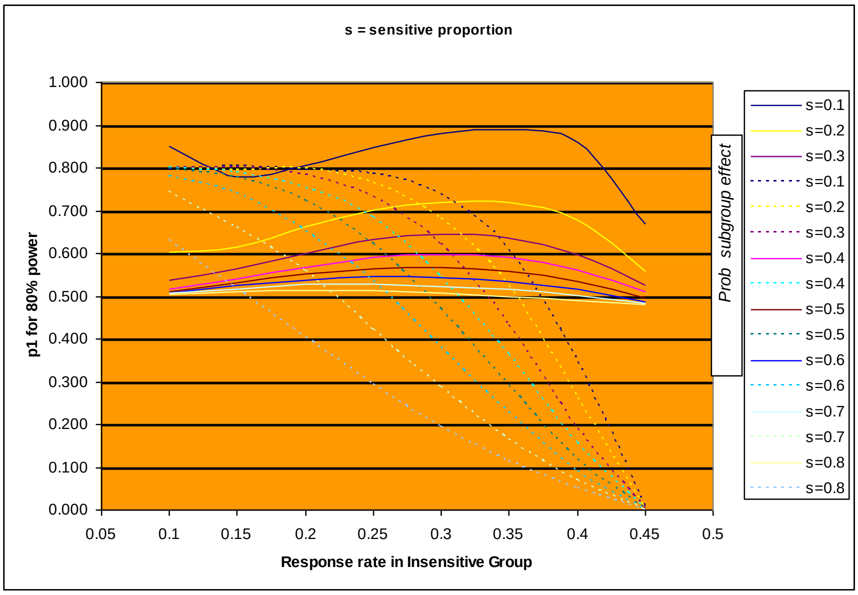

23.2 Appendix B: Sample Size Calculations

TOPARP sample size calculations, **Part A** : alpha 2.2%, Power 90%

Ho : Null Hypothesis Response rate (p0) is 5%

Probability reject **0.97818** *alpha* 2.2%

Reject first stage 0.81218 (Probability of early termination, sum of values in light blue)

Reject second stage 0.166 (sum of values in dark blue)

sum 0.97818

N First	po	N second stage 0	1	2	3	4	5	6	7	8	……	15
	0.05	0.46329	0.3657562	0.13475	0.03073	0.00485	0.00056	4.9E-05	3.3E-06	1.8E-07	……	3.1E-20
0	0.214639											
1	0.338903											
2	0.258637											
3	0.12705	0.059	0.046	0.017	0.004	0.001	0	0	0	0		0
4	0.045136	0.021	0.017	0.006	0.001	0	0	0	0	0		0
5	0.012353	0.006	0.005	0.002	0	0	0	0	0	0		0
6	0.002709	0.001	0.001	0	0	0	0	0	0	0		0
7	0.000489	0	0	0	0	0	0	0	0	0		0
8	7.4E-05	0	0	0	0	0	0	0	0	0		0
9	9.52E-06	0	0	0	0	0	0	0	0	0		0
10	1.05E-06	0	0	0	0	0	0	0	0	0		0
11	1.01E-07	0	0	0	0	0	0	0	0	0		0
12	8.39E-09	0	0	0	0	0	0	0	0	0		0
13	6.11E-10	0	0	0	0	0	0	0	0	0		0
14	3.91E-11	0	0	0	0	0	0	0	0	0		0
15	2.19E-12											
16	1.08E-13											
17	4.69E-15											
18	1.78E-16											
….												
26	3.33E-30											
27	2.59E-32											
28	1.46E-34											
29	5.31E-37											
30	9.31E-40											

Stage I 2.31E-12
probability of incorrect stopping

H1 : Null Hypothesis Response rate (p1) is 20%

Probability reject **0.10318** *Power* 89.7%

Reject first stage 0.04418 (Probability of early termination, sum of values in light blue)

Reject second stage 0.059 (sum of values in dark blue)

N First	po	N second stage 0	1	2	3	4	5	6	7	8	……	15
	0.2	0.03518	0.1319414	0.2309	0.25014	0.1876	0.10318	0.04299	0.01382	0.00345	……	3.3E-11
0	0.001238											
1	0.009285											
2	0.033656											
3	0.078532	0.003	0.01	0.018	0.02	0.015	0.008	0.003	0.001	0		0
4	0.132522	0.005	0.017	0.031	0.033	0.025	0.014	0.006	0.002	0		0
5	0.172279	0.006	0.023	0.04	0.043	0.032	0.018	0.007	0.002	0.001		0
6	0.179457	0.006	0.024	0.041	0.045	0.034	0.019	0.008	0.002	0.001		0
7	0.153821	0.005	0.02	0.036	0.038	0.029	0.016	0.007	0.002	0.001		0
8	0.110559	0.004	0.015	0.026	0.028	0.021	0.011	0.005	0.002	0		0
9	0.067564	0.002	0.009	0.016	0.017	0.013	0.007	0.003	0.001	0		0
10	0.035471	0.001	0.005	0.008	0.009	0.007	0.004	0.002	0	0		0
11	0.016123	0.001	0.002	0.004	0.004	0.003	0.002	0.001	0	0		0
12	0.006382	0	0.001	0.001	0.002	0.001	0.001	0	0	0		0
13	0.002209	0	0	0.001	0.001	0	0	0	0	0		0
14	0.000671	0	0	0	0	0	0	0	0	0		0
15	0.000179											
16	4.19E-05											
17	8.63E-06											
18	1.56E-06											
….												
26	7.53E-15											
27	2.79E-16											
28	7.47E-18											
29	1.29E-19											
30	1.07E-21											

Stage I 0.000231
probability of incorrect stopping

TOPARP sample size calculations, **Part A**, rule to be applied if no subgroup is found:

Ho : Null Hypothesis Response rate (p0) is 30%

		Reject first stage	0.94505	alpha	5.5%
**N**	**p0**		(sum of values in light blue)		
	0.3				
0	1.07E-07	16	0.0896266	32	1.3E-08
1	2.06E-06	17	0.0655253	33	2.2E-09
2	1.95E-05	18	0.0436835	34	3.3E-10
3	0.00012	19	0.0266043	35	4.5E-11
4	0.000538	20	0.0148224	36	5.4E-12
5	0.00189	21	0.0075624	37	5.6E-13
6	0.005401	22	0.0035357	38	5E-14
7	0.012895	23	0.0015153	39	3.9E-15
8	0.026251	24	0.0005953	40	2.5E-16
9	0.046252	25	0.0002143	41	1.3E-17
10	0.071361	26	7.065E-05	42	5.3E-19
11	0.09731	27	2.131E-05	43	1.6E-20
12	0.118162	28	5.87E-06	44	3.1E-22
13	0.12855	29	1.475E-06	45	3E-24
14	0.125927	30	3.371E-07		
15	0.111535	31	6.991E-08		

Ha : Alternate Hypothesis Response rate (p1) is 50%

		Reject first stage	0.11635	Power	88.4%
**N**	**p1**				
	0.5				
0	2.84E-14	16	0.0183782	32	0.00207
1	1.28E-12	17	0.0313511	33	0.00082
2	2.81E-11	18	0.0487684	34	0.00029
3	4.03E-10	19	0.0693024	35	9.1E-05
4	4.23E-09	20	0.0900931	36	2.5E-05
5	3.47E-08	21	0.1072537	37	6.1E-06
6	2.31E-07	22	0.1170041	38	1.3E-06
7	1.29E-06	23	0.1170041	39	2.3E-07
8	6.13E-06	24	0.1072537	40	3.5E-08
9	2.52E-05	25	0.0900931	41	4.2E-09
10	9.07E-05	26	0.0693024	42	4E-10
11	0.000288	27	0.0487684	43	2.8E-11
12	0.000817	28	0.0313511	44	1.3E-12
13	0.002075	29	0.0183782		
14	0.004743	30	0.0098017		
15	0.009802	31	0.0047428		

TOPARP sample size calculations, **Part B** – each dose group (one-stage A'Hern Design):

Ho : Null Hypothesis Response rate (p0) is 30%

N	p0	N		N	
	0.3	Reject first stage	0.95628 (sum of values in light blue)	alpha	5%
0	1.53E-07	16	0.0825133	32	5.4E-09
1	2.88E-06	17	0.0582447	33	8.4E-10
2	2.66E-05	18	0.037443	34	1.2E-10
3	0.000159	19	0.0219591	35	1.4E-11
4	0.0007	20	0.0117638	36	1.5E-12
5	0.0024	21	0.0057619	37	1.4E-13
6	0.006687	22	0.0025816	38	1.1E-14
7	0.015556	23	0.0010583	39	7.4E-16
8	0.030835	24	0.0003969	40	4E-17
9	0.05286	25	0.0001361	41	1.7E-18
10	0.07929	26	4.261E-05	42	5.1E-20
11	0.105033	27	1.218E-05	43	1E-21
12	0.123789	28	3.168E-06	44	9.8E-24
13	0.130591	29	7.491E-07		
14	0.123928	30	1.605E-07		
15	0.106224	31	3.107E-08		

Ha : Alternate Hypothesis Response rate (p1) is 50%

N	p1	N		N	
	0.5	Reject first stage	0.14561	Power	85%
0	5.68E-14	16	0.0236875	32	0.0012
1	2.5E-12	17	0.0390147	33	0.00044
2	5.38E-11	18	0.058522	34	0.00014
3	7.53E-10	19	0.0800828	35	4E-05
4	7.72E-09	20	0.1001035	36	1E-05
5	6.17E-08	21	0.114404	37	2.2E-06
6	4.01E-07	22	0.1196042	38	4E-07
7	2.18E-06	23	0.114404	39	6.2E-08
8	1.01E-05	24	0.1001035	40	7.7E-09
9	4.03E-05	25	0.0800828	41	7.5E-10
10	0.000141	26	0.058522	42	5.4E-11
11	0.000436	27	0.0390147	43	2.5E-12
12	0.001199	28	0.0236875	44	5.7E-14
13	0.002951	29	0.013069		
14	0.006534	30	0.0065345		
15	0.013069	31	0.0029511		

Assessment of optimal dose level of olaparib in part B:

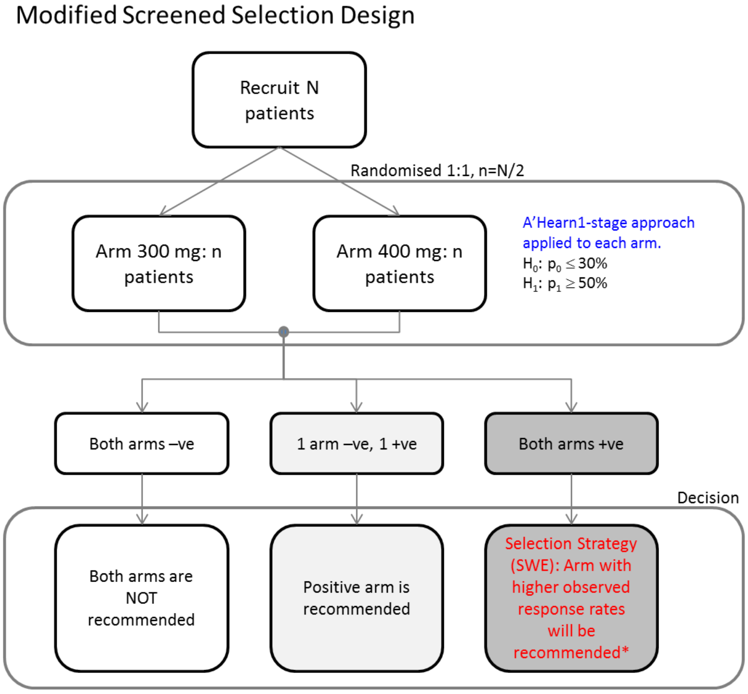

With 44 patients per dose group, assuming that the true response rate for each dose group is over 40% (both dose groups are positive), the probability of choosing the dose group with the highest response rate[1] would be (under different scenarios):

Probability of choosing the group with the highest response rate

scenario	400 mg group #resp	400 mg group %resp	300 mg group #resp	300 mg group %resp	Difference	Pick the winner prob
1	19	43.2%	19	43.2%	0.0%	0.5
2	20	45.5%	19	43.2%	2.3%	0.58472373
3	21	47.7%	19	43.2%	4.5%	0.66537278
4	22	50.0%	19	43.2%	6.8%	0.73891881
5	24	54.5%	19	43.2%	11.4%	0.85695024
6	30	68.2%	19	43.2%	25.0%	0.99165164
7	21	47.7%	21	47.7%	0.0%	0.5
8	22	50.0%	21	47.7%	2.3%	0.58421083
9	24	54.5%	21	47.7%	6.8%	0.73848956
10	26	59.1%	21	47.7%	11.4%	0.85745305
11	28	63.6%	21	47.7%	15.9%	0.93419277
12	30	68.2%	21	47.7%	20.5%	0.97501485
13	22	50.0%	22	50.0%	0.0%	0.5
14	24	54.5%	22	50.0%	4.5%	0.66489082
15	26	59.1%	22	50.0%	9.1%	0.80391727
16	28	63.6%	22	50.0%	13.6%	0.90214408
17	30	68.2%	22	50.0%	18.2%	0.95954131

The previous table show that, if both dose groups are positive, and the difference in response rate between dose groups is less than 10%, the probability of picking the dose with the true higher response rate is <80%. In that case, the dose selection would be based not only in response rate but also in duration of responses, tolerability and drug exposure data.

[1] Computed using online calculator http://www.cct.cuhk.edu.hk/stat/phase2/Randomized.htm

23.3 Appendix C: Evidence of PD to Determine Trial Entry Eligibility

Patients being considered for trial entry should have evidence of disease progression by either PSA, modified RECIST1.1 (appendix E) or bone scan to be eligible.

PSA: PSA evidence of disease progression based on the prostate cancer working group (PCWG) 2 criteria consist of a minimum PSA level 2.0 ng/ml that has risen on at least 2 successive occasions, at least 1 weeks apart. The reference value # 1 is the last value before the rise in PSA was observed. If the confirmatory PSA value (Figure 5, # 3A) is greater than the screening value then progression by PSA is met and the patient is eligible for trial enrolment on the basis of PSA alone. If the confirmatory PSA (Figure 5, #3B) value is less than the screening PSA (Figure 5, #2) value, then an additional test for rising PSA (#4) will be required to document progression before the patient can be enrolled.

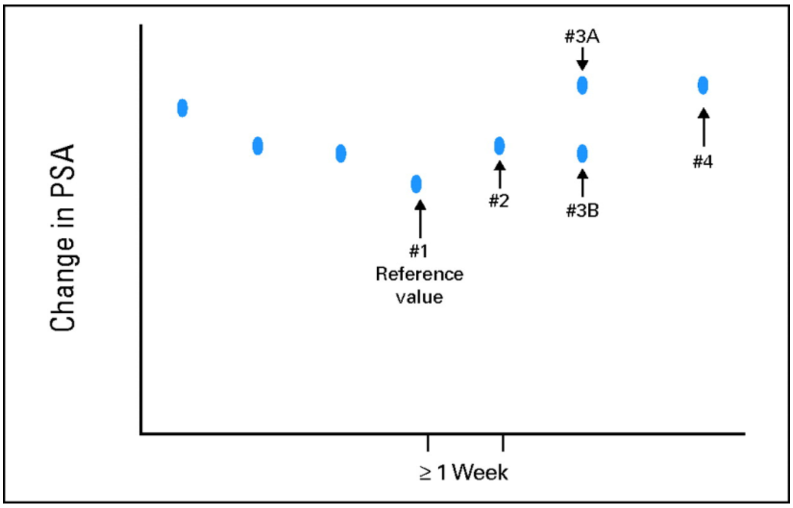

Figure 5: Eligibility Based on PSA

Target lesion/measurable disease: Patients are not required to have evidence of disease progression by measurable disease if they meet the criteria for disease progression on the basis of PSA or bone scan. Evidence of nodal or visceral disease RECIST progression however is sufficient for trial entry independent of PSA readings. Because lymph nodes may be enlarged due to benign pathology, only lymph nodes that are ≥ 2cm should be used for disease evaluation.

Bone scan: Evidence of disease progression based on bone scan appearance is sufficient for trial entry independent of PSA readings. If the appearance of the bone scan is the only indicator of progression then there must be ≥ 2 new bone lesions compared with the prior bone scans. If there is ambiguity about the appearance of the bony lesions such as traumatic in nature or secondary to a flare reaction then it is recommended that an alternative imaging modality such as MRI or fine-cut CT be used to evaluate these lesions further.

23.4 Appendix D: Outcome Measures of Response / Progression Post Study Treatment

Procedures for Assessing PSA Response / Progression Post Study Treatment

PSA measurements will be done every 12 weeks on day1 of cycle 4,7,10 and every 3rd cycle thereafter. Increases and decreases will be tracked in order to assess disease response. The PSA readings on its own will not be used to define disease progression in this protocol. PSA response and PSA progression will be defined according to the consensus guidelines of the PCWG 2:

- PSA partial response is defined as a ≥ 50% decline in PSA from cycle1 day1 (baseline) PSA value. This PSA decline must be confirmed to be sustained by a second PSA value obtained 4 or more weeks later.
- PSA progression date is defined as the date that a ≥ 25% increase and an absolute increase of ≥ 2 ng/mL above the nadir (minimum value observed so far) is documented, which is confirmed by a second consecutive value obtained four or more weeks later. The first PSA reading will be obtained at week 12.

Procedures for Assessing Radiological Responses / Progression Post Study Treatment

Categorisation of response in this study will be based on modified RECIST1.1 (appendix E) for soft tissue disease and appearance of bone scan for bone metastasis.

Bone metastasis evaluation:

The first bone scan should not be performed before 12 weeks post commencement of treatment unless clinically indicated as there may be evidence of a flare response early on which may confuse the response evaluation. A patient will be considered to have progressed on bone scan if the following criteria are met:

- If the first bone scan with 2 new lesions is observed ≥12 weeks and ≤13 weeks from the point of Cycle1 Day1 and is confirmed by a follow up scan ≥ 6 weeks later showing 2 additional new lesions (i.e. a total of ≥ 4 new lesions compared with the baseline bone scan).
- The first bone scan with ≥ 2 new lesions compared with baseline is observed > 13 weeks from Cycle 1 Day1 and the new lesions are verified on the next bone scan ≥ 6 weeks later (i.e. a total of ≥ 2 new lesions compared with baseline). If the second scan confirms progression the data of the progression is the date of the first bone scan rather than the second confirmatory scan.

Measurable Disease Evaluation:
Changes in nodal and visceral disease should be recorded using the modified RECIST1.1 criteria defined in Appendix E.

Table 9: Radiological Criteria for Ascribing Disease Progression

**Evidence of Progression**	**Confirmation**	**Action**
Bone Disease Appearance of two or more new bone lesions on bone scan	≥ 2 new lesions at the first scheduled reassessment ≥12 weeks and ≤13 weeks from Cycle 1 Day 1 compared with baseline must be confirmed by a second scan performed 6 or more weeks later. Confirmatory scans should show an additional 2 new lesions compared to the first post treatment scan (i.e. a total of ≥ 4 new lesions compared with the baseline bone scan).	Investigators are highly encouraged to maintain the patient's treatment with study medication unless progression is confirmed.
Appearance of two or more new bone lesions on bone scan	≥ 2 new lesions at the first scheduled reassessment >13 weeks from Cycle 1 Day 1 compared with baseline must be confirmed by a second scan performed 6 or more weeks later. Confirmatory scans should confirm the presence of the 2 new lesions compared the baseline scan. (i.e. a total of ≥ 2 new lesions compared with the baseline bone scan).	Investigators are highly encouraged to maintain the patient's treatment with study medication unless progression is confirmed.
Soft Tissue Disease as defined by RECIST on CT/MRI	Progression at any scheduled reassessment ≥ 12 weeks does not need to be confirmed.	Investigators are highly encouraged to maintain the patient's treatment with study medication unless radiological progression is observed.

23.5 Appendix E: Modified RECIST1.1

The primary efficacy objective to this trial is based on tumour assessment. For the purpose of this study, definitions of soft tissue tumour assessments will be based on a Modified Response Evaluation Criteria in Solid Tumours 1.1 (RECIST1.1). Pertinent points are summarised below.

Evaluation of measurable and non-measurable lesions

1. **Measurable lesions** – lesions that can be accurately measured in at least one dimension with the longest diameter ≥ 20 mm using conventional techniques or ≥ 10 mm, with a spiral CT scan.

2. **Non-measurable lesions –** all other lesions, including small lesions (longest diameter ≤ 10 mm, with a spiral CT scan), i.e., blastic bone lesions, leptomeningeal disease, ascites, pleural or pericardial effusion, inflammatory breast disease, lymphangitic involvement of skin or lung, abdominal masses/abdominal organomegaly identified by physical exam that is not measurable by reproducible imaging techniques.

3. Tumour lesions situated in a previously irradiated area, or in an area subjected to other loco-regional therapy, should not be included in assessment of response, but should be assessed for progression.

4. For this study, bone lesions are not considered "non-measurable" lesions for RECIST. Bone lesions will be assessed by bone scan only.

5. All measurements should be taken and recorded in metric notation, using a ruler or calipers. Preferably, all screening evaluations should be performed as closely as possible to the beginning of treatment and never more than 4 weeks before the beginning of treatment.

6. The same method of assessment and the same technique should be used to characterise each identified and reported lesion at baseline and during follow up.

7. Clinical lesions will only be considered measurable when they are superficial (e.g., skin nodules and palpable lymph nodes). For the case of skin lesions, documentation by colour photography, including a ruler to estimate the size of the lesion is recommended.

Methods of measurement:

1. CT and MRI are the best currently available and reproducible methods to measure target lesions selected for response assessment. Conventional CT and MRI should be performed with cuts of 10 mm or less in slice thickness. Spiral CT should be performed using a 5-mm contiguous reconstruction algorithm. This applies to tumours of the chest, abdomen and pelvis. Head and neck tumours and those of extremities usually require specific protocols.

2. Lesions on chest X-ray are acceptable as measurable lesions when they are clearly defined and surrounded by aerated lung, however CT scans are preferable.

3. Ultrasound (US), endoscopy and laparoscopy should not be used to measure tumour lesions. It is however a possible alternative to clinical measurements of superficial palpable lymph nodes, subcutaneous lesions and thyroid nodules. Ultrasound may also be used to confirm the complete disappearance of superficial lesions usually assessed by clinical examination.

4. Cytology and histology can be used to differentiate between PR and CR in rare cases (e.g., after treatment to differentiate between residual benign lesions and residual malignant lesions.

Baseline documentation of target and non-target lesions

1. All measurable lesions up to a maximum of 2 lesions per organ and 5 lesions in total representative of all involved organs should be identified as target lesions and recorded and measured at baseline and at the time points specified in the protocol.

2. Target lesions should be selected on the basis of their size (lesions with the longest diameter) and their suitability for accurate repeated measurements.

3. A sum of the longest diameters (LD) for all target lesions will be calculated and reported as the baseline sum of LD. The baseline sum LD will be used as reference by which to characterise the objective tumour response.

4. All other lesions (or sites of disease) should be identified as non-target lesions and should also be recorded at baseline. Non-target lesions will also be monitored throughout the study, and an overall assessment of response will be made. Details of any new lesions will also be recorded. It is possible to record multiple nontarget lesions involving the same organ as a single item on the case record form (e.g. “multiple enlarged pelvic lymph nodes” or “multiple liver metastases”).

Table 10: Response Criteria

**Response criteria**	**Evaluation of target lesions**
Complete Response (CR)	Disappearance of all target lesions
Partial Response (PR)	At least 30% decrease in the sum of LD of target lesions, taking as reference the baseline sum of LD.
Progressive Disease (PD)	At least 20% increase in the sum of LD of target lesions, taking as reference the smallest sum LD recorded since the treatment started or the appearance of one or more new lesions.
Stable Disease (SD)	Whether sufficient shrinkage to qualify for PR nor sufficient increase to qualify for PD, taking as reference the smallest sum LD since the treatment started.

Table 10: Responses in patients with both target and non-target disease

Target lesions	Non-target lesions	New Lesions	Overall response
CR	CR	No	CR
CR	Non-CR/nonPD	No	PR
CR	Not evaluated	No	PR
PR	Non-PD or not all evaluated	No	PR
SD	Non-PD or not all evaluated	No	SD
Not all evaluated	Non-PD	No	NE
PD	Any	Yes or No	PD
Any	PD	Yes or No	PD
Any	Any	Yes	PD

CR = complete response, PR= partial response, SD = stable disease, PD= progressive disease, NE= inevaluable

Table 11: Responses in patients with only non-target disease

Non-target Lesions	New Lesions	Overall Response
CR	No	CR
Non-CR/nonPD	No	Non-CR/nonPD*
Not all evaluated	No	NE
Unequivocal PD	Yes or No	PD
Any	Yes	PD

CR = complete response, PR= partial response, SD = stable disease,
PD= progressive disease, NE= inevaluable

Reproduced from: http://www.recist.com/recist-comparative/01.html and Eisenhauer EA, Therasse P, Bogaerts J et al. New response evaluation criteria in solid tumours: Revised RECIST guideline (version 1.1). Eur J Cancer 2009; 45:228-247.

Patients with a global deterioration of health status requiring discontinuation of treatment without objective evidence of disease progression at that time should be classified as having "symptomatic deterioration". Every effort should be made to document the objective progression even after discontinuation of treatment.

In some circumstances it may be difficult to distinguish residual disease from normal tissue. When the evaluation of complete response depends on this determination, it is recommended that the residual lesion be investigated (fine needle aspirate/biopsy) to confirm the complete response status.

Confirmation
To be assigned a status of PR or CR, changes in tumour measurements must be confirmed by repeat assessments. This should be conducted at the next study visit that is at least 4 weeks after the criteria for response was met.

In the case of SD, follow-up measurements must have met the SD criteria at least once after study entry at a minimum interval (in general, not less than 12 weeks) that is defined in the study protocol.

Evaluation of best overall response

The best overall response is the best response recorded from the start of the treatment until disease progression/recurrence (taking as reference for PD the smallest measurements recorded since the treatment started). In general, the patient's best response assignment will depend on the achievement of both measurement and confirmation criteria.

Duration of overall response

The duration of overall response is measured from the time measurement criteria are met for CR or PR (whichever status is recorded first) until the first date that recurrence or PD is objectively documented, taking as reference for PD the smallest measurements recorded since the treatment started.

Duration of stable disease

SD is measured from the start of the treatment until the criteria for disease progression are met, taking as reference the smallest measurements recorded since the treatment started.

23.6 Appendix F: ECOG Performance Status

Table 12: ECOG Performance Status

ECOG Performance Status	Function
0	Fully active, able to carry on all pre-disease performance without restriction
1	Restricted in physically strenuous activity but ambulatory and able to carry out work of a light or sedentary nature, e.g., light house work, office work
2	Ambulatory and capable of all self-care but unable to carry out any work activities. Up and about more than 50% of waking hours
3	Capable of only limited self-care, confined to bed or chair more than 50% of waking hours
4	Completely disabled. Cannot carry on any self-care. Totally confined to bed or chair
5	Dead

23.7 Appendix G: National Cancer Institute Common Terminology Criteria for Adverse Events (version 4.02)

V4.02 (CTCAE): publish date August 9, 2010:
ctep.cancer.gov/reporting/ctc.html

23.8 Appendix H: World Medical Association Declaration of Helsinki

http://www.wma.net/e/policy/b3.html

23.9 Appendix I: Brief Pain Inventory (Short Form)

1903

PLEASE USE BLACK INK PEN

Date: ____ / ____ / ____ (month) (day) (year)

Subject's Initials : ____________

Study Subject #: ____

Study Name: ____________________

Protocol #: ____________________

PI: ____________________

Revision: 07/01/05

Brief Pain Inventory (Short Form)

1. Throughout our lives, most of us have had pain from time to time (such as minor headaches, sprains, and toothaches). Have you had pain other than these everyday kinds of pain today?

☐ Yes ☐ No

2. On the diagram, shade in the areas where you feel pain. Put an X on the area that hurts the most.

Front Back

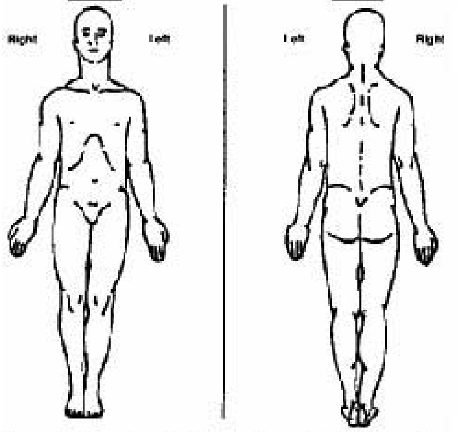

3. Please rate your pain by marking the box beside the number that best describes your pain at its worst in the last 24 hours.

☐ 0 ☐ 1 ☐ 2 ☐ 3 ☐ 4 ☐ 5 ☐ 6 ☐ 7 ☐ 8 ☐ 9 ☐ 10

No Pain — Pain As Bad As You Can Imagine

4. Please rate your pain by marking the box beside the number that best describes your pain at its least in the last 24 hours.

☐ 0 ☐ 1 ☐ 2 ☐ 3 ☐ 4 ☐ 5 ☐ 6 ☐ 7 ☐ 8 ☐ 9 ☐ 10

No Pain — Pain As Bad As You Can Imagine

5. Please rate your pain by marking the box beside the number that best describes your pain on the average.

☐ 0 ☐ 1 ☐ 2 ☐ 3 ☐ 4 ☐ 5 ☐ 6 ☐ 7 ☐ 8 ☐ 9 ☐ 10

No Pain — Pain As Bad As You Can Imagine

6. Please rate your pain by marking the box beside the number that tells how much pain you have right now.

☐ 0 ☐ 1 ☐ 2 ☐ 3 ☐ 4 ☐ 5 ☐ 6 ☐ 7 ☐ 8 ☐ 9 ☐ 10

No Pain — Pain As Bad As You Can Imagine

Page 1 of 2

Copyright 1991 Charles S. Cleeland, PhD
Pain Research Group
All rights reserved

1903

Date: ___ ___ / ___ ___ / ___ ___ (month) (day) (year)

Subject's Initials : ______________

Study Subject #: ___ ___ ___ ___

PLEASE USE BLACK INK PEN

Study Name: ______________________

Protocol #: ______________________

PI: ______________________

Revision: 07/01/05

7. What treatments or medications are you receiving for your pain?

8. In the last 24 hours, how much relief have pain treatments or medications provided? Please mark the box below the percentage that most shows how much relief you have received.

0%	10%	20%	30%	40%	50%	60%	70%	80%	90%	100%
☐ No Relief	☐	☐	☐	☐	☐	☐	☐	☐	☐	☐ Complete Relief

9. Mark the box beside the number that describes how, during the past 24 hours, pain has interfered with your:

A. General Activity

☐ 0 Does Not Interfere ☐ 1 ☐ 2 ☐ 3 ☐ 4 ☐ 5 ☐ 6 ☐ 7 ☐ 8 ☐ 9 ☐ 10 Completely Interferes

B. Mood

☐ 0 Does Not Interfere ☐ 1 ☐ 2 ☐ 3 ☐ 4 ☐ 5 ☐ 6 ☐ 7 ☐ 8 ☐ 9 ☐ 10 Completely Interferes

C. Walking ability

☐ 0 Does Not Interfere ☐ 1 ☐ 2 ☐ 3 ☐ 4 ☐ 5 ☐ 6 ☐ 7 ☐ 8 ☐ 9 ☐ 10 Completely Interferes

D. Normal Work (includes both work outside the home and housework)

☐ 0 Does Not Interfere ☐ 1 ☐ 2 ☐ 3 ☐ 4 ☐ 5 ☐ 6 ☐ 7 ☐ 8 ☐ 9 ☐ 10 Completely Interferes

E. Relations with other people

☐ 0 Does Not Interfere ☐ 1 ☐ 2 ☐ 3 ☐ 4 ☐ 5 ☐ 6 ☐ 7 ☐ 8 ☐ 9 ☐ 10 Completely Interferes

F. Sleep

☐ 0 Does Not Interfere ☐ 1 ☐ 2 ☐ 3 ☐ 4 ☐ 5 ☐ 6 ☐ 7 ☐ 8 ☐ 9 ☐ 10 Completely Interferes

G. Enjoyment of life

☐ 0 Does Not Interfere ☐ 1 ☐ 2 ☐ 3 ☐ 4 ☐ 5 ☐ 6 ☐ 7 ☐ 8 ☐ 9 ☐ 10 Completely Interferes

Copyright 1991 Charles S. Cleeland, PhD
Pain Research Group
All rights reserved

23.10 Appendix J: Total blood volumes drawn at each time point

Part A

Study time points	Months on study	Total volume of safety and research bloods per month
Screening	-1	92.5mls
Cycle 1 Day 1	1	156.5 mls
Cycle 1 Day 8		
Cycle 1 Day 15		
Cycle 2 Day 1	2	74mls
Cycle 3 Day 1	3	76.5mls
Cycle 4 Day 1	4	38mls
Cycle 5 Day 1	5	44.5mls
Cycle 6 Day 1	6	24mls
Cycle 7 Day 1	7	38mls
Cycle 8 Day 1	8	12mls
Cycle 9 Day 1	9	12mls
Cycle 10 Day 1	10	38mls
Cycle 11 Day 1	11	12mls
Cycle 12 Day 1	12	12mls
End of study		92.5mls

Part B

Study time points	Months on study	Safety Bloods	Research Bloods	Total volume of bloods per month
Screening	-1	18mls	73mls	91mls
Cycle 1 Day 1	1	30mls	153mls	183mls
Cycle 1 Day 8				
Cycle 2 Day 1	2	12mls	62mls	74mls
Cycle 3 Day 1	3	12mls	64.5mls	76.5mls
Cycle 4 Day 1	4	18mls	50mls	68mls
Cycle 5 Day 1	5	12mls	32.5mls	44.5mls
Cycle 6 Day 1	6	12mls	42mls	54mls
Cycle 7 Day 1	7	18mls	50mls	68mls
Cycle 8 Day 1	8	12mls	30mls	42mls
Cycle 9 Day 1	9	12mls	30mls	42mls
Cycle 10 Day 1	10	18mls	50mls	68mls
Cycle 11 Day 1	11	12mls	30mls	42mls
Cycle 12 Day 1	12	12mls	30mls	42mls
Dose Escalation (300mg group only)		18mls	52.5mls	70.5mls
End of Treatment		18mls	73mls	91mls

*Please note patients who dose escalate will have PSA checked at day 1 of every cycle instead of every 3 cycles, thus an extra 6ml of blood will be taken for this.

TOPARP

Phase II Trial of Olaparib in Patients with Advanced Castration Resistant Prostate Cancer

Statistical Analysis Plan
Version 3.0

21/01/2019

This statistical analysis plan is based on protocol version 7.0 [15th March 2018]

REC reference no: 11/LO/2019

ICR-CTSU no: ICR-CTSU/2011/10030

CR UK (other funder) no: CRUK/C12540/A12354

CTA reference no:22138/0016/001/0001

EudraCT no: 2011-000601-49

This statistical analysis plan is a framework to guide statistical analysis and may be supplemented by additional and exploratory analyses. Trial statisticians reserve the right to amend analysis methods as appropriate after discussion with the ICR-CTSU Scientific Lead.

This statistical analysis plan has been approved by the following personnel:

Senior Trial Statistician: Nuria Porta

Signed: ________________________

Date: __/__/____

ICR-CTSU Scientific Lead: Emma Hall

Signed: ________________________

Date: __/__/____

Chief Investigator: Johann de Bono

Signed: ________________________

Date: __/__/____

Document history

Version	Author	Date	Changes made
1.0	Eleftheria Kalaitzaki	03/10/2012	First draft
1.1	Eleftheria Kalaitzaki	22/12/14	Draft version. Update on continuing recruitment to stage 2 of part A. Amended wording for confirmation of CTC response.
2.0	Helen Mossop	18/02/2015	Final version. Update on evaluable patient population. Removal of biomarker analysis details.
3.0	David Dolling/Nuria Porta	21/01/2019	Final version. Update for version 7.0 of protocol and introduction of two dose groups to stage B and a screened selection design. Include pre-planned exploratory subgroup analyses for Part-B.

Contents

1. Introduction 5
1.1. Trial design 5
1.2. Study population 6
2. Trial Objectives 10
2.1. Primary Objective 10
2.2. Secondary Objectives 10
2.3. Tertiary/exploratory Objectives 10
3. Sample size & power 10
3.1. Part A (comprised of stages 1 & 2) 10
3.1.1. Stage 1 11
3.1.2. Stage 2 11
3.1.3. Deriving a classifier for identification of tumours with high susceptibility to olaparib 11
3.2. Part B 11
4. Randomisation/ recruitment procedures 12
5. Endpoints 12
5.1. Primary Endpoint 12
5.2. Secondary Endpoints 12
5.3. Tertiary/exploratory Endpoints 13
6. Data completeness and consistency 13
6.1. Patient flow through trial 13
6.2. Compliance with the assessments 13
6.3. Consistency of reporting 13
6.4. Missing data 13
7. Analysis Methods 13
7.1. Analysis Populations 13
7.2. Baseline Characteristics 14
7.3. Analyses of defined endpoints 14
7.3.1. Primary endpoint 14
7.3.2. Secondary endpoints 14
7.3.3. Tertiary/exploratory endpoints 17
7.3.4. Treatment compliance 17
8. General Considerations 18
8.1. Progression from Part A to Part B: 18
8.2. Screened Selection Design 18
8.3. Subgroup analyses 18
9. Independent Data Monitoring Committee (IDMC) and interim analyses 19
10. Analysis Programs 20
11. Analysis program locations 20
References 20
Appendix A: Detectable Effects (TOPARP-A) 21
Appendix B: Sample Size Calculations for Part A 22
Appendix C: Sample Size Calculations for Part B 24
Appendix D: Modified RECIST 25
Appendix E: Bone metastases evaluation 28

1. Introduction

This statistical analysis plan provides guidelines for the presentation, analysis and publication for the TOPARP trial. This plan, along with all other documents relating to the analysis of this trial, will be stored in the Statistical section of the Trial Master File.

It is intended that the results reported in these papers will not divert from the strategy set out here; subsequent papers of a more exploratory nature will not be bound by this strategy- though they are expected to follow the broad principles laid down for the main papers. The principles are not intended to curtail exploratory analysis (for example, to decide cut-points for categorisation of continuous variables), nor to prohibit sensible practices (for example, data transformation prior to analysis), but they are intended to establish the rules that will be followed, as closely as possible, when analysing and reporting the trial.

The analysis strategy will be available for information and can be viewed at request. Suggestions for subsequent analyses by journal editors or referees, will be considered carefully, and carried out as far as possible in line with the principles of this analysis strategy; if reported, the source of the suggestion will be acknowledged.

Procedures for monitoring data accuracy and data entry quality are provided in the central statistical monitoring plan and are not reproduced here.

1.1. Trial design

The study is divided into 2 parts. Part A was an efficacy screening stage for sensitive biomarkers which included 45 patients. Part A was designed as an open-labelled, non-randomised two stage design both to allow the activity of olaparib in unselected advanced CRPC patients to be evaluated and to permit the development of predictive biomarkers of response to olaparib. Patients were administered oral olaparib tablets, 400mg BID continuously until disease progression is documented and confirmed (i.e. confirmed radiographic progression) or withdrawal for another reason.

Part B is a subsequent open-label, single validation stage assessment of anti-tumour activity of olaparib in preselected patients with genomic defects associated with olaparib sensitivity previously identified in Part A. Part B will follow a phase II randomised "pick-the-winner" design: 88 patients will be randomly allocated in a 1:1 ratio to be administered oral olaparib tablets at doses of 300mg BID or 400mg BID with the goal of recommending an optimal dose of olaparib in mCRPC to be evaluated in further clinical trials. Patients will be in treatment until disease progression is documented and confirmed (i.e. confirmed radiographic progression) or withdrawal for another reason.

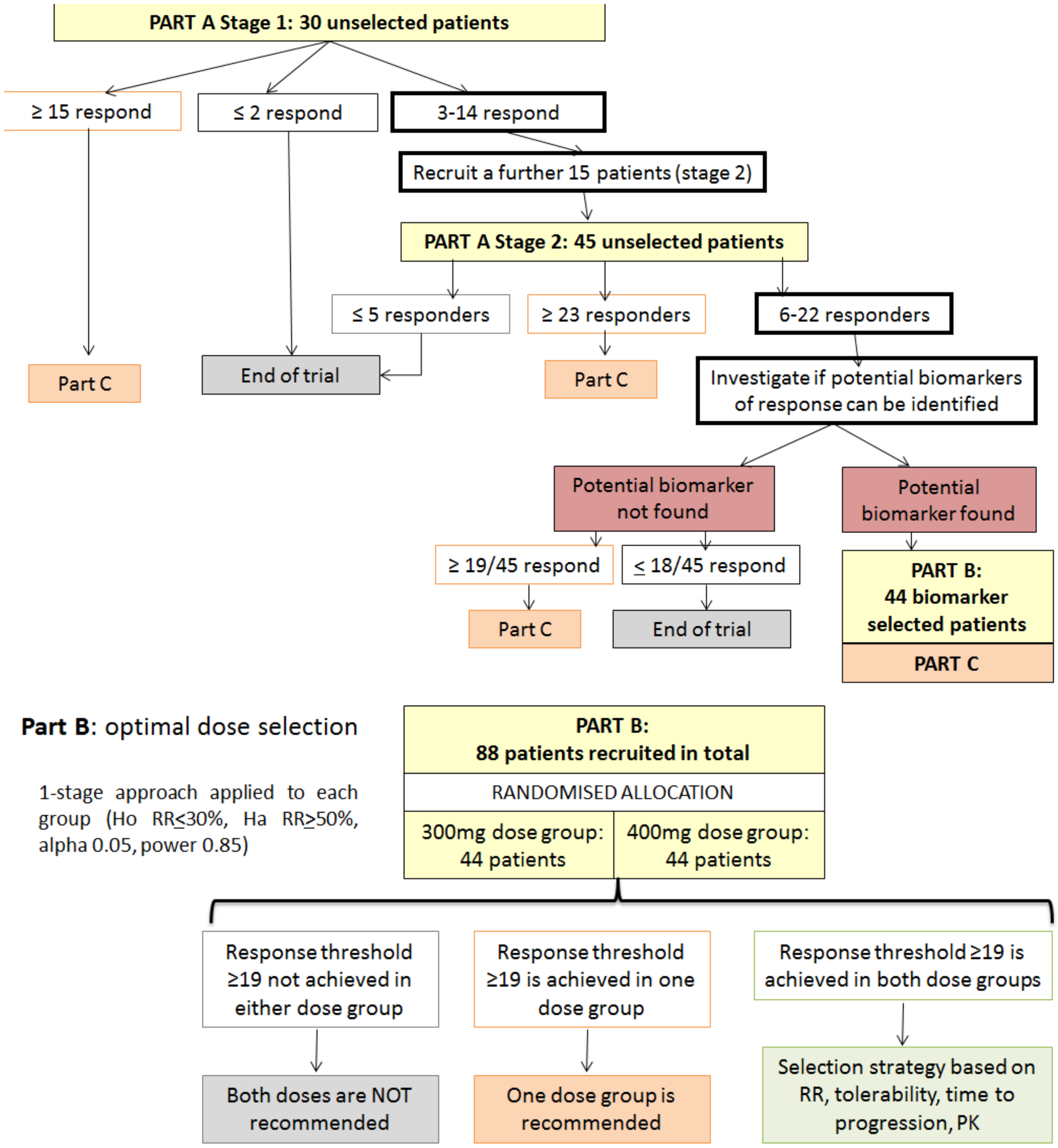

1.2. Study population

Subjects with advanced progressing CRPC who have had either one or two previous lines of taxane-based chemotherapy will be invited to participate in the TOPARP study.

Inclusion Criteria

Each patient must meet the following criteria to be enrolled in the study:

1. The subject is capable of understanding and complying with the protocol requirements and has signed the informed consent document.

2. Age ≥ 18 years.

3. Histologically confirmed adenocarcinoma of the prostate with archival tumour tissue available for molecular analyses. If the patient does not have a prior histological diagnosis then the planned

baseline fresh biopsy may be used for both the purpose of confirming the histological diagnosis prior to trial entry and for subsequent biomarker analysis. All patients must be willing to have fresh biopsies to obtain tumour tissue for biomarker analysis
Note: In part B, fresh biopsies are optional in those patients for whom sufficient archival tumour tissue is available for biomarker studies.

4. At least one but no more than two previous taxane-based chemotherapy regimens. If docetaxel chemotherapy is used more than once, this will be considered as one regime. Patients may have had prior exposure to cabazitaxel treatment.

5. At least 28 days since the completion of prior therapy, including major surgery, chemotherapy and other investigational agents. Additionally, clinically relevant sequelae should have resolved to grade 1 or less prior to recommencing treatment. For hormonal treatment and radiotherapy refer to the guidelines below:

- At least 28 days since the completion of prior flutamide treatment. Patients whose PSA did not decline in response to antiandrogens given as a second line or later intervention will only require a 14 days washout prior to Cycle 1, Day 1.
- At least 42 days since the completion of prior bicalutamide (Casodex) and nilutimide (Nilandron) treatment. Patients whose PSA did not decline for 3 or 4 months in response to antiandrogens given as second line or later intervention will require only a 14 day washout period prior to Cycle 1 Day1.
- At least 14 days from any radiotherapy with the exception of a single fraction of radiotherapy for the purposes of palliation (confined to one field) is permitted.

6. Documented prostate cancer progression as assessed by the investigator with one of the following:

- PSA progression defined by a minimum of three rising PSA levels with an interval of ≥ 1 week between each determination. The PSA value at the Screening visit should be ≥ 2 μg/L (2 ng/ml); patients on systemic glucorticoids for control of symptoms must have documented PSA progression by PCWG22 while on systemic glucocorticoids prior to commencing Cycle1 Day1 of treatment.
- Radiographic progression of soft tissue disease by modified RECIST criteria or of bone metastasis with two or more documented new bone lesions on a bone scan with or without PSA progression.

7. Surgically or medically castrated, with testosterone levels of < 50 ng/dL (< 2.0 nM). If the patient is being treated with LHRH agonists/antagonists (patient who have not undergone orchidectomy), this therapy must have been initiated at least 4 weeks prior to Cycle 1 Day 1 and must be continued throughout the study.

8. Eastern Cooperative Oncology Group (ECOG) Performance Status of ≤ 2 (Karnofsky Performance Status ≥ 50%)

9. Life expectancy > 12 weeks.

10. Patient must be able to swallow a whole tablet.

11. Patient and the patient's partner of childbearing potential, must agree to use medically accepted methods of contraception (e.g., barrier methods, including male condom, female condom, or diaphragm with spermicidal gel) during the course of the study and for 3 months after the last dose of study drug.

12. Agreeable to have all the biomarker studies including the paired fresh tumour biopsies.
Note: For Part B paired fresh tumour biopsies are optional however a fresh tumour biopsy will be required during pre-screening if no archival tumour tissue is available for molecular analyses.

13. Subjects must have a CTC count of ≥5 cells/7.5mls blood at screening confirmed by the central laboratory.
Note: For part B of the study, the minimum CTC count at screening is not mandatory if the patient has measurable disease according to modified RECIST1.1 (appendix E) and a lesion >2 cms in size at screening and screening PSA ≥ 2 μg/L (2 ng/ml).

14. Subjects must have adequate bone marrow, hepatic and renal function documented within 7 days of registration (TOPARP-A) / randomisation (TOPARP-B), defined as:
• Haemoglobin ≥9g/dL
• Absolute neutrophil count ≥1.5x109/L
• Platelets ≥100 x109/L
• Total bilirubin ≤ 1.5 x upper limit of normal (ULN) except for patients with known Gilbert's syndrome.
• Aspartate transaminase (AST) (SGOT) and alanine transaminase (ALT) (SGPT) ≤2.5 x ULN or ≤5 x ULN in the presence of liver metastases.
• Serum creatinine ≤1.5 x ULN or a calculated creatinine clearance >40mL/min for patients with creatinine levels above institutional normal. For GFR estimation, the Cockcroft and Gault equation should be used:
GFR = CrCl (ml/min) = (140 - age) × wt (kg)/(serum creatinine × 72)
• Albumin >25 g/dl

15. For Part B only – Subjects must have genomic defects associated with olaparib sensitivity as identified by next generation sequencing (anticipated to be approximately 30% of patients) confirmed by the central laboratory or, if there is a next generation sequencing result report from an external laboratory, the patient could be enrolled prior to confirmation of these results by the central laboratory **following approval by the Chief Investigator** (in such cases the tumour sample will still be tested at the central laboratory).

Exclusion Criteria
Patients who meet any of the following criteria will be excluded from the study:

1. Surgery, or local prostatic intervention (excluding a prostatic biopsy) less than 28 days of Cycle 1 Day 1.

2. Less than 28 days from any active anticancer therapy or investigational agents. For hormonal treatment and radiotherapy refer to the guidelines outlined in the inclusion criteria.

3. Prior treatment with a PARP inhibitor, platinum, cyclophosphamide or mitoxantrone chemotherapy.
Note: If patients have received a treatment with the listed compounds for non-prostate cancer, ICR-CTSU should be contacted to facilitate discussions with the CI as to the patients' suitability.

4. Uncontrolled intercurrent illness including, but not limited to, active infection, symptomatic congestive heart failure (New York Heart Association Class III or IV heart disease), unstable angina pectoris, cardiac arrhythmia, uncontrolled hypertension or psychiatric illness/social situations that would limit compliance with study requirements.

5. Any acute toxicities due to prior chemotherapy and / or radiotherapy that have not resolved to a NCI-CTCAE v4.02 grade ≤1 with the exception of chemotherapy induced alopecia and grade 2 peripheral neuropathy.

6. Malignancy within the previous 2-years with a > 30% probability of recurrence within 12 months with the exception of non-melanoma skin cancer, in-situ or superficial bladder cancer.

7. Patients with myelodysplastic syndrome/acute myeloid leukaemia.

8. Patients with known symptomatic brain metastasis are not suitable for enrolment.
Patients with asymptomatic, stable, treated brain metastases are eligible for study entry.

9. Patients with symptomatic or impending cord compression unless appropriately treated beforehand and clinically stable and asymptomatic.

10. Patients who have experienced a seizure or seizures within 6 months of study treatment or who are currently being treated with cytochrome P450 enzyme inducing anti-epileptic drugs for seizures (use of anti-epileptic drugs to control pain is allowed in patients not suffering from seizures unless drug is excluded due to CYP3A4 induction - phenytoin, carbamazepine, phenobarbital

11. Patients receiving any of the following classes of inhibitors of CYP3A4:
− Azole antifungals
− Macrolide antibiotics
− Protease inhibitors

12. Patients with gastrointestinal disorders likely to interfere with absorption of the study medication.

13. Initiating bisphosphonate therapy or adjusting bisphosphonate dose/regimen within 30 days prior to Cycle 1 Day 1. Patients on a stable bisphosphonate regimen are eligible and may continue.

14. Presence of a condition or situation, which, in the investigator's opinion, may put the patient at significant risk, may confound the study results, or may interfere significantly with patient's participation in the study.

15. The subject is unable or unwilling to abide by the study protocol or cooperate fully with the investigator or designee

2. Trial Objectives

2.1. Primary Objective

The primary objective of this study is to evaluate the anti-tumour activity of olaparib in patients with advanced CRPC who have progressed following one or two chemotherapy regimens including taxane-based regimens.

2.2. Secondary Objectives

The secondary objectives are to evaluate:

- Radiological progression-free survival (rPFS)
- Time to radiological progression
- Progression free survival (PFS)
- Overall survival
- Time to PSA progression
- Duration of PSA response (decline of ≥50%)
- PSA objective response (PSA-ORR)
- The utility of CTCs as an early indication of benefit from PARPi therapy
- Further characterise the safety profile of olaparib in advanced CRPC patients'
- TOPARP-B only - drug exposure (pharmacokinetics) with the two dose levels of 300mg or 400mg of the tablet formulation and correlation of drug exposure with safety events and antitumour activity.
- TOPARP-B only – evaluation of the two dose levels and recommendation of dose to investigate further.

2.3. Tertiary/exploratory Objectives

To evaluate clinical benefit as indicated by an improvement in pain measurements (BPI-SF).

- To evaluate DW-MRI imaging in selected patients and to correlate these results with PSA, CTC and radiological responses by conventional imaging.
- TOPARP-B only – to evaluate the response rate following dose-escalation from 300mg to 400mg twice daily.

3. Sample size & power

The aim is to identify a sensitive subgroup with a 50% response rate on the basis that high response rates are necessary to be confident of demonstrating a survival benefit, the primary goal of the phase II strategy (See Appendix A and B).

3.1. Part A (comprised of stages 1 & 2)

The screening trial will involve all comers (unselected patients) and is an open-labelled, multi-centre two-stage design based on H0: $p0=0.05$; Ha: $p1=0.20$; $\alpha=0.02$ and $\beta=0.10$. It will recruit a **maximum of 45 patients** (30 in stage 1 and 15 in stage 2) with an initial efficacy level of 20% and an inefficacy level of 5%, to allow for the fact that only a small subset of patients may be sensitive to olaparib. This part will evaluate the anti-tumour activity of olaparib in unselected CRPC patients and will identify potential biomarkers of response.

3.1.1. Stage 1

The first stage will enrol 30 patients and when all 30 have been followed up for a minimum of 6 months, or have been assessed for the primary endpoint, an interim analysis will take place to evaluate the response rate. In the absence of any safety concerns recruitment will continue to part A stage 2 whilst response data on the first 30 patients accumulates.

Given the efficacy level of 20% and inefficacy level of 5% the trial will be terminated with the conclusion of inefficacy if there are **2 or fewer responses**. If there are **15 or more responses** in the first stage, broad efficacy will be concluded and no more patients will be entered and Part C will commence in unselected patients. If between **3 to 14 patients respond**, patient accrual will continue to a total of 45 patients (stage 2) and an investigation into the identification of a potential biomarker of response will be carried out.

3.1.2. Stage 2

If the trial progresses to stage 2, a further 15 patients will be recruited, giving a total of 45 patients for part A. An interim analysis will be triggered once 45 patients have been followed up for a minimum of 6 months, or alternatively we observe 23 or more responses. The recruitment will be halted. If **23 or more** patients respond, Part C will be initiated. If **5 or fewer patients** respond, the null hypothesis will be accepted and the study will be terminated. If **between 6 and 22 patients respond**, an investigation into the identification of potential biomarkers of response will be carried out. If a potential biomarker subgroup is found, Part B will be undertaken. If a subgroup is not found, then Part C will be undertaken if at least 19 of the patients have responded, otherwise the study will be terminated since sufficient activity has not been observed based on H0: p0=0.3; Ha ; p1=0.5; α=0.059 and β=0.11.

3.1.3. Deriving a classifier for identification of tumours with high susceptibility to olaparib

All patients participating in TOPARP-A will have their archival tissue, fresh tumour biopsies (and circulating tumour cells collected prior to starting olaparib. At the outset, the primary biomarkers identified for evaluation include PTEN loss by IHC and FISH, *ETS* gene rearrangements by FISH and RAD51 foci formation.

The goal will be to develop a classifier which identifies a group with a response rate of 40% or more on the basis that the levels for inefficacy and efficacy are set at 30% and 50% respectively.

The biomarker defined subgroup satisfying the above criteria will be selected for validation in Part B. If convincing evidence arises from other translational studies of olaparib or other PARP inhibitors the IDMC will be asked to advise whether or not an analysis should take place.

3.2. Part B

The biomarker status of patients registered in Part B will be evaluated and only patients with the presence of the putative biomarker subgroup identified in Part A will be entered. Part B will follow a phase II randomised "pick-the-winner" design: eighty-eight patients will be randomised to receive either 400 mg BID or 300 mg BID.

Each dose group will be assessed independently for antitumour activity: the minimum desired activity for each dose group will be assessed by a one-stage A'Hern design, which assumes H_0: p_0=0.3; H_a ; p_1=0.5; α=0.05 and β=0.15. If **19 or more** of the **44 patients** in one dose group respond (43%), then the dose group is considered successful. If **18 or less** patients in one dose

group respond, the dose group will be considered unsuccessful. If the 400 mg BID dose group meets the criteria for "success", the biomarker identified in Part A will be considered validated.

In the case where both dose groups are successful, the play-the-winner selection strategy proposed by Simon, Wittes and Ellenberg [1] modified as suggested by Yap et al [2], is applied to select the superior dose group. If the difference in response rate is larger than 10%, the dose group with higher response rate will be recommended. A variety of scenarios, shown in Appendix C, demonstrate that the probability of choosing the arm with the highest true response if the difference in response rates is larger than 10% was greater than 80%. If the difference in response rate is ≤10%, dose selection will be based on additional criteria outlined in 8.2.

4. Randomisation/ recruitment procedures

Patients who meet the eligibility and screening requirements (including pre-dose biopsy, blood samples and CTCs) will be enrolled to the study. For part A there will be only 1 treatment arm, olaparib 400mg BID. For part B, patients will be randomised to receive 300mg or 400mg of olaparib BID on a 1:1 basis. Treatment allocation in part B is by minimisation with a random element, with screening CTC value as a balancing factor.

5. Endpoints

5.1. Primary Endpoint

Response will be defined on the basis of the following outcomes; if any of these occur patients will be considered to have responded:

- Objective response by modified RECIST (baseline lymph node size must be ≥2cm to be considered a target lesion) or
- Conversion of circulating tumour cell count (CTC) from ≥5/7.5 ml blood at baseline to <5/7.5 ml blood nadir confirmed by a second consecutive value obtained four or more weeks later or
- PSA decline of ≥50% (according to the PCWG2)

Evaluable patients with no confirmed response as defined above will be classified as non-responders.

5.2. Secondary Endpoints

- Radiographic Progression Free Survival (rPFS)
- Time to Radiographic Progression
- Progression Free Survival (PFS)
- Overall Survival (OS)
- Time-to-PSA Progression
- Duration of PSA response
- Percentage change in PSA from baseline to 12 weeks (or earlier if discontinued therapy) and maximum PSA decline while on treatment
- Proportion of patients with conversion of CTC count to <5/7.5ml blood nadir
- Safety and tolerability profile of olaparib in patients with metastatic CRPC

5.3. Tertiary/exploratory Endpoints

- Proportion of patients achieving pain improvement
- Impact of olaparib treatment on bone metastases imaged by DW-MRI in selected patients (optional) and to correlate these results with PSA, CTC and radiologic responses by conventional imaging.
- TOPARP-B only – response rate following dose-escalation from 300mg to 400mg twice daily.

6. Data completeness and consistency

6.1. Patient flow through trial

As part of the principal analysis of the primary endpoint for publication a CONSORT diagram will be provided. This will include the number of patients available for the efficacy population and safety population and the number of patients who discontinued with trial treatment and reasons for discontinuation.

6.2. Compliance with the assessments

Compliance with trial treatment, safety and follow-up will be assessed by the IDMC (via tables and listings in the IDMC report) and through regular data monitoring of the trial.

6.3. Consistency of reporting

Consistency of reporting by centres will be assessed by the IDMC (via tables and listings in the IDMC report) and through regular data monitoring of the trial.

6.4. Missing data

Missing data will be investigated by centre and overall for key variables. Should any patterns emerge then these will be examined. It is expected that the majority of missing data will be at random (except for variables where assessments are not routine done at the centre, e.g. CgA, CgB results) and therefore, it is not expected that imputation methods will be used for individual patients or variables.

7. Analysis Methods

7.1. Analysis Populations

Analysis populations will be defined as follows:

- *Intention to treat* (ITT): This population includes all patients enrolled into the study regardless of whether they are later found to be ineligible, a protocol violator, never treated or not evaluated. Patients for whom the primary endpoint cannot be evaluated will be treated as non-responders.
- *Evaluable-patient* population: Patients who meet all of the inclusion and exclusion criteria and who start trial treatment are considered evaluable, unless they discontinue treatment prior to 12 weeks for reasons which aren't drug or disease related.
- Per protocol population: This population contains all enrolled patients who received at least 1 cycle of olaparib without major protocol violations
- *Safety* population: This population includes all enrolled patients who received at least 1 dose of olaparib.

The analysis of the primary outcome will use the evaluable-patient population with sensitivity analyses in the ITT and per protocol population. All other analyses will use the ITT population. The

ITT analysis will treat non-evaluable patients as non-responders. The safety population will be used to characterise the safety profile of olaparib in advanced CRPC patients.

7.2. Baseline Characteristics

Baseline demographics and clinical characteristics will be tabulated overall and by phase/stage/dose group (Part B only) of the trial using the ITT population. Numbers (with percentages) for binary, categorical variables and ordered categories plus means (and standard deviations), or medians (with lower and upper quartiles) for continuous variables will be presented. Graphical representation will also be used as appropriate.

7.3. Analyses of defined endpoints

7.3.1. Primary endpoint

RECIST response is reported as a category on eCRFs, but individual disease measurements are also provided and these will be used to check the classification (RECIST v1.1 response criteria as outlined in a protocol appendix). Discrepancies will be queried.

Response will be defined on the basis of the following outcomes; if any of these occur patients will be considered to have responded:

- Objective response (CR or PR) by modified RECIST or
- Conversion of circulating tumour cell count (CTC) from ≥5/7.5 ml blood at baseline to <5/7.5 ml blood nadir confirmed by a second consecutive value obtained four or more weeks later or
- PSA decline of ≥50% (according to the PCWG2)

In Part B, results will be presented by dose group.

Response will be evaluated 6 months post registration (Part A)/post-randomisation (Part B), however the primary analysis may be conducted prior to this if all patients without 6 months follow up have been evaluated for the primary endpoint or have experienced a treatment failure and discontinued study treatment. The last value of PSA, CTC count, CT scan and bone scan on or up to four weeks before the date of first study treatment will be used as the baseline value for this assessment. The response rate will be presented along with its exact two-sided 95% confidence interval. In Part B, results will be presented by dose group. For analyses including any patient for whom response has not been confirmed the patient will by default be treated as a non-responder.

7.3.2. Secondary endpoints

The following endpoints will be assessed **6 months post registration (Part A)/post-randomisation (Part B)**. However, decisions made within the trial to comply with the adaptive design will not necessarily require 6 months follow up on all patients, e.g. it may be possible to enter patients into the second stage of Part A when it is clear between 3 and 14 responses will occur in the first stage even though not all 30 patients have 6 months follow up. In Part B, all endpoints will be presented separately for each dose group.

PSA response and **PSA progression** are defined according to the consensus guidelines of the *Prostate Cancer Clinical Trials Working Group 2*:

- PSA partial response is defined as a ≥ 50% decline in PSA value from cycle1 day 1 (baseline). This PSA decline must be confirmed to be sustained by a second PSA value obtained 4 or more weeks later.

- PSA progression date is defined as the date that a ≥ 25% increase and an absolute increase of ≥ 2 ng/mL above the nadir is documented, which is confirmed by a second consecutive value obtained four or more weeks later. The first PSA reading will be obtained at week 12.

Pattern of PSA Responses
Waterfall plots will be presented (as per PCWG2 recommendations) that show the percentage of change in PSA from baseline to 12 weeks (or earlier for those who discontinue therapy), as well as the maximum decline in PSA that occurs at any point after treatment. Waterfall plots provide a broader display of PSA changes than dichotomised responses. In Part B, the dose group will be displayed using different colours. Longitudinal spaghetti plots of PSA values over time will also be presented to explore the different patterns of response, in Part B this will be conducted for each dose group. Longitudinal models of PSA values over time, such as mixed effect models, may be explored.

CTC Response
The proportion of patients with a CTC conversion to <5/7.5 ml blood at nadir (confirmed by a second consecutive value four or more weeks later) will be presented along an exact two-sided 95% confidence interval. Waterfall plots of CTC falls will also be presented that show the percentage change in CTC counts from baseline to 12 weeks as well as maximal CTC count declines that occur at any point after treatment. In Part B, the dose group will be displayed using different colours. Longitudinal spaghetti plots of CTC values over time will also be presented to explore the different patterns of response in Part B this will be conducted for each dose group. CTC response's association with PFS and OS will be examined via landmark analyses (to compare OS or PFS between CTC responders and non-responders at a given time point). Longitudinal models of CTC values over time, such as mixed effect models, may be explored.

The following endpoints will be evaluated using survival analysis methods (Kaplan-Meier estimation for survival/probability curves):

Radiographic Progression Free Survival (rPFS)
Radiographic progression free survival (rPFS) will be defined by either RECIST progression and /or progression on bone scan (Appendix D and E). It will be measured from the date of trial entry to the first occurrence of radiographic progression or death from any cause. If no event exists, then rPFS will be censored at the last scheduled disease assessment on study.

Time to Radiographic Progression
Time to radiographic progression (progression defined by either RECIST progression and /or progression on bone scan) will be measured from the date of trial entry to the first occurrence of radiographic progression. Death from prostate cancer or any other cause without prior radiographic evidence of progression will not count as an event. If no event exists, then time to radiographic progression will be censored at the last scheduled disease assessment on study or date of death whichever occurs earlier.

Progression Free Survival (PFS)
Progression free survival will be measured from the date of trial entry until radiographic progression, unequivocal clinical progression or death. If no event exists, then PFS will be censored at the last scheduled disease assessment on study.

Time-to-PSA Progression
For patients who have achieved a ≥ 50% decrease from the cycle 1 day 1 (baseline), the PSA progression date is defined as the date that a ≥ 25% increase and an absolute increase of ≥ 2 ng/mL above the nadir is documented. This must be confirmed by a second consecutive value obtained 4 or more weeks later. For patients without a PSA decrease of this magnitude or no decrease at all, PSA progression date is defined as the date that a ≥ 25% increase and an absolute increase of ≥ 2 ng/mL above the baseline is documented. This must also be confirmed by a second consecutive value 4 or more weeks later.

Duration of PSA response
Duration of PSA response is calculated from the time the PSA value first declines by at least 50% of the cycle 1 day 1 (baseline) value (must be confirmed by a second value) until the time there is an increase of 25% of PSA nadir, provided the absolute increase is at least 2 ng/mL. The increase must be confirmed by a second consecutive measurement that is at least 25% above the nadir. If the PSA never shows a 25% increase over the nadir value, then the patient will be censored at the last PSA measurement. Duration of PSA response will be summarised by the median and presented along its 95% confidence interval. Time to PSA response on the duration of response (e.g. by fitting left-truncated models of adjusting by time to response) may be explored.

Duration of response
Duration of response as defined above is calculated form the time response is confirmed until radiographic progression, unequivocal clinical progression or death. If progression free and alive at the end of follow-up, the patient will be censored. Median duration with its 95%CI will be presented. The impact of time to response on the duration of response (e.g. by fitting left-truncated models of adjusting by time to response) may be explored.

Overall Survival (OS)
OS will be measured from the date of trial entry to the date of death (whatever the cause). Survival time of living patients will be censored on the last date a patient is known to be alive or lost to follow-up.

Safety Endpoints
Safety analysis will be summarised using the safety population. Extent of exposure to study drug will be summarised and details will be provided. Treatment emergent adverse events (AEs) are those events that occur or worsen on or after first dose of study drug up through 30 days post last dose. The worst grade from screening or cycle 1 day 1 will be used as the baseline assessment. Adverse events will be graded according to the NCI-CTCAE v4.02. All SAEs and AEs will be coded according to the latest version of MedDRA available. Adverse events will be summarised using preferred term (PT) and system organ class (SOC) by grade, according to the worst grade experienced. In addition, most frequently observed AEs will be summarised using PT and SOC. In the summary of AE, an AE occurs more than once within a SOC and PT will be counted only once

using the worst grade experienced. Serious AE and deaths observed within 30 days of the last dose of study treatment will be provided in a listing. The proportion of patients with a worse treatment emerging grade greater than grade 1 and grade 3 will be displayed. In Part B, these adverse event summaries will be displayed by dose group.

All adverse events resulting in discontinuation, dose modification, dosing interruption, and/or treatment delay of study drug will also be listed and tabulated by preferred term. In Part B, these will be tabulated by dose group. Clinical laboratory test results will be collected pre-treatment and through 30 days post last dose of study treatment. All laboratory test results will be classified according to the NCI CTCAE v4.02 criteria. Standard reference ranges will be used for missing or discrepant normal ranges. Baseline laboratory test values are the results from the last blood samples drawn on or prior to the first day of study treatment. On-study laboratory test values are those results from blood samples drawn a day after the first study treatment up until 30 days after the last dose of study treatment.

7.3.3. Tertiary/exploratory endpoints

Pain palliation

Pain palliation will be assessed using the Brief Pain Inventory's (BPI Short Form) worst pain intensity score and average score. A composite of the four pain items (worse, least, average, right now) will be presented as supplemental information. Change in BPI score from baseline will be assessed at 6 months post registration. A reduction of two points in the worst pain score relates to an improvement of pain. The proportion of patients achieving pain improvement will be presented along with its exact 2-sided 95% confidence interval. In Part B, this will be displayed by dose group. The analgesic relief received will be described.

Impact of treatment with olaparib on bone metastases imaging by DW-MRI

Diffusion-weighted MRI scan will be offered to patients recruited in part A and part B and we expect approximately 50% acceptance rate. This analysis will be purely exploratory and will examine whether response to olaparib expressed by changes in ADCs produced by the DW-MRI scan correlate with 1) PSA decline, 2) CTC conversion and 3) radiological responses coming from conventional imaging. In 20 patients it is possible to detect a correlation coefficient of 0.6 which is equivalent to the parameter explaining 36% of the variation using a 2-sided 5% significance level.

Dose Escalation

Patients in Part B allocated to the 300mg BID dose group will be allowed to dose-escalate to 400mg BID upon confirmation of disease progression, providing it is considered clinically indicated in each case and that the patient did not require a dose reduction while on therapy. The rate of response following dose escalation will be calculated using the same definitions for response specified in section 7.3.1. Baseline measurements for the dose escalation analysis will be from the date of dose escalation visit.

7.3.4. Treatment compliance

An investigation of treatment compliance of olaparib will involve a summary, either by tabulations or graphically of the following:

- Numbers enrolled
- eligible/ineligible (including reasons)
- discontinuations prior to protocol completion (including reasons)

- doses missed, dose reductions & delays (including reasons)

8. General Considerations

8.1. Progression from Part A to Part B:

All patients participating in this trial will have their archival tissue, fresh tumour biopsies and CTCs collected prior to starting olaparib. For full details of the biomarker analyses, refer to the Biomarker Statistical Analysis Plan v1.0 (18/02/2015) and the TOPARP-A exploratory statistical analysis plan v1.0 (09/07/2016).

8.2. Screened Selection Design

Based on the ideas of Yap et al [2], determining the recommended olaparib dose group in Part B will use a screened selection design. Activity of a dose group if initially determined using a one-stage A'hern design. A dose group is considered to be positive if ≥19 patients achieve a response.

- **Scenario 1:** Both doses negative
  Neither dose group are recommended for further investigation.
- **Scenario 2:** One dose group positive
  This dose group is recommended for further investigation.
- **Scenario 3:** Both dose groups positive
  If the difference in response rate between dose groups is ≥10% then this dose is recommended for further investigation.
  When the difference in response rate is <10%, progression free survival will initially be used to determine a dose for further investigation. If the hazard ratio for a dose, as measured using a Cox proportional hazards model of progression free survival, is <0.66 then this dose will be investigated for further study (A hazard ratio of 0.66/1.5 has 50% power and 5% significance and is felt to be a clinically meaningful difference). If the difference in PFS is smaller than this, then the dose with longest duration of response will be selected for further investigation (median time of duration of response or time to progression will be compared).

8.3. Subgroup analyses

A pre-planned exploratory subgroup analysis will be conducted for TOPARP-B based on the fact that patients with prostate cancers with several different genomic aberrations, that have slightly different impacts on PARP inhibitor sensitization, were recruited to this trial [3,4]. This will include an evaluation of the primary endpoint (response rate) as well as of the secondary endpoints (rPFS and PFS). The commonest different genomic aberration tumour subsets recruited to TOPARP-B will be sub-classified and outcome data reported for each sub-group. The subgroups will be defined, based on their frequency, as:

- **Sub-group 1:** Prostate cancers with BRCA gene deleterious aberrations, including both BRCA2 and BRCA1. This is anticipated to be the commonest sub-group; BRCA1/2 loss of function results in homologous recombination defects (HRD) in DNA repair, with bi-allelic loss of function of both these genes arguably sensitizing more than other genomic aberrations. This largest sub-group will be further sub-categorised into cancers with, and without, clear evidence of loss of both *BRCA1/2* gene alleles based on next generation sequencing (including HRD signatures; immuno-histochemistry for BRCA1/2 proteins is not available).
- **Sub-group 2:** Prostate cancers with *ATM* gene aberrations; these tumours do not cause typical homologous recombination defects (HRD) although they have been proven to sensitize to PARP inhibition, albeit less than HRD. This is anticipated to be one of the commoner sub-

groups. This sub-group will be further sub-categorised into cancers with, and without, clear evidence of deleterious aberrations of both *ATM* gene alleles based on next generation sequencing and immunohistochemistry for ATM.

- **Sub-group 3:** Prostate cancers with *CDK12* gene alterations. This is anticipated to be one of the commoner sub-groups. This sub-group will be further sub-categorised into cancers with, and without, clear evidence of deleterious aberrations of both *CDK12* gene alleles based on next generation sequencing and specifically the described exome/genome bimodal tandem duplication signature [5,6]; immunohistochemistry for CDK12 protein is not available).
- **Sub-group 4**: Prostate cancers with *PALB2* gene alterations. This is likely to be a smaller subgroup. This sub-group will be further sub-categorised into cancers with, and without, clear evidence of deleterious aberrations of both *PALB2* gene alleles based on next generation sequencing (including HRD signatures).
- **Sub-group 5:** Prostate cancers with less common deleterious genomic aberrations in DNA repair genes not listed above. These data will be presented as a sub-group and if indicated based on disease biology as smaller subsets.
- **Sub-group 6:** Prostate cancers found to have a genomic signature of homologous recombination based on exome, or low pass whole genome, next generation sequencing.

Patients will be allocated in these subgroups according to the mutations/deletions described in the pre-screening biomarker reports, regardless of whether these were based on archival or fresh biopsy tumour sequencing. If a patient has deleterious aberrations in more than one qualifying DNA repair gene, that patient will be assigned to a subgroup ranked based on 1) the presence of biallelic loss of function rather than monoallelic loss; and 2) aberrations previously reported as pathogenic versus those that may not be definitely known to be pathogenic or of unknown significance. If after applying these criteria a patient may still be assigned to more than one group, he will be included in the analyses of all the relevant subgroups. These subgroup analyses will be conducted combining both dose groups (300 mg and 400 mg).

9. Independent Data Monitoring Committee (IDMC) and interim analyses

An Independent Data Monitoring Committee will meet in confidence at regular intervals, firstly to review the safety analysis following the first 5 and 10 patients recruited and subsequently every 6 months (or at intervals determined by them) to review accrual, safety and efficacy data. A report of the findings and recommendations will be produced following each meeting. This report will be submitted to the Trial Management Group and Trial Steering Committee, the main REC and the MHRA, as required.

The replacement of patients who withdraw from the study or who are unevaluable for reasons deemed not to be treatment related will be based on the advice of the committee. The committee will review the results of each interim analysis (progression from stage 1 to stage 2 and progression to part B) and will be asked to advise. These analyses will be supplied in strict confidence by the trial statistician to the IDMC together with any other analyses that the IDMC may request. No results will be made available to investigators or any other party unless the IDMC determines that it would be unethical to withhold the interim results.

The main criterion for early stopping of the trial by the Trial Steering Committee, upon advice from the IDMC and endorsement from the Trial Management Group, will be that evidence from the trial and from other sources suggests a) proof beyond reasonable doubt that for all, or for some types

of patient, the treatment regimen is clearly indicated or contra-indicated in terms of response rate and/or toxicity, and b) evidence exists that might reasonably be expected to influence routine clinical practice.

The IDMC will, however, reserve the right to release any data on outcome or side-effects through the Trial Steering Committee to the Trial Management Group (and if appropriate to participants) if it determines at any stage that the combined evidence from this and other studies justifies it.

10. Analysis Programs

Analysis will be conducted using Stata version 13 (or subsequent versions). Toxicities will be coded according to the latest version of MedDRA available.

11. Analysis program locations

All programs will be stored in the analyses folder for TOPARP (N:\ANALYSES\Toparp) on the ICR-CTSU server. Only the TOPARP trial statistician(s), ICR-CTSU IT staff, Director and Deputy Directors of ICR-CTSU will be able to see the analysis folder. Programmes will be stored under the type of analysis e.g. 'IDMC' or 'Primary analyses'. All official analysis reports that are to be circulated externally of ICR-CTSU will be password protected. Hard copies of reports will be stored securely in the statistical section of the TOPARP trial master file held in a locked fire proof cupboard with restricted access.

References

[1] Simon, R., Wittes, R.E. and Ellenberg, S.S., 1985. Randomized phase II clinical trials. *Cancer treatment reports*, *69*(12), pp.1375-1381.

[2] Yap, C., Pettitt, A. and Billingham, L., 2013. Screened selection design for randomised phase II oncology trials: an example in chronic lymphocytic leukaemia. *BMC medical research methodology*, *13*(1), p.87.

[3] McCabe MT, Low JA, Daignault S, et al: 2006. Inhibition of DNA methyltransferase activity prevents tumorigenesis in a mouse model of prostate cancer. *Cancer Res* 66:385-92

[4] Murai, J. et al. 2012. Trapping of PARP1 and PARP2 by clinical PARP inhibitors. *Cancer Res*. 72, 5588–5599

[5] Wu YM, Cieślik M, Lonigro RJ, Vats P, Reimers MA, Cao X, et al, 2018. Inactivation of CDK12 Delineates a Distinct Immunogenic Class of Advanced Prostate Cancer. *Cell;173:*1770–1782.e14.

[6] Popova T, Manié E, Boeva V, Battistella A, Goundiam O, Smith NK, et al, 2016. Ovarian cancers harbouring inactivanting mutations in CDK12 display a distinct genomic instability pattern characterized by large tandem duplications. *Cancer Res. 76(7)*1882-91.

Appendix A: Detectable Effects (TOPARP-A)

It is possible to calculate detectable effects in the sensitive group (80% power, y-axis) based on the proportion of sensitive patients (individual lines) and the response rate (x-axis) in the insensitive subgroup. Also shown is the probability a subgroup effect is detected (dashed lines), with 80% power this probability cannot exceed 80%. The fall in the probability of detecting a subgroup effect with increasing response rate in insensitive patients is therefore balanced by an increase in the probability of detecting an overall effect. Thus, in the extreme, if the response rate in insensitive patients is 45% and the response rate in sensitive patients is about 50% a conclusion of overall efficacy will be reached with 80% power, for subgroups of size 30% or more. Using cut offs for Part A of a_0=15/30, a_1=23/45, as intended.

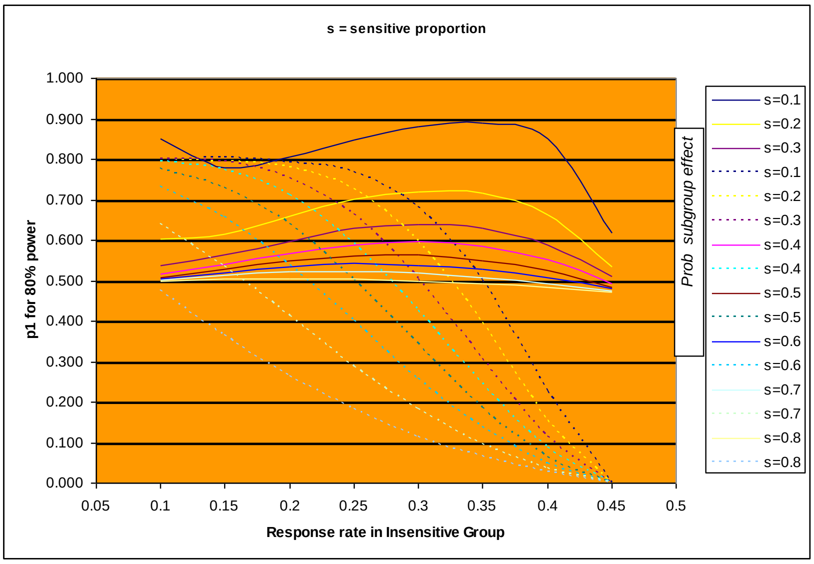

Using cut offs for Part A of a_0=18/30, a_1=27/45 the probability of detecting a subgroup effect if the sensitive group is large (>50%) can be improved.

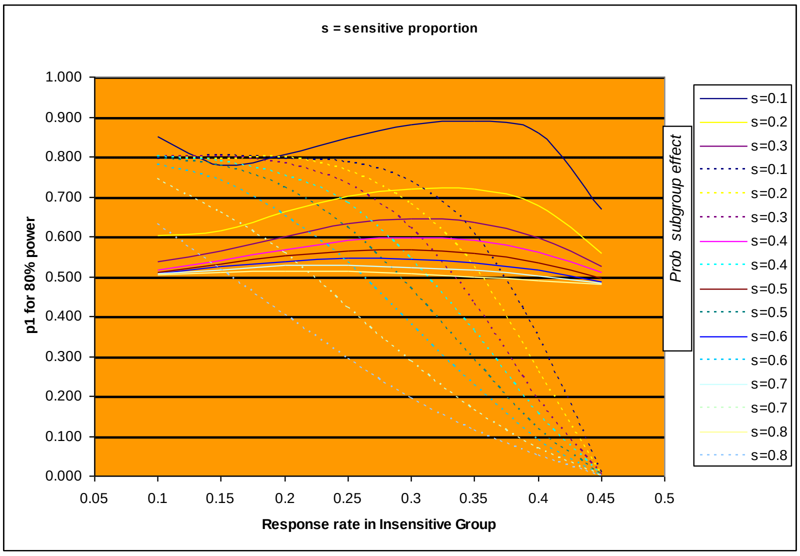

Appendix B: Sample Size Calculations for Part A

TOPARP sample size calculations, **Part A**: alpha 2.2%, Power 90%

Ho : Null Hypothesis Response rate (p0) is 5%

Probability reject **0.97818** *alpha* 2.2%

Reject first stage 0.81218 (Probability of early termination, sum of values in light blue)

Reject second stage 0.166 (sum of values in dark blue)

sum 0.97818

N First	po											
	0.05											
0	0.214639	N second stage										
1	0.338903	0	1	2	3	4	5	6	7	8		15
2	0.258637	0.46329	0.3657562	0.13475	0.03073	0.00485	0.00056	4.9E-05	3.3E-06	1.8E-07	……	3.1E-20
3	0.12705	0.059	0.046	0.017	0.004	0.001	0	0	0	0		0
4	0.045136	0.021	0.017	0.006	0.001	0	0	0	0	0		0
5	0.012353	0.006	0.005	0.002	0	0	0	0	0	0		0
6	0.002709	0.001	0.001	0	0	0	0	0	0	0		0
7	0.000489	0	0	0	0	0	0	0	0	0		0
8	7.4E-05	0	0	0	0	0	0	0	0	0		0
9	9.52E-06	0	0	0	0	0	0	0	0	0		0
10	1.05E-06	0	0	0	0	0	0	0	0	0		0
11	1.01E-07	0	0	0	0	0	0	0	0	0		0
12	8.39E-09	0	0	0	0	0	0	0	0	0		0
13	6.11E-10	0	0	0	0	0	0	0	0	0		0
14	3.91E-11	0	0	0	0	0	0	0	0	0		0
15	2.19E-12											
16	1.08E-13											
17	4.69E-15											
18	1.78E-16											
….												
26	3.33E-30											
27	2.59E-32											
28	1.46E-34											
29	5.31E-37											
30	9.31E-40											

Stage I 2.31E-12
probability of incorrect stopping

H1 : Null Hypothesis Response rate (p1) is 20%

Probability reject **0.10318** *Power* 89.7%

Reject first stage 0.04418 (Probability of early termination, sum of values in light blue)

Reject second stage 0.059 (sum of values in dark blue)

N First	po											
	0.2											
0	0.001238	N second stage										
1	0.009285	0	1	2	3	4	5	6	7	8		15
2	0.033656	0.03518	0.1319414	0.2309	0.25014	0.1876	0.10318	0.04299	0.01382	0.00345	……	3.3E-11
3	0.078532	0.003	0.01	0.018	0.02	0.015	0.008	0.003	0.001	0		0
4	0.132522	0.005	0.017	0.031	0.033	0.025	0.014	0.006	0.002	0		0
5	0.172279	0.006	0.023	0.04	0.043	0.032	0.018	0.007	0.002	0.001		0
6	0.179457	0.006	0.024	0.041	0.045	0.034	0.019	0.008	0.002	0.001		0
7	0.153821	0.005	0.02	0.036	0.038	0.029	0.016	0.007	0.002	0.001		0
8	0.110559	0.004	0.015	0.026	0.028	0.021	0.011	0.005	0.002	0		0
9	0.067564	0.002	0.009	0.016	0.017	0.013	0.007	0.003	0.001	0		0
10	0.035471	0.001	0.005	0.008	0.009	0.007	0.004	0.002	0	0		0
11	0.016123	0.001	0.002	0.004	0.004	0.003	0.002	0.001	0	0		0
12	0.006382	0	0.001	0.001	0.002	0.001	0.001	0	0	0		0
13	0.002209	0	0	0.001	0.001	0	0	0	0	0		0
14	0.000671	0	0	0	0	0	0	0	0	0		0
15	0.000179											
16	4.19E-05											
17	8.63E-06											
18	1.56E-06											
….												
26	7.53E-15											
27	2.79E-16											
28	7.47E-18											
29	1.29E-19											
30	1.07E-21											

Stage I 0.000231
probability of incorrect stopping

TOPARP sample size calculations, **Part A**, rule to be applied if no subgroup is found:

Ho : Null Hypothesis Response rate (p0) is 30%

Reject first stage 0.94505 alpha 5.5%

(sum of values in light blue)

N	p0	N	p0	N	p0
	0.3				
0	1.07E-07	16	0.0896266	32	1.3E-08
1	2.06E-06	17	0.0655253	33	2.2E-09
2	1.95E-05	18	0.0436835	34	3.3E-10
3	0.00012	19	0.0266043	35	4.5E-11
4	0.000538	20	0.0148224	36	5.4E-12
5	0.00189	21	0.0075624	37	5.6E-13
6	0.005401	22	0.0035357	38	5E-14
7	0.012895	23	0.0015153	39	3.9E-15
8	0.026251	24	0.0005953	40	2.5E-16
9	0.046252	25	0.0002143	41	1.3E-17
10	0.071361	26	7.065E-05	42	5.3E-19
11	0.09731	27	2.131E-05	43	1.6E-20
12	0.118162	28	5.87E-06	44	3.1E-22
13	0.12855	29	1.475E-06	45	3E-24
14	0.125927	30	3.371E-07		
15	0.111535	31	6.991E-08		

Ha : Alternate Hypothesis Response rate (p1) is 50%

Reject first stage 0.11635 Power 88.4%

N	p1	N	p1	N	p1
	0.5				
0	2.84E-14	16	0.0183782	32	0.00207
1	1.28E-12	17	0.0313511	33	0.00082
2	2.81E-11	18	0.0487684	34	0.00029
3	4.03E-10	19	0.0693024	35	9.1E-05
4	4.23E-09	20	0.0900931	36	2.5E-05
5	3.47E-08	21	0.1072537	37	6.1E-06
6	2.31E-07	22	0.1170041	38	1.3E-06
7	1.29E-06	23	0.1170041	39	2.3E-07
8	6.13E-06	24	0.1072537	40	3.5E-08
9	2.52E-05	25	0.0900931	41	4.2E-09
10	9.07E-05	26	0.0693024	42	4E-10
11	0.000288	27	0.0487684	43	2.8E-11
12	0.000817	28	0.0313511	44	1.3E-12
13	0.002075	29	0.0183782		
14	0.004743	30	0.0098017		
15	0.009802	31	0.0047428		

Appendix C: Sample Size Calculations for Part B

Setting 1: alpha=0.05 per arm (overall 0.10), power=0.85 (original Part B design)

a) A'hern 1-stage design: recruit 44 patients per arm, arm positive if 19 respond, 19/44=43.2%

b) If both arms are positive, i.e. both 300mg and 400mg have 19 or more responses, apply SWE to pick the winner: select the arm with more responses. With 44 patients, assuming that the true response rate for each arm is over 40% (both arms are +), the probability of choosing the arm with the highest response rate[1] would be (under different scenarios):

Probability of choosing the arm with the highest response rate

	400		300			
Scenario	#resp	%resp	#resp	%resp	Difference	Pick the winner prob
1	19	43.2%	19	43.2%	0.0%	0.50
2	20	45.5%	19	43.2%	2.3%	0.58
3	21	47.7%	19	43.2%	4.5%	0.67
4	22	50.0%	19	43.2%	6.8%	0.74
5	24	54.5%	19	43.2%	11.4%	0.86
6	30	68.2%	19	43.2%	25.0%	0.99
7	21	47.7%	21	47.7%	0.0%	0.50
8	22	50.0%	21	47.7%	2.3%	0.58
9	24	54.5%	21	47.7%	6.8%	0.74
10	26	59.1%	21	47.7%	11.4%	0.86
11	28	63.6%	21	47.7%	15.9%	0.93
12	30	68.2%	21	47.7%	20.5%	0.98
13	22	50.0%	22	50.0%	0.0%	0.50
14	24	54.5%	22	50.0%	4.5%	0.66
15	26	59.1%	22	50.0%	9.1%	0.80
16	28	63.6%	22	50.0%	13.6%	0.90
17	30	68.2%	22	50.0%	18.2%	0.96

c) The previous table show that, for those scenarios, if small differences are expected (below 10%), the probability of choosing arm with the highest true response rate is less than 80%. In this case, we could apply the modified selection criteria following Yap:

If both arms are positive, and the difference between arms is less than 10%, the dose with largest duration of response will be selected for further investigation (median time of duration of response or time to TTP will be compared).

[1] Computed using online calculator http://www.cct.cuhk.edu.hk/stat/phase2/Randomized.htm

Appendix D: Modified RECIST

The primary efficacy objective to this trial is based on tumour assessment. For the purpose of this study, definitions of soft tissue tumour assessments will be based on a Modified Response Evaluation Criteria in Solid Tumours 1.1 (RECIST1.1). Pertinent points are summarised below.

Evaluation of measurable and non-measurable lesions

1. **Measurable lesions** – lesions that can be accurately measured in at least one dimension with the longest diameter $\geq$ 20 mm using conventional techniques or $\geq$ 10 mm, with a spiral CT scan.
2. **Non-measurable lesions** – all other lesions, including small lesions (longest diameter $\leq$ 10 mm, with a spiral CT scan), i.e., blastic bone lesions, leptomeningeal disease, ascites, pleural or pericardial effusion, inflammatory breast disease, lymphangitic involvement of skin or lung, abdominal masses/abdominal organomegaly identified by physical exam that is not measurable by reproducible imaging techniques.
3. Tumour lesions situated in a previously irradiated area, or in an area subjected to other loco-regional therapy, should not be included in assessment of response, but should be assessed for progression.
4. For this study, bone lesions are not considered "non-measurable" lesions for RECIST. Bone lesions will be assessed by bone scan only.
5. All measurements should be taken and recorded in metric notation, using a ruler or calipers. Preferably, all screening evaluations should be performed as closely as possible to the beginning of treatment and never more than 4 weeks before the beginning of treatment.
6. The same method of assessment and the same technique should be used to characterise each identified and reported lesion at baseline and during follow up.
7. Clinical lesions will only be considered measurable when they are superficial (e.g., skin nodules and palpable lymph nodes). For the case of skin lesions, documentation by colour photography, including a ruler to estimate the size of the lesion is recommended.

Methods of measurement:

1. CT and MRI are the best currently available and reproducible methods to measure target lesions selected for response assessment. Conventional CT and MRI should be performed with cuts of 10 mm or less in slice thickness. Spiral CT should be performed using a 5-mm contiguous reconstruction algorithm. This applies to tumours of the chest, abdomen and pelvis. Head and neck tumours and those of extremities usually require specific protocols.
2. Lesions on chest X-ray are acceptable as measurable lesions when they are clearly defined and surrounded by aerated lung, however CT scans are preferable.
3. Ultrasound (US), endoscopy and laparoscopy should not be used to measure tumour lesions. It is however a possible alternative to clinical measurements of superficial palpable lymph nodes, subcutaneous lesions and thyroid nodules. Ultrasound may also be used to confirm the complete disappearance of superficial lesions usually assessed by clinical examination.
4. Cytology and histology can be used to differentiate between PR and CR in rare cases (e.g., after treatment to differentiate between residual benign lesions and residual malignant lesions.

Baseline documentation of target and non-target lesions

1. All measurable lesions up to a maximum of 2 lesions per organ and 5 lesions in total representative of all involved organs should be identified as target lesions and recorded and measured at baseline and at the time points specified in the protocol.
2. Target lesions should be selected on the basis of their size (lesions with the longest diameter) and their suitability for accurate repeated measurements.
3. A sum of the longest diameters (LD) for all target lesions will be calculated and reported as the baseline sum of LD. The baseline sum LD will be used as reference by which to characterise the objective tumour response.
4. All other lesions (or sites of disease) should be identified as non-target lesions and should also be recorded at baseline. Non-target lesions will also be monitored throughout the study, and an overall assessment of response will be made. Details of any new lesions will also be recorded. It is possible to record multiple nontarget lesions involving the same organ as a single item on the case record form (e.g. "multiple enlarged pelvic lymph nodes" or "multiple liver metastases").

Response Criteria

Response criteria	Evaluation of target lesions
Complete Response (CR)	Disappearance of all target lesions
Partial Response (PR)	At least 30% decrease in the sum of LD of target lesions, taking as reference the baseline sum of LD.
Progressive Disease (PD)	At least 20% increase in the sum of LD of target lesions, taking as reference the smallest sum LD recorded since the treatment started or the appearance of one or more new lesions.
Stable Disease (SD)	Whether sufficient shrinkage to qualify for PR nor sufficient increase to qualify for PD, taking as reference the smallest sum LD since the treatment started.

Responses in patients with both target and non-target disease

Target lesions	Non-target lesions	New Lesions	Overall response
CR	CR	No	CR
CR	Non-CR/nonPD	No	PR
CR	Not evaluated	No	PR
PR	Non-PD or not all evaluated	No	PR
SD	Non-PD or not all evaluated	No	SD
Not all evaluated	Non-PD	No	NE
PD	Any	Yes or No	PD
Any	PD	Yes or No	PD
Any	Any	Yes	PD

CR = complete response, PR= partial response, SD = stable disease, PD= progressive disease, NE= inevaluable

Responses in patients with only non-target disease

Non-target Lesions	New Lesions	Overall Response
CR	No	CR
Non-CR/nonPD	No	Non-CR/nonPD*
Not all evaluated	No	NE
Unequivocal PD	Yes or No	PD
Any	Yes	PD

CR = complete response, PR= partial response, SD = stable disease,
PD= progressive disease, NE= inevaluable

Reproduced from: http://www.recist.com/recist-comparative/01.html and Eisenhauer EA, Therasse P, Bogaerts J et al. New response evaluation criteria in solid tumours: Revised RECIST guideline (version 1.1). Eur J Cancer 2009; 45:228-247.

Patients with a global deterioration of health status requiring discontinuation of treatment without objective evidence of disease progression at that time should be classified as having "symptomatic deterioration". Every effort should be made to document the objective progression even after discontinuation of treatment. In some circumstances it may be difficult to distinguish residual disease from normal tissue. When the evaluation of complete response depends on this determination, it is recommended that the residual lesion be investigated (fine needle aspirate/biopsy) to confirm the complete response status.

Confirmation

To be assigned a status of PR or CR, changes in tumour measurements must be confirmed by repeat assessments that should be performed no less than 4 weeks after the criteria for response are first met.

In the case of SD, follow-up measurements must have met the SD criteria at least once after study entry at a minimum interval (in general, not less than 12 weeks) that is defined in the study protocol.

Evaluation of best overall response

The best overall response is the best response recorded from the start of the treatment until disease progression/recurrence (taking as reference for PD the smallest measurements recorded since the treatment started). In general, the patient's best response assignment will depend on the achievement of both measurement and confirmation criteria.

Duration of overall response

The duration of overall response is measured from the time measurement criteria are met for CR or PR (whichever status is recorded first) until the first date that recurrence or PD is objectively documented, taking as reference for PD the smallest measurements recorded since the treatment started.

Duration of stable disease

SD is measured from the start of the treatment until the criteria for disease progression are met, taking as reference the smallest measurements recorded since the treatment started.

Appendix E: Bone metastases evaluation

The first bone scan should not be performed before 12 weeks post commencement of treatment unless clinically indicated as there may be evidence of a flare response early on which may confuse the response evaluation. A patient will be considered to have progressed on bone scan if the following criteria are met:

- If the first bone scan with 2 new lesions is observed ≤13 weeks from the point of Cycle1 Day1 and is confirmed by a follow up scan ≥ 6 weeks later showing 2 additional new lesions (i.e. a total of ≥ 4 new lesions compared with the baseline bone scan).
- The first bone scan with ≥ 2 new lesions compared with baseline is observed > 13 weeks from Cycle 1 Day1 and the new lesions are verified on the next bone scan ≥ 6 weeks later (i.e. a total of ≥ 2 new lesions compared with baseline). If the second scan confirms progression the data of the progression is the date of the first bone scan rather than the second confirmatory scan.

Measurable Disease Evaluation:

Changes in nodal and visceral disease should be recorded using the modified RECIST criteria defined in Appendix E.

Radiological Criteria for Ascribing Disease Progression

Evidence of Progression	Confirmation	Action
Bone Disease<br>Appearance of two or more new bone lesions on bone scan	≥ 2 new lesions at the first scheduled reassessment ≤13 weeks from Cycle 1 Day 1 compared with baseline must be confirmed by a second scan performed 6 or more weeks later.<br>Confirmatory scans should show an additional 2 new lesions compared to the first post treatment scan (i.e. a total of ≥ 4 new lesions compared with the baseline bone scan).	Investigators are highly encouraged to maintain the patient's treatment with study medication unless progression is confirmed.
Appearance of two or more new bone lesions on bone scan	≥ 2 new lesions at the first scheduled reassessment >13 weeks from Cycle 1 Day 1 compared with baseline must be confirmed by a second scan performed 6 or more weeks later.<br>Confirmatory scans should confirm the presence of the 2 new lesions compared the baseline scan. (i.e. a total of ≥ 2 new lesions compared with the baseline bone scan).	Investigators are highly encouraged to maintain the patient's treatment with study medication unless progression is confirmed.
Soft Tissue Disease as defined by RECIST on CT/MRI	Progression at any scheduled reassessment ≥ 12 weeks does not need to be confirmed.	Investigators are highly encouraged to maintain the patient's treatment with study medication unless radiological progression is observed.
